# Supplementary material for: Fish CDK2 recruits Dtx4 to degrade TBK1 through ubiquitination in the antiviral response
Source: eLife. 2026 Jan 14;13:RP98357. doi: 10.7554/eLife.98357 (PMC12803515; doi:10.7554/eLife.98357)
Supplement: Figure 1—source data 1. [file elife-98357-fig1-data1.zip › Figure 1-source data 1/Figure 1-source data.pdf]

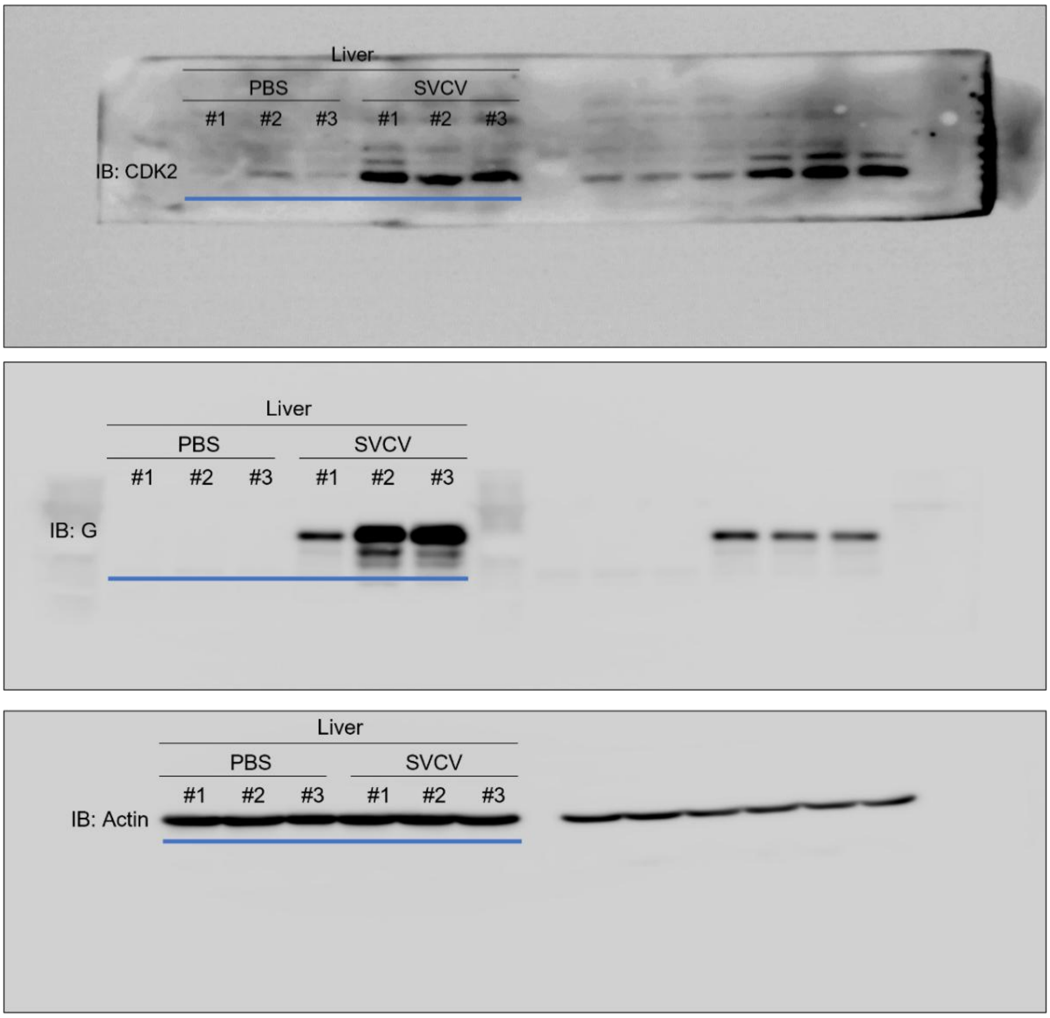


Figure 1, Source Data 1. Original membranes corresponding to Figure 1, panel E (Liver). Each membrane is labelled with the relevant information. The blue lines indicate the corresponding bands.

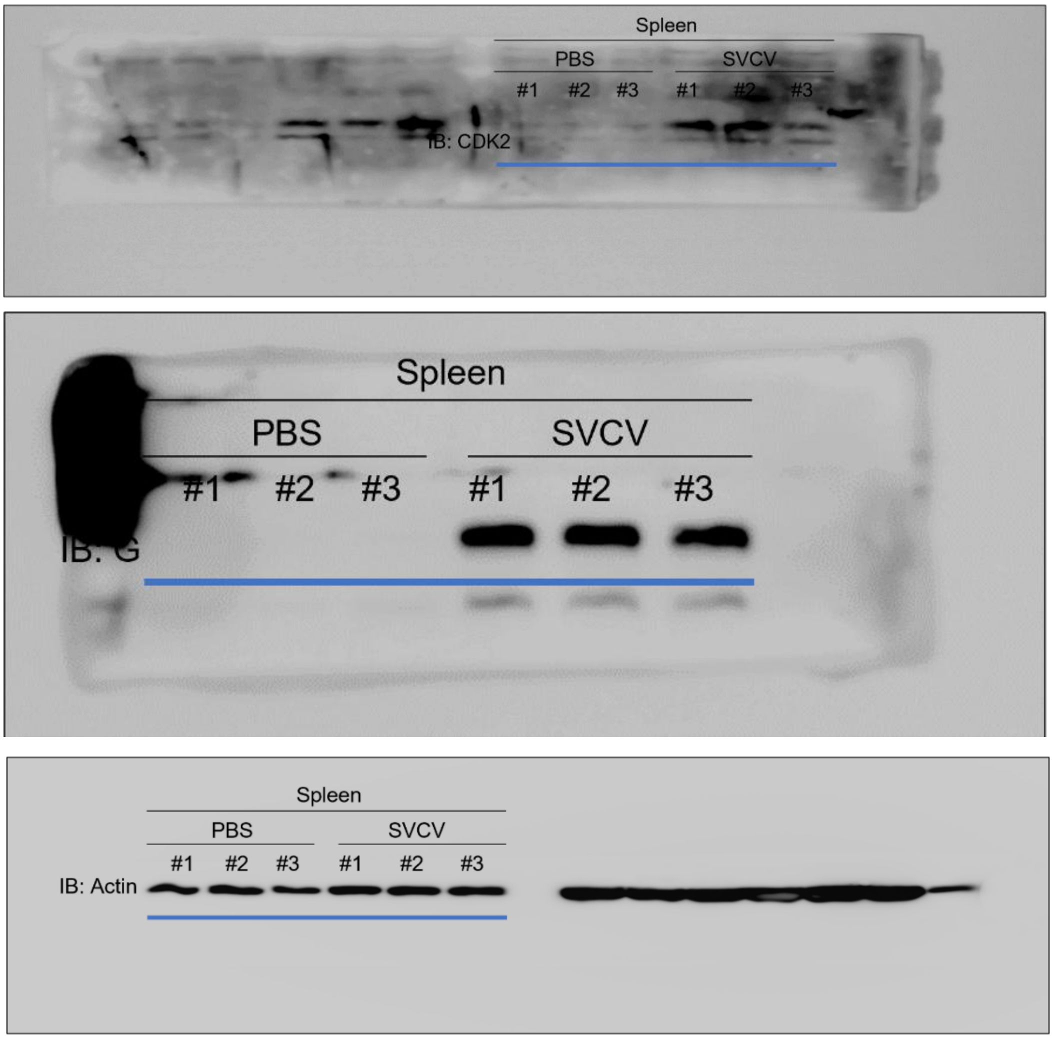


Figure 1, Source Data 1. Original membranes corresponding to Figure 1, panel E (Spleen). Each membrane is labelled with the relevant information. The blue lines indicate the corresponding bands.

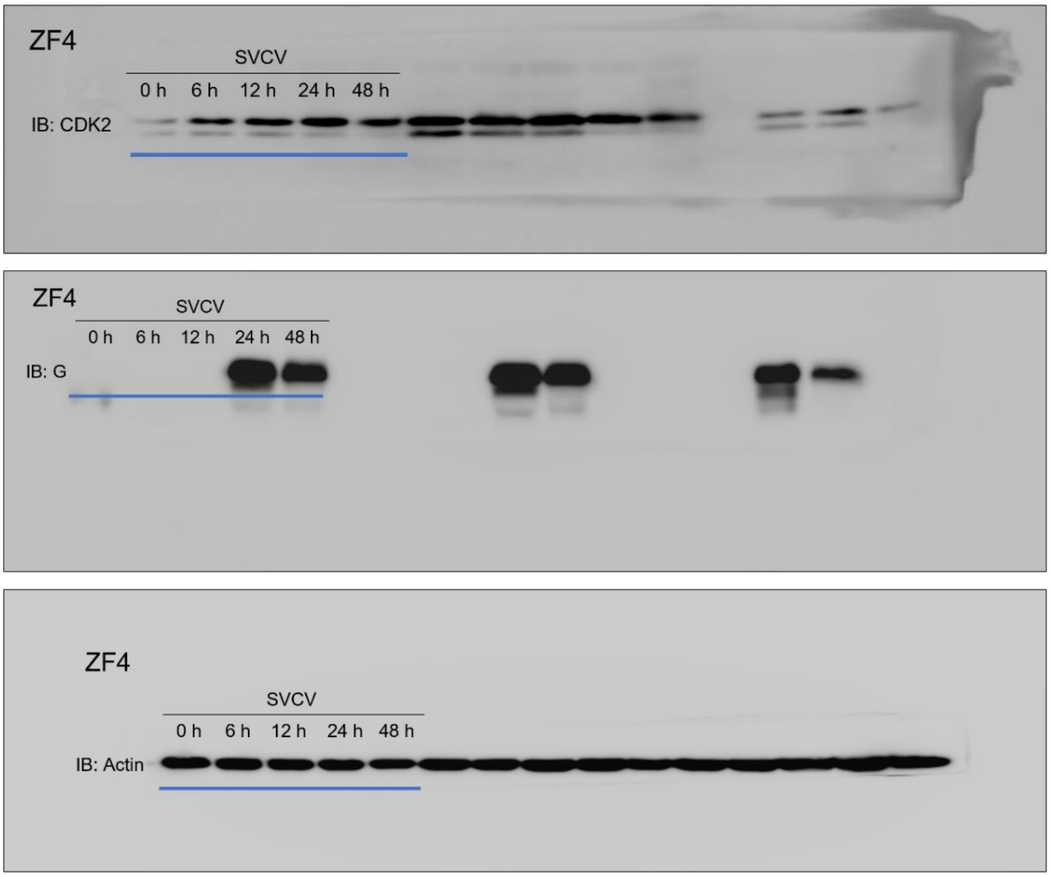


Figure 1, Source Data 1. Original membranes corresponding to Figure 1, panel F (ZF4 cells). Each membrane is labelled with the relevant information. The blue lines indicate the corresponding bands.

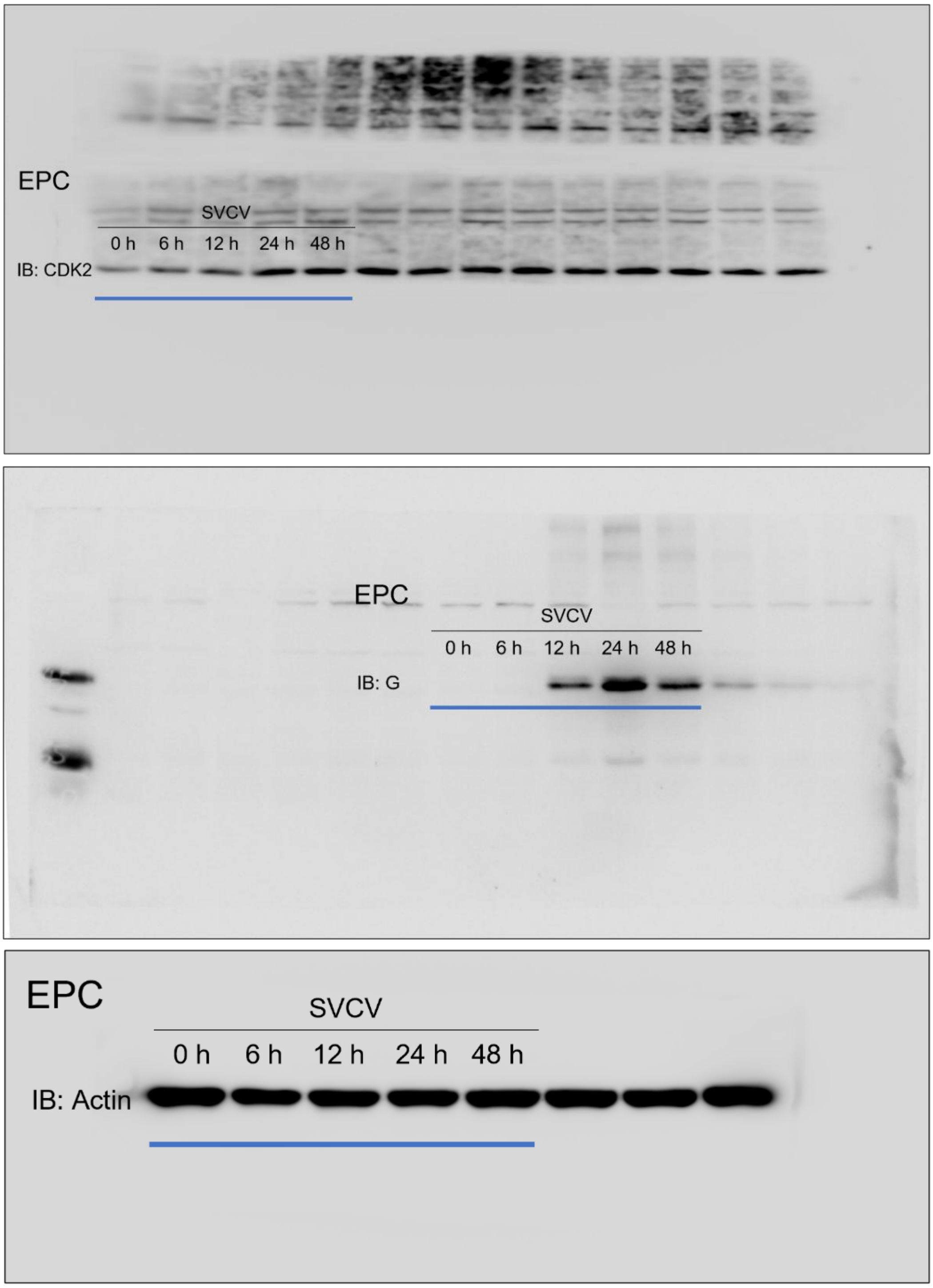


Figure 1, Source Data 1. Original membranes corresponding to Figure 1, panel F (EPC cells). Each membrane is labelled with the relevant information. The blue lines indicate the corresponding bands.
