# Supplementary figures and images for: Fish CDK2 recruits Dtx4 to degrade TBK1 through ubiquitination in the antiviral response (part 1 of 3)
Source: eLife. 2026 Jan 14;13:RP98357. doi: 10.7554/eLife.98357 (PMC12803515; doi:10.7554/eLife.98357)

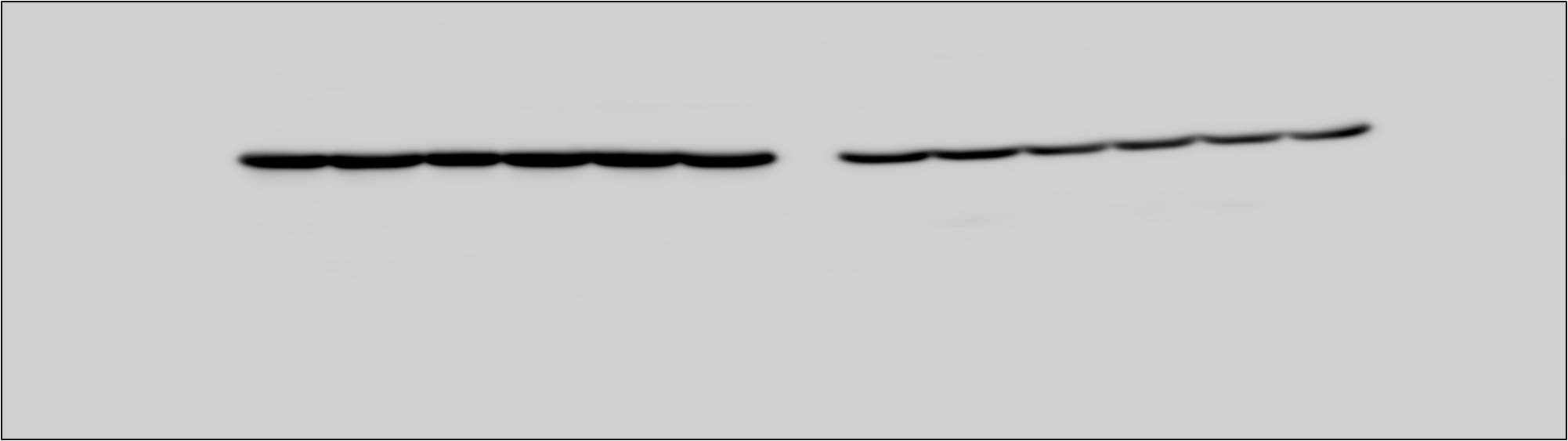

Supplement: Figure 1—source data 2. [file elife-98357-fig1-data2.zip › Figure 1-source data 2/1 E Liver-Actin.tif]

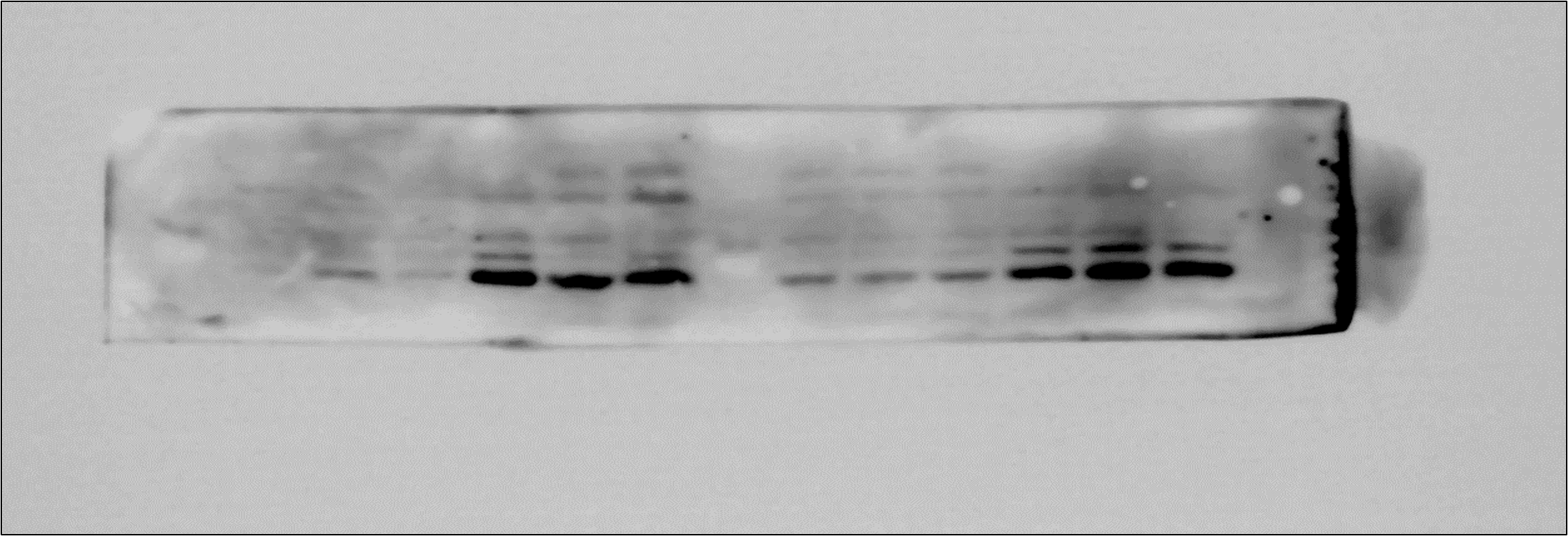

Supplement: Figure 1—source data 2. [file elife-98357-fig1-data2.zip › Figure 1-source data 2/1 E Liver-CDK2.tif]

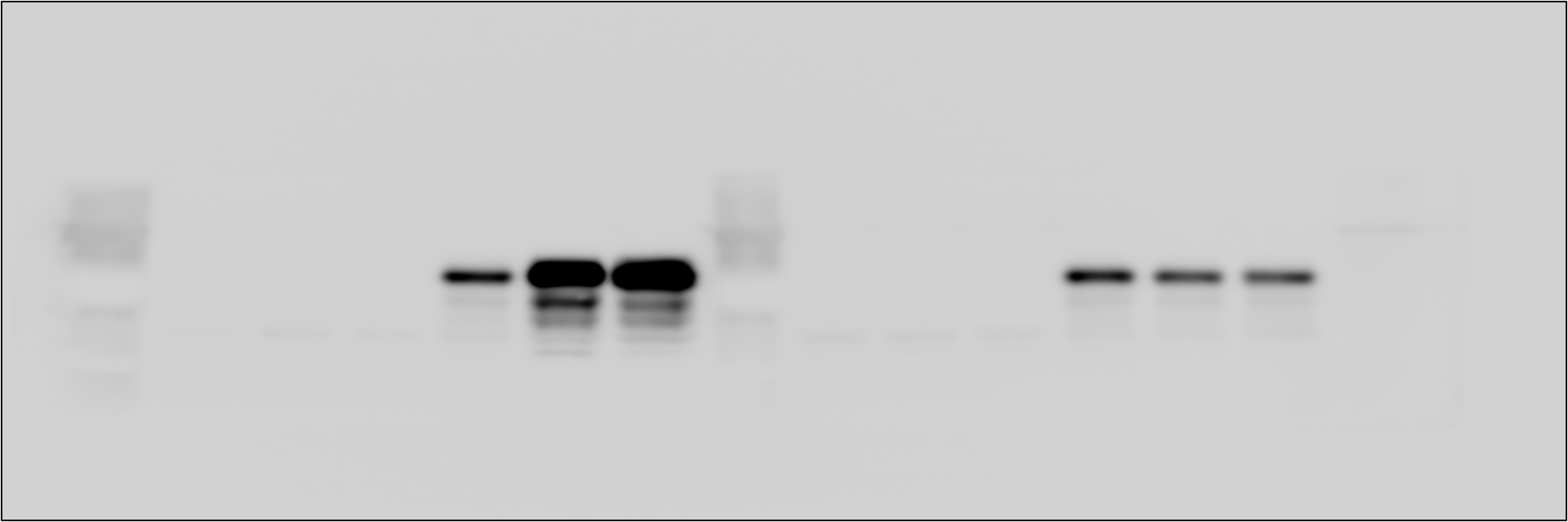

Supplement: Figure 1—source data 2. [file elife-98357-fig1-data2.zip › Figure 1-source data 2/1 E Liver-G.tif]

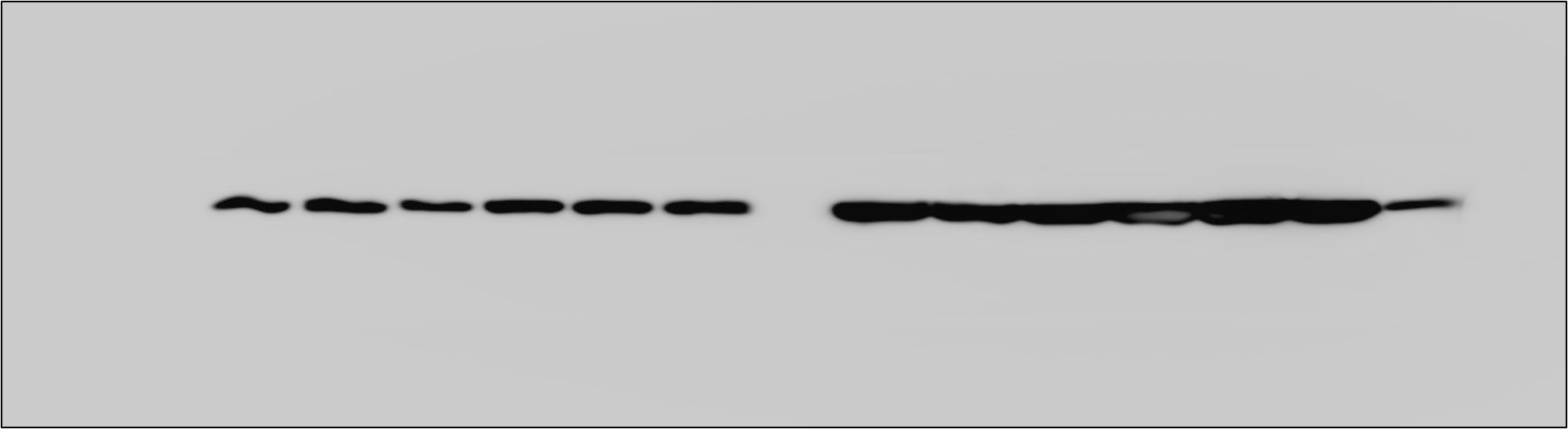

Supplement: Figure 1—source data 2. [file elife-98357-fig1-data2.zip › Figure 1-source data 2/1 E Spleen-Actin.tif]

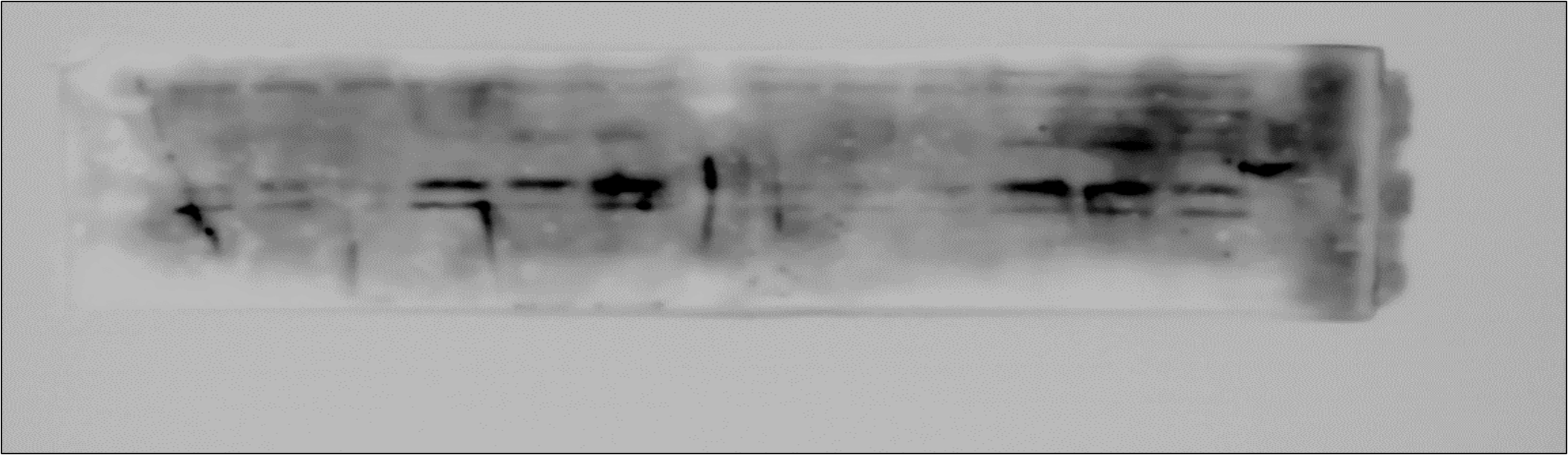

Supplement: Figure 1—source data 2. [file elife-98357-fig1-data2.zip › Figure 1-source data 2/1 E Spleen-CDK2.tif]

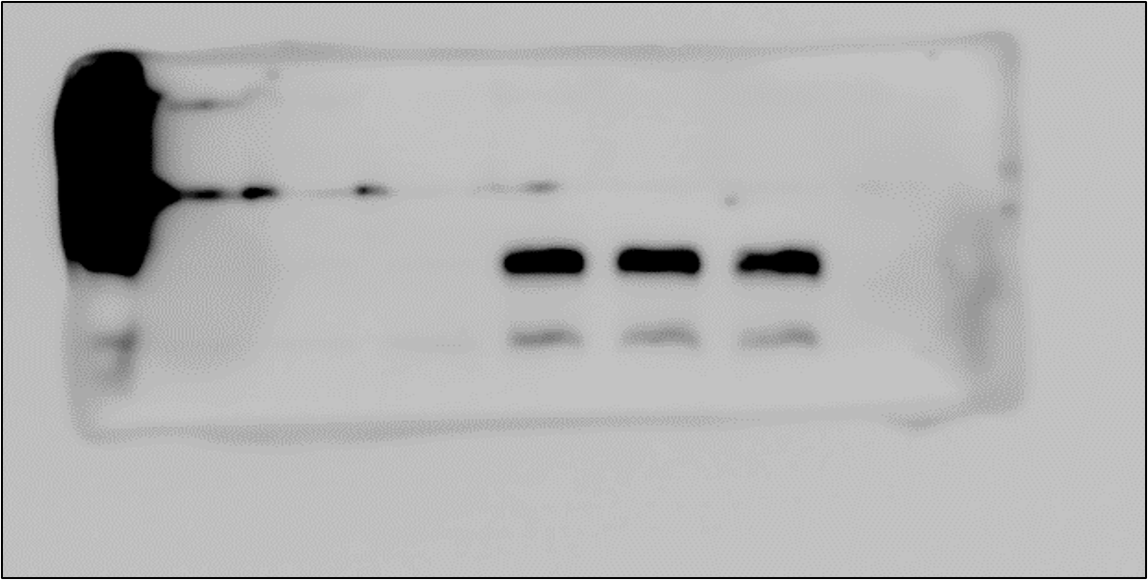

Supplement: Figure 1—source data 2. [file elife-98357-fig1-data2.zip › Figure 1-source data 2/1 E Spleen-G.tif]

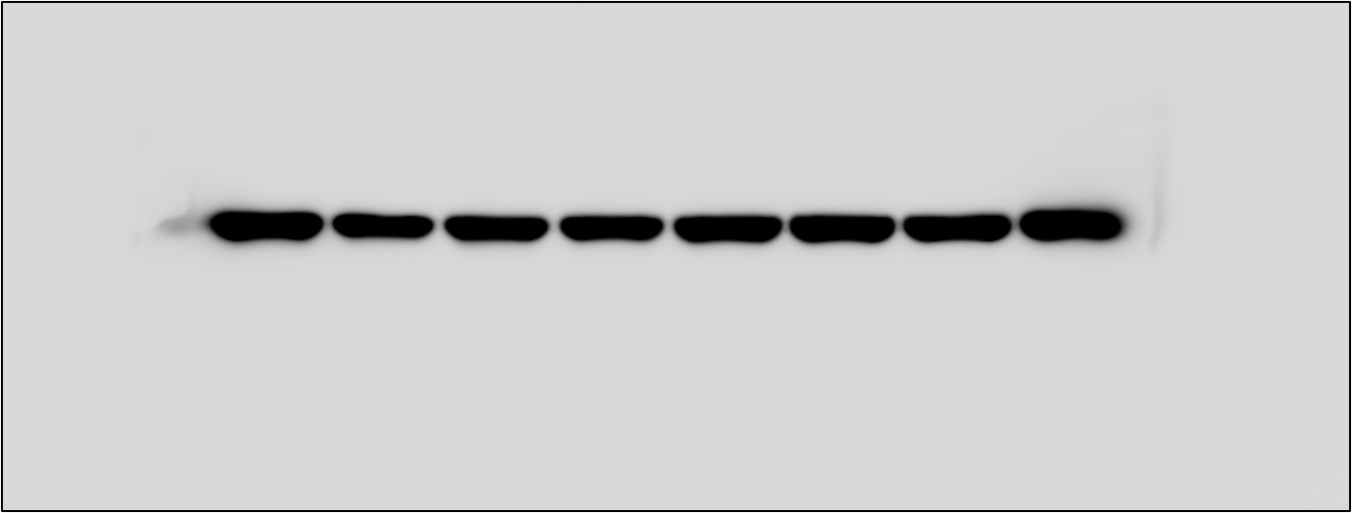

Supplement: Figure 1—source data 2. [file elife-98357-fig1-data2.zip › Figure 1-source data 2/1 F EPC-Actin.tif]

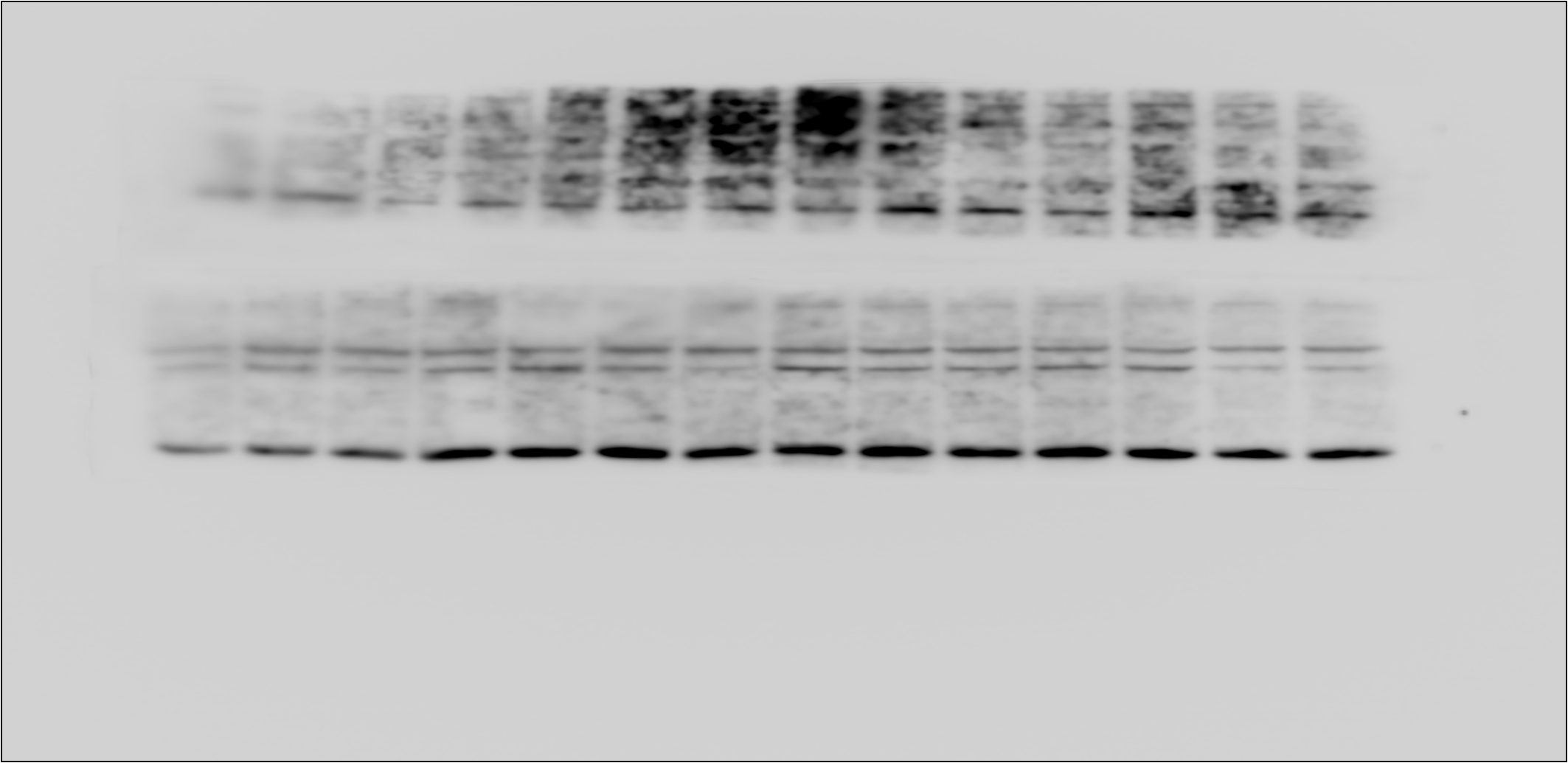

Supplement: Figure 1—source data 2. [file elife-98357-fig1-data2.zip › Figure 1-source data 2/1 F EPC-CDK2.tif]

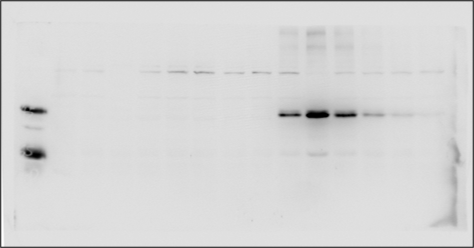

Supplement: Figure 1—source data 2. [file elife-98357-fig1-data2.zip › Figure 1-source data 2/1 F EPC-G.tif]

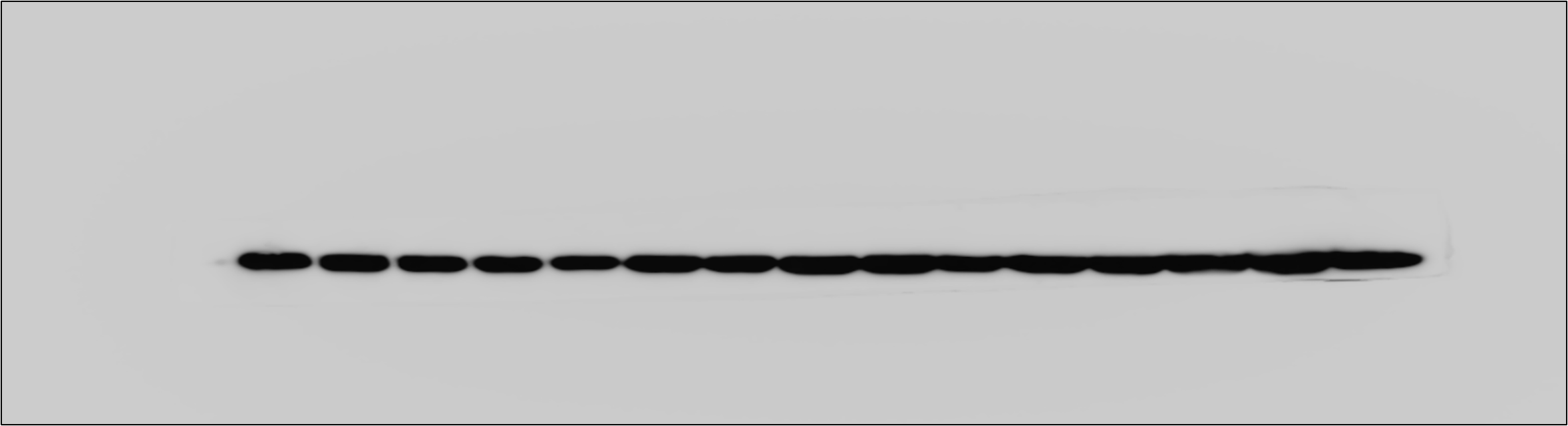

Supplement: Figure 1—source data 2. [file elife-98357-fig1-data2.zip › Figure 1-source data 2/1 F ZF4-Actin.tif]

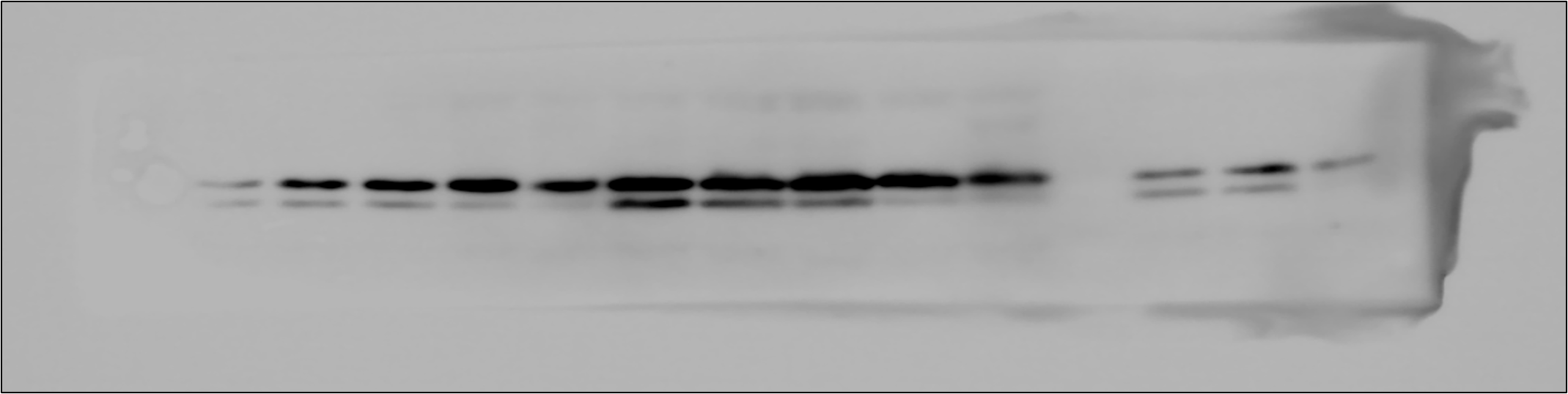

Supplement: Figure 1—source data 2. [file elife-98357-fig1-data2.zip › Figure 1-source data 2/1 F ZF4-CDK2.tif]

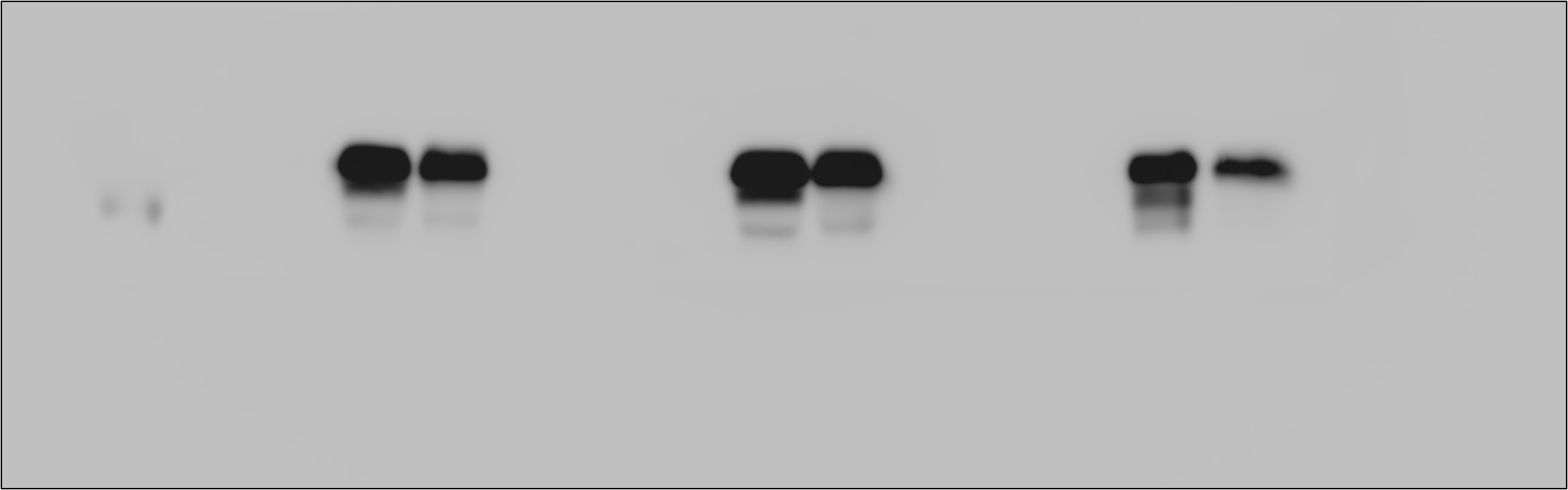

Supplement: Figure 1—source data 2. [file elife-98357-fig1-data2.zip › Figure 1-source data 2/1 F ZF4-G.tif]

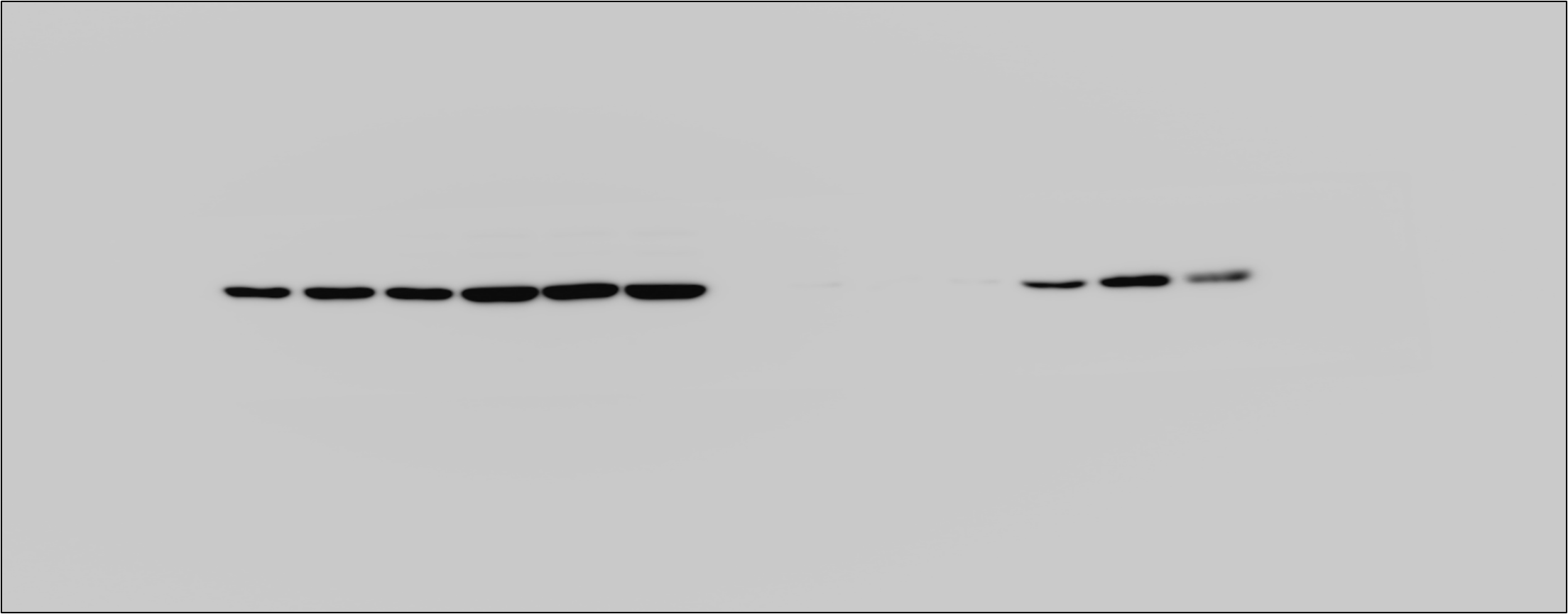

Supplement: Figure 2—source data 2. [file elife-98357-fig2-data2.zip › Figure 2-source data 2/2D Kidney-Actin.tif]

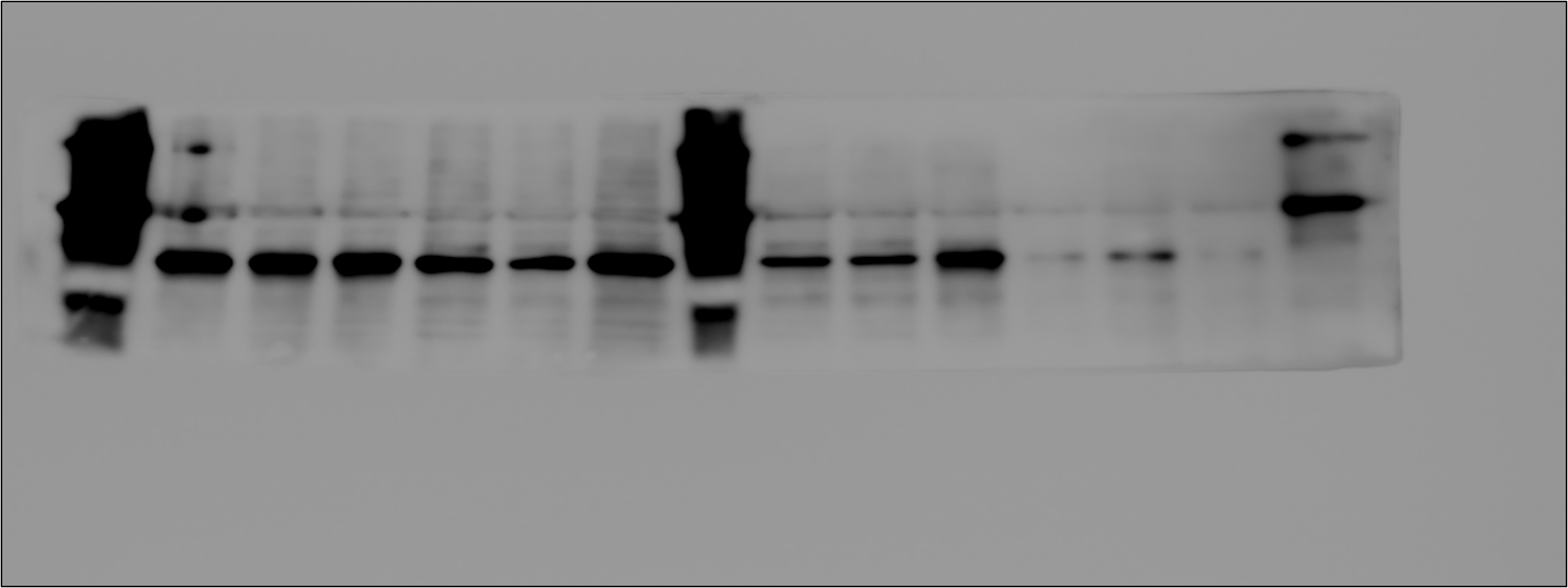

Supplement: Figure 2—source data 2. [file elife-98357-fig2-data2.zip › Figure 2-source data 2/2D Kidney-G.tif]

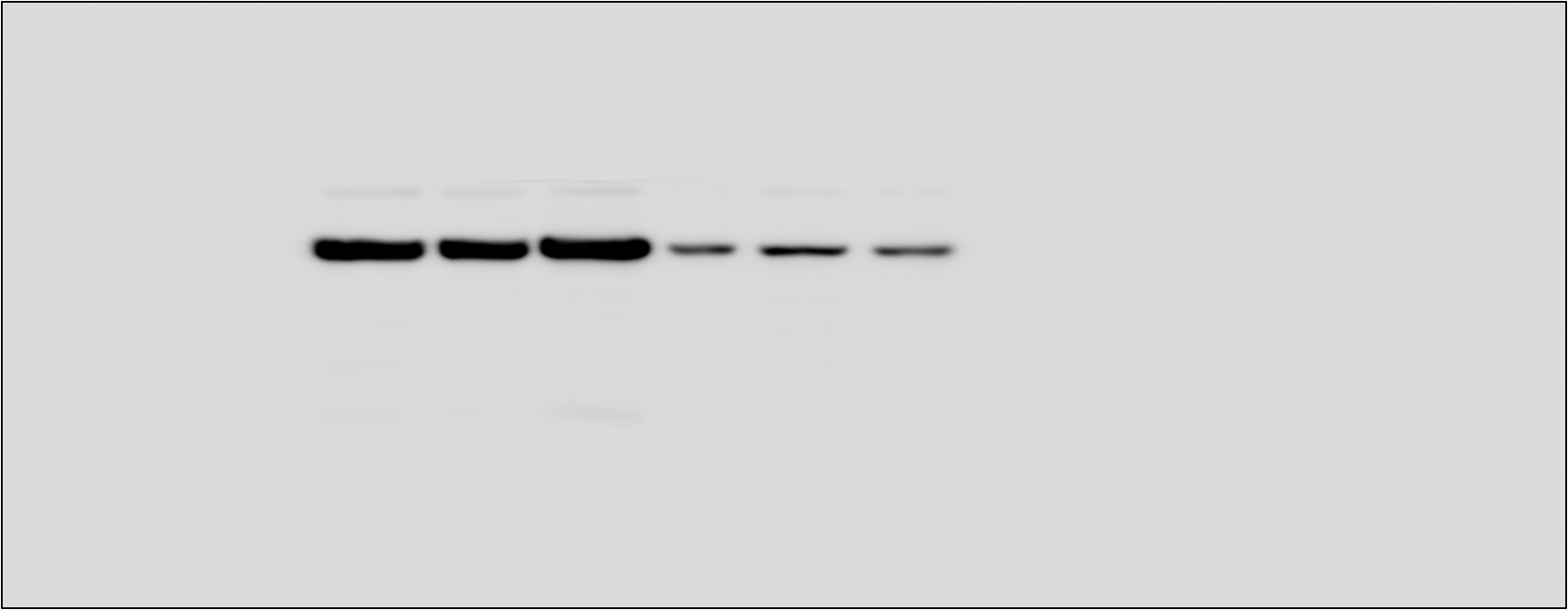

Supplement: Figure 2—source data 2. [file elife-98357-fig2-data2.zip › Figure 2-source data 2/2D Kidney-N.tif]

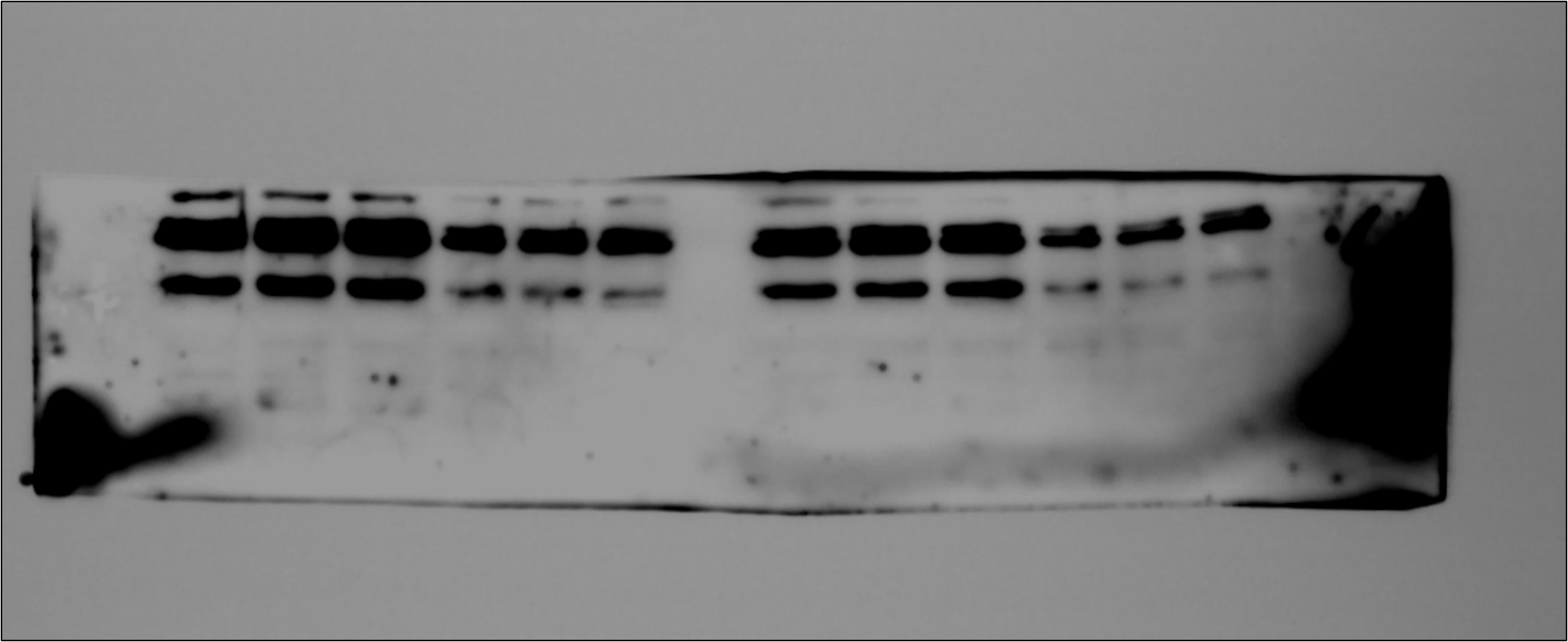

Supplement: Figure 2—source data 2. [file elife-98357-fig2-data2.zip › Figure 2-source data 2/2D Kidney-P.tif]

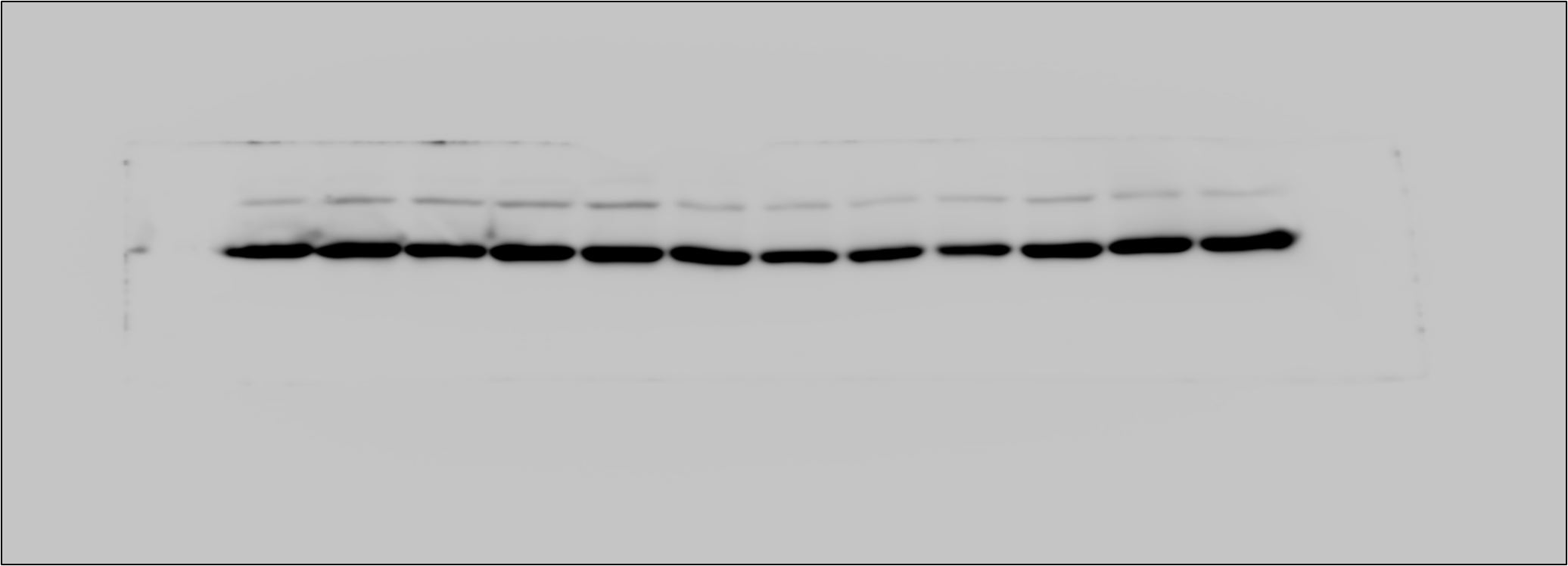

Supplement: Figure 2—source data 2. [file elife-98357-fig2-data2.zip › Figure 2-source data 2/2D Liver-Actin.tif]

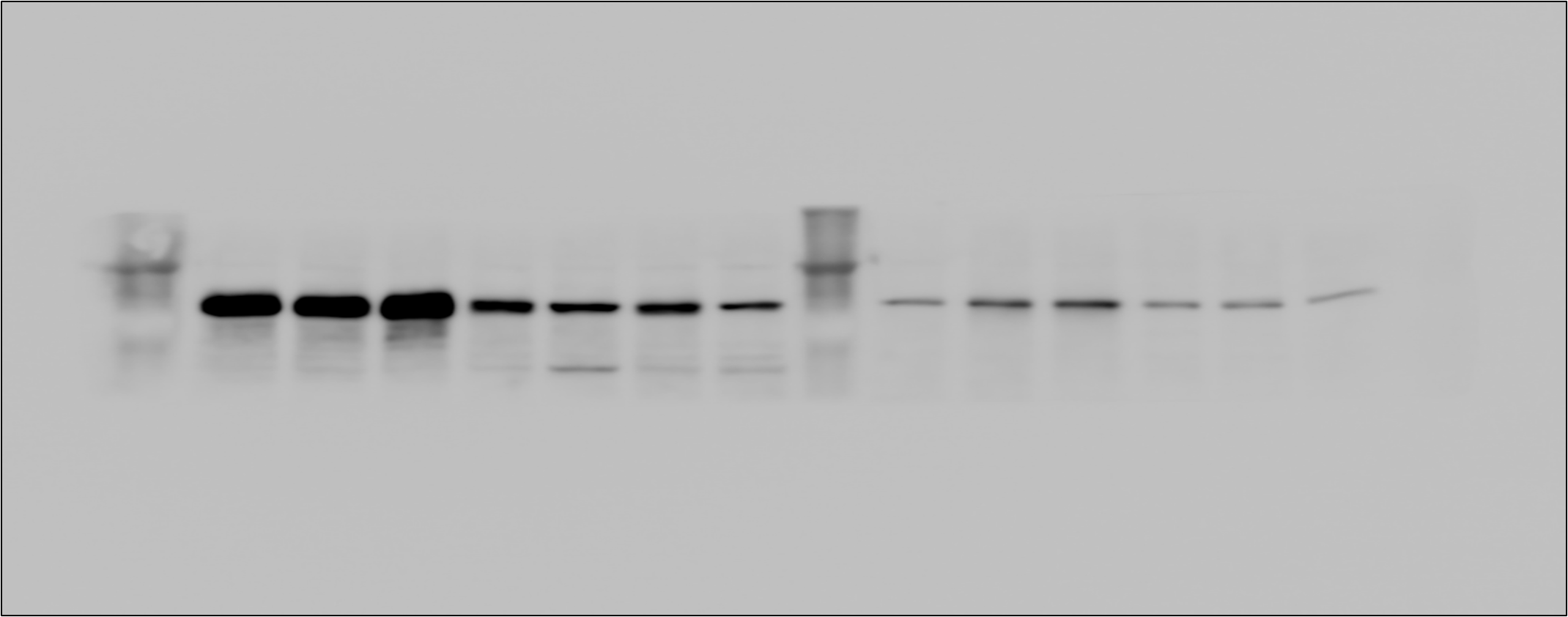

Supplement: Figure 2—source data 2. [file elife-98357-fig2-data2.zip › Figure 2-source data 2/2D Liver-G.tif]

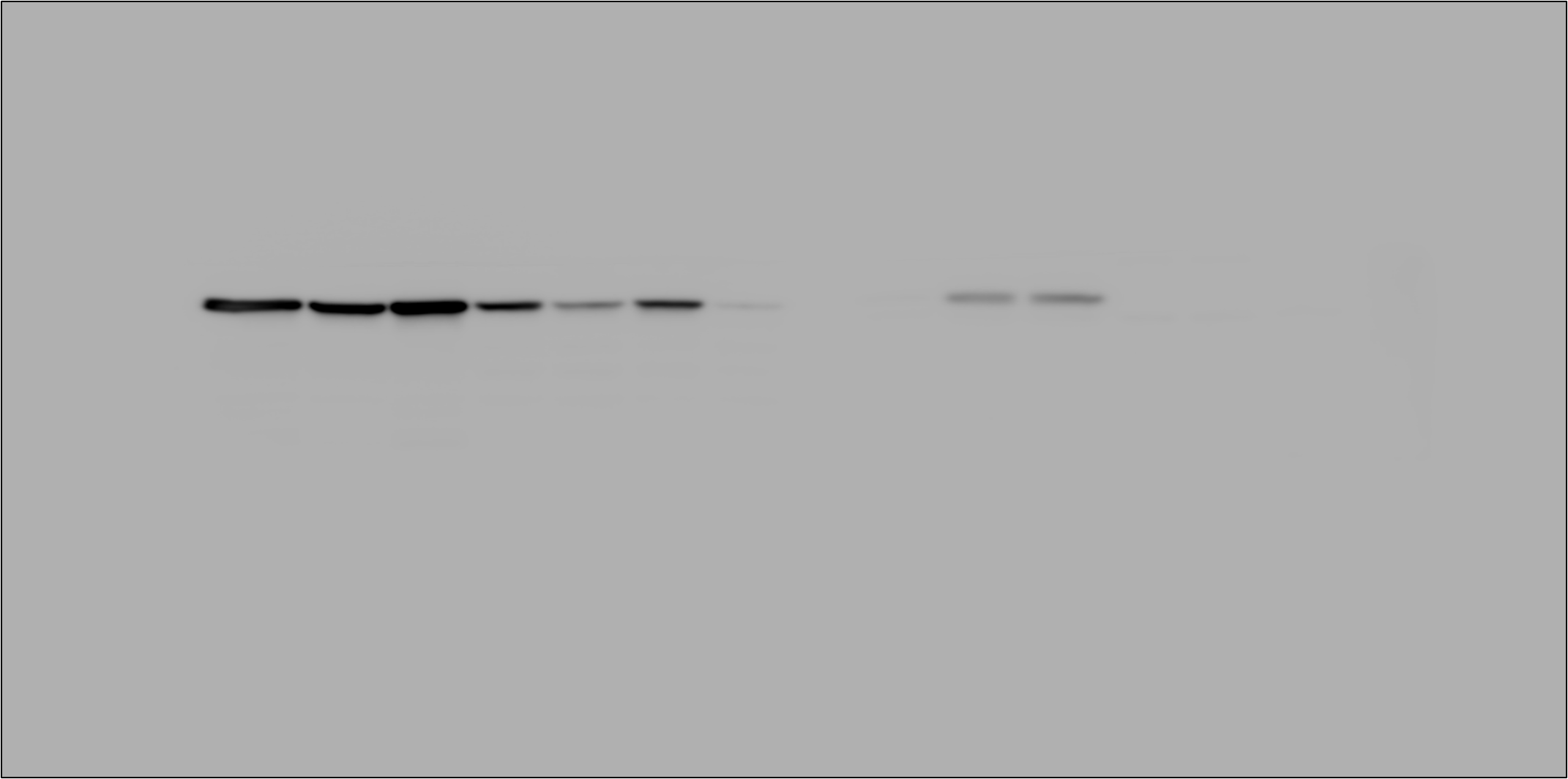

Supplement: Figure 2—source data 2. [file elife-98357-fig2-data2.zip › Figure 2-source data 2/2D Liver-N.tif]

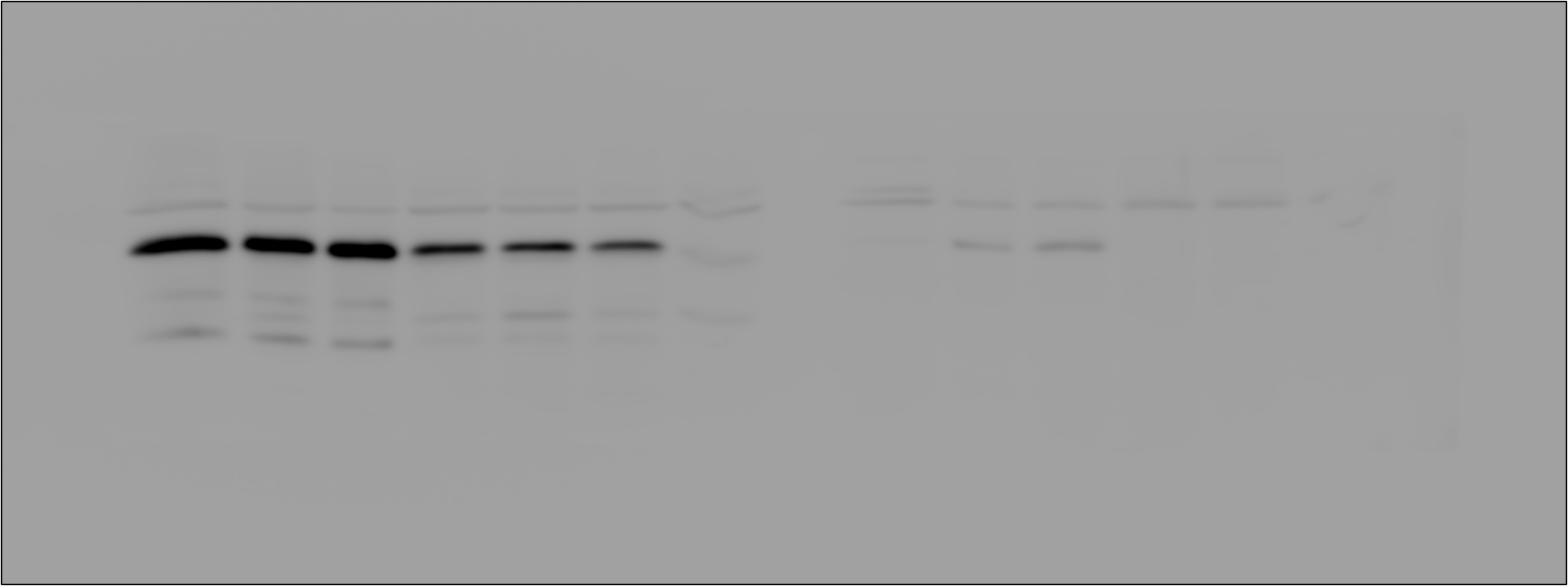

Supplement: Figure 2—source data 2. [file elife-98357-fig2-data2.zip › Figure 2-source data 2/2D Liver-P.tif]

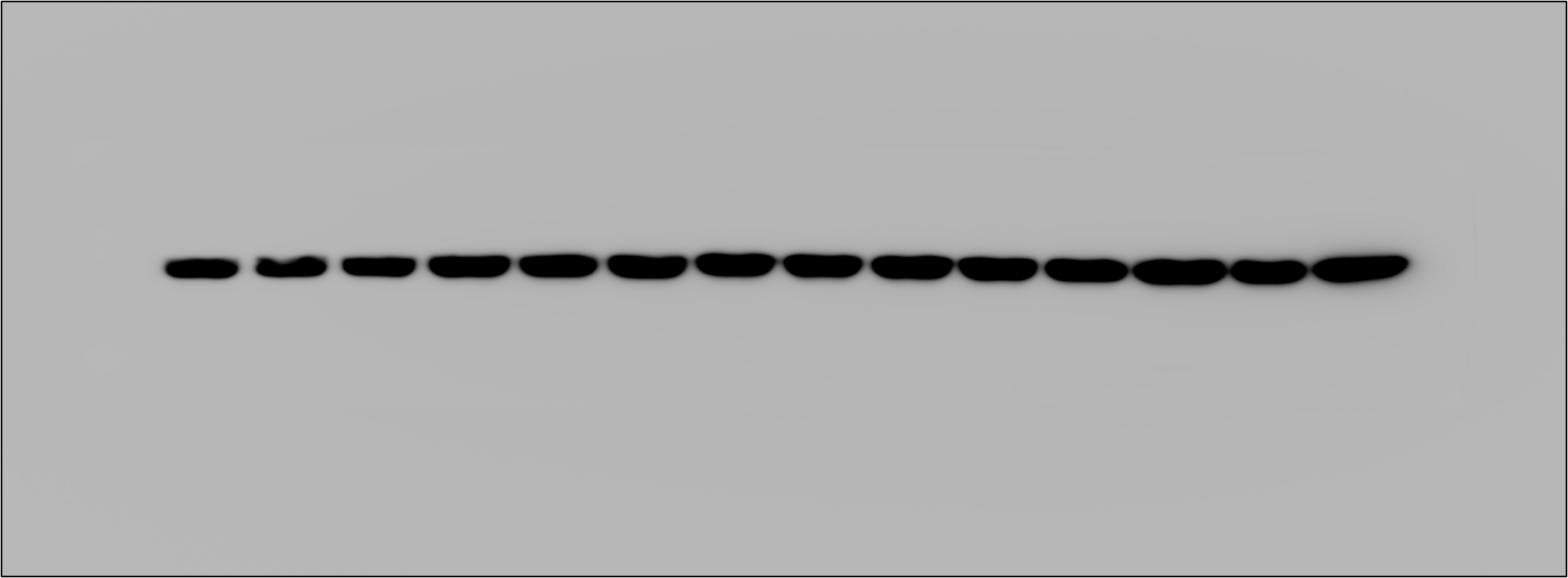

Supplement: Figure 2—source data 2. [file elife-98357-fig2-data2.zip › Figure 2-source data 2/2D Spleen-Actin.tif]

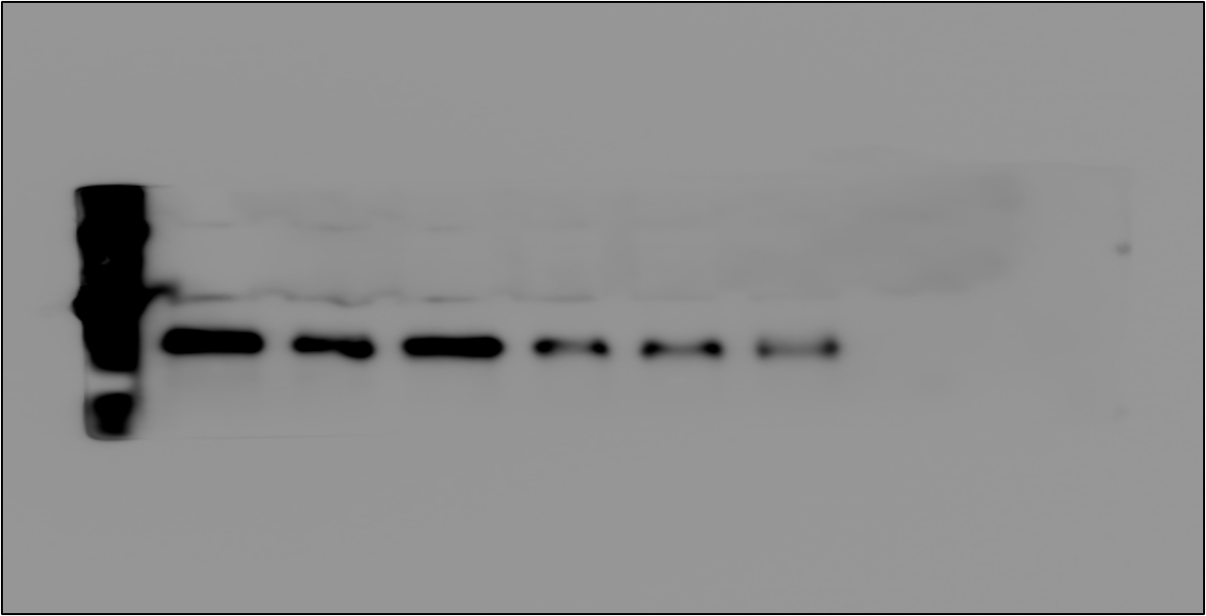

Supplement: Figure 2—source data 2. [file elife-98357-fig2-data2.zip › Figure 2-source data 2/2D Spleen-G.tif]

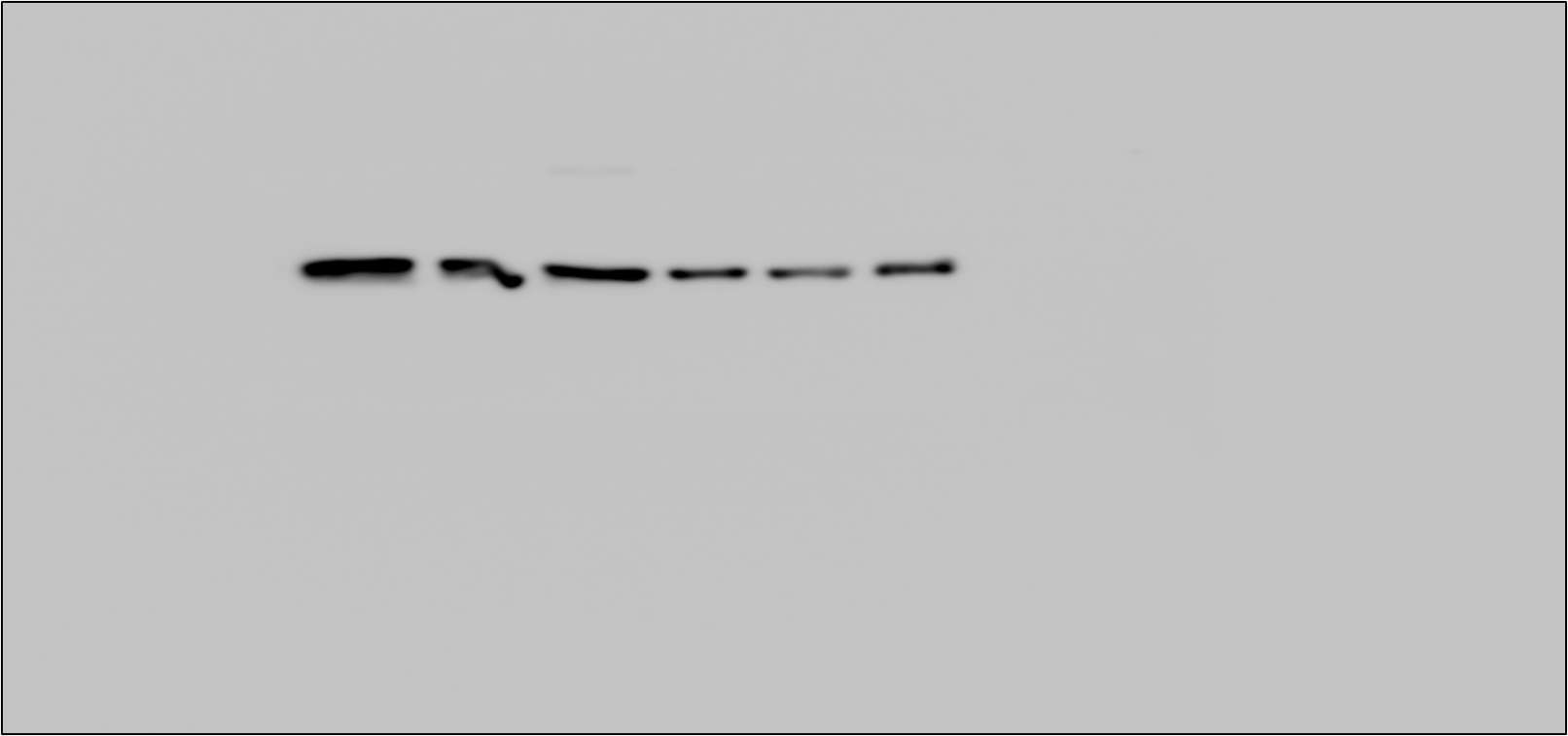

Supplement: Figure 2—source data 2. [file elife-98357-fig2-data2.zip › Figure 2-source data 2/2D Spleen-N.tif]

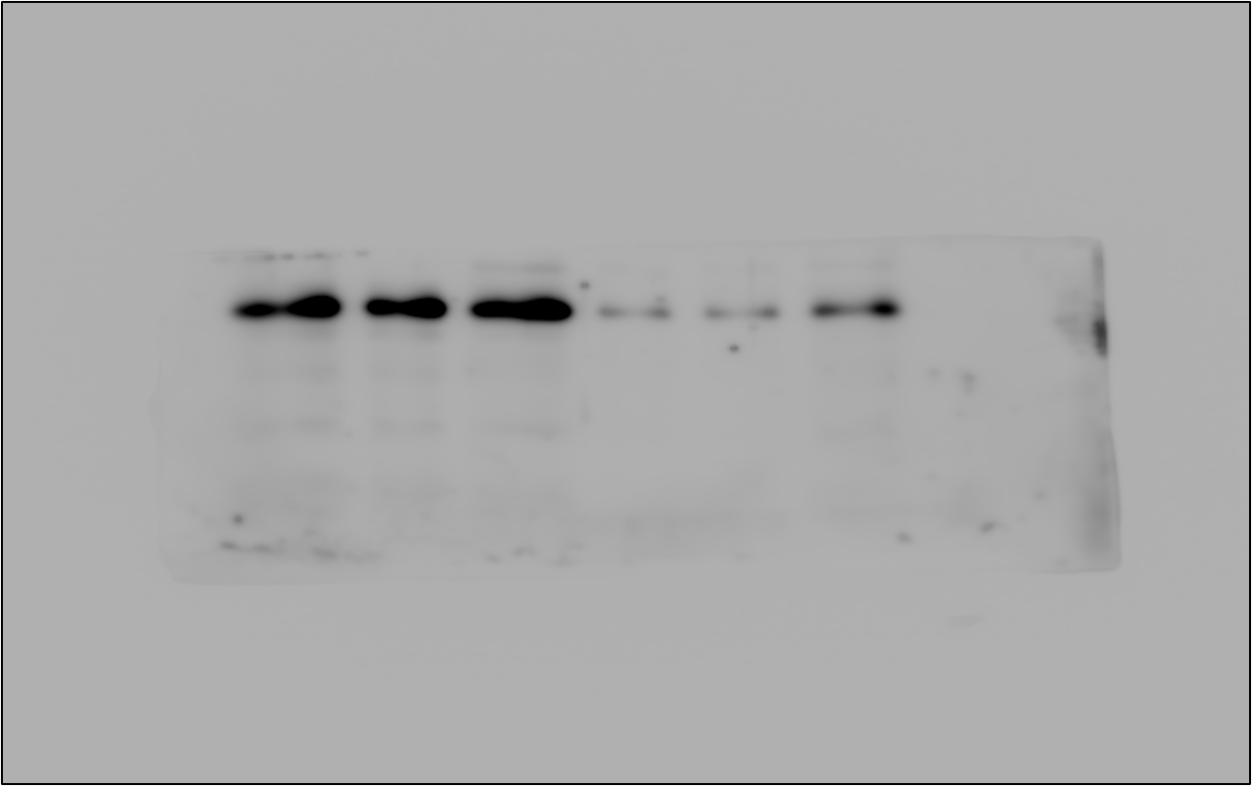

Supplement: Figure 2—source data 2. [file elife-98357-fig2-data2.zip › Figure 2-source data 2/2D Spleen-P.tif]

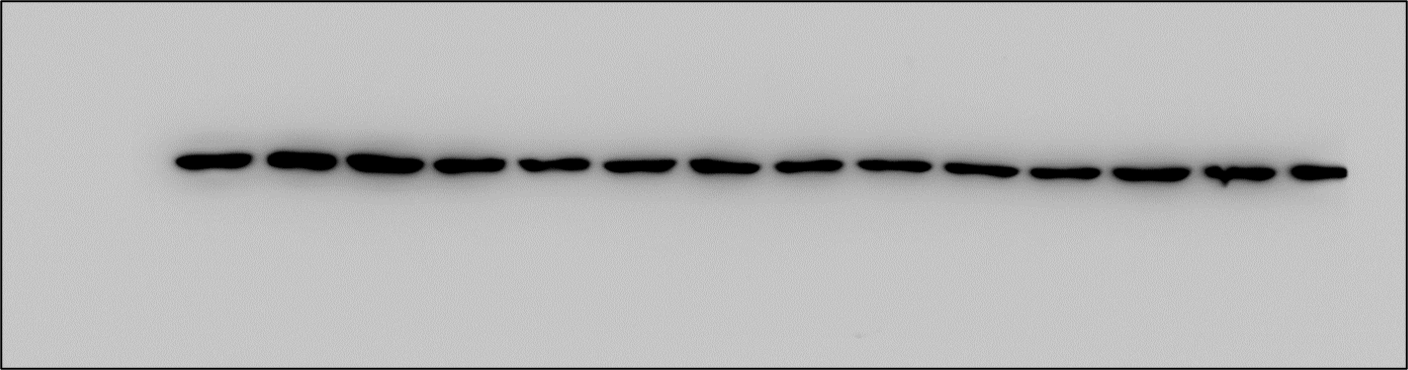

Supplement: Figure 3—source data 2. [file elife-98357-fig3-data2.zip › Figure 3-source data 2/3 B-Actin.tif]

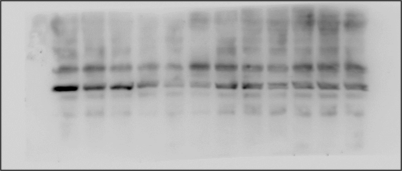

Supplement: Figure 3—source data 2. [file elife-98357-fig3-data2.zip › Figure 3-source data 2/3 B-CDK2.tif]

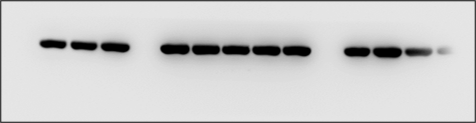

Supplement: Figure 3—source data 2. [file elife-98357-fig3-data2.zip › Figure 3-source data 2/3 H CDK2-Actin.tif]

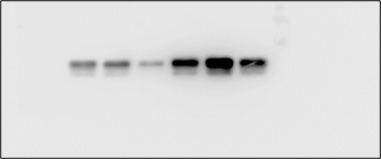

Supplement: Figure 3—source data 2. [file elife-98357-fig3-data2.zip › Figure 3-source data 2/3 H CDK2-G.tif]

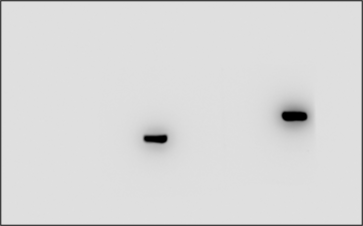

Supplement: Figure 3—source data 2. [file elife-98357-fig3-data2.zip › Figure 3-source data 2/3 H CDK2-HA.tif]

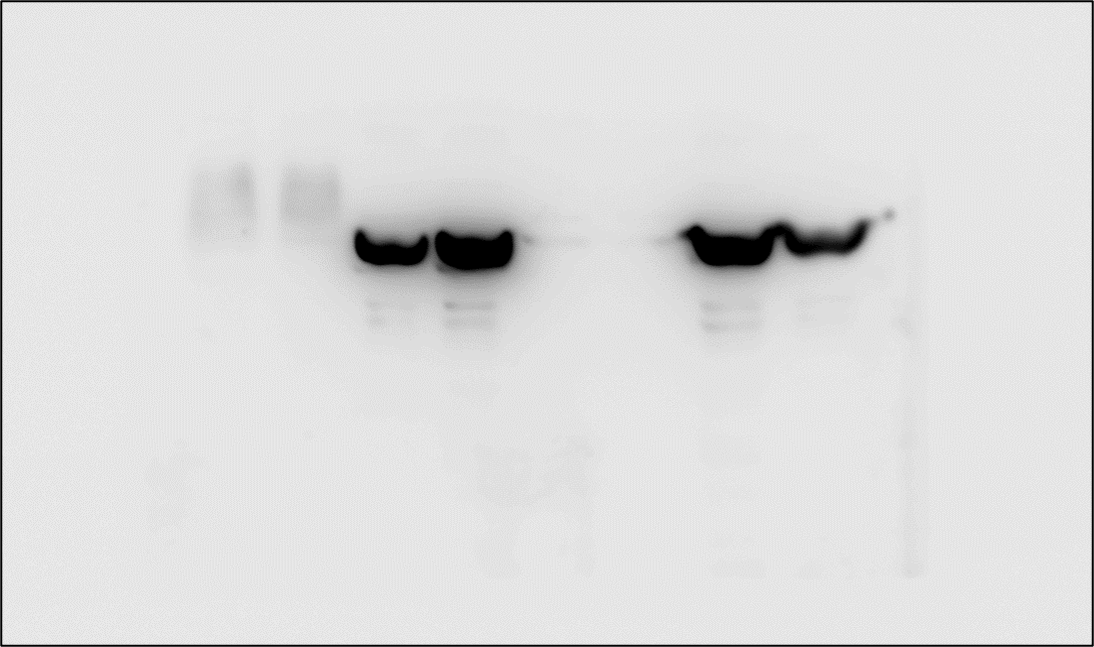

Supplement: Figure 3—source data 2. [file elife-98357-fig3-data2.zip › Figure 3-source data 2/3 H CDK2-N.tif]

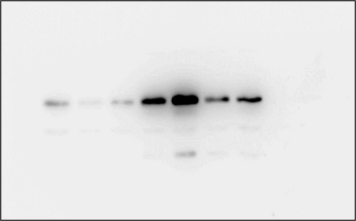

Supplement: Figure 3—source data 2. [file elife-98357-fig3-data2.zip › Figure 3-source data 2/3 H CDK2-P.tif]

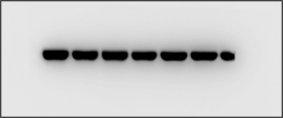

Supplement: Figure 3—source data 2. [file elife-98357-fig3-data2.zip › Figure 3-source data 2/3 H shcdk2-actin.tif]

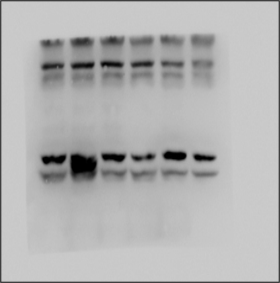

Supplement: Figure 3—source data 2. [file elife-98357-fig3-data2.zip › Figure 3-source data 2/3 H shcdk2-CDK2.tif]

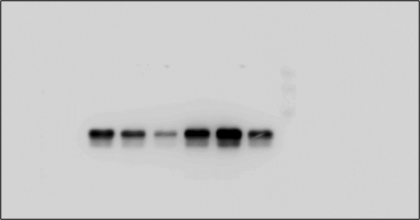

Supplement: Figure 3—source data 2. [file elife-98357-fig3-data2.zip › Figure 3-source data 2/3 H shcdk2-G.tif]

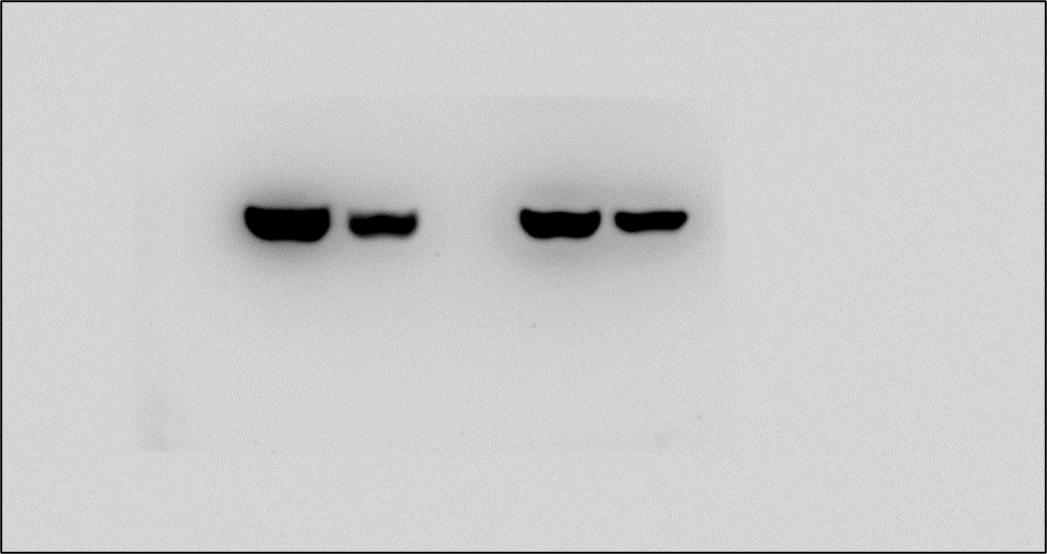

Supplement: Figure 3—source data 2. [file elife-98357-fig3-data2.zip › Figure 3-source data 2/3 H shcdk2-N.tif]

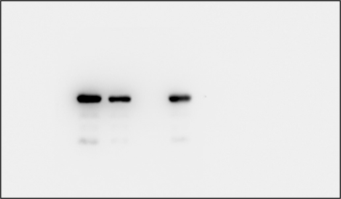

Supplement: Figure 3—source data 2. [file elife-98357-fig3-data2.zip › Figure 3-source data 2/3 H shcdk2-P.tif]

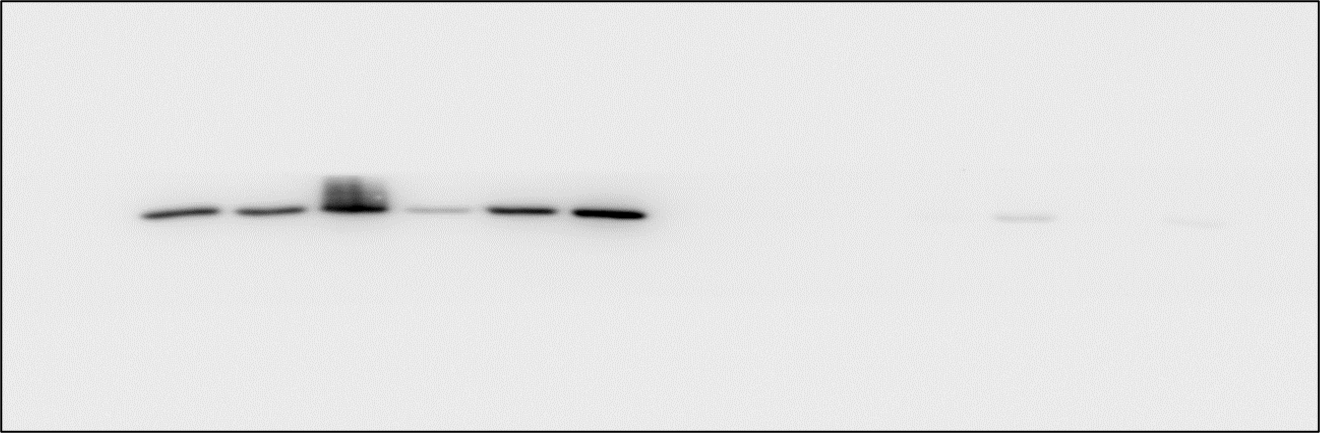

Supplement: Figure 4—source data 2. [file elife-98357-fig4-data2.zip › Figure 4-source data 2/4 F Input-HA.tif]

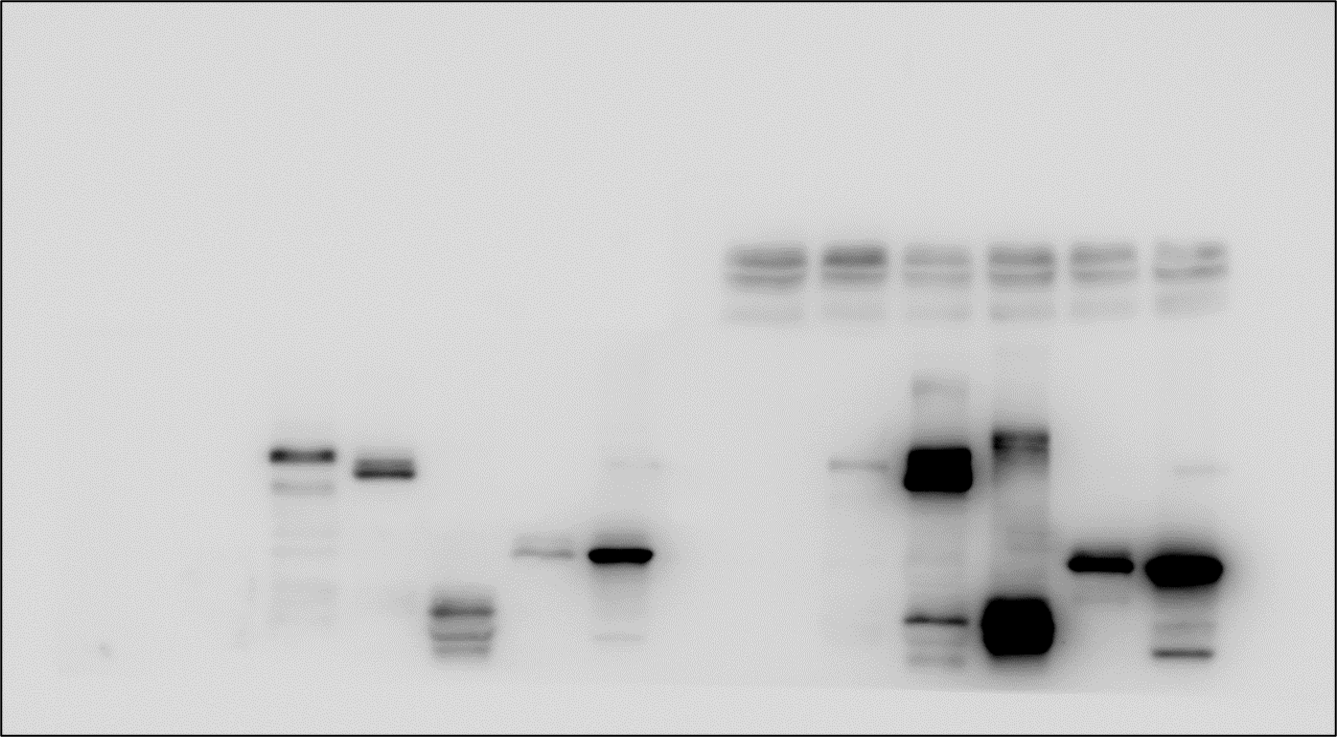

Supplement: Figure 4—source data 2. [file elife-98357-fig4-data2.zip › Figure 4-source data 2/4 F Input-Myc.tif]

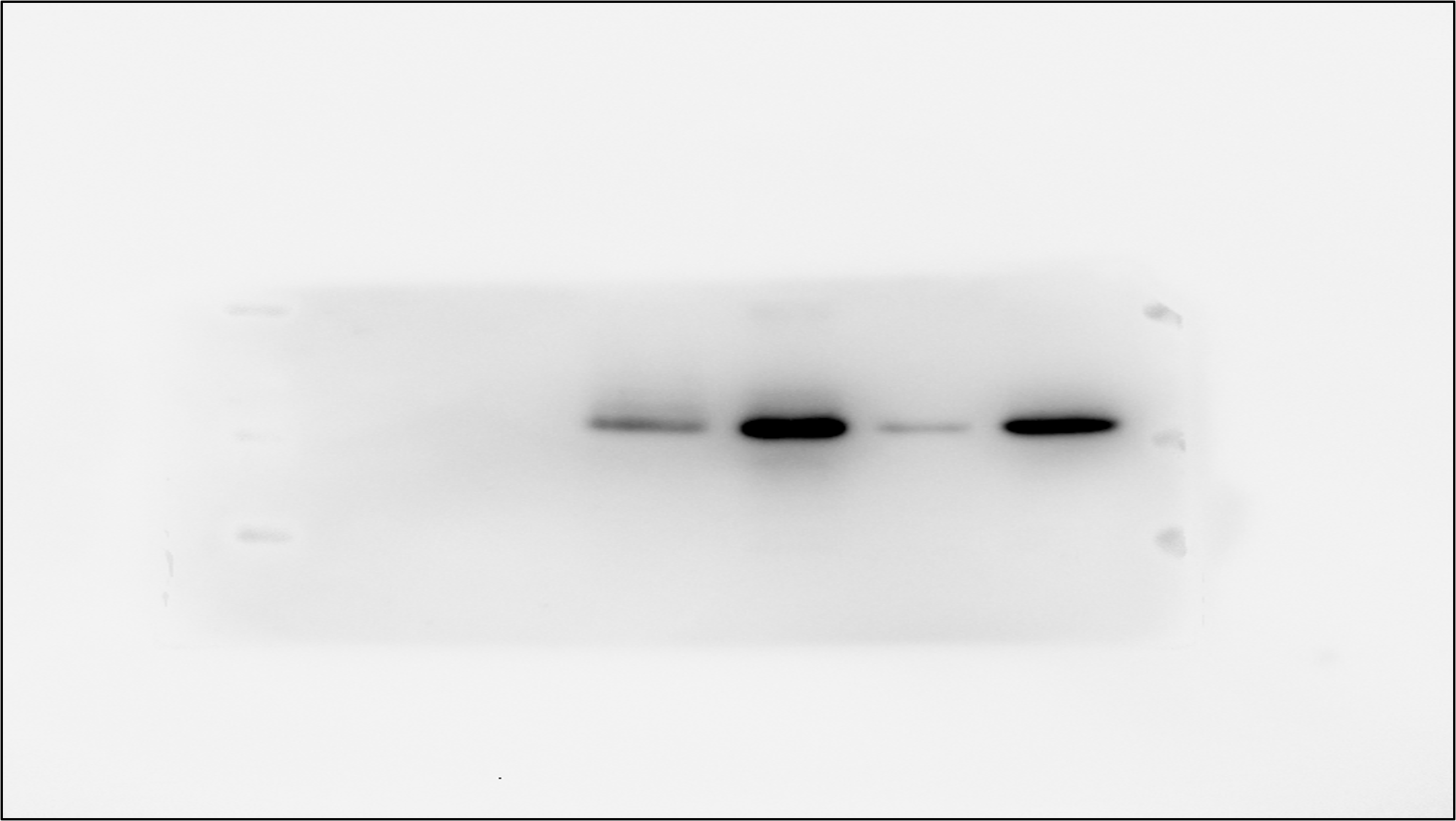

Supplement: Figure 4—source data 2. [file elife-98357-fig4-data2.zip › Figure 4-source data 2/4 F IP-HA.tif]

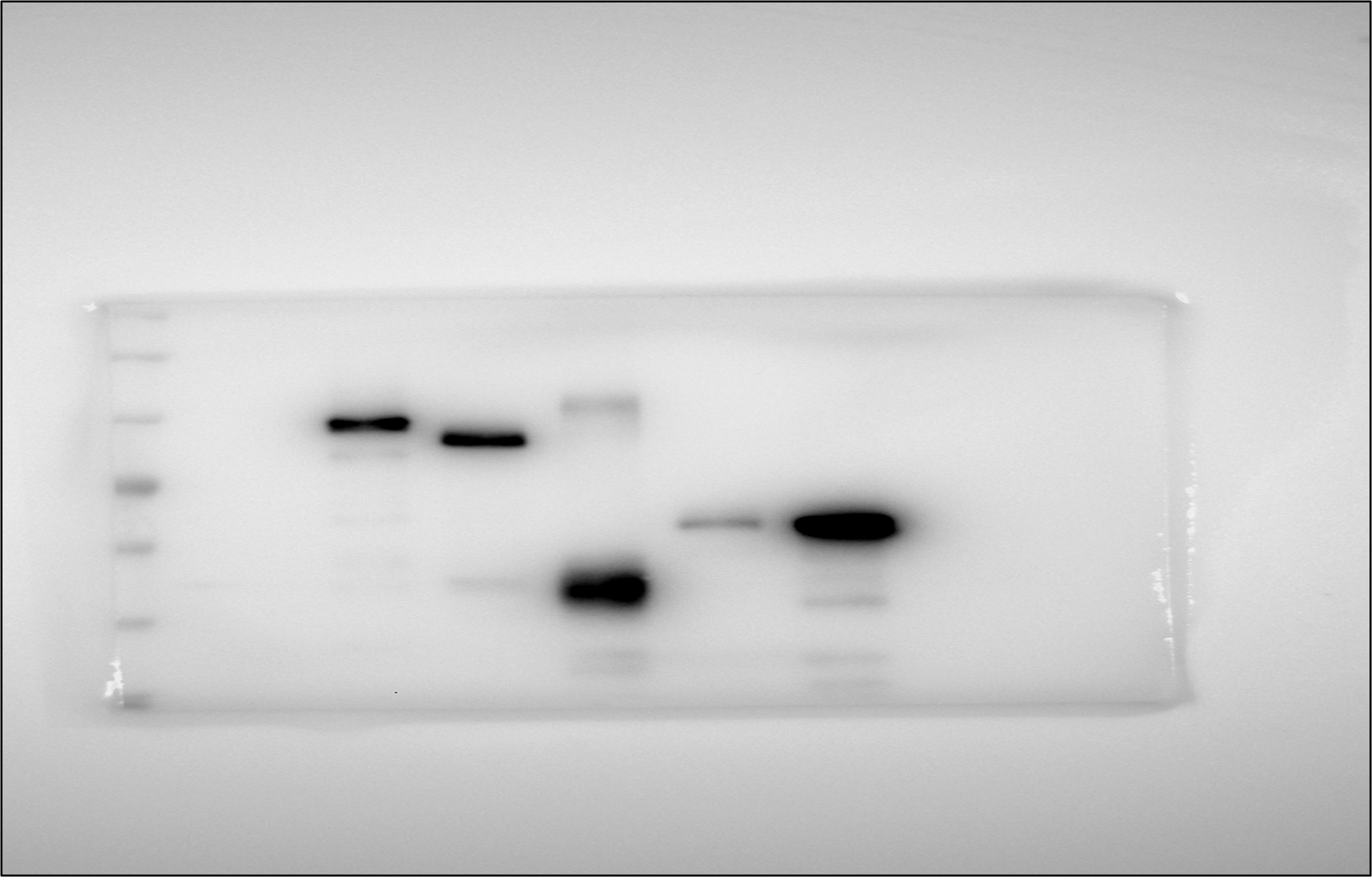

Supplement: Figure 4—source data 2. [file elife-98357-fig4-data2.zip › Figure 4-source data 2/4 F IP-Myc.tif]

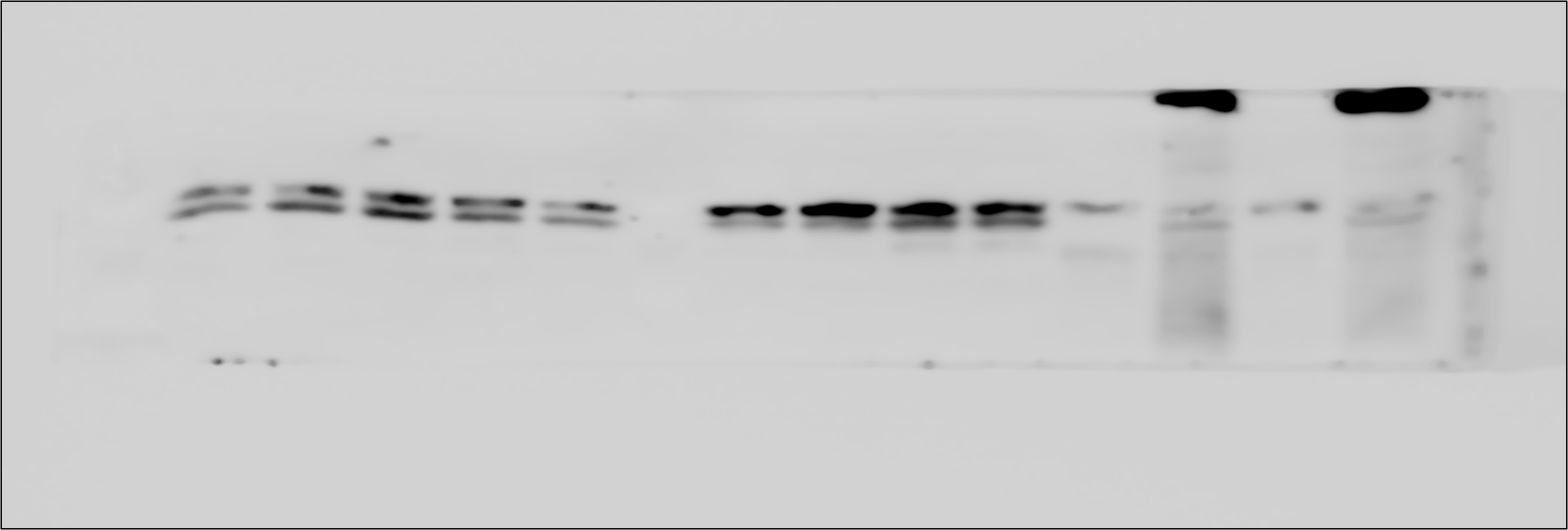

Supplement: Figure 4—source data 2. [file elife-98357-fig4-data2.zip › Figure 4-source data 2/4 G IP CDK2-CDK2-Input.tif]

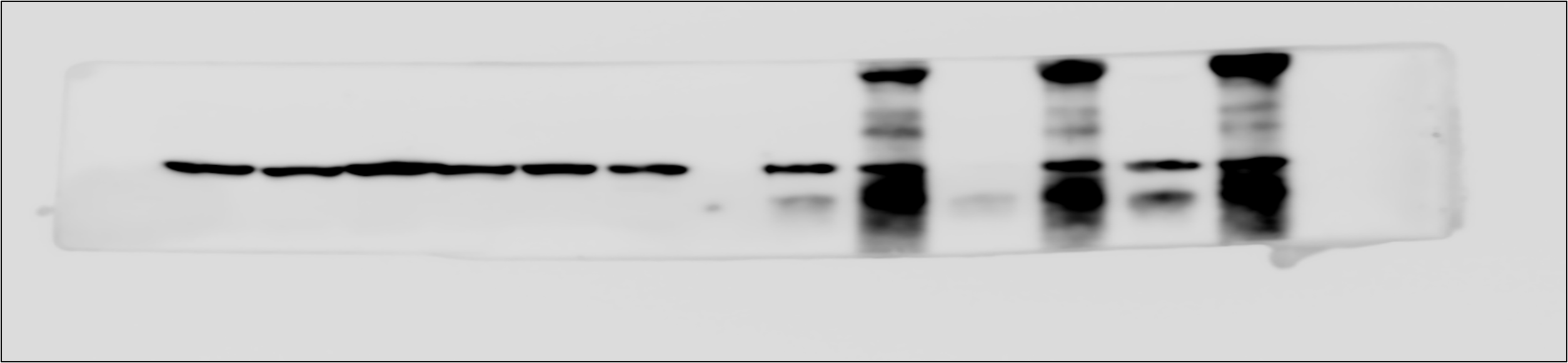

Supplement: Figure 4—source data 2. [file elife-98357-fig4-data2.zip › Figure 4-source data 2/4 G IP CDK2-CDK2.tif]

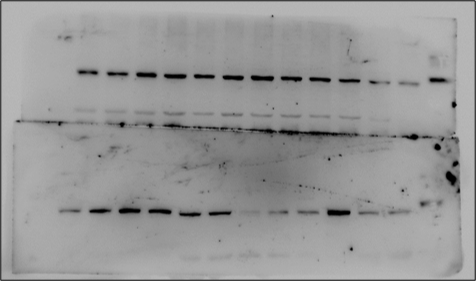

Supplement: Figure 4—source data 2. [file elife-98357-fig4-data2.zip › Figure 4-source data 2/4 G IP CDK2-TBK1-Input.tif]

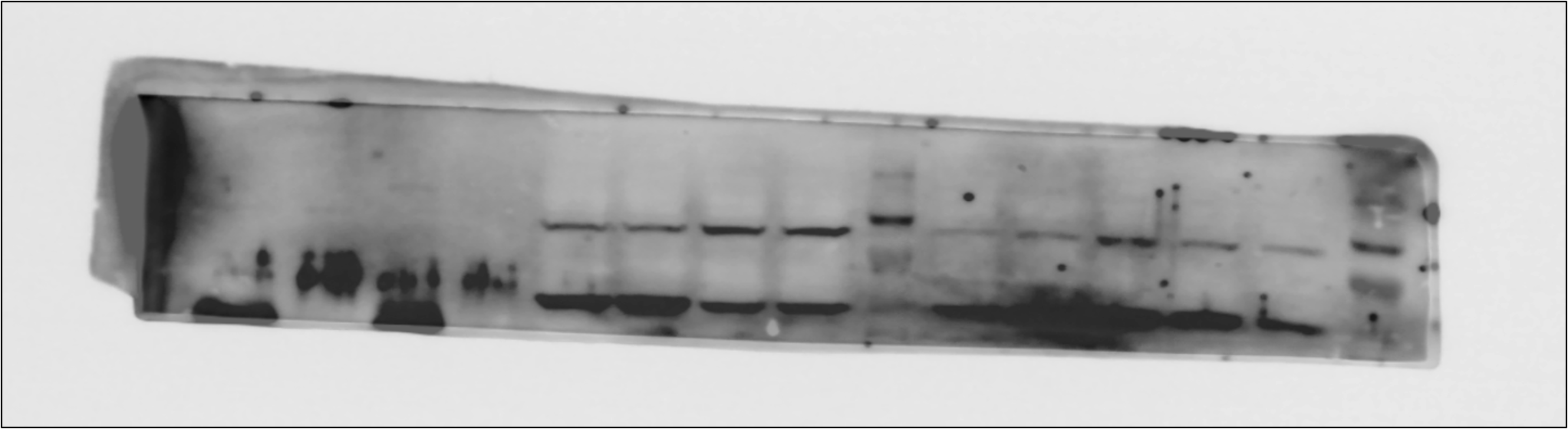

Supplement: Figure 4—source data 2. [file elife-98357-fig4-data2.zip › Figure 4-source data 2/4 G IP CDK2-TBK1.tif]

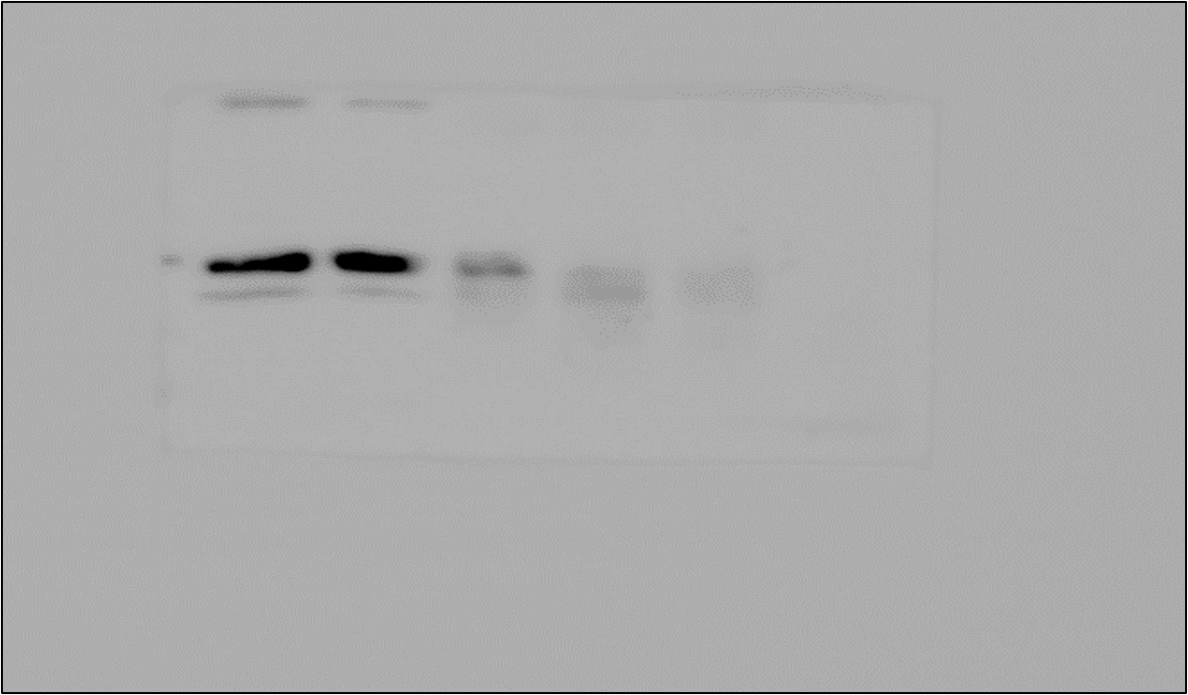

Supplement: Figure 4—source data 2. [file elife-98357-fig4-data2.zip › Figure 4-source data 2/4 G IP TBK-CDK2-Input.tif]

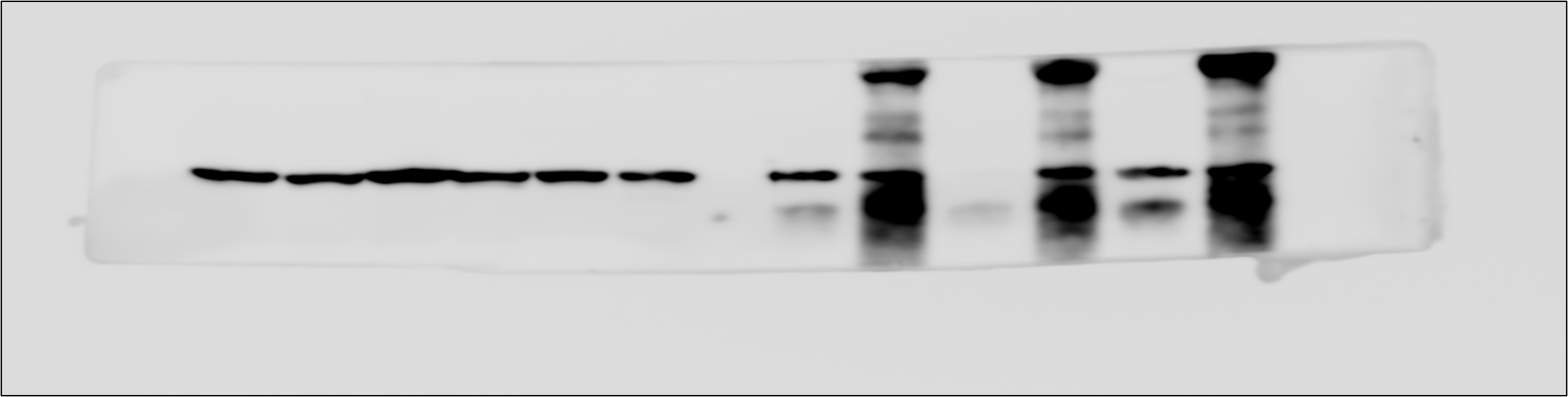

Supplement: Figure 4—source data 2. [file elife-98357-fig4-data2.zip › Figure 4-source data 2/4 G IP TBK-CDK2.tif]

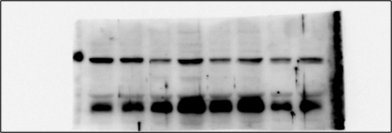

Supplement: Figure 4—source data 2. [file elife-98357-fig4-data2.zip › Figure 4-source data 2/4 G IP TBK-TBK1-Input.tif]

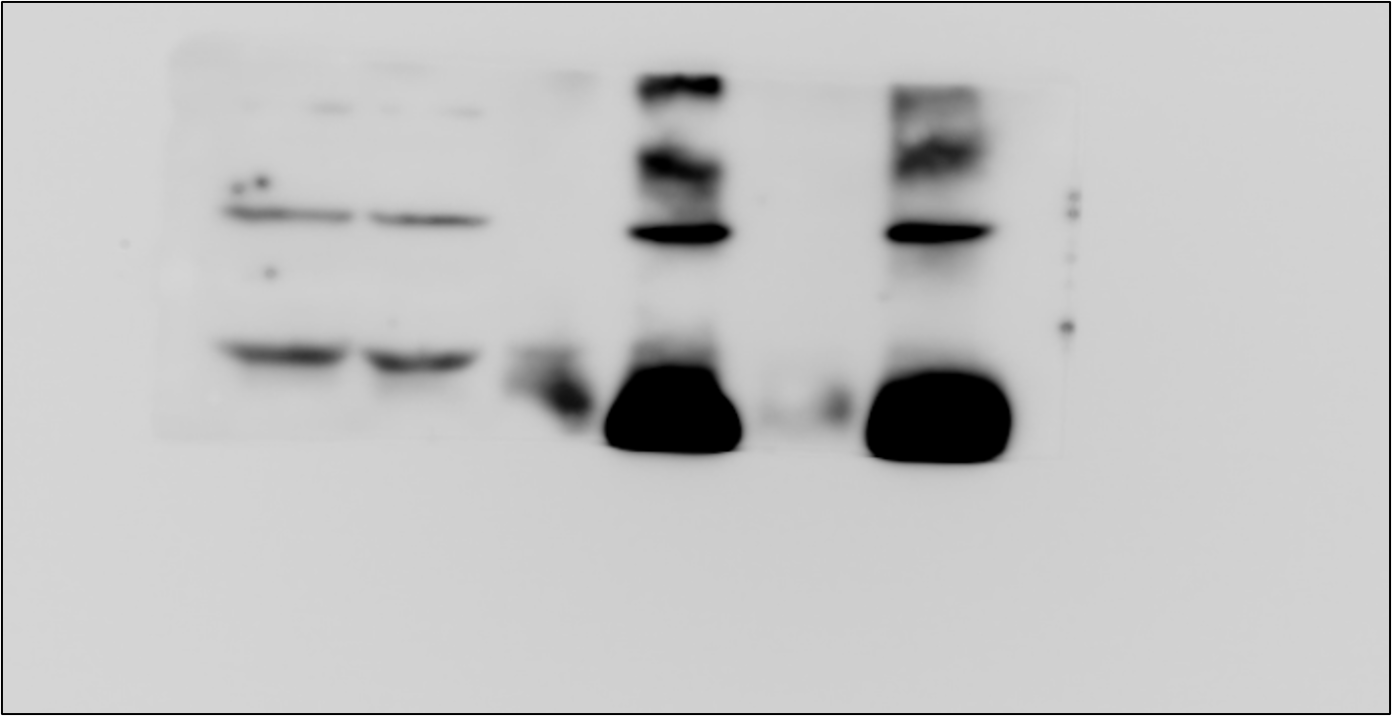

Supplement: Figure 4—source data 2. [file elife-98357-fig4-data2.zip › Figure 4-source data 2/4 G IP TBK-TBK1.tif]

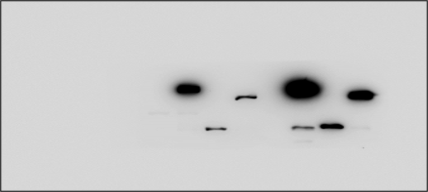

Supplement: Figure 4—source data 2. [file elife-98357-fig4-data2.zip › Figure 4-source data 2/4 I IP Flag-Flag-Input.tif]

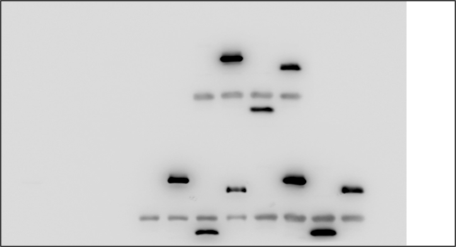

Supplement: Figure 4—source data 2. [file elife-98357-fig4-data2.zip › Figure 4-source data 2/4 I IP Flag-Flag.tif]

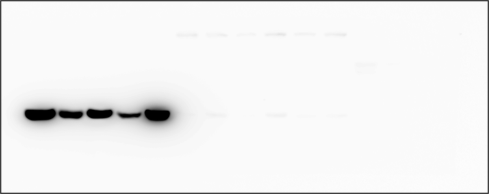

Supplement: Figure 4—source data 2. [file elife-98357-fig4-data2.zip › Figure 4-source data 2/4 I IP Flag-Myc-Input.tif]

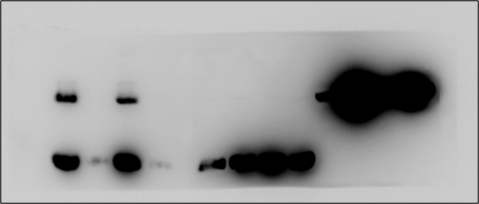

Supplement: Figure 4—source data 2. [file elife-98357-fig4-data2.zip › Figure 4-source data 2/4 I IP Flag-Myc.tif]

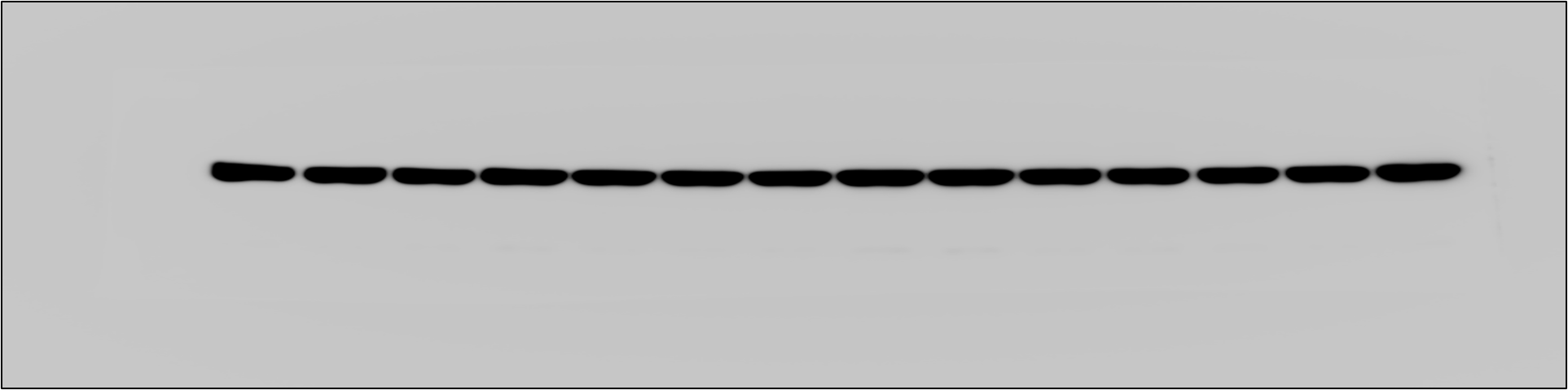

Supplement: Figure 4—source data 2. [file elife-98357-fig4-data2.zip › Figure 4-source data 2/4 J-Actin.tif]

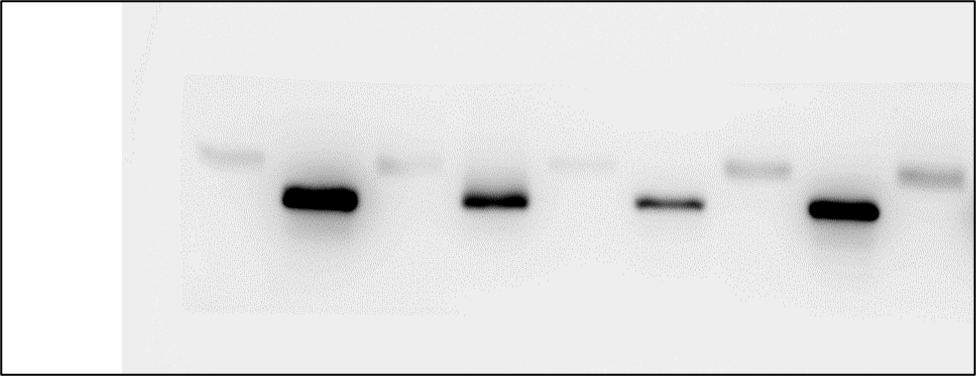

Supplement: Figure 4—source data 2. [file elife-98357-fig4-data2.zip › Figure 4-source data 2/4 J-HA.tif]

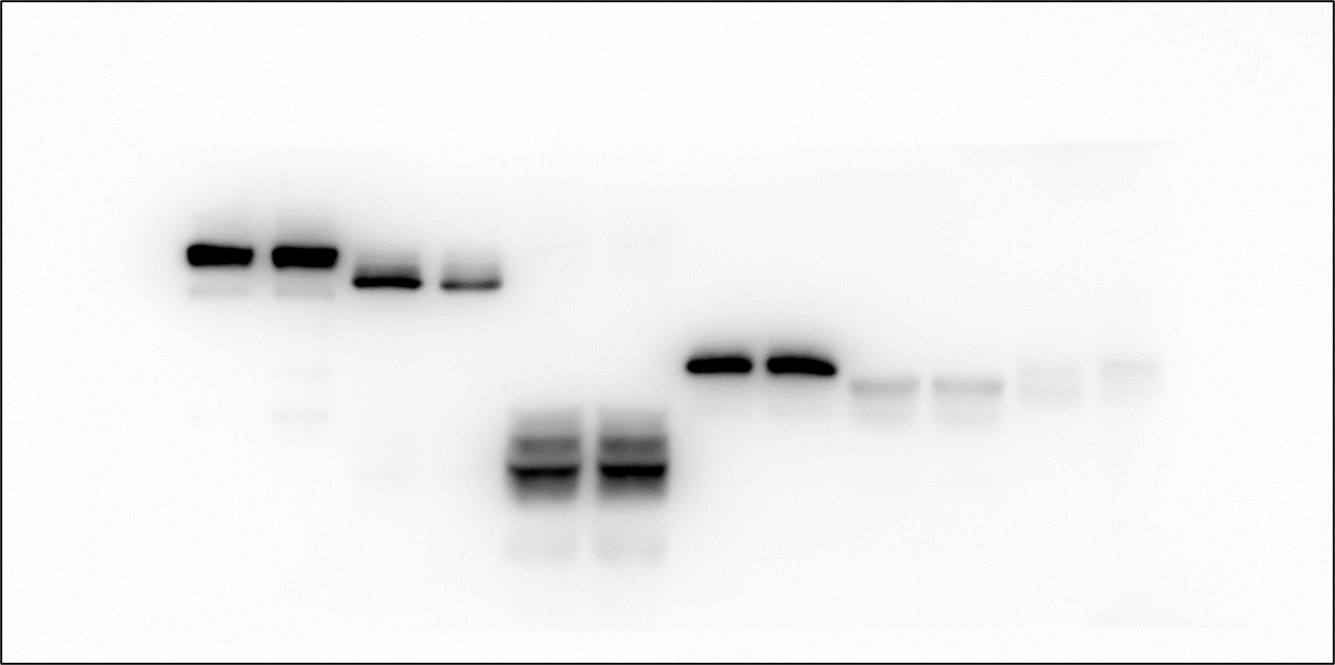

Supplement: Figure 4—source data 2. [file elife-98357-fig4-data2.zip › Figure 4-source data 2/4 J-Myc.tif]

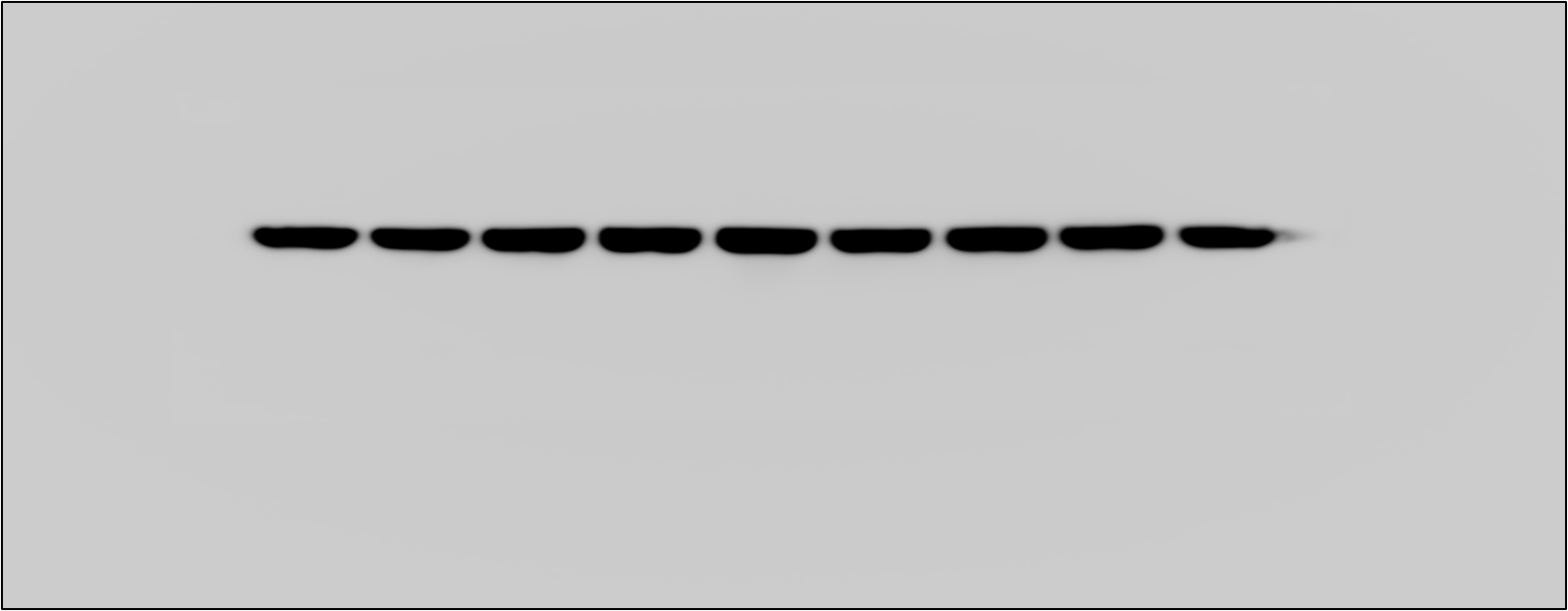

Supplement: Figure 4—source data 2. [file elife-98357-fig4-data2.zip › Figure 4-source data 2/4 K-Actin.tif]

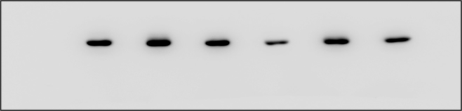

Supplement: Figure 4—source data 2. [file elife-98357-fig4-data2.zip › Figure 4-source data 2/4 K-HA.tif]

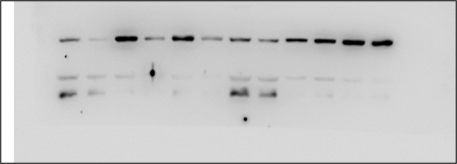

Supplement: Figure 4—source data 2. [file elife-98357-fig4-data2.zip › Figure 4-source data 2/4 K-TBK1.tif]

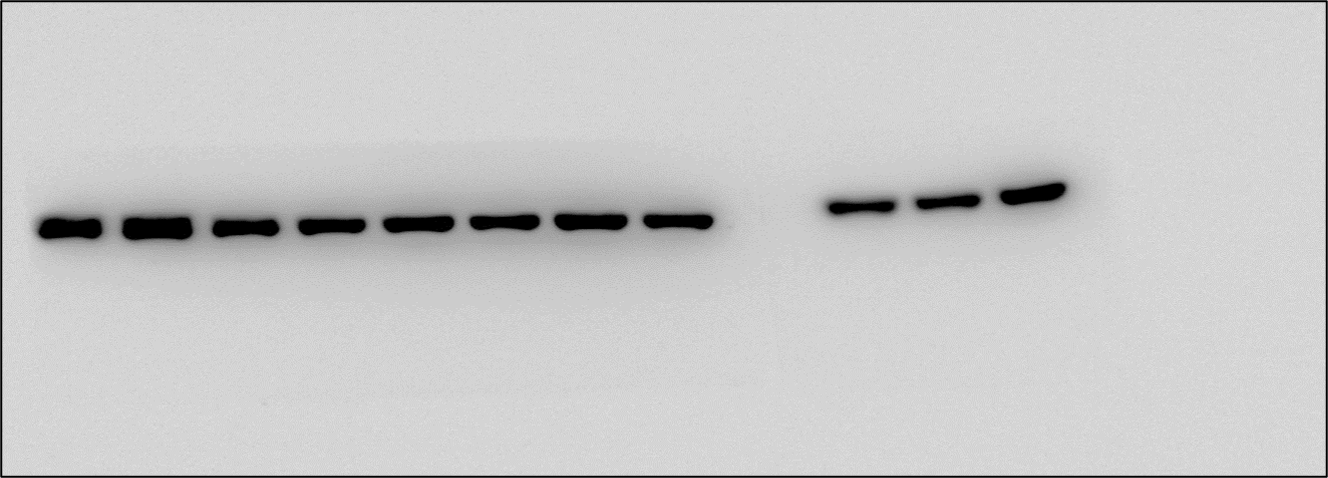

Supplement: Figure 4—source data 2. [file elife-98357-fig4-data2.zip › Figure 4-source data 2/4 L-Actin.tif]

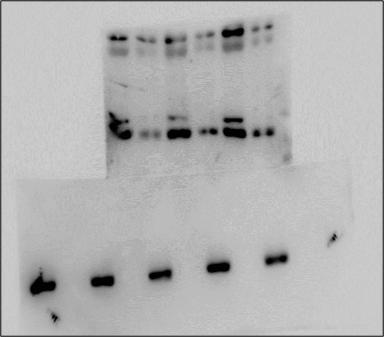

Supplement: Figure 4—source data 2. [file elife-98357-fig4-data2.zip › Figure 4-source data 2/4 L-CDK2.tif]

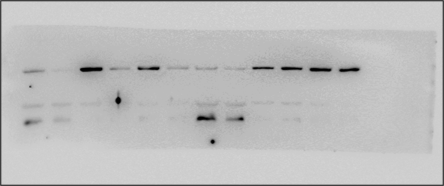

Supplement: Figure 4—source data 2. [file elife-98357-fig4-data2.zip › Figure 4-source data 2/4 L-TBK1.tif]

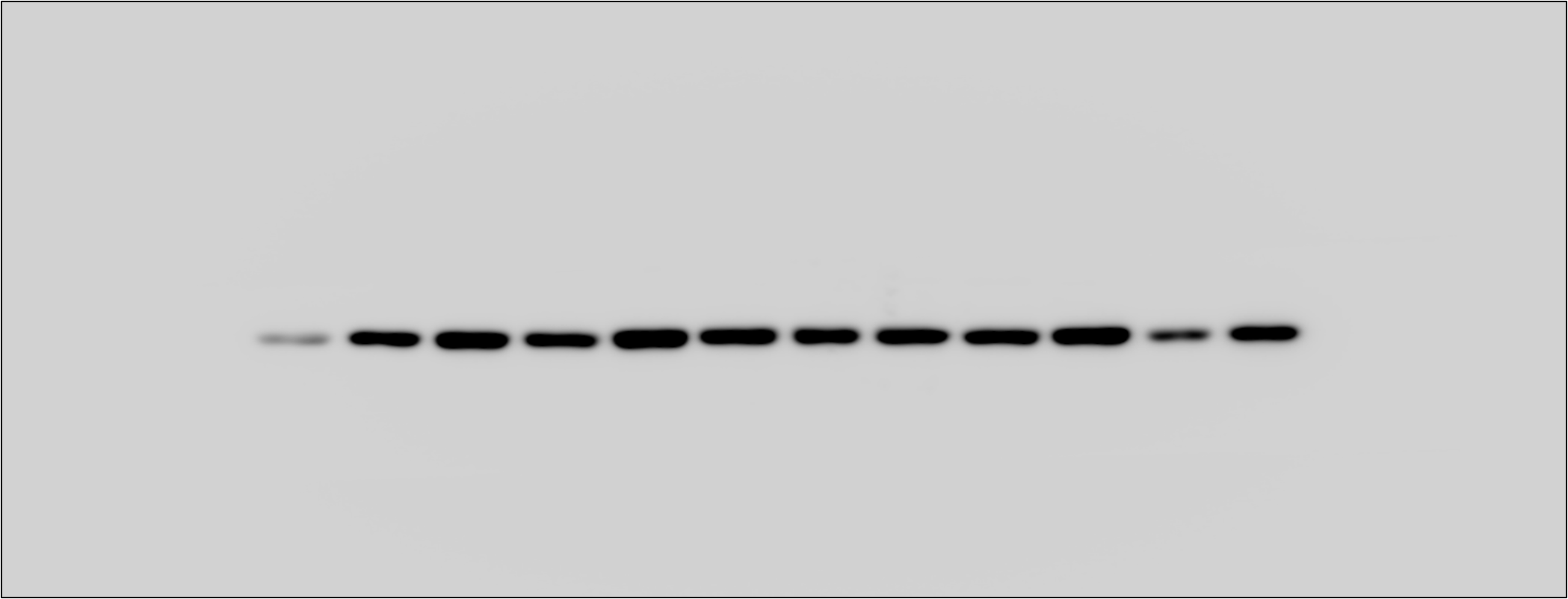

Supplement: Figure 4—source data 2. [file elife-98357-fig4-data2.zip › Figure 4-source data 2/4 M-Kidney-Actin.tif]

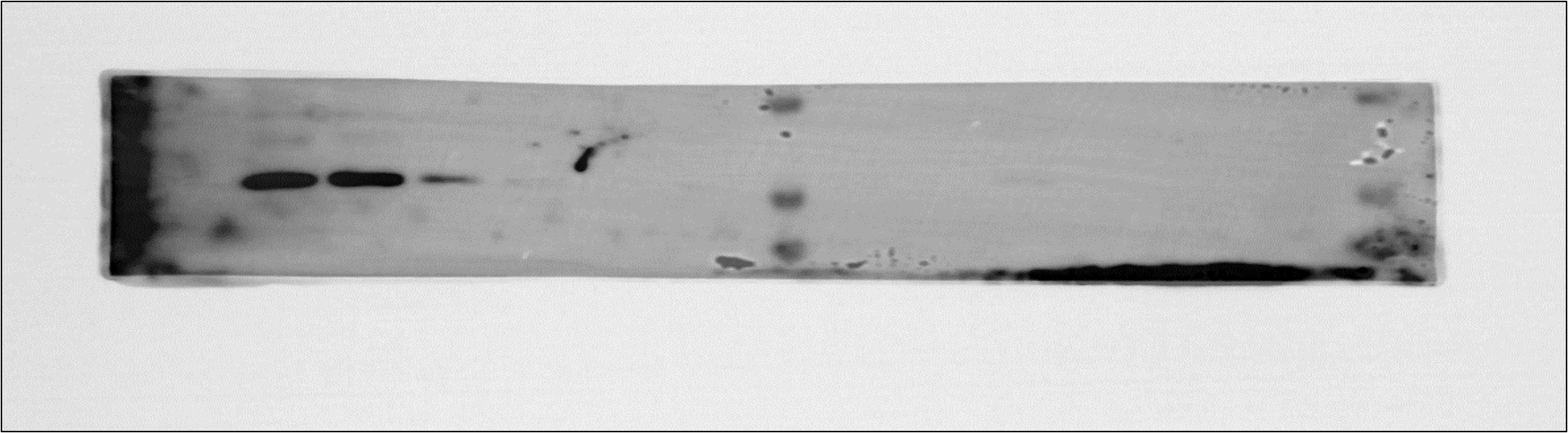

Supplement: Figure 4—source data 2. [file elife-98357-fig4-data2.zip › Figure 4-source data 2/4 M-Kidney-CDK2.tif]

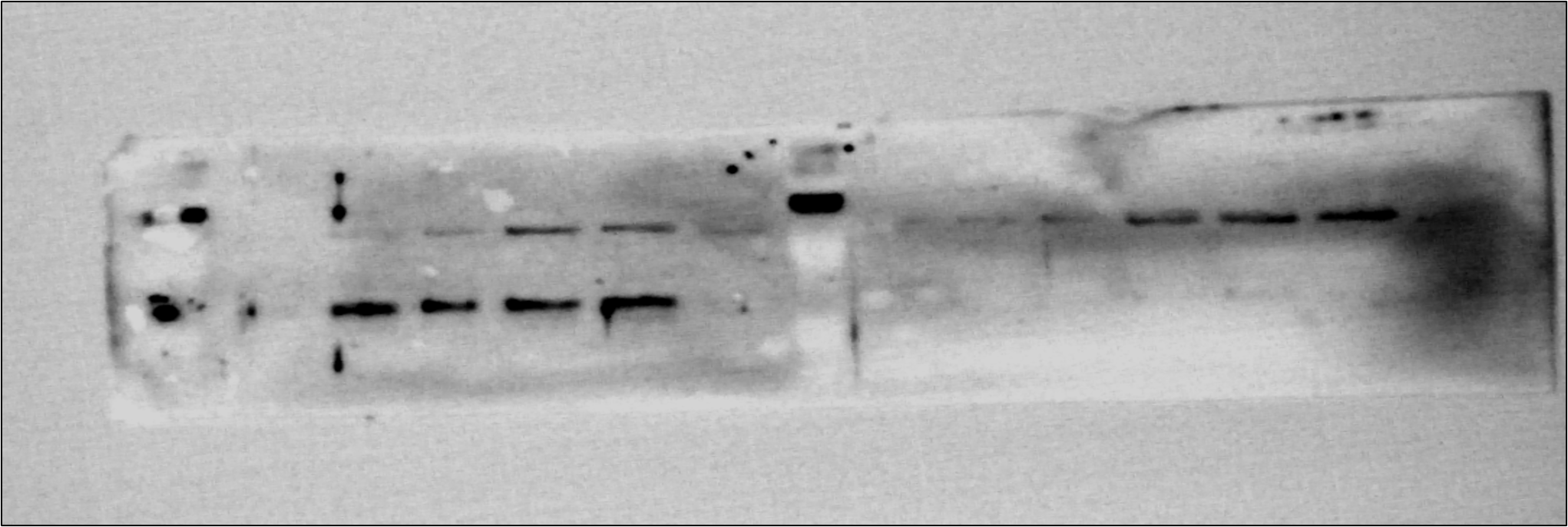

Supplement: Figure 4—source data 2. [file elife-98357-fig4-data2.zip › Figure 4-source data 2/4 M-Kidney-TBK1.tif]

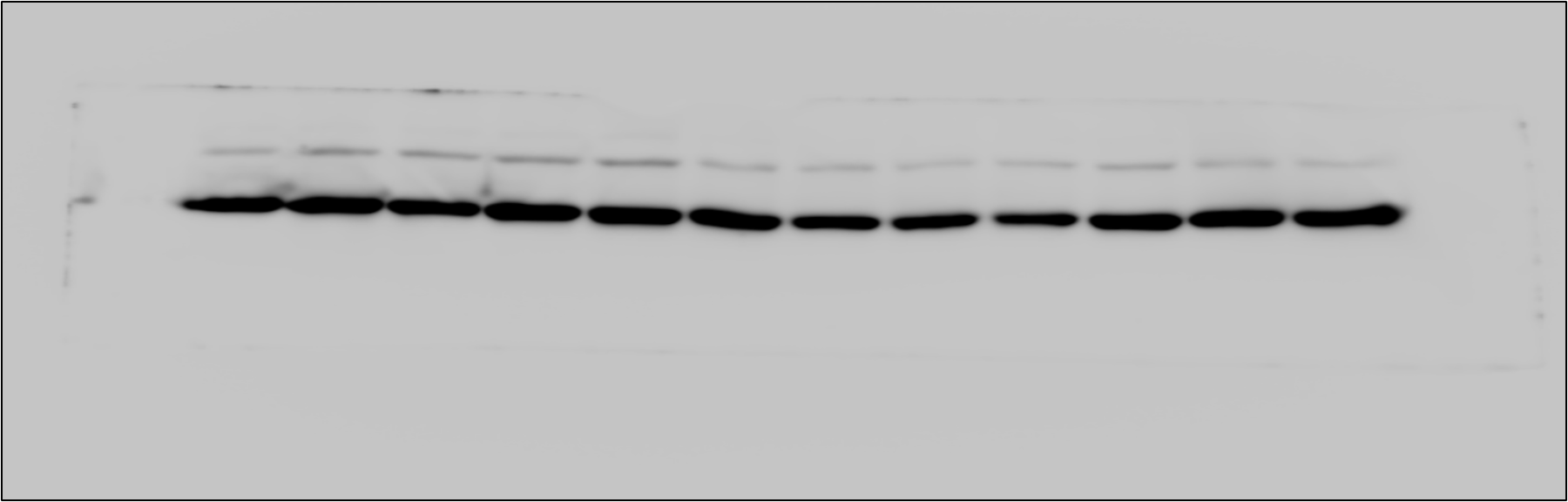

Supplement: Figure 4—source data 2. [file elife-98357-fig4-data2.zip › Figure 4-source data 2/4 M-liver-Actin.tif]

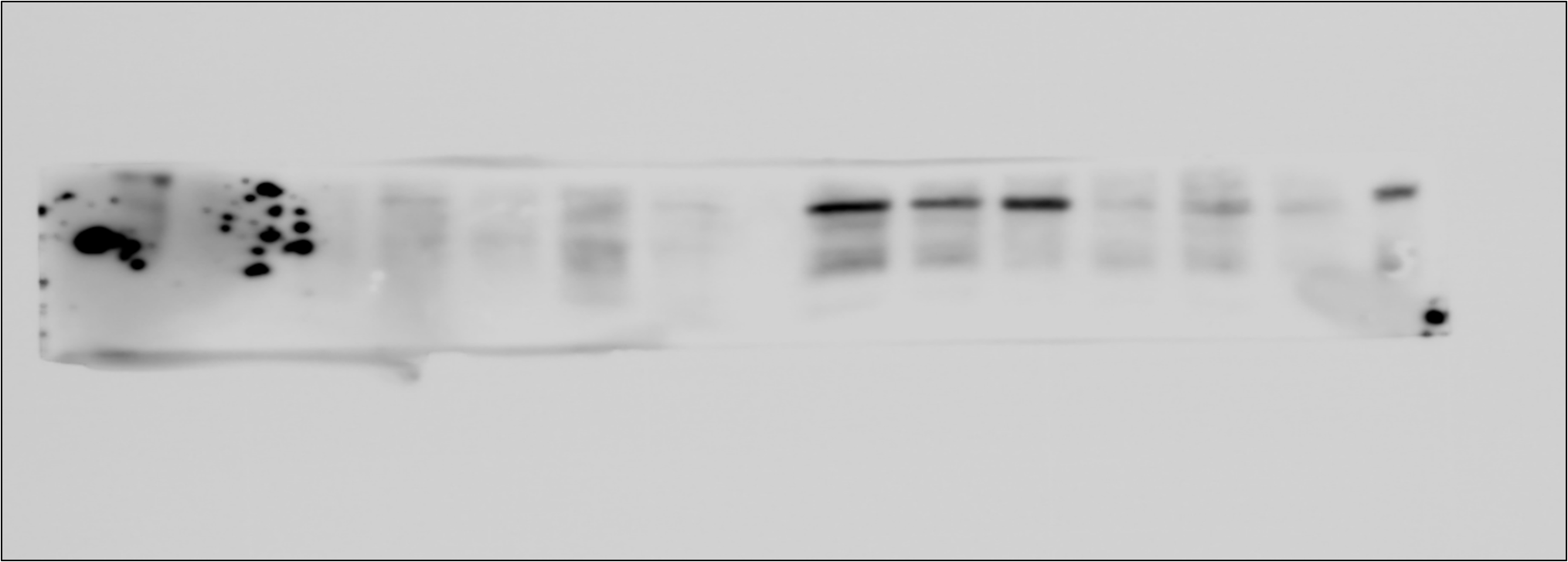

Supplement: Figure 4—source data 2. [file elife-98357-fig4-data2.zip › Figure 4-source data 2/4 M-liver-CDK2.tif]

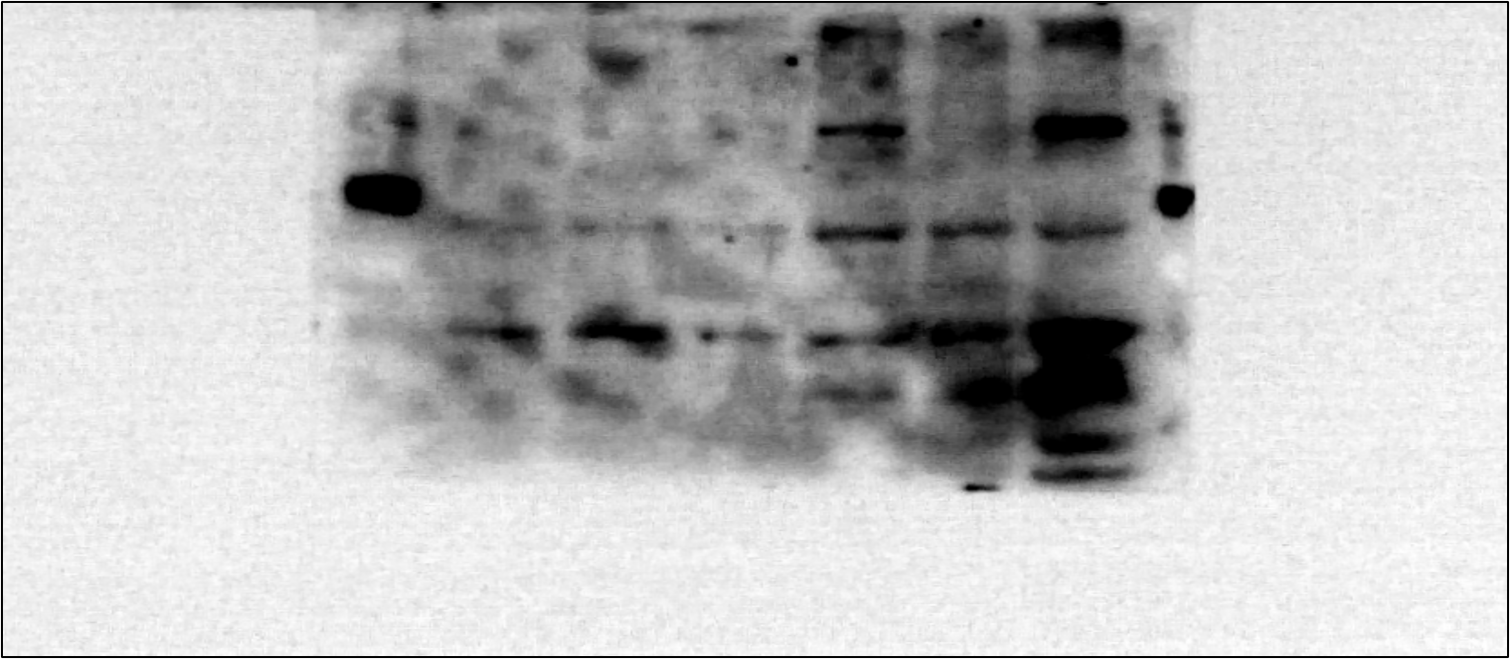

Supplement: Figure 4—source data 2. [file elife-98357-fig4-data2.zip › Figure 4-source data 2/4 M-liver-TBK1.tif]

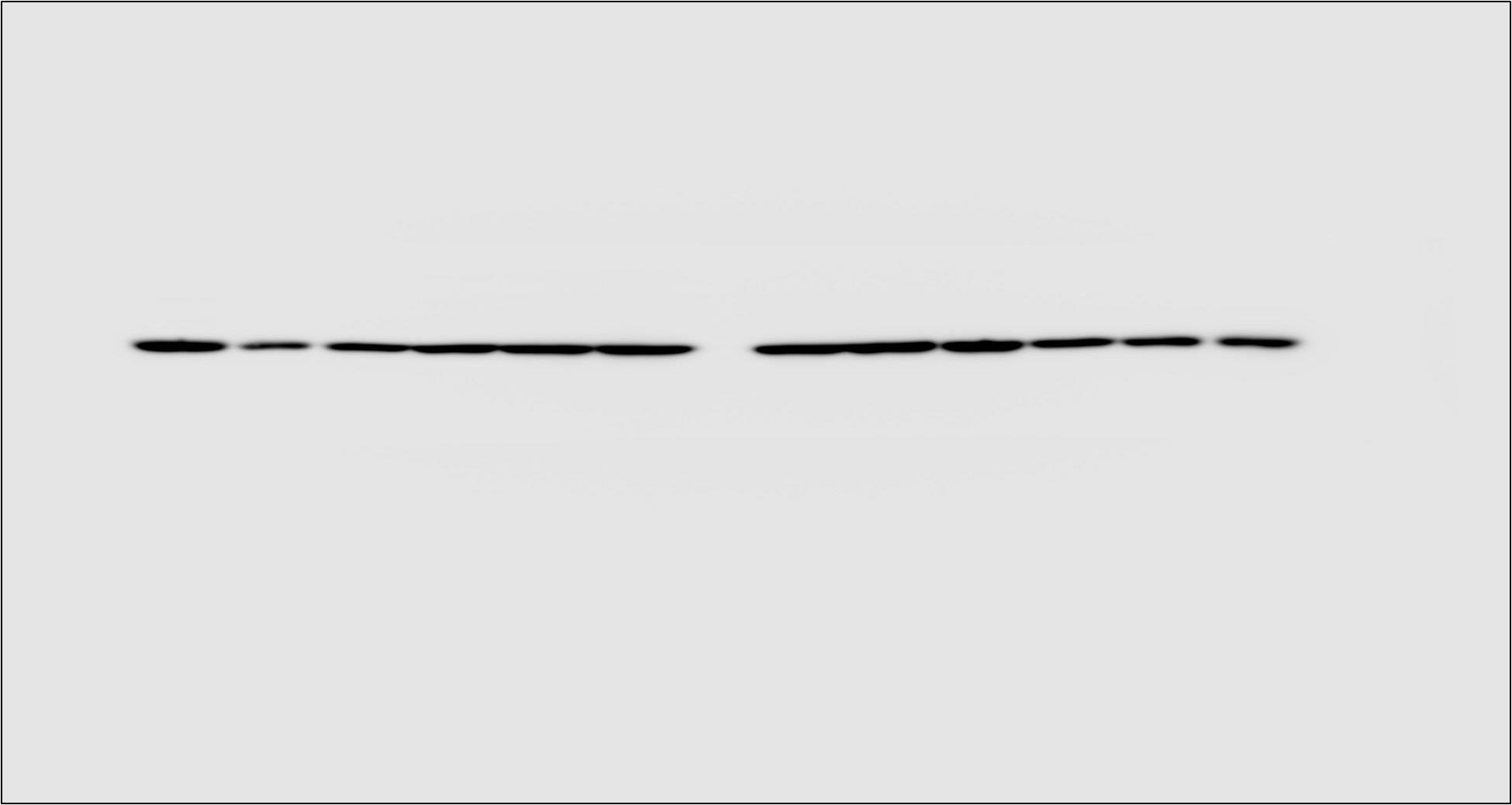

Supplement: Figure 4—source data 2. [file elife-98357-fig4-data2.zip › Figure 4-source data 2/4 M-Spleen-Actin.tif]

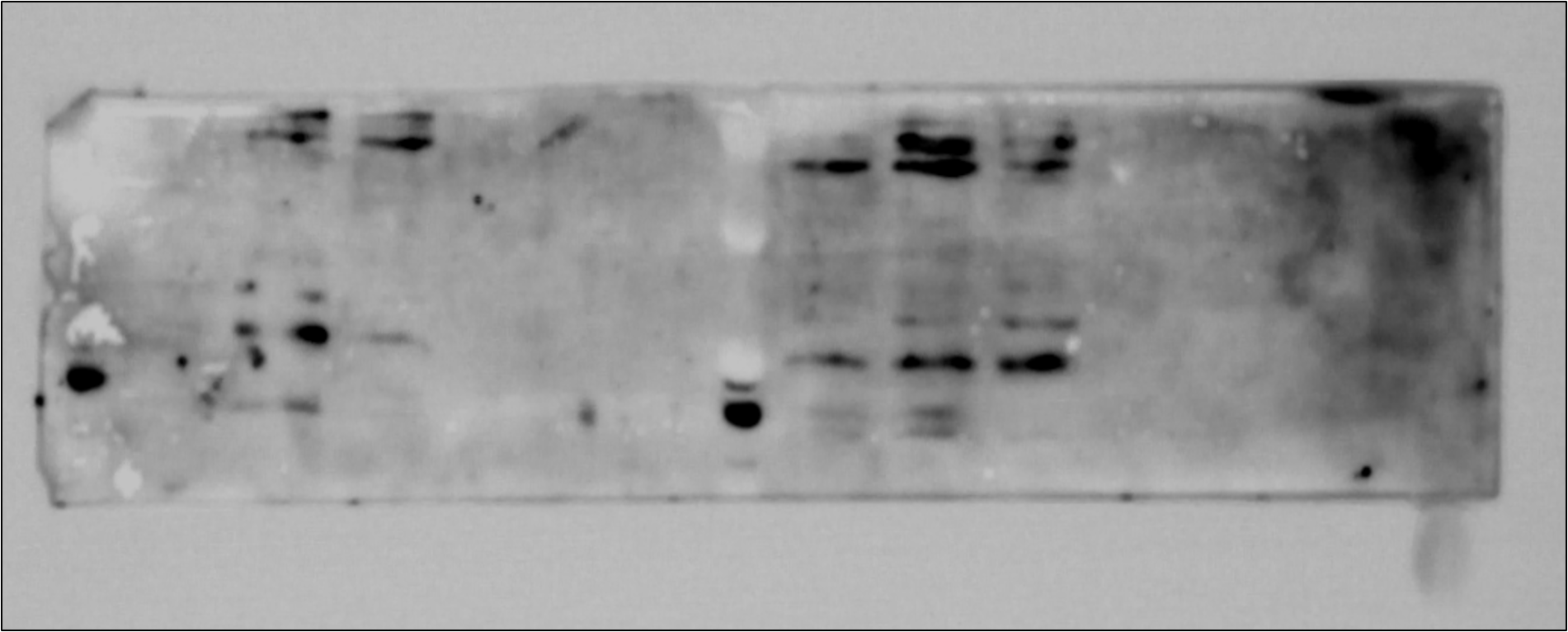

Supplement: Figure 4—source data 2. [file elife-98357-fig4-data2.zip › Figure 4-source data 2/4 M-Spleen-CDK2.tif]

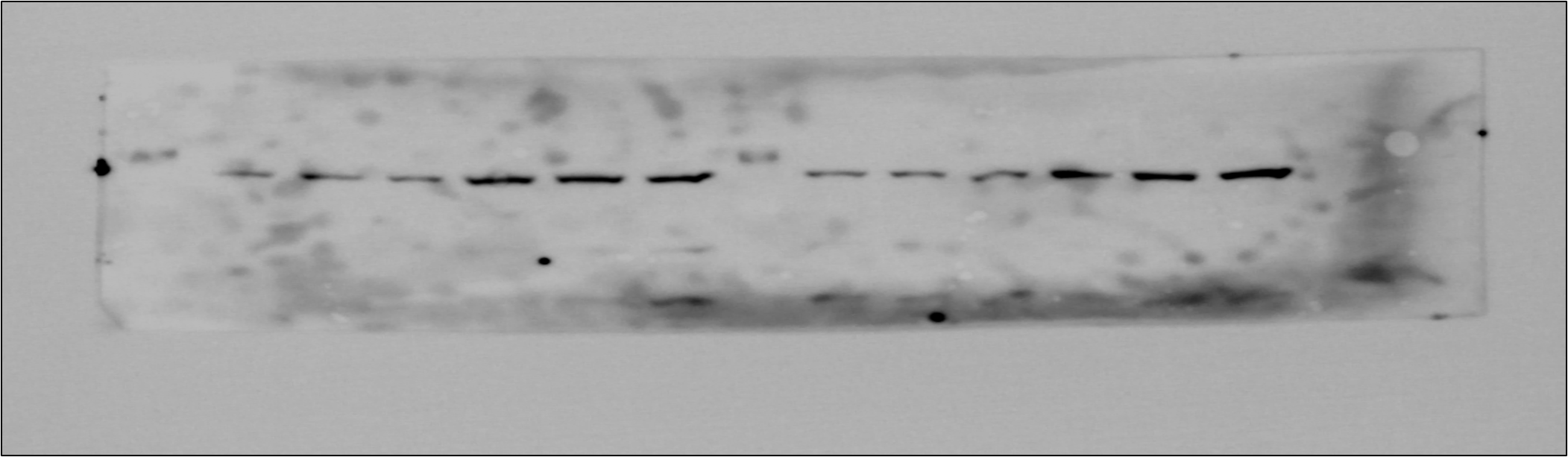

Supplement: Figure 4—source data 2. [file elife-98357-fig4-data2.zip › Figure 4-source data 2/4 M-Spleen-TBK1.tif]

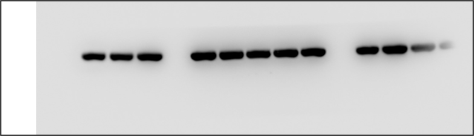

Supplement: Figure 4—source data 2. [file elife-98357-fig4-data2.zip › Figure 4-source data 2/4 O-Actin.tif]

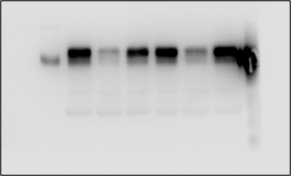

Supplement: Figure 4—source data 2. [file elife-98357-fig4-data2.zip › Figure 4-source data 2/4 O-G.tif]

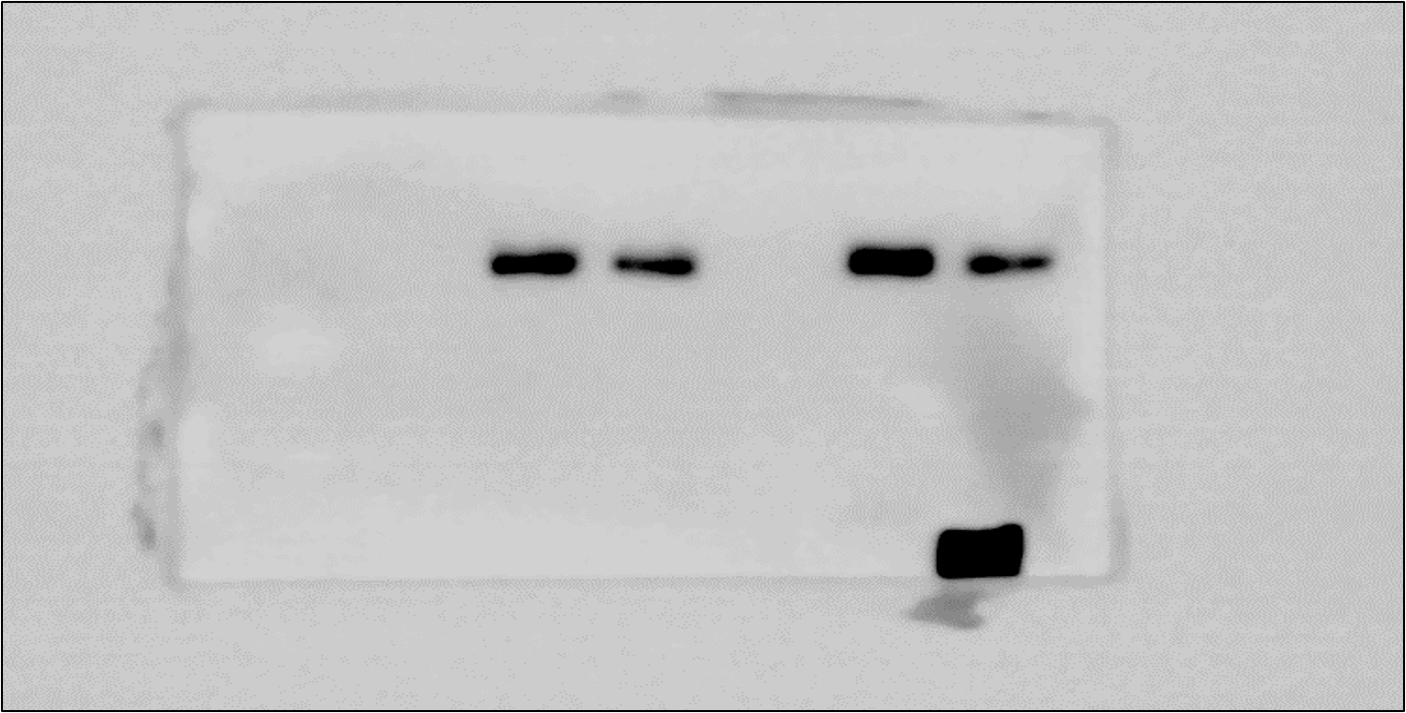

Supplement: Figure 4—source data 2. [file elife-98357-fig4-data2.zip › Figure 4-source data 2/4 O-Myc.tif]

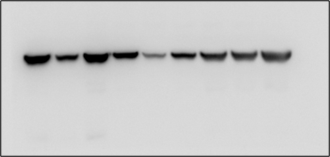

Supplement: Figure 4—source data 2. [file elife-98357-fig4-data2.zip › Figure 4-source data 2/4 O-N.tif]

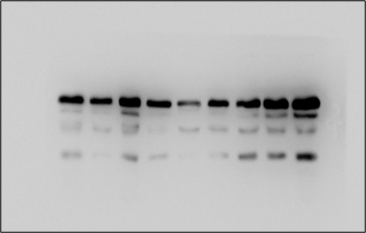

Supplement: Figure 4—source data 2. [file elife-98357-fig4-data2.zip › Figure 4-source data 2/4 O-P.tif]

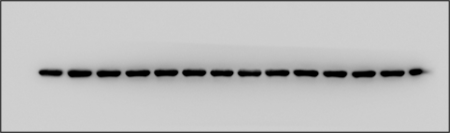

Supplement: Figure 5—source data 2. [file elife-98357-fig5-data2.zip › Figure 5-source data 2/5 B-Actin.tif]

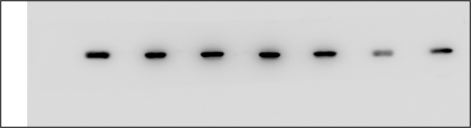

Supplement: Figure 5—source data 2. [file elife-98357-fig5-data2.zip › Figure 5-source data 2/5 B-HA.tif]

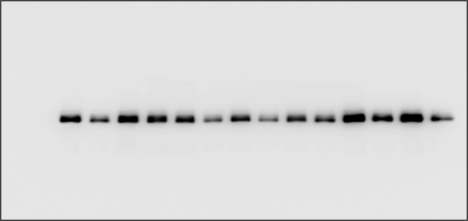

Supplement: Figure 5—source data 2. [file elife-98357-fig5-data2.zip › Figure 5-source data 2/5 B-Myc.tif]

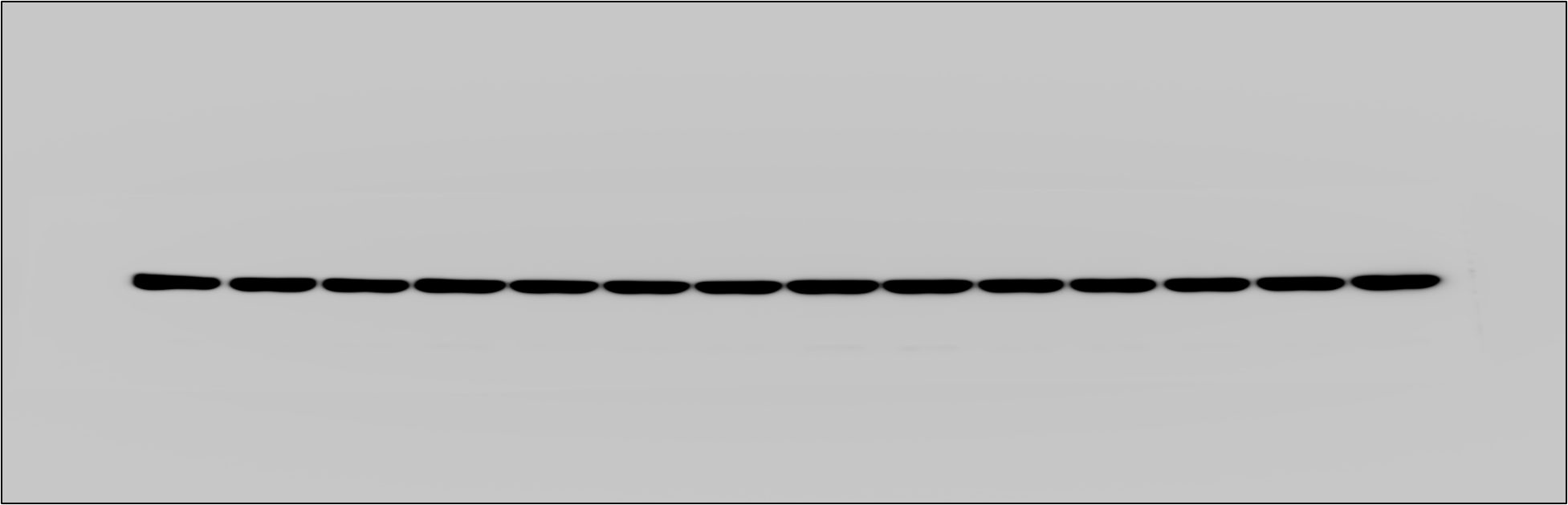

Supplement: Figure 5—source data 2. [file elife-98357-fig5-data2.zip › Figure 5-source data 2/5 C-Actin.tif]

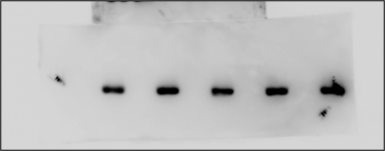

Supplement: Figure 5—source data 2. [file elife-98357-fig5-data2.zip › Figure 5-source data 2/5 C-HA.tif]

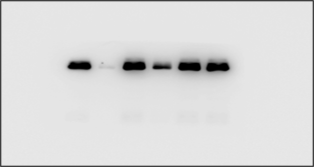

Supplement: Figure 5—source data 2. [file elife-98357-fig5-data2.zip › Figure 5-source data 2/5 C-Myc.tif]

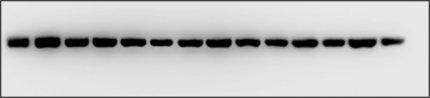

Supplement: Figure 5—source data 2. [file elife-98357-fig5-data2.zip › Figure 5-source data 2/5 D-Actin.tif]

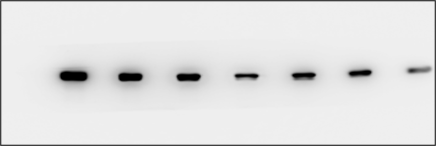

Supplement: Figure 5—source data 2. [file elife-98357-fig5-data2.zip › Figure 5-source data 2/5 D-HA.tif]

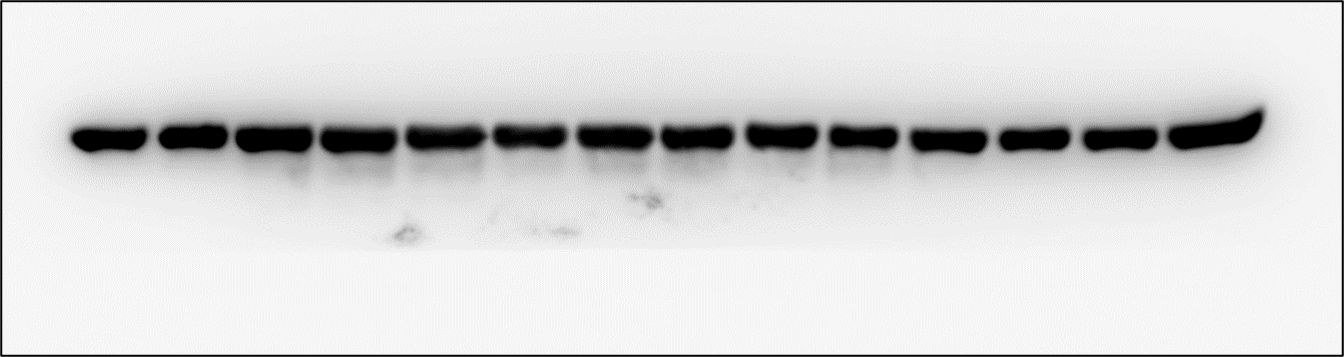

Supplement: Figure 5—source data 2. [file elife-98357-fig5-data2.zip › Figure 5-source data 2/5 D-poly IC-Actin.tif]

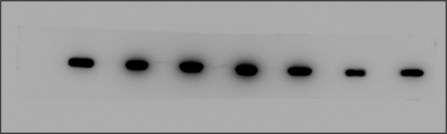

Supplement: Figure 5—source data 2. [file elife-98357-fig5-data2.zip › Figure 5-source data 2/5 D-poly IC-HA.tif]

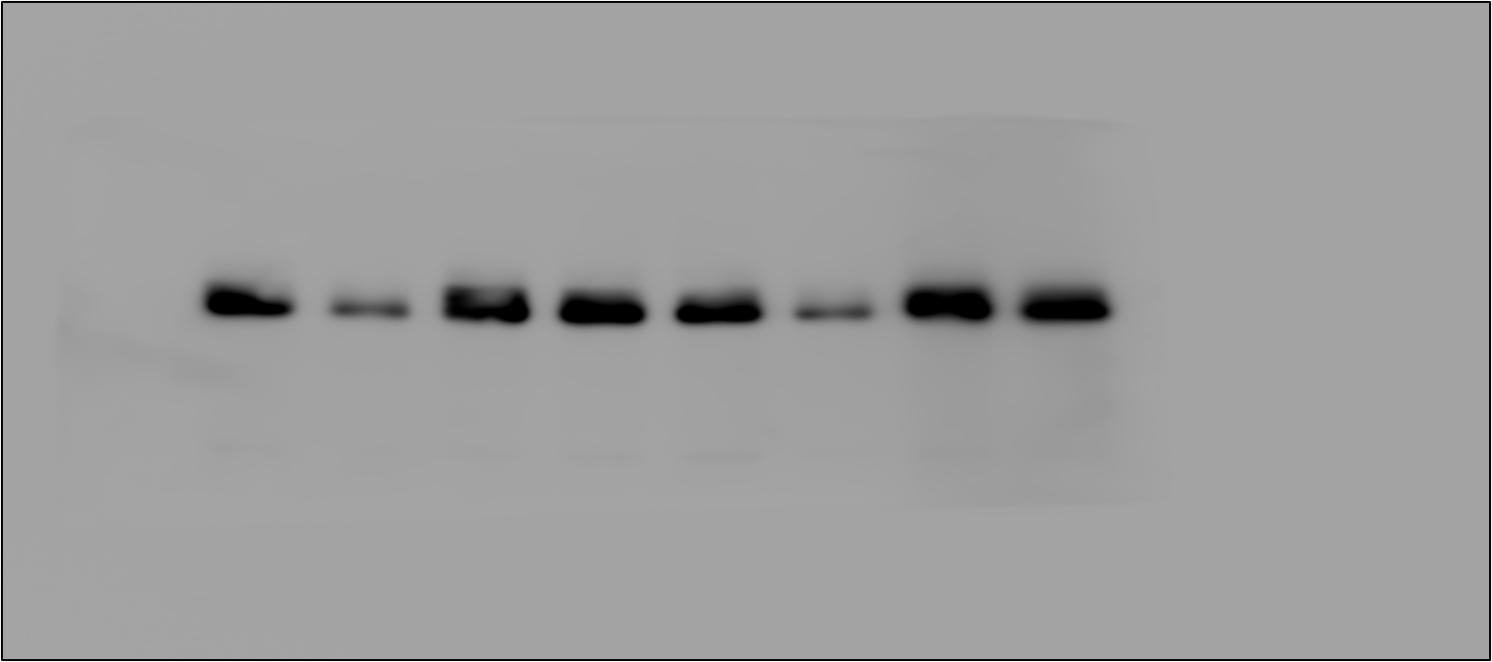

Supplement: Figure 5—source data 2. [file elife-98357-fig5-data2.zip › Figure 5-source data 2/5 D-poly IC-TBK1.tif]

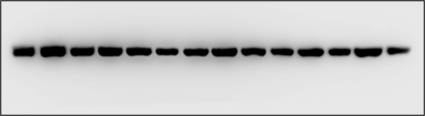

Supplement: Figure 5—source data 2. [file elife-98357-fig5-data2.zip › Figure 5-source data 2/5 D-SVCV-Actin.tif]

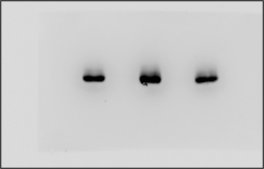

Supplement: Figure 5—source data 2. [file elife-98357-fig5-data2.zip › Figure 5-source data 2/5 D-SVCV-HA.tif]

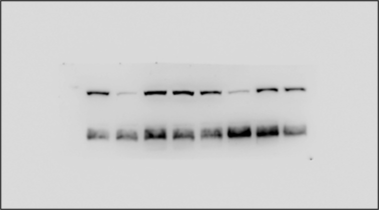

Supplement: Figure 5—source data 2. [file elife-98357-fig5-data2.zip › Figure 5-source data 2/5 D-SVCV-TBK1.tif]

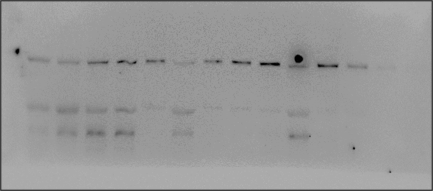

Supplement: Figure 5—source data 2. [file elife-98357-fig5-data2.zip › Figure 5-source data 2/5 D-TBK1.tif]

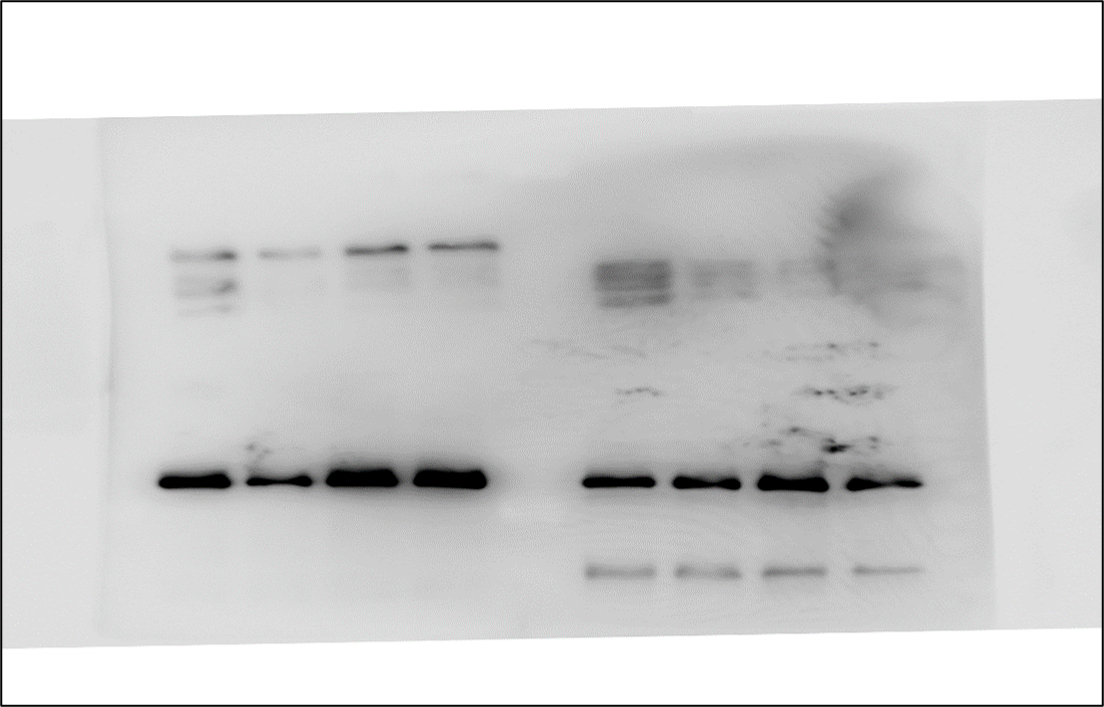

Supplement: Figure 5—source data 2. [file elife-98357-fig5-data2.zip › Figure 5-source data 2/5 E-IP-Myc.tif]

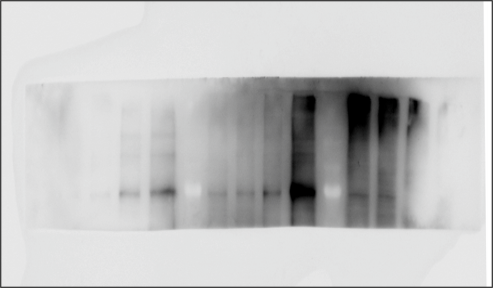

Supplement: Figure 5—source data 2. [file elife-98357-fig5-data2.zip › Figure 5-source data 2/5 E-IP-TBK1-HA-Ub.tif]

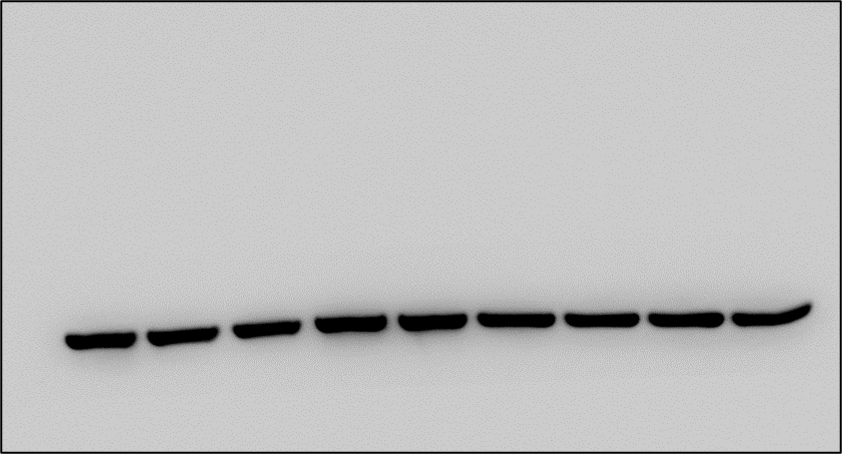

Supplement: Figure 5—source data 2. [file elife-98357-fig5-data2.zip › Figure 5-source data 2/5 E-WCL-Actin.tif]

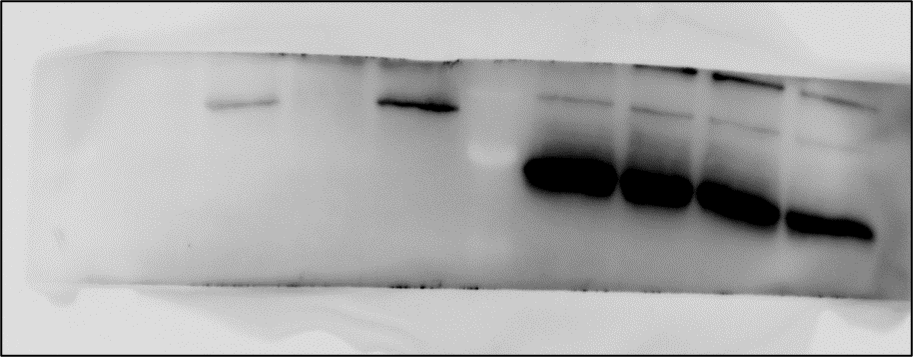

Supplement: Figure 5—source data 2. [file elife-98357-fig5-data2.zip › Figure 5-source data 2/5 E-WCL-HA.tif]

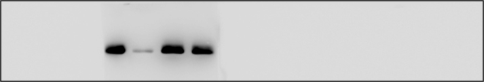

Supplement: Figure 5—source data 2. [file elife-98357-fig5-data2.zip › Figure 5-source data 2/5 E-WCL-Myc.tif]

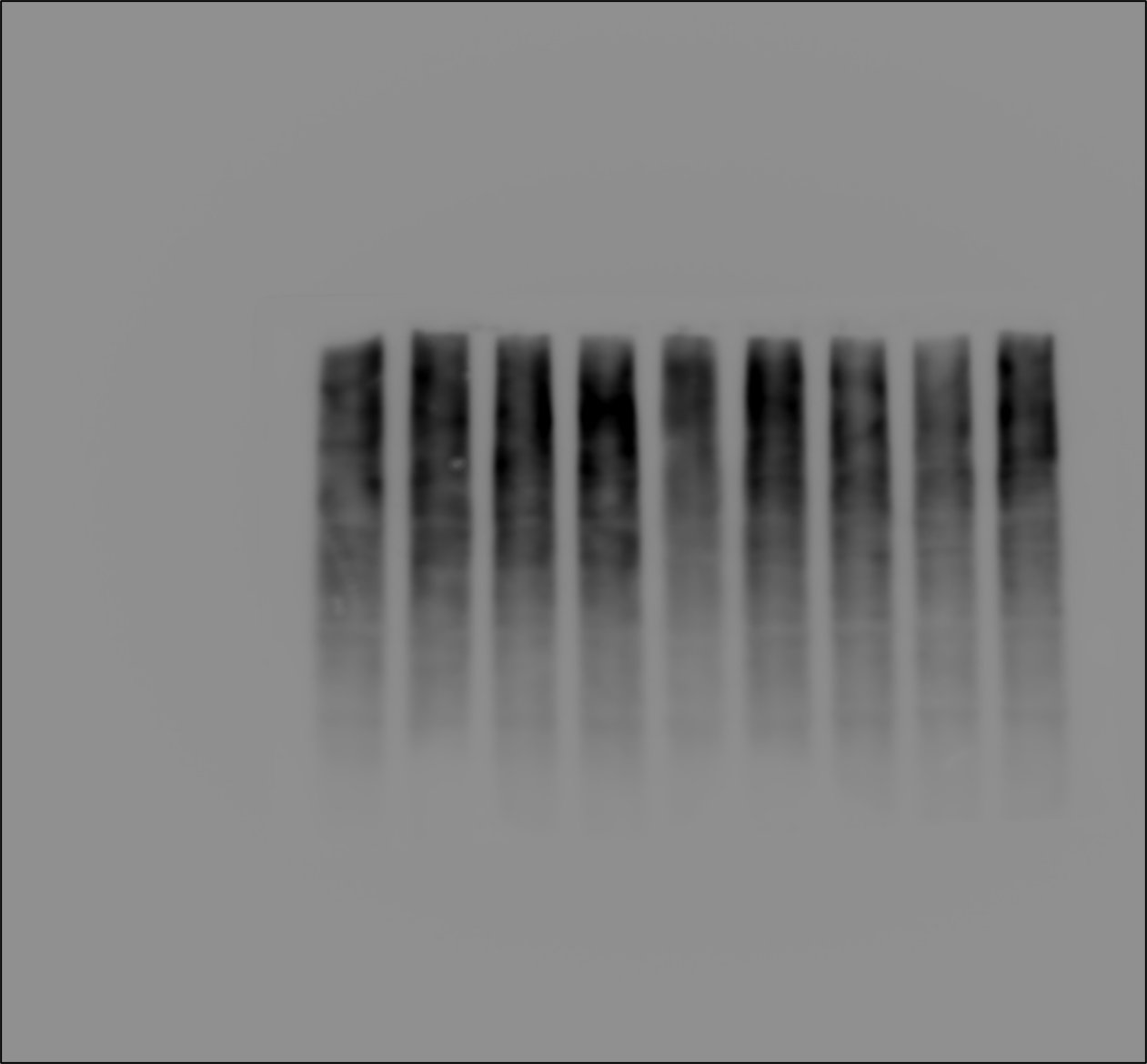

Supplement: Figure 5—source data 2. [file elife-98357-fig5-data2.zip › Figure 5-source data 2/5 E-WCL-TBK1-HA-Ub.tif]

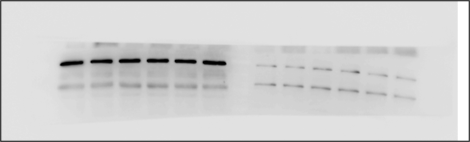

Supplement: Figure 5—source data 2. [file elife-98357-fig5-data2.zip › Figure 5-source data 2/5 G-IP-Myc.tif]

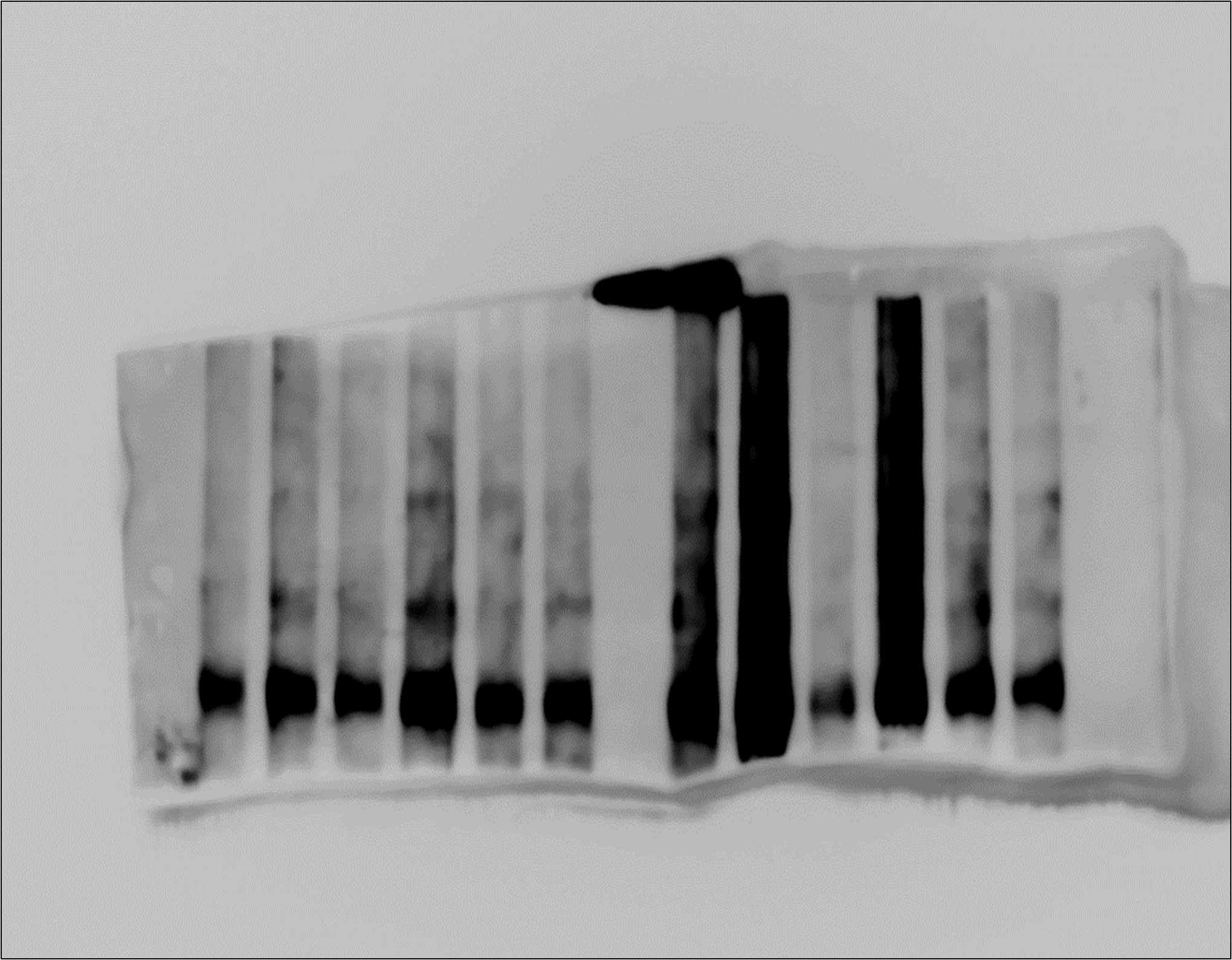

Supplement: Figure 5—source data 2. [file elife-98357-fig5-data2.zip › Figure 5-source data 2/5 G-IP-TBK1-HA-Ub.tif]

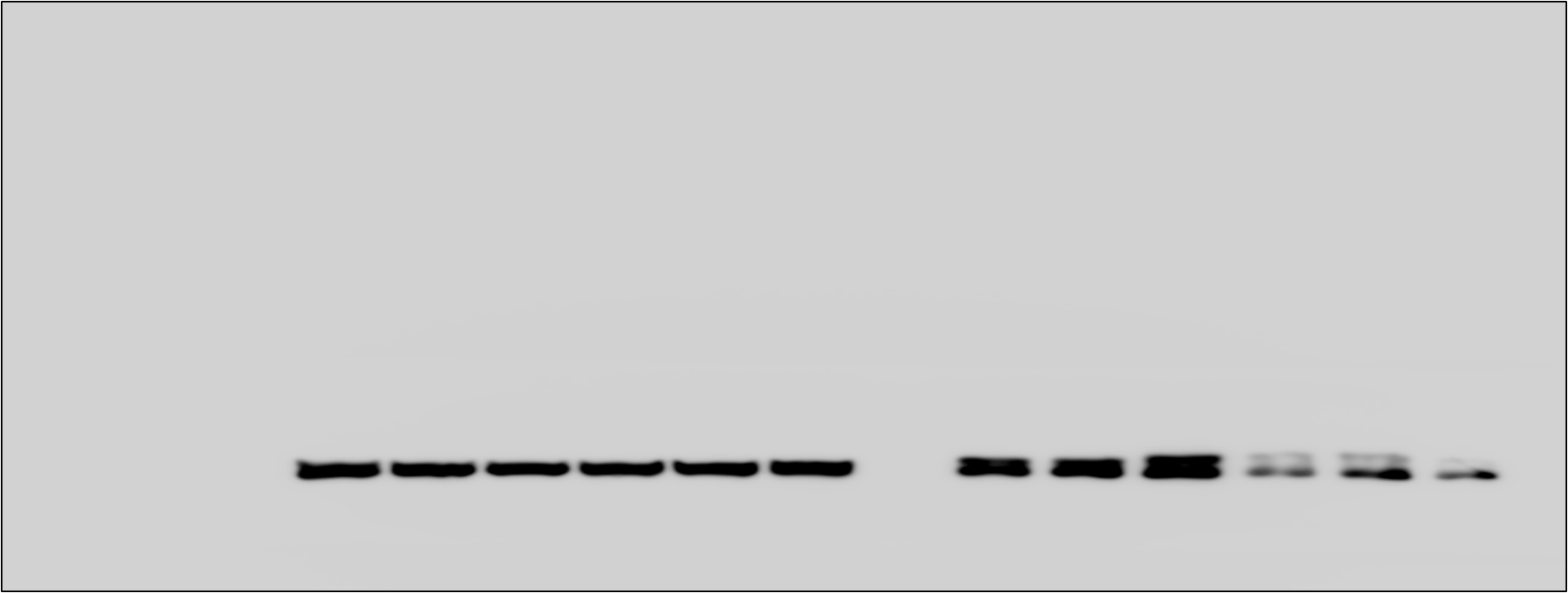

Supplement: Figure 5—source data 2. [file elife-98357-fig5-data2.zip › Figure 5-source data 2/5 G-WCL-Actin.tif]

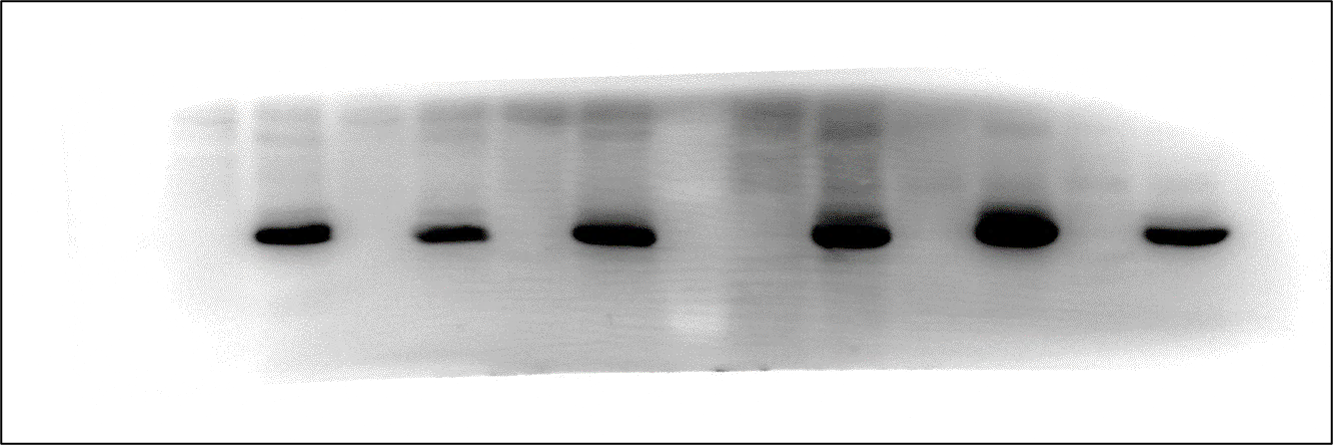

Supplement: Figure 5—source data 2. [file elife-98357-fig5-data2.zip › Figure 5-source data 2/5 G-WCL-HA.tif]
