# Supplementary material for: Fish CDK2 recruits Dtx4 to degrade TBK1 through ubiquitination in the antiviral response
Source: eLife. 2026 Jan 14;13:RP98357. doi: 10.7554/eLife.98357 (PMC12803515; doi:10.7554/eLife.98357)
Supplement: Figure 2—source data 1. [file elife-98357-fig2-data1.zip › Figure 2-source data 1/Figure 2-source data.pdf]

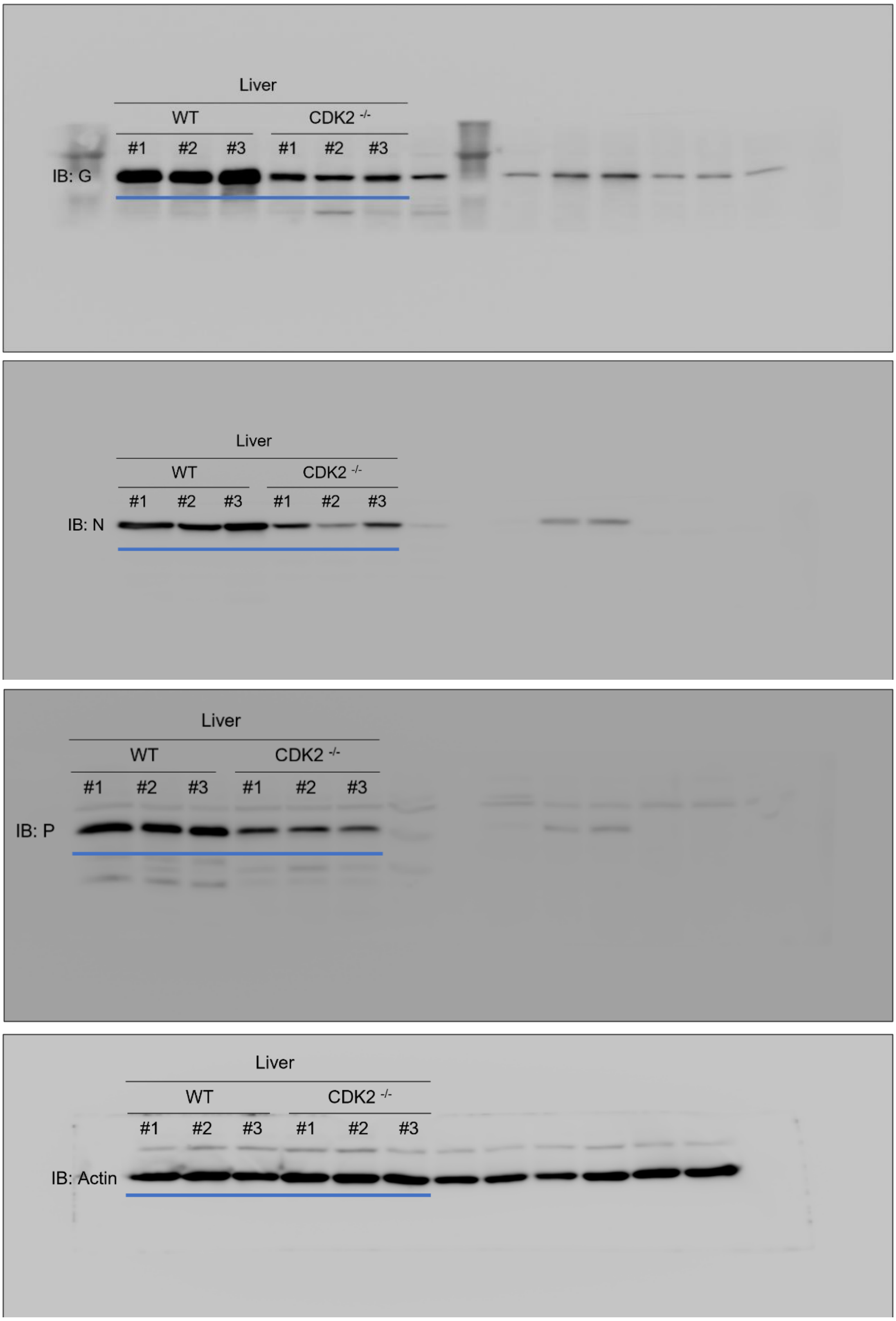

Figure 2, Source Data 1. Original membranes corresponding to Figure 2, panel D (Liver). Each membrane is labelled with the relevant information. The blue lines indicate the corresponding bands.

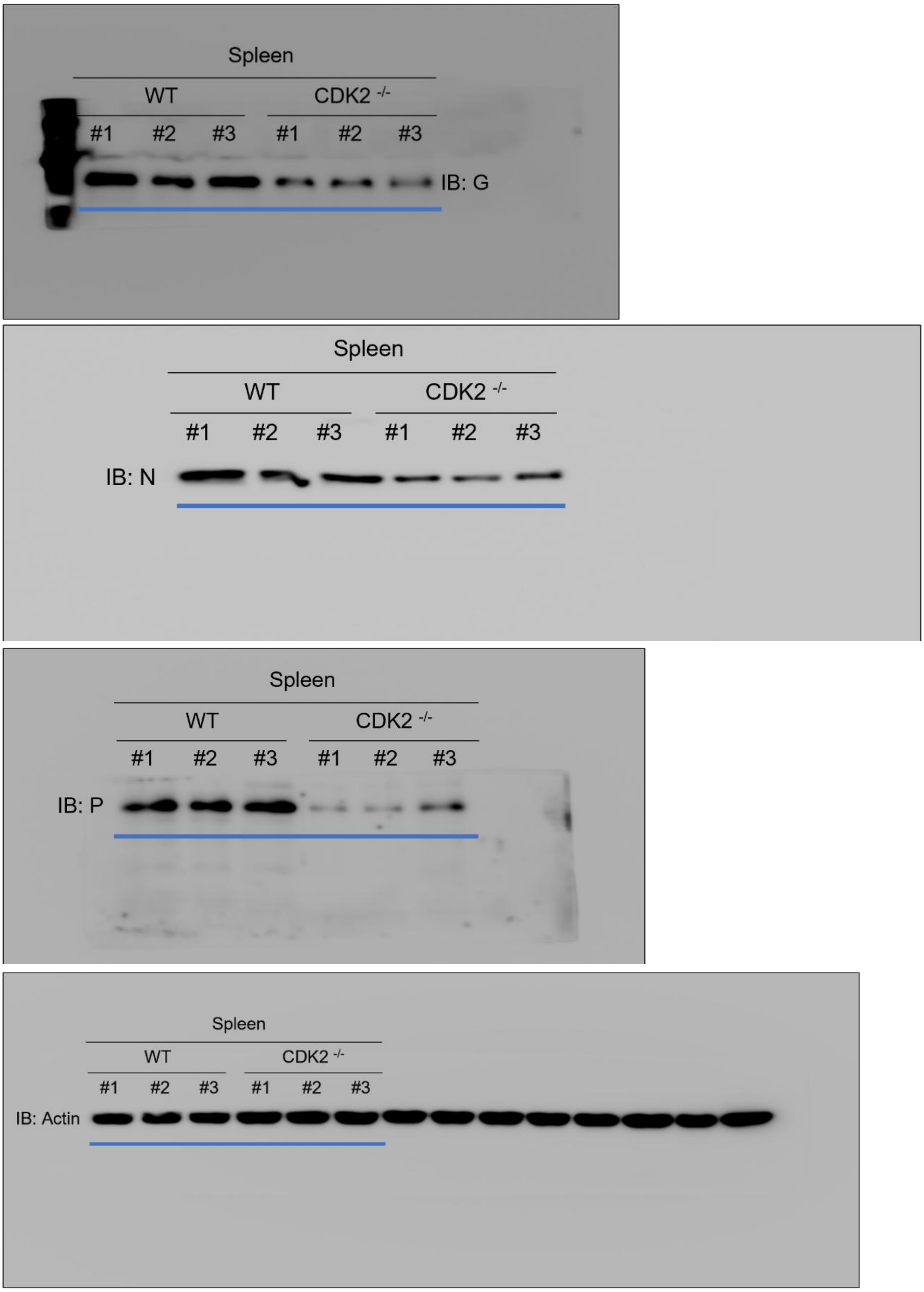

Figure 2, Source Data 1. Original membranes corresponding to Figure 2, panel D (Spleen). Each membrane is labelled with the relevant information. The blue lines indicate the corresponding bands.

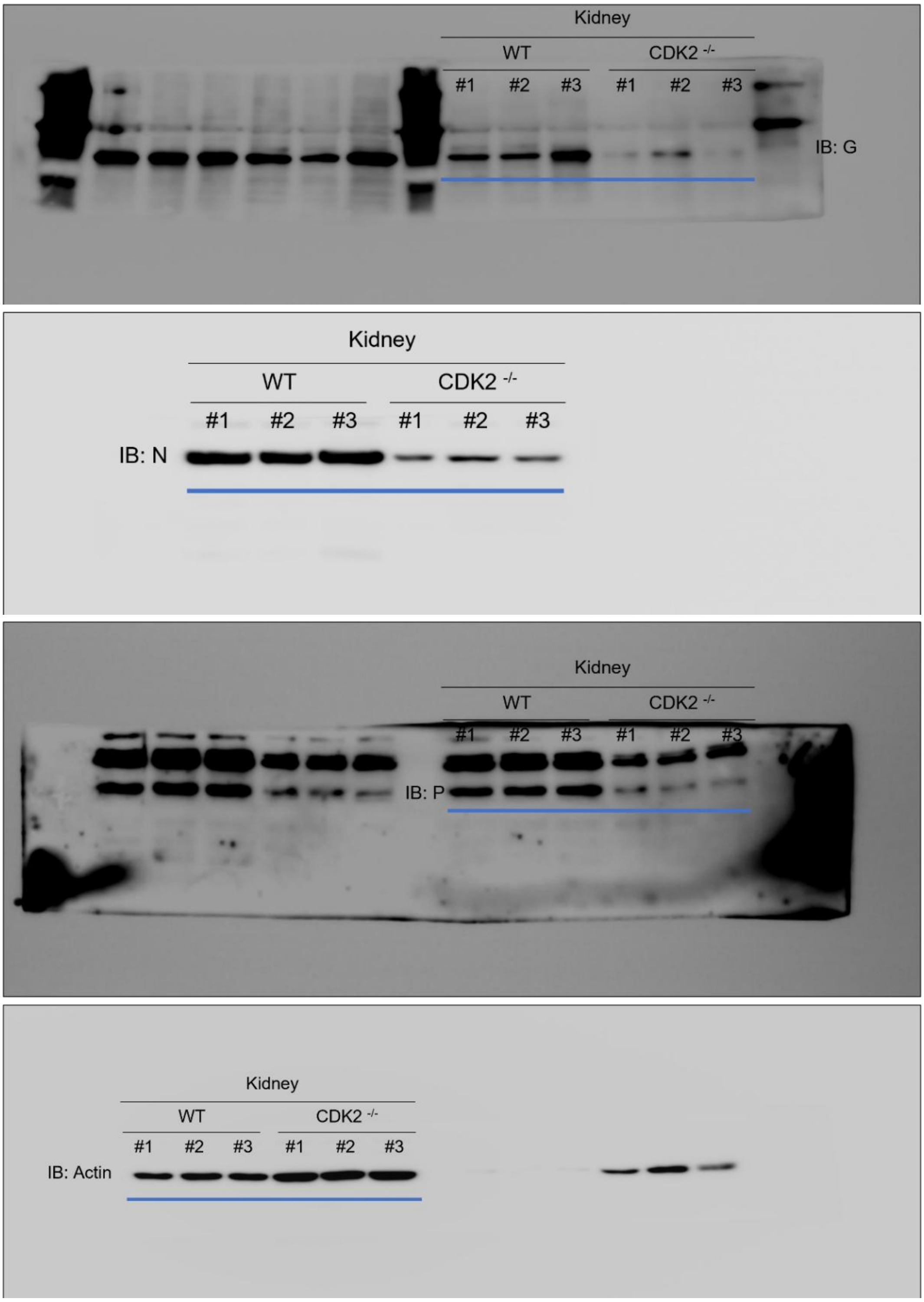

Figure 2, Source Data 1. Original membranes corresponding to Figure 2, panel D (Kidney). Each membrane is labelled with the relevant information. The blue lines indicate the corresponding bands.
