# Supplementary figures and images for: Fish CDK2 recruits Dtx4 to degrade TBK1 through ubiquitination in the antiviral response (part 2 of 3)
Source: eLife. 2026 Jan 14;13:RP98357. doi: 10.7554/eLife.98357 (PMC12803515; doi:10.7554/eLife.98357)

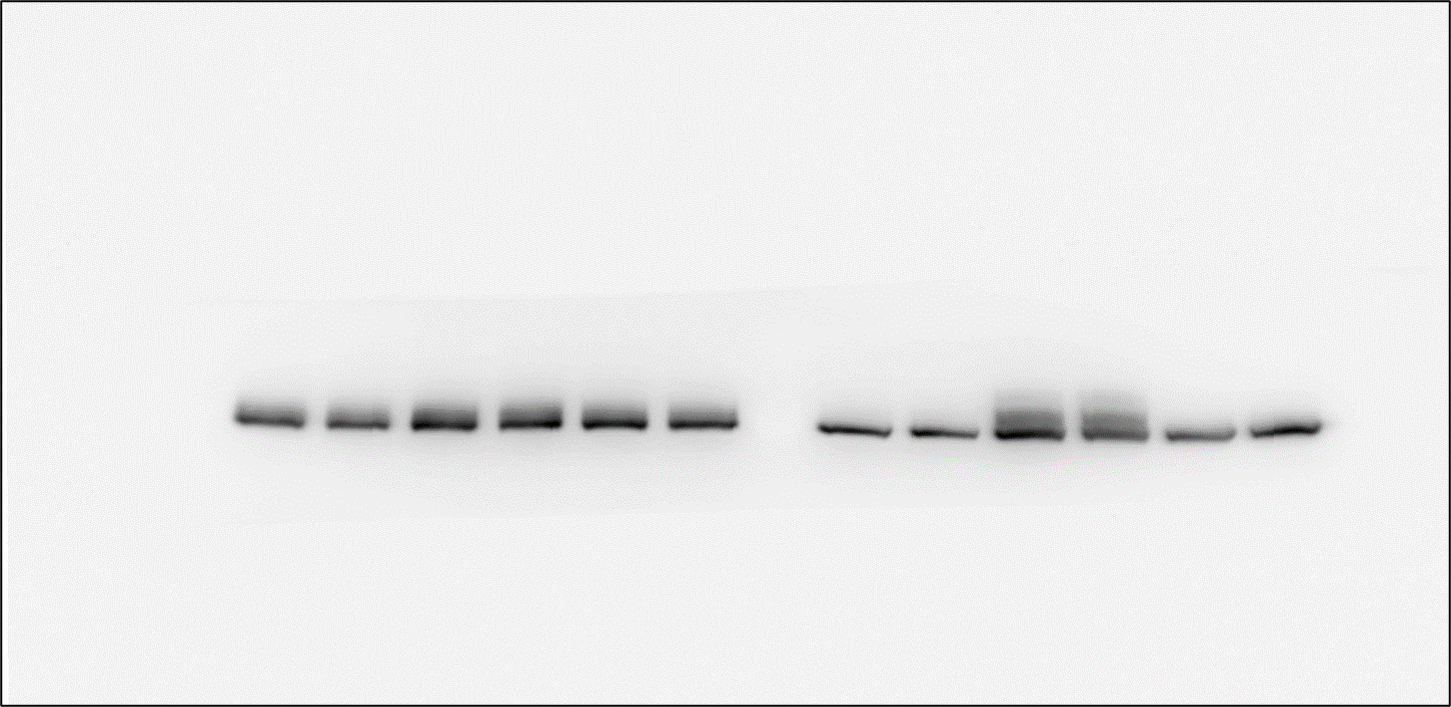

Supplement: Figure 5—source data 2. [file elife-98357-fig5-data2.zip › Figure 5-source data 2/5 G-WCL-Myc.tif]

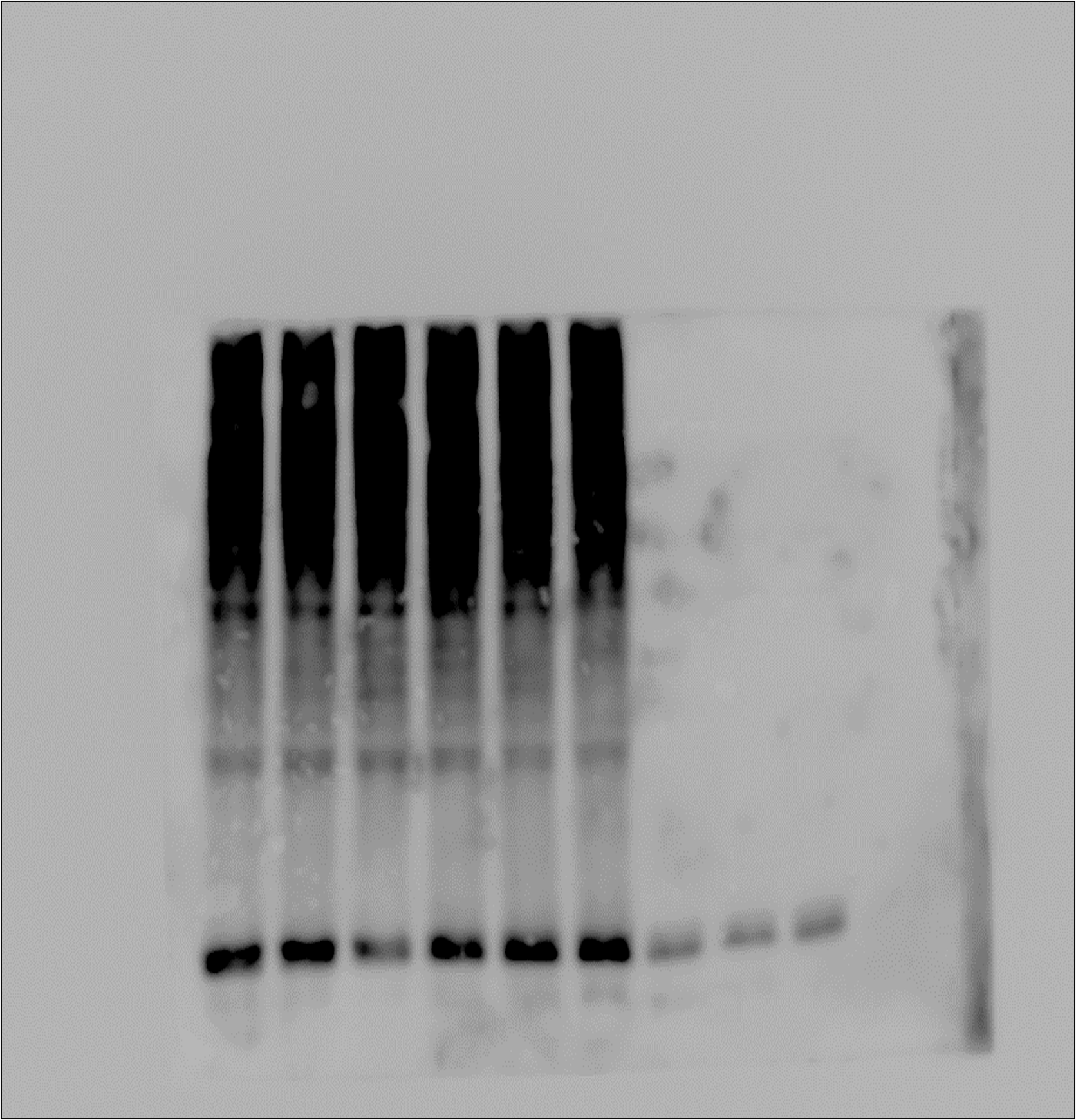

Supplement: Figure 5—source data 2. [file elife-98357-fig5-data2.zip › Figure 5-source data 2/5 G-WCL-TBK1-HA-Ub.tif]

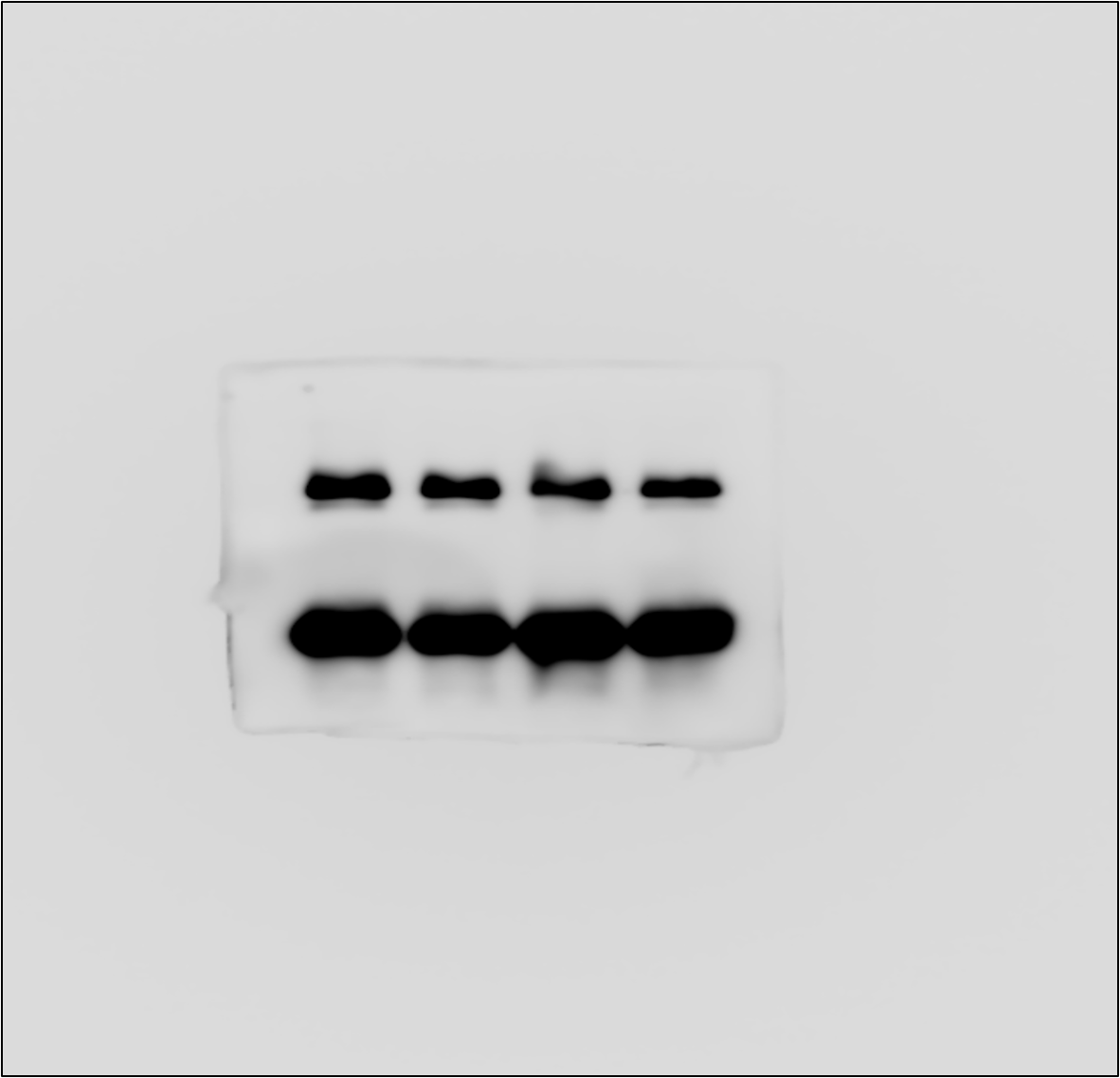

Supplement: Figure 5—source data 2. [file elife-98357-fig5-data2.zip › Figure 5-source data 2/5 H-IP-Myc.tif]

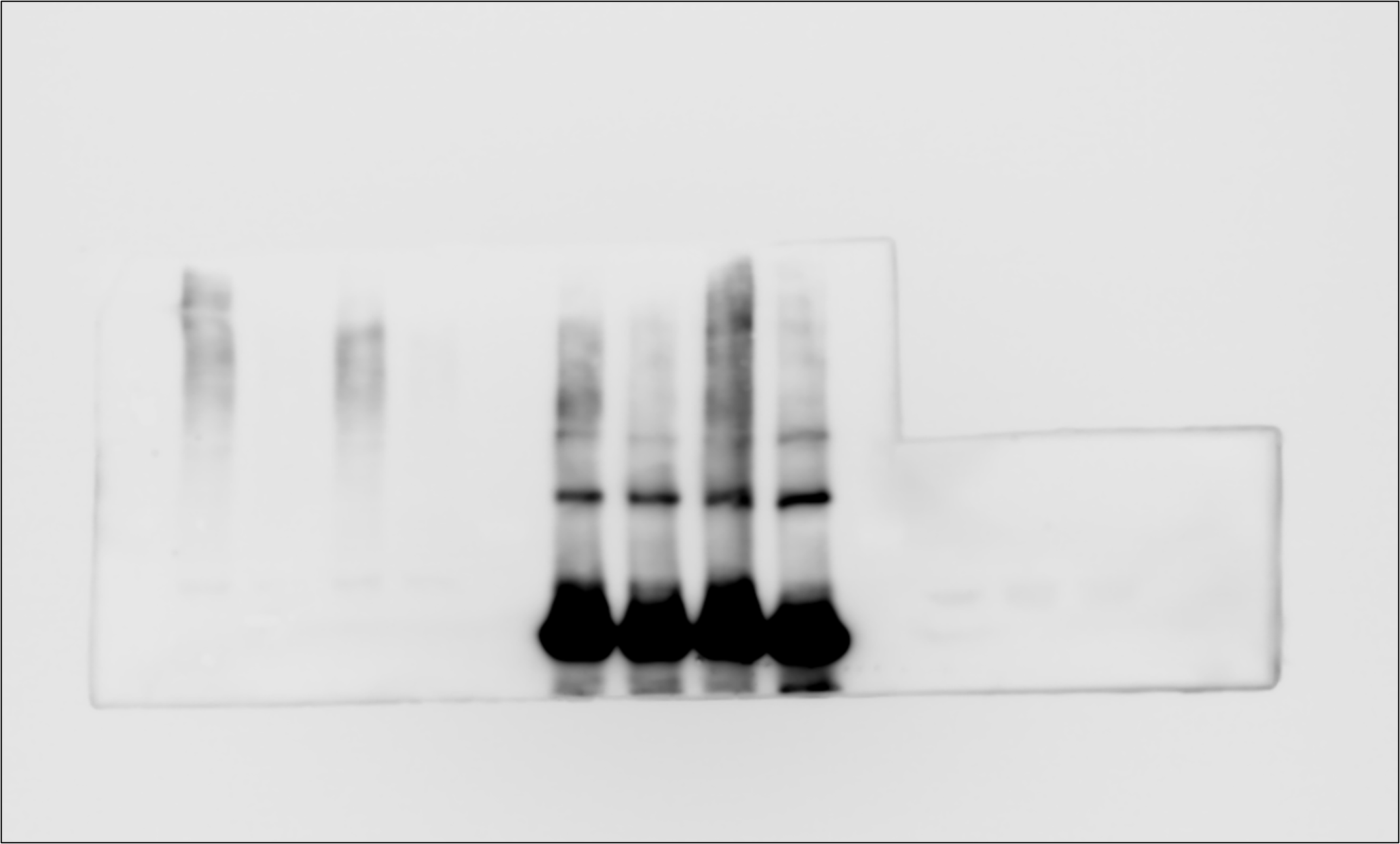

Supplement: Figure 5—source data 2. [file elife-98357-fig5-data2.zip › Figure 5-source data 2/5 H-IP-TBK1-HA-Ub.tif]

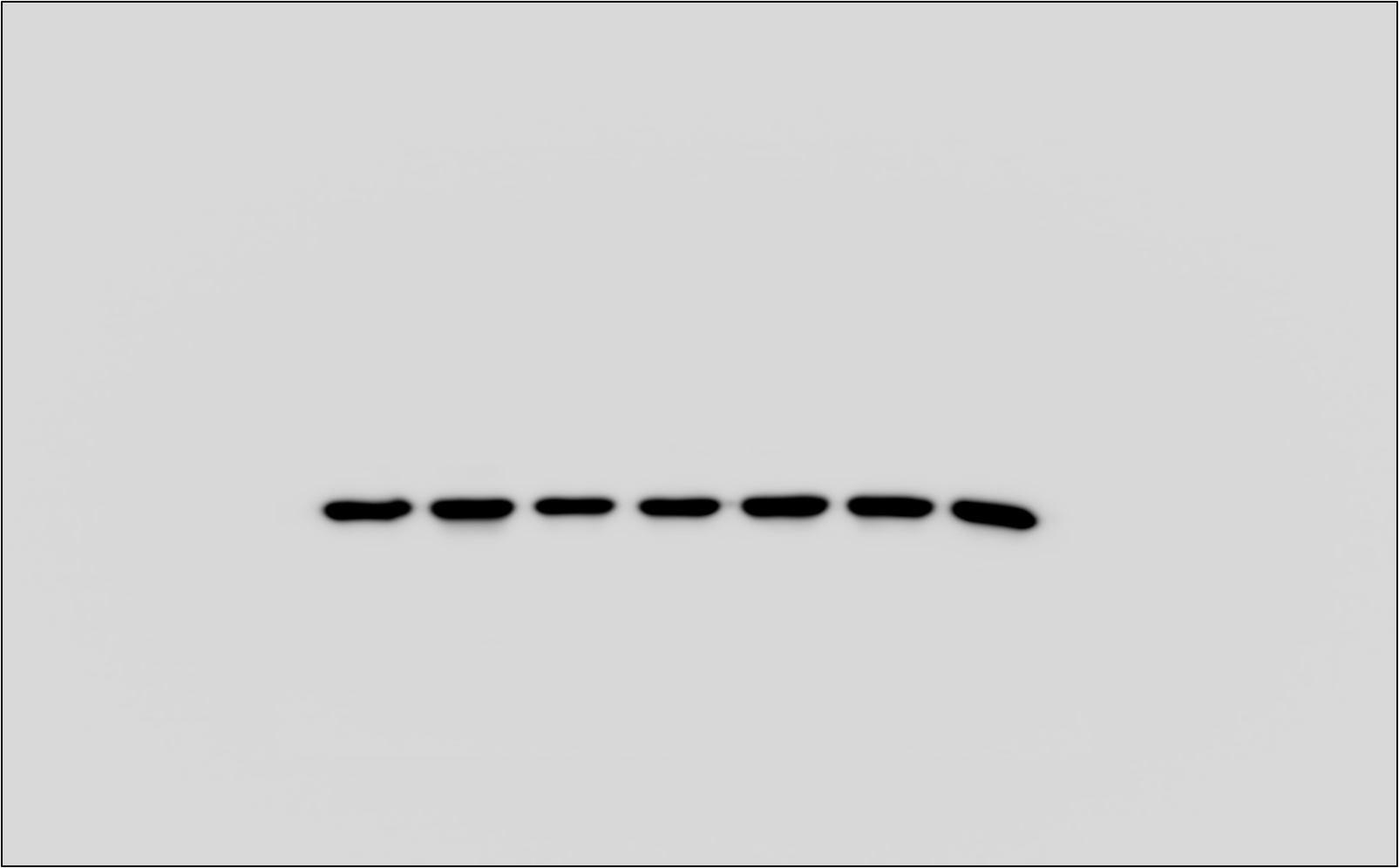

Supplement: Figure 5—source data 2. [file elife-98357-fig5-data2.zip › Figure 5-source data 2/5 H-WCL-Actin.tif]

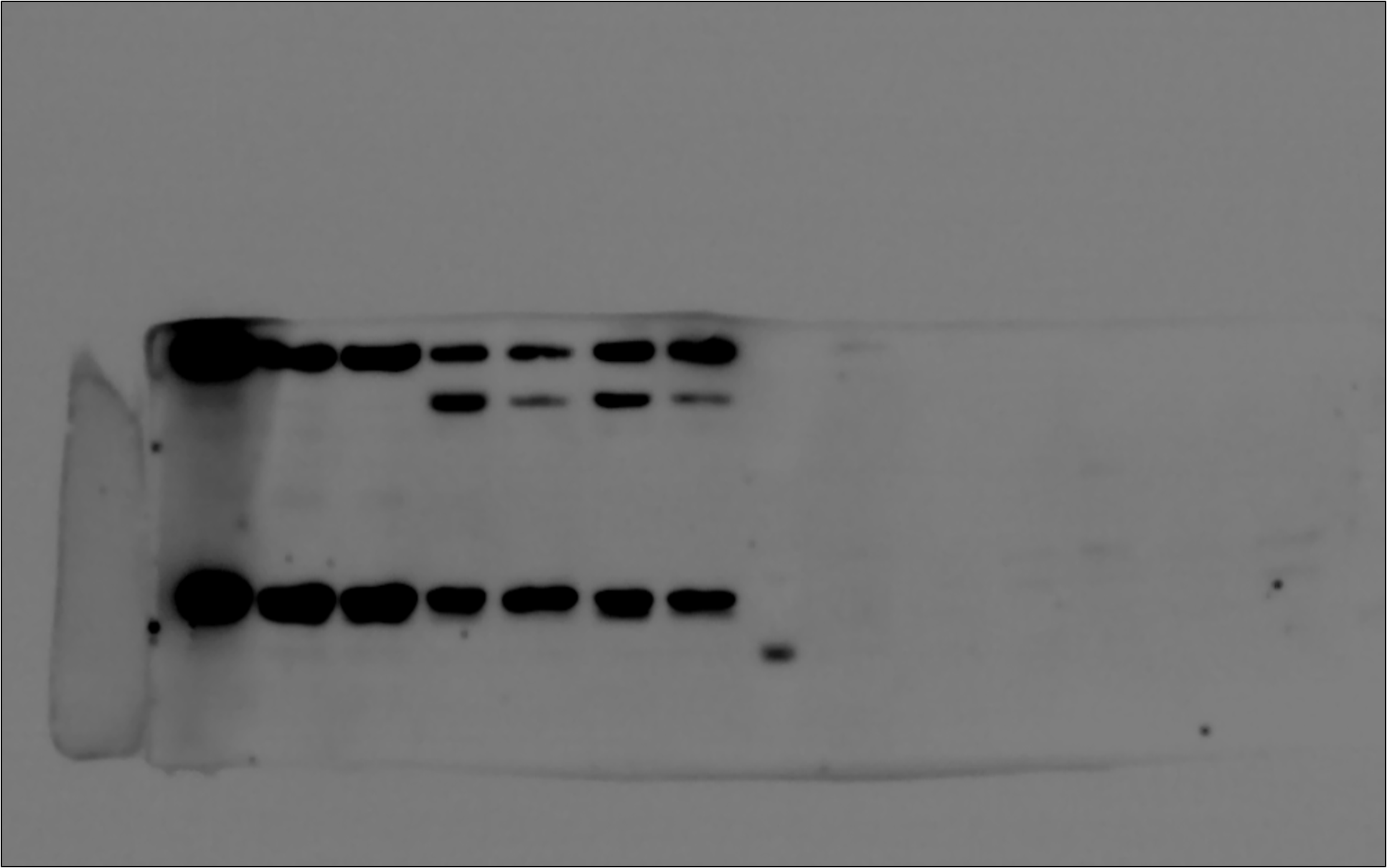

Supplement: Figure 5—source data 2. [file elife-98357-fig5-data2.zip › Figure 5-source data 2/5 H-WCL-CDK2.tif]

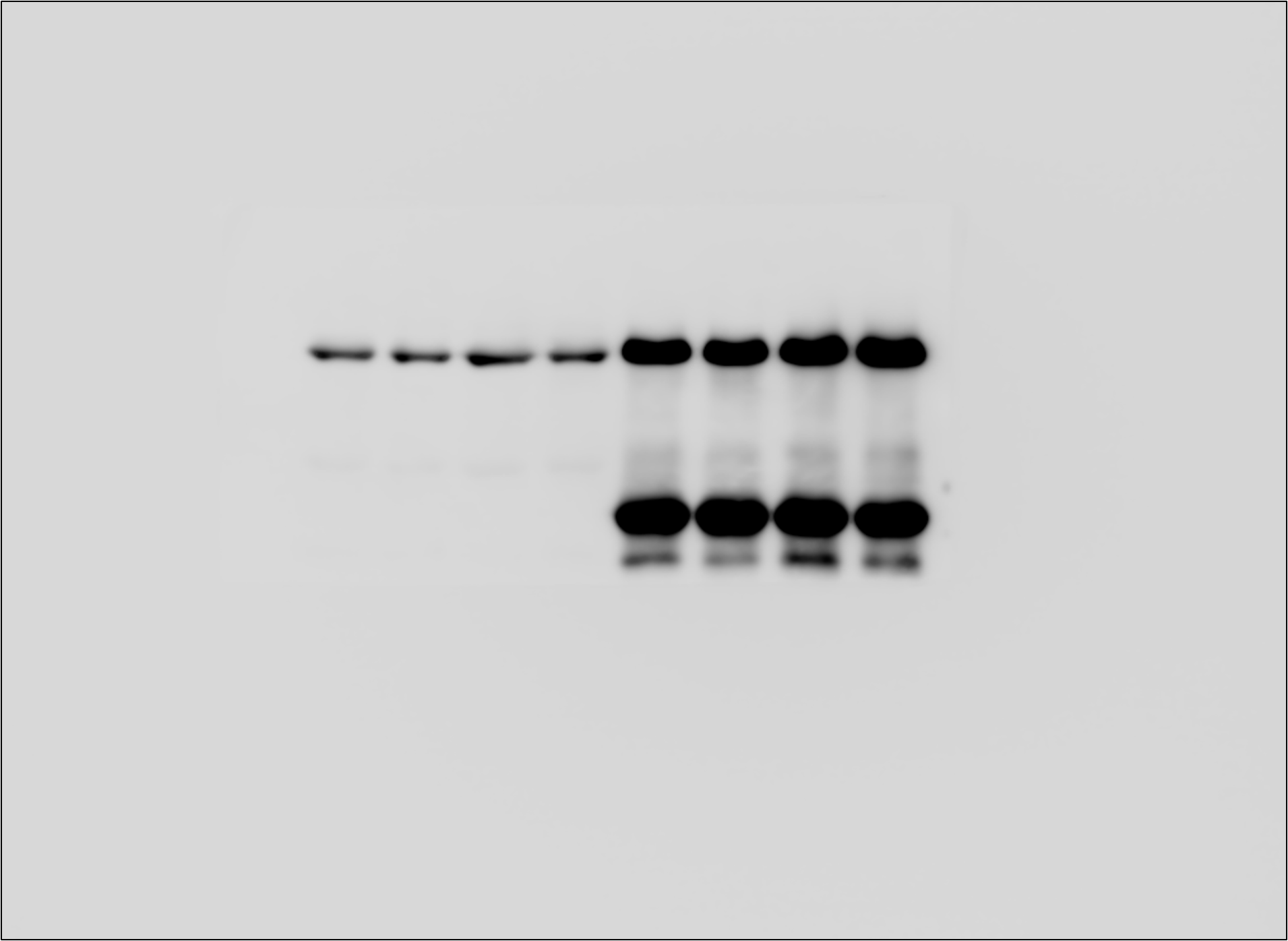

Supplement: Figure 5—source data 2. [file elife-98357-fig5-data2.zip › Figure 5-source data 2/5 H-WCL-Myc.tif]

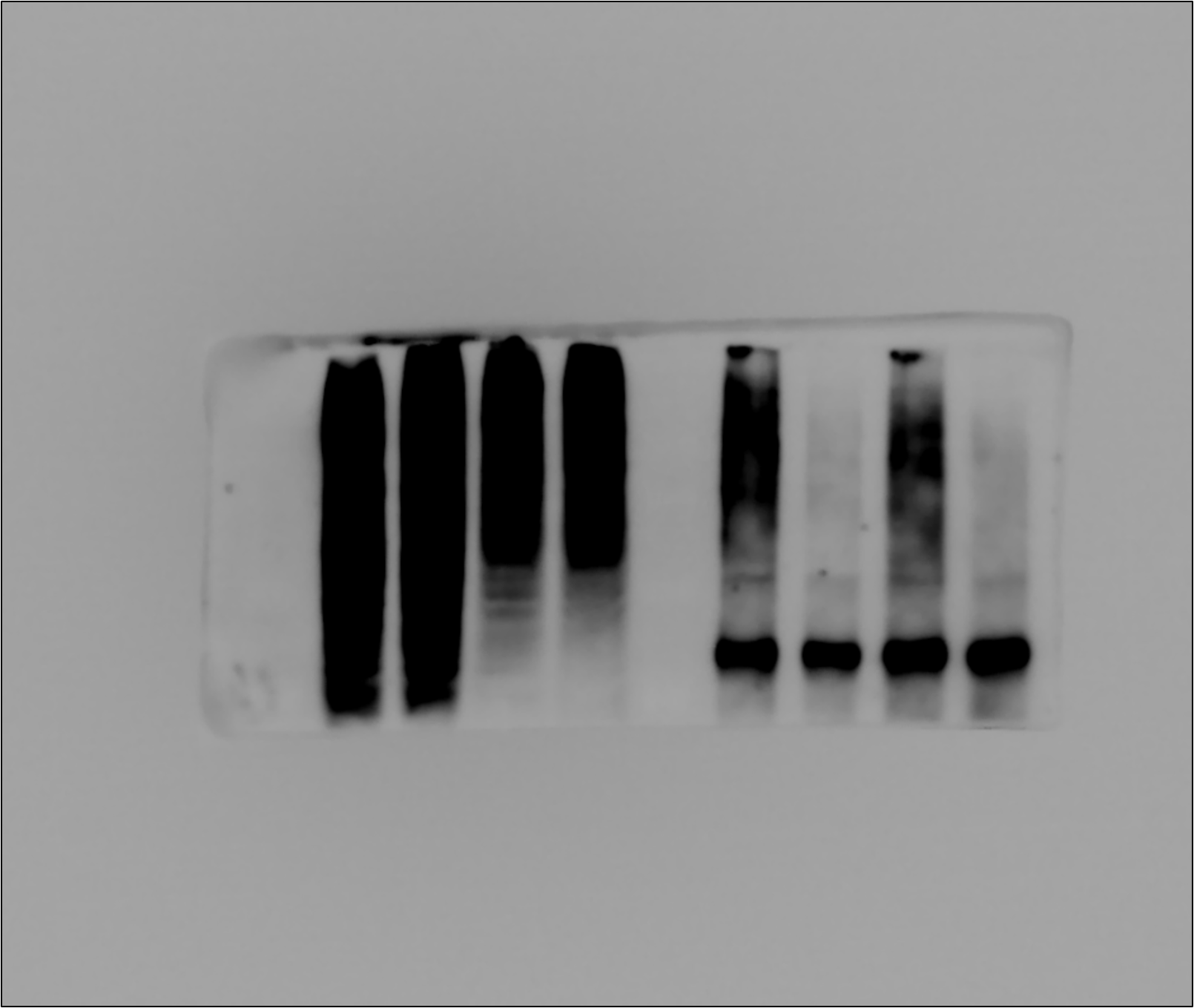

Supplement: Figure 5—source data 2. [file elife-98357-fig5-data2.zip › Figure 5-source data 2/5 H-WCL-TBK1-HA-Ub.tif]

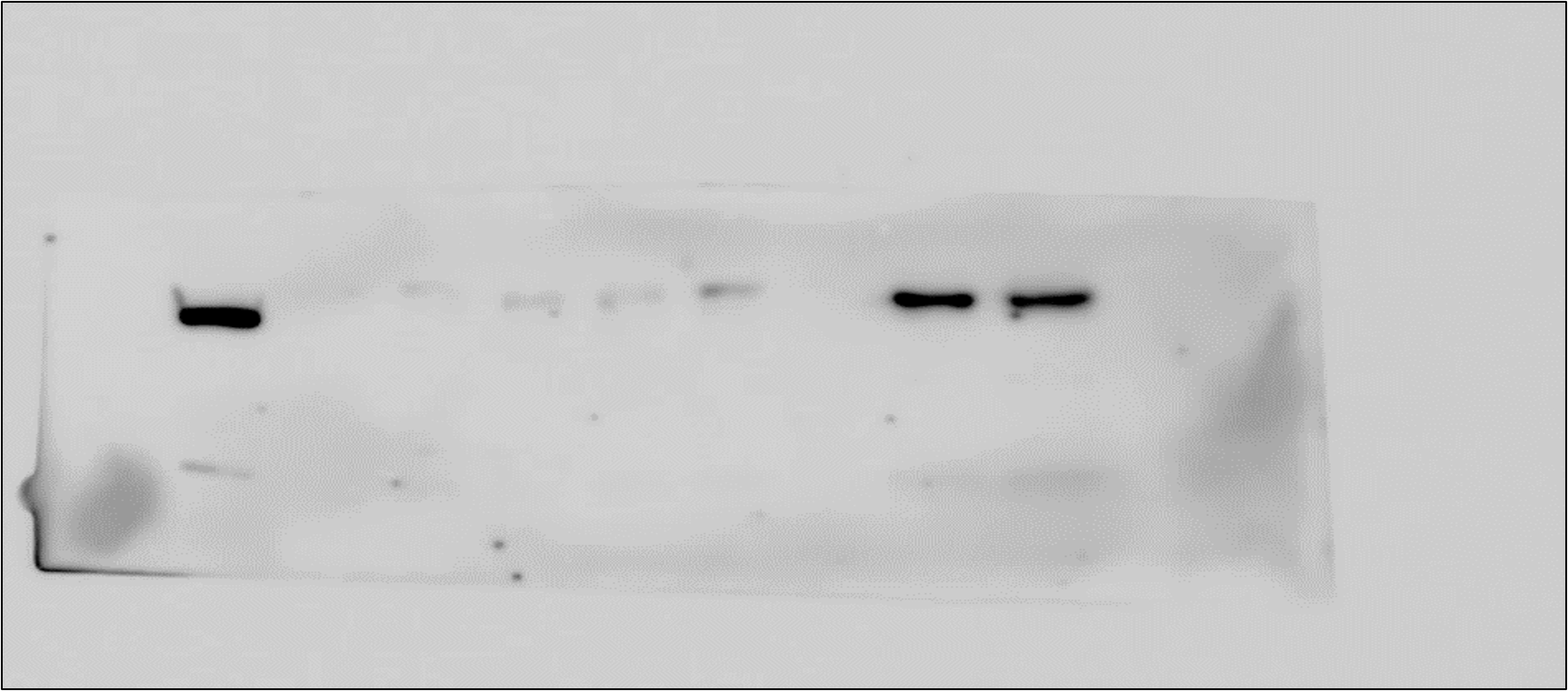

Supplement: Figure 6—source data 2. [file elife-98357-fig6-data2.zip › Figure 6-source data 2/6A-IP-Flag-1.tif]

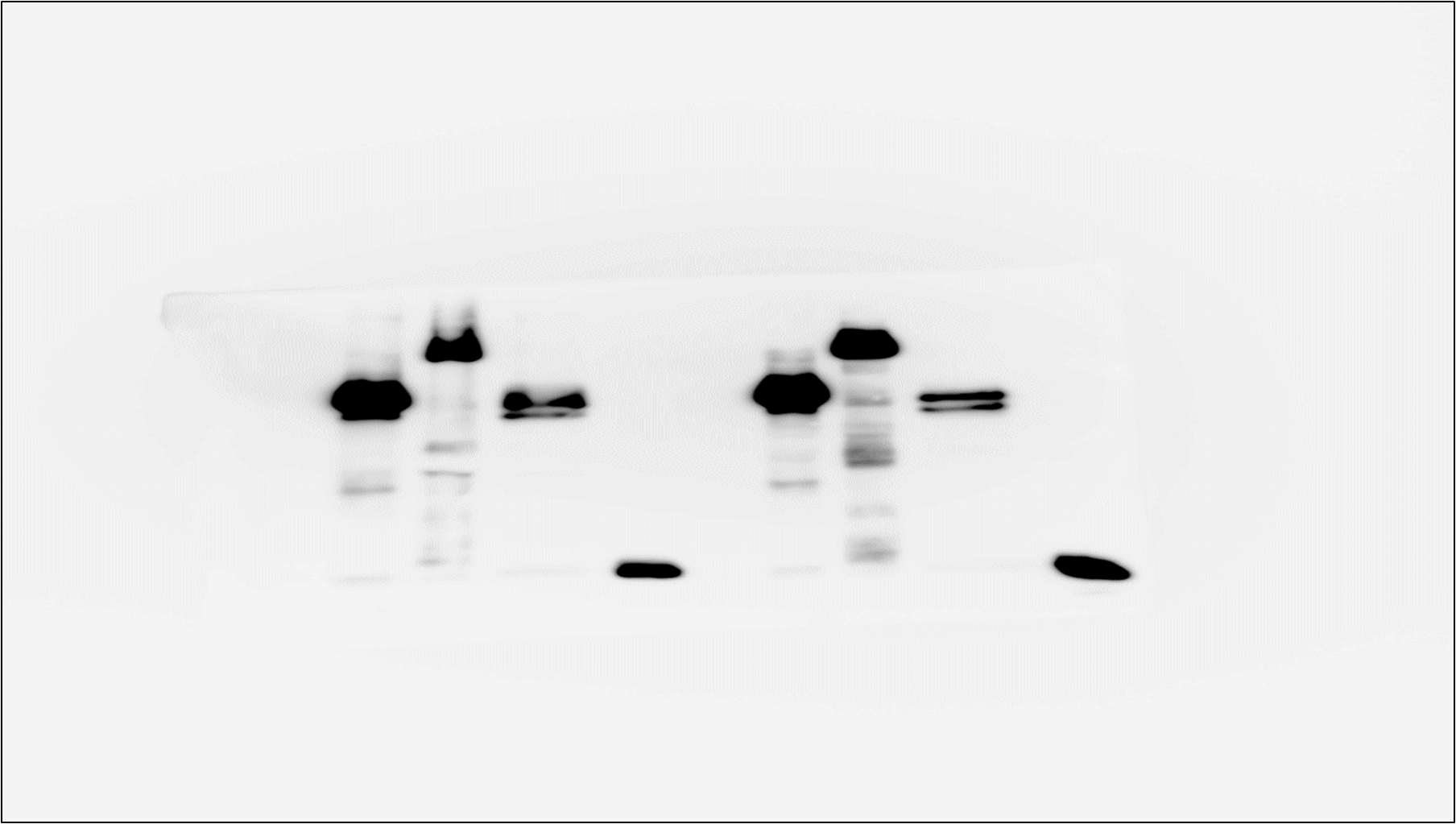

Supplement: Figure 6—source data 2. [file elife-98357-fig6-data2.zip › Figure 6-source data 2/6A-IP-Myc-1.tif]

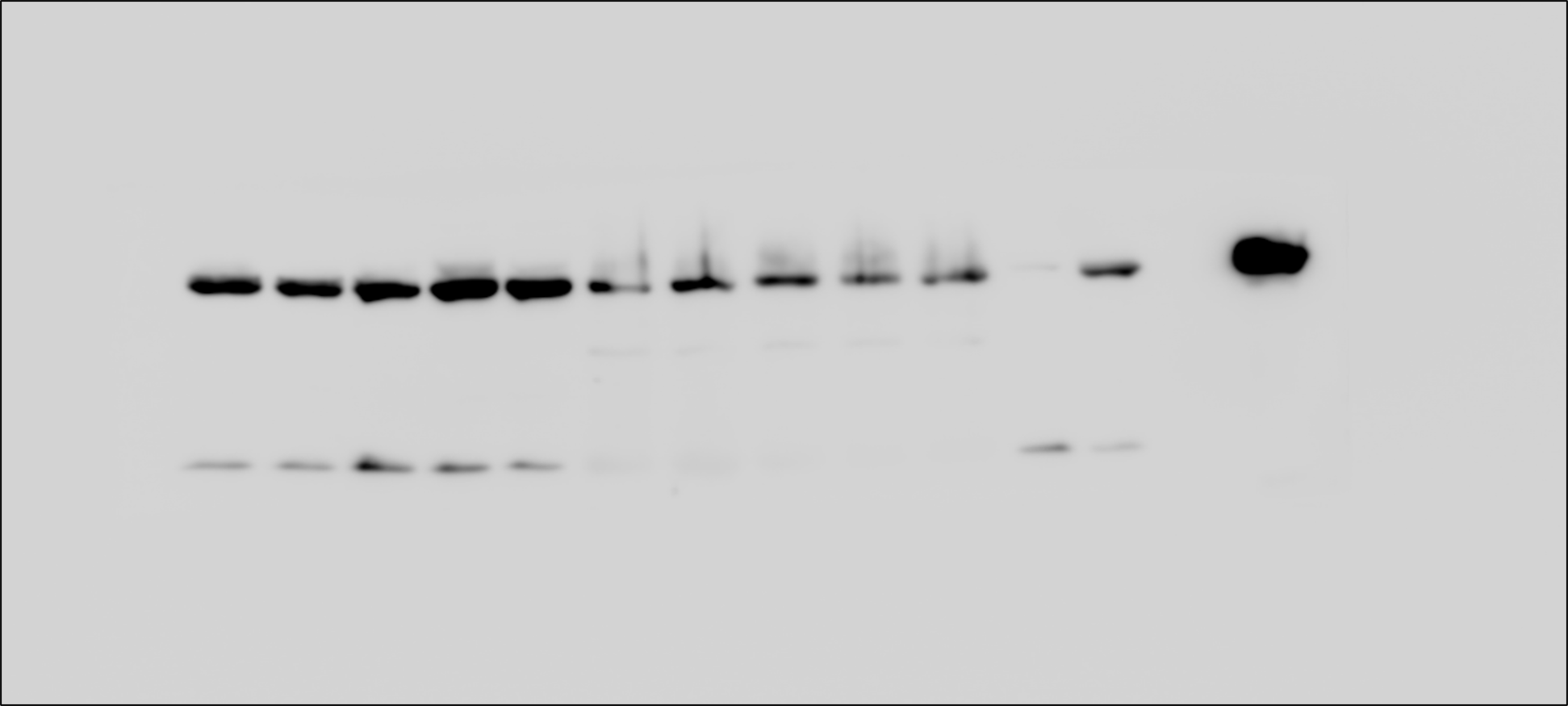

Supplement: Figure 6—source data 2. [file elife-98357-fig6-data2.zip › Figure 6-source data 2/6A-WCL-Flag-1.tif]

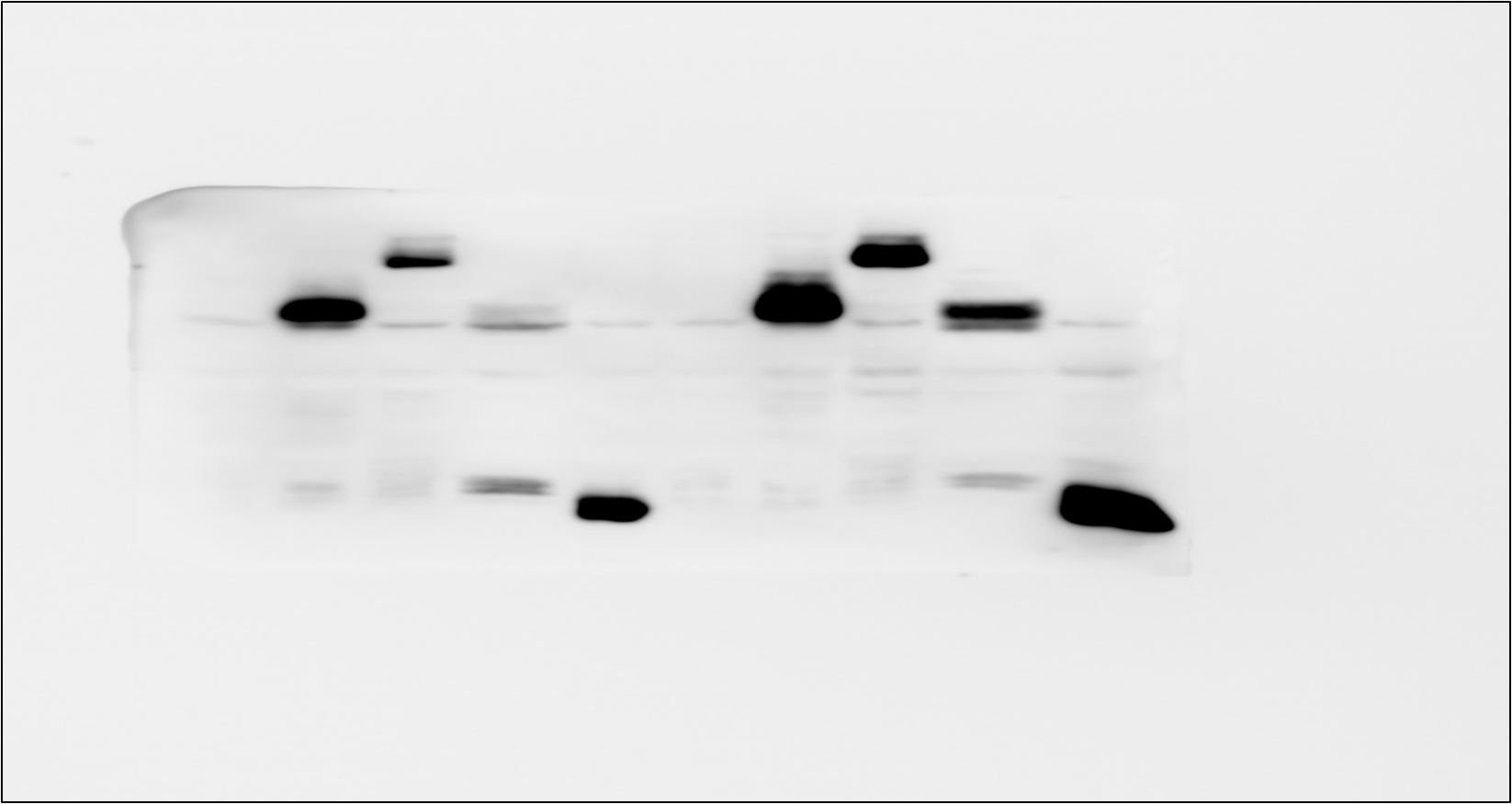

Supplement: Figure 6—source data 2. [file elife-98357-fig6-data2.zip › Figure 6-source data 2/6A-WCL-Myc-1.tif]

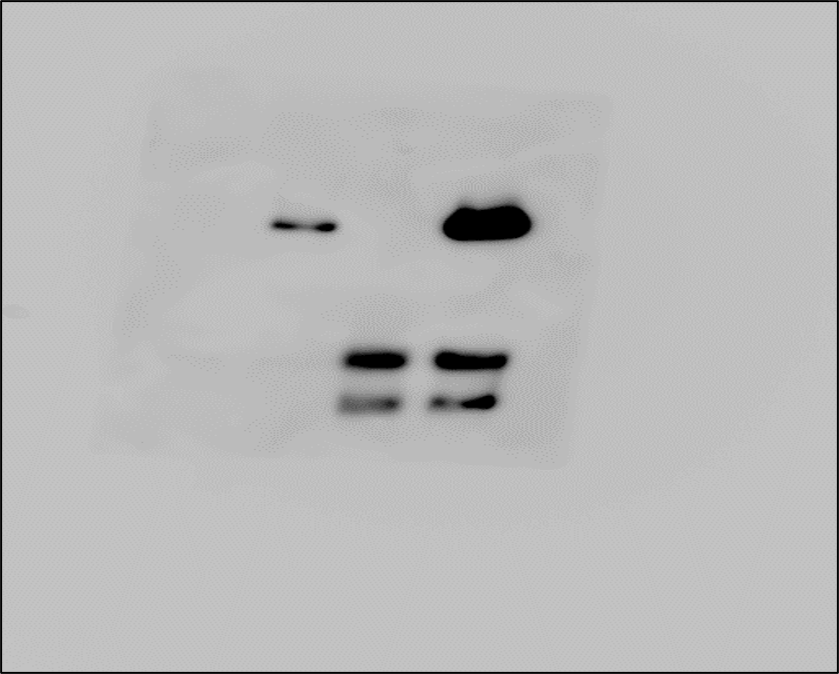

Supplement: Figure 6—source data 2. [file elife-98357-fig6-data2.zip › Figure 6-source data 2/6B-IP-Dtx4-Flag-1.tif]

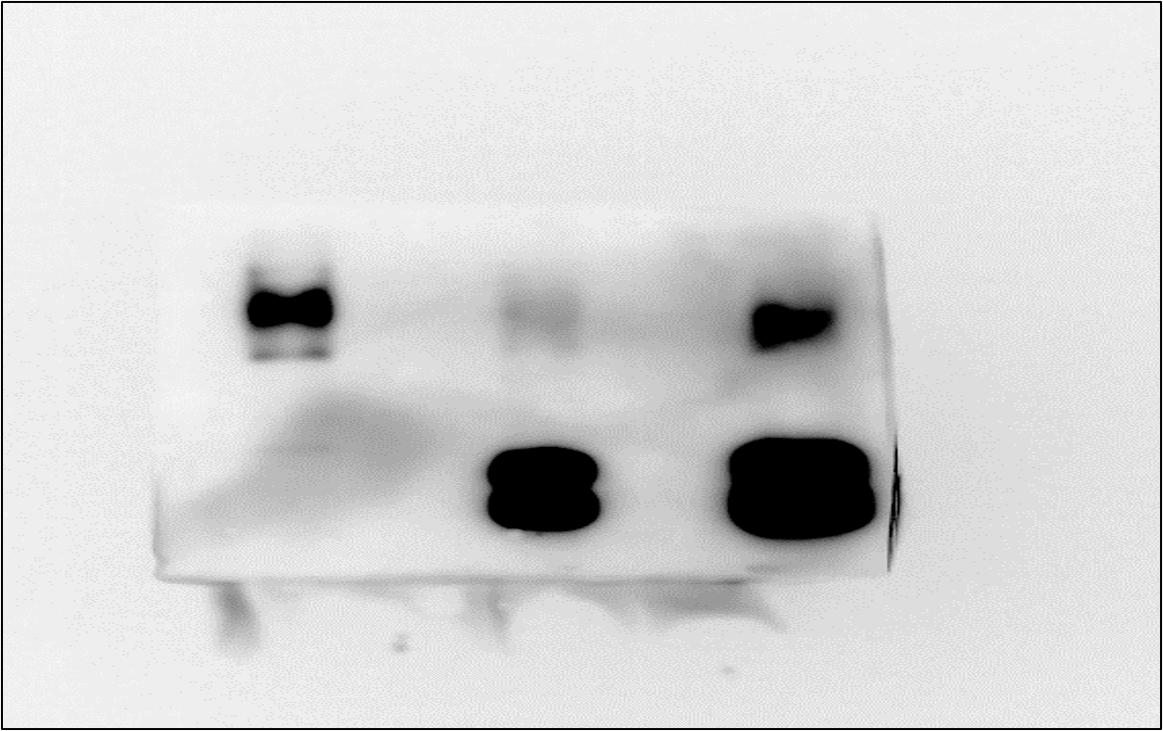

Supplement: Figure 6—source data 2. [file elife-98357-fig6-data2.zip › Figure 6-source data 2/6B-IP-Dtx4-HA-1.tif]

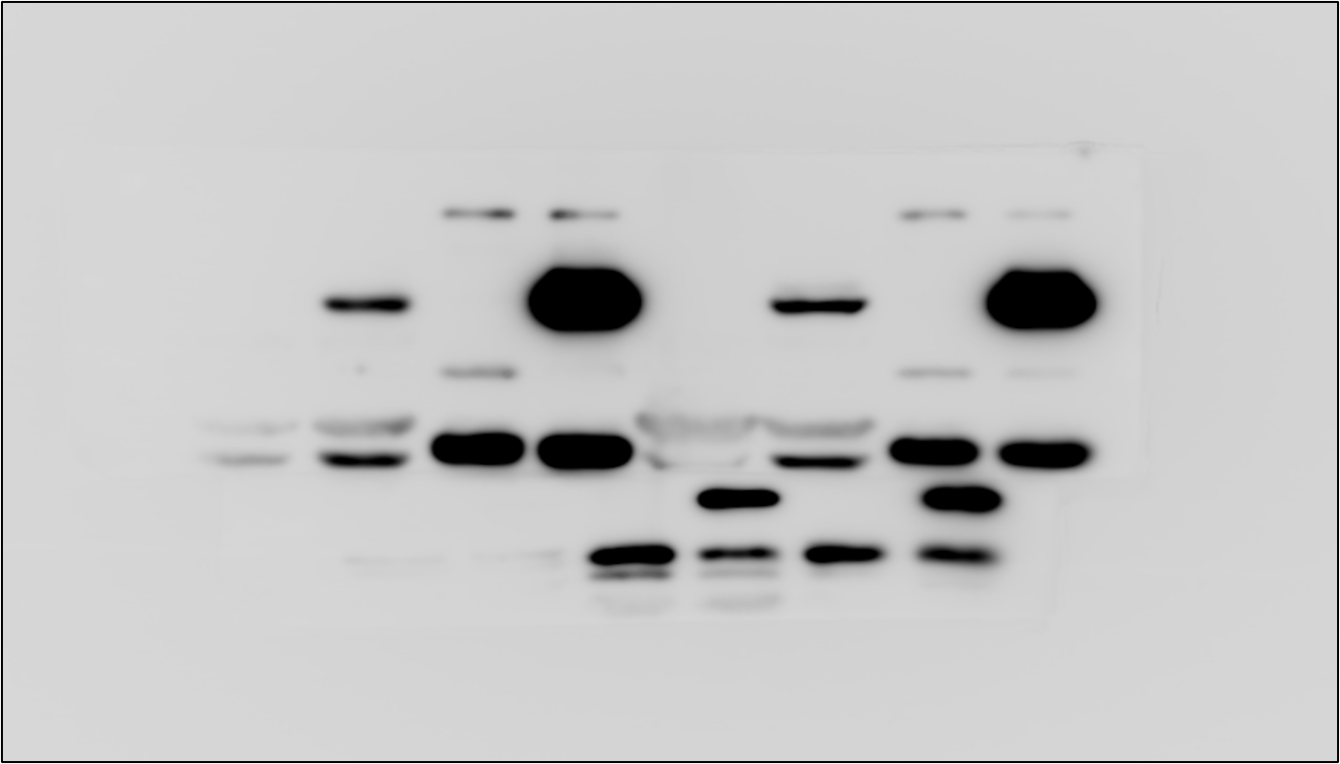

Supplement: Figure 6—source data 2. [file elife-98357-fig6-data2.zip › Figure 6-source data 2/6B-IP-Flag-1.tif]

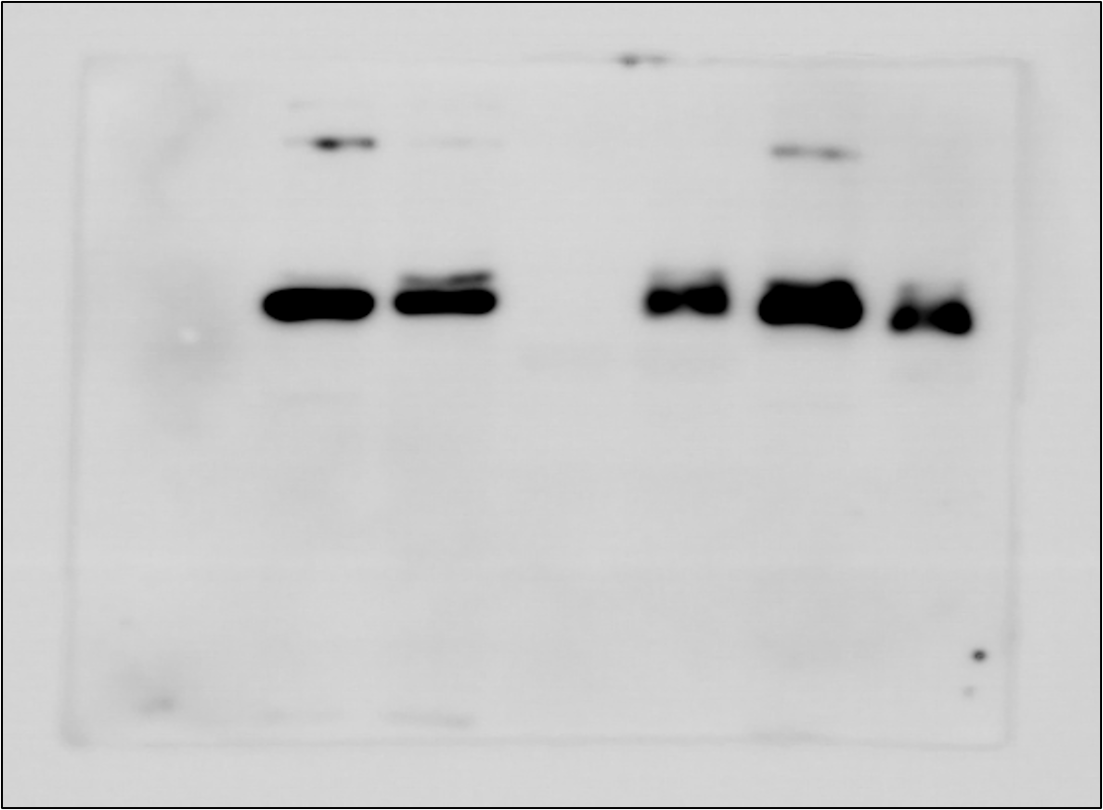

Supplement: Figure 6—source data 2. [file elife-98357-fig6-data2.zip › Figure 6-source data 2/6B-IP-Myc-1.tif]

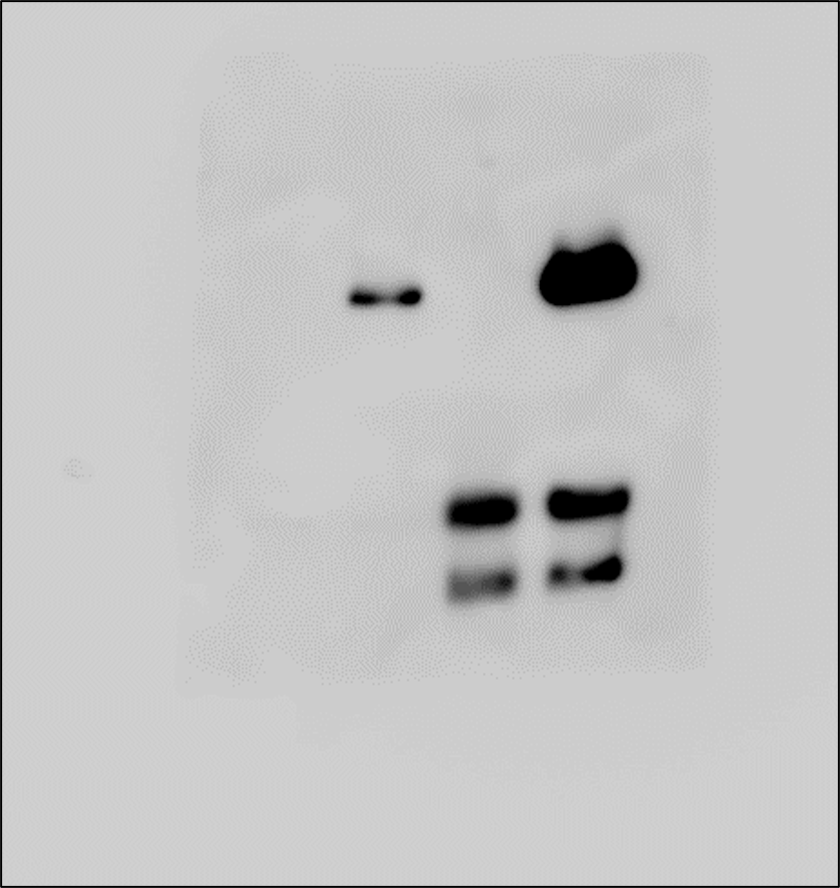

Supplement: Figure 6—source data 2. [file elife-98357-fig6-data2.zip › Figure 6-source data 2/6B-WCL-Dtx4-Flag-1.tif]

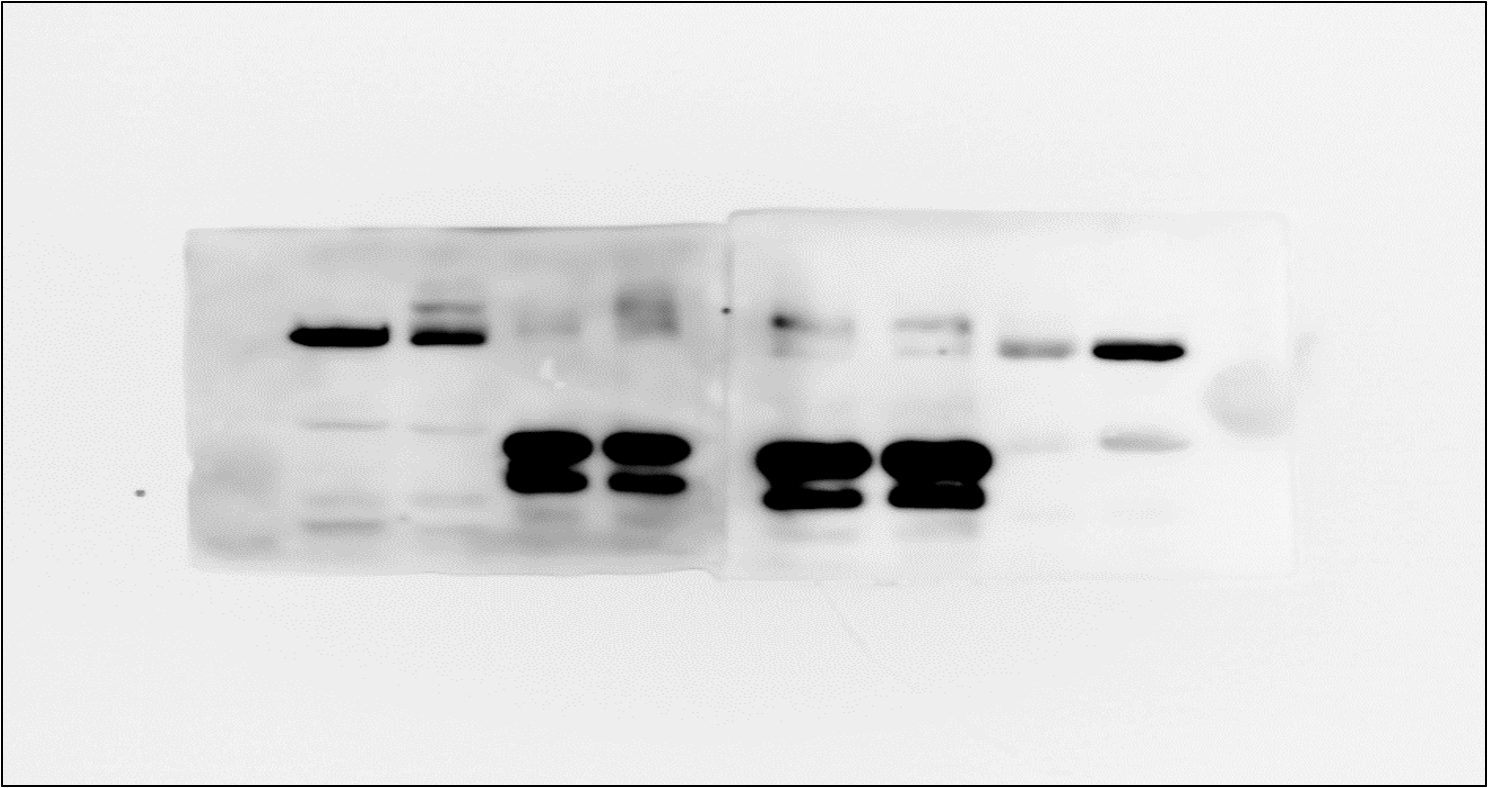

Supplement: Figure 6—source data 2. [file elife-98357-fig6-data2.zip › Figure 6-source data 2/6B-WCL-Dtx4-HA-1.tif]

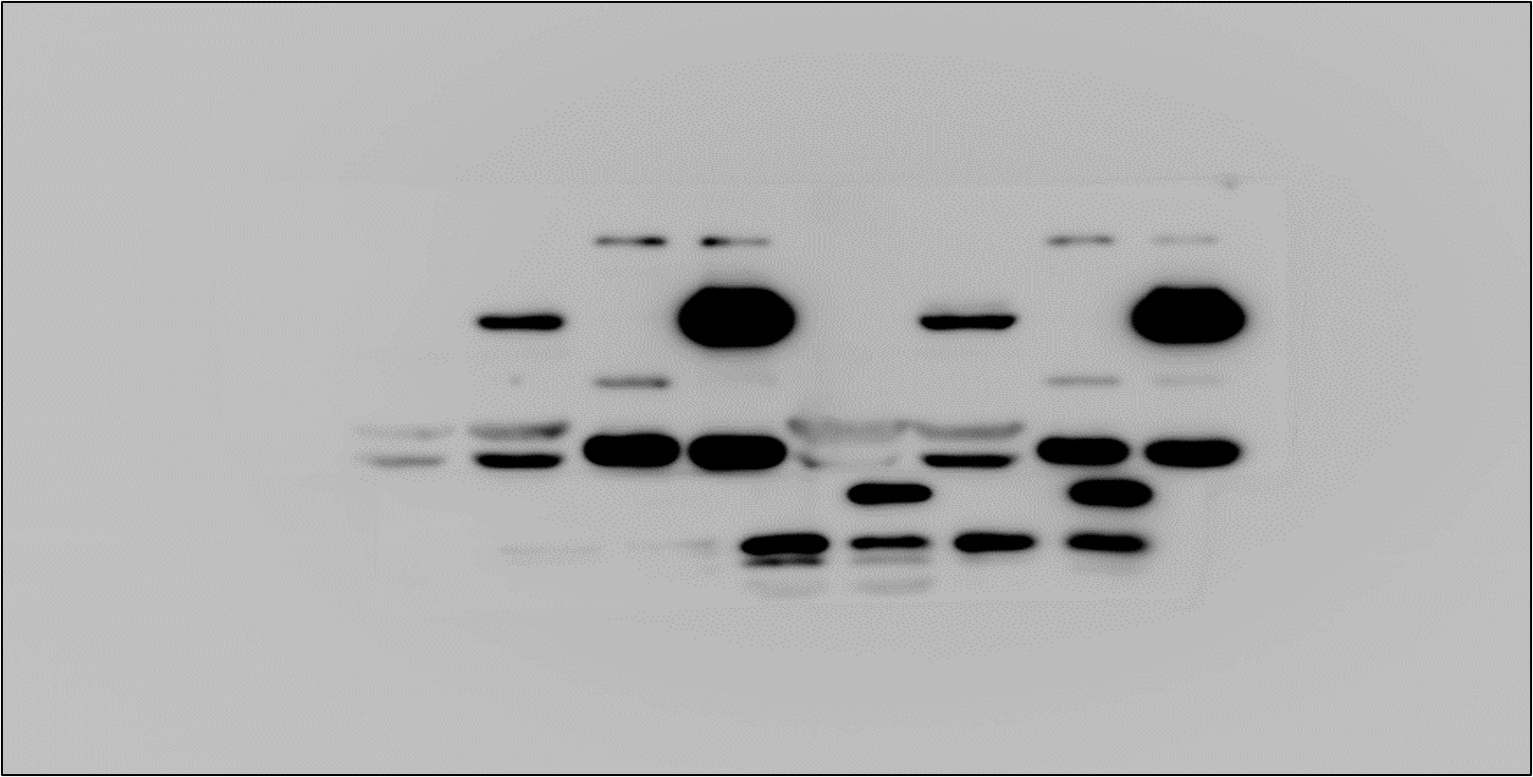

Supplement: Figure 6—source data 2. [file elife-98357-fig6-data2.zip › Figure 6-source data 2/6B-WCL-Flag-1.tif]

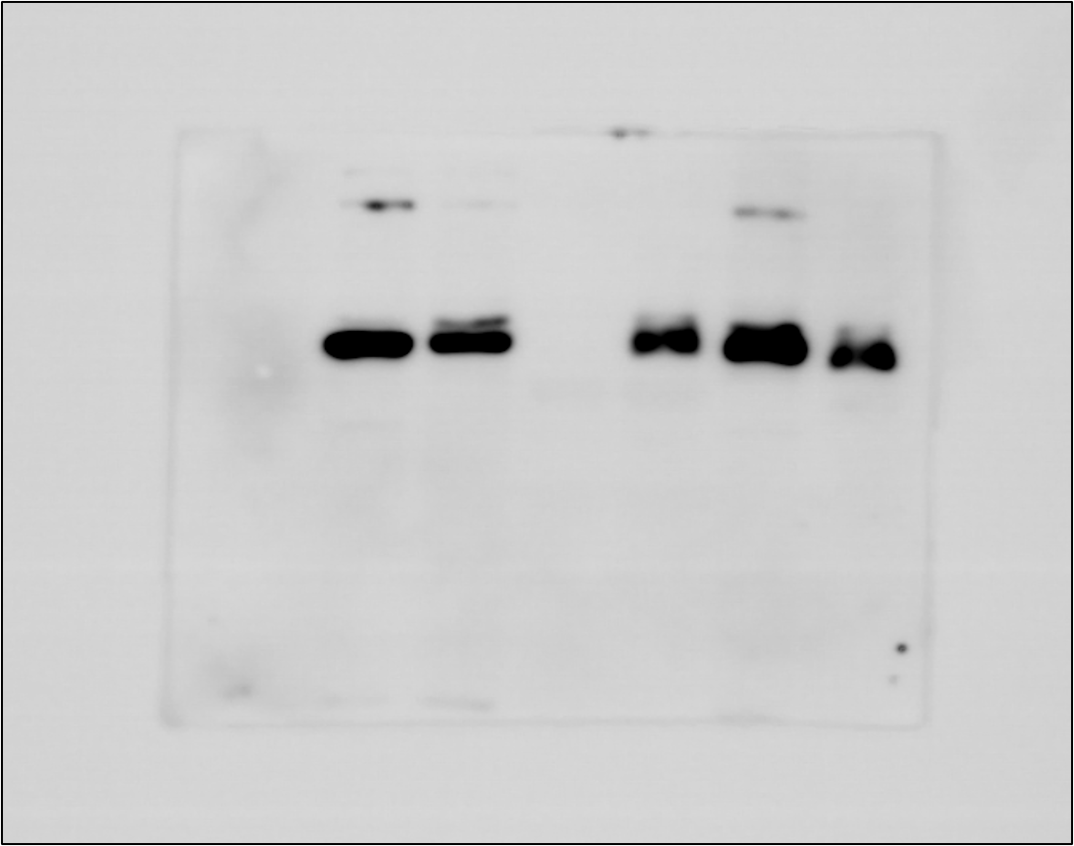

Supplement: Figure 6—source data 2. [file elife-98357-fig6-data2.zip › Figure 6-source data 2/6B-WCL-Myc-1.tif]

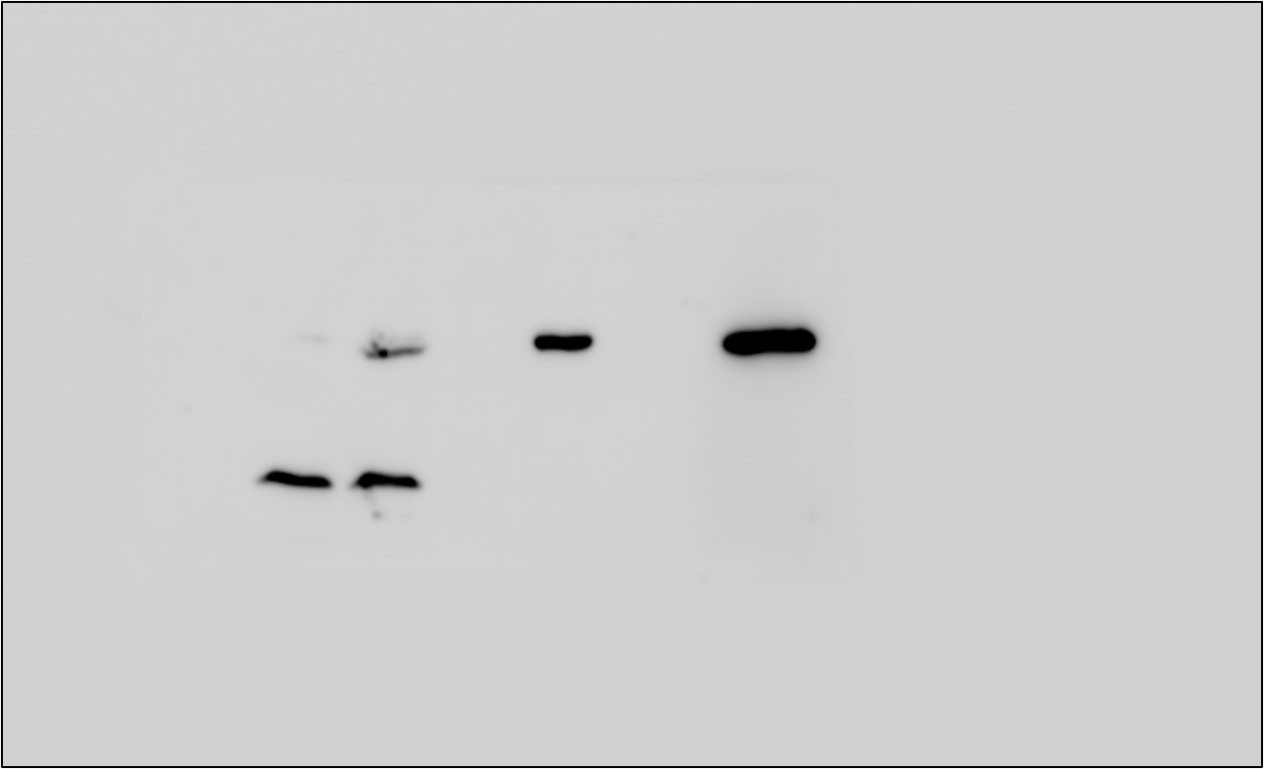

Supplement: Figure 6—source data 2. [file elife-98357-fig6-data2.zip › Figure 6-source data 2/6C-IP-Dtx4-Flag-1.tif]

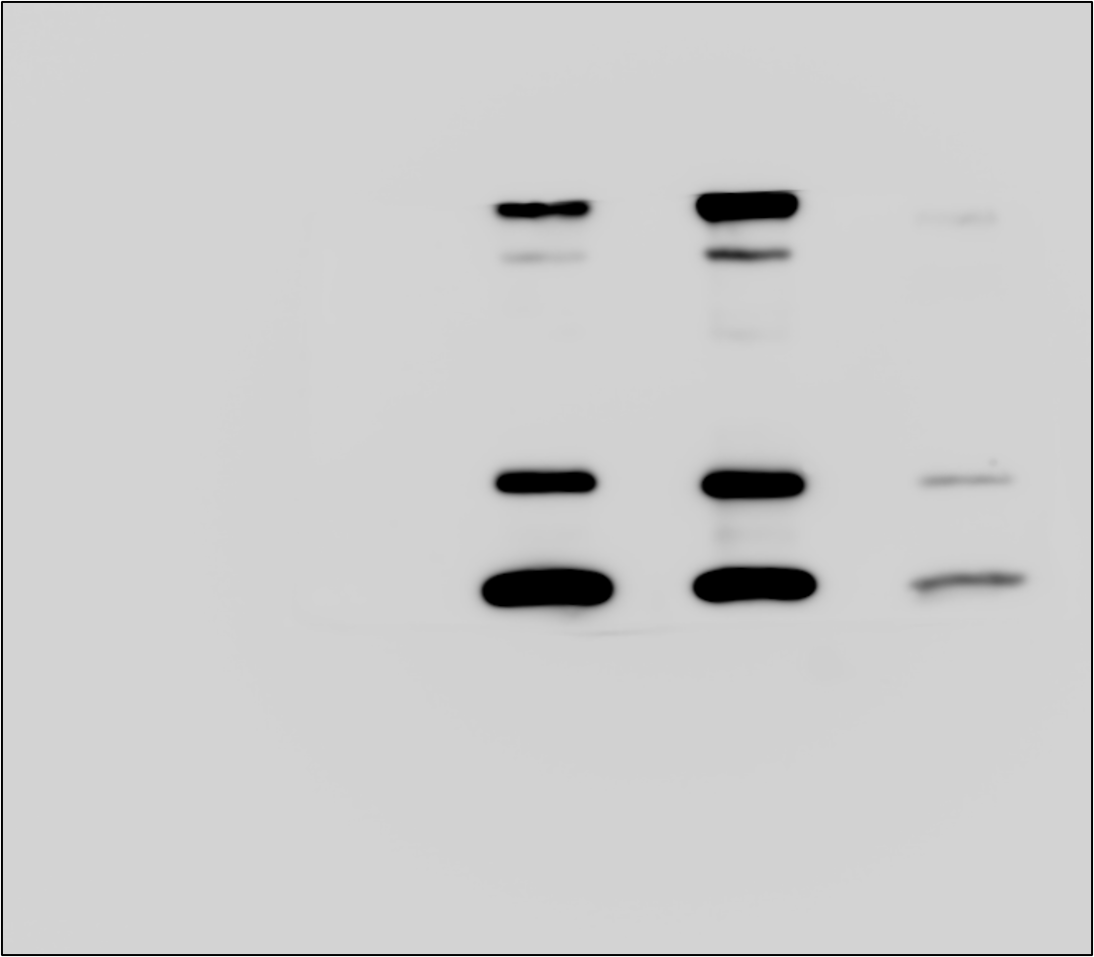

Supplement: Figure 6—source data 2. [file elife-98357-fig6-data2.zip › Figure 6-source data 2/6C-IP-Dtx4-Myc-1.tif]

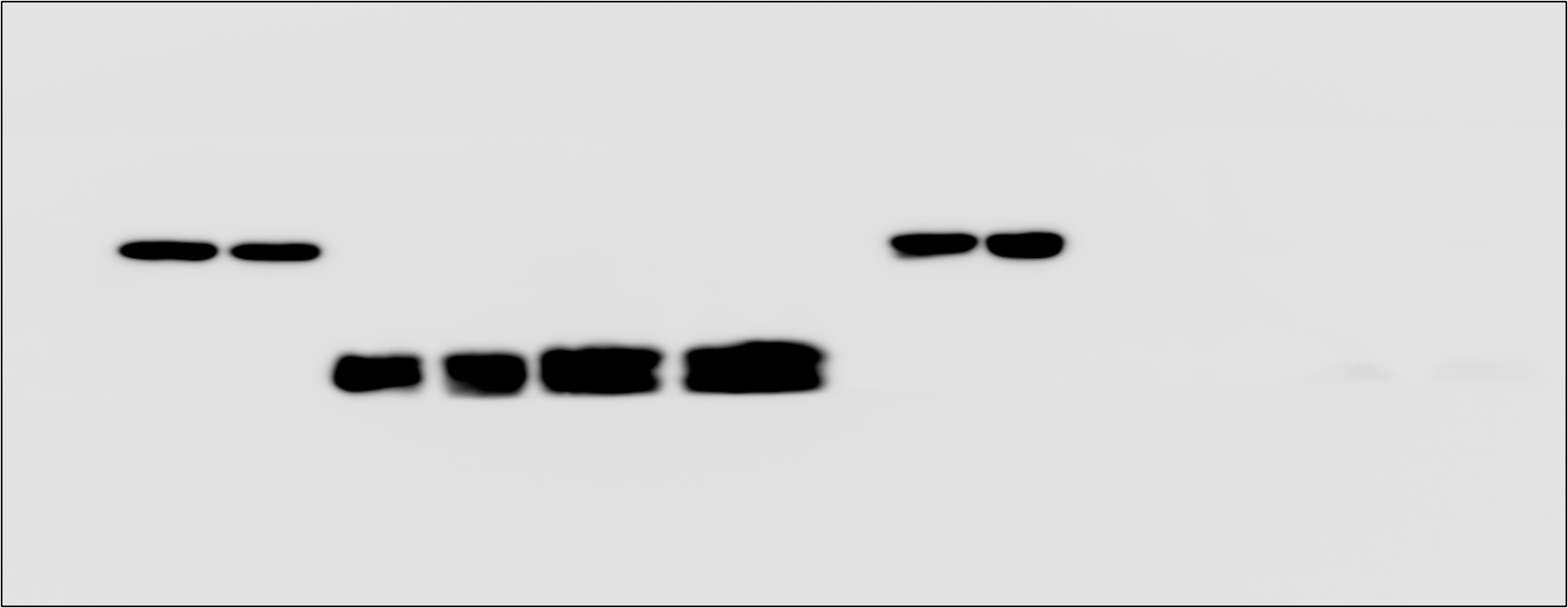

Supplement: Figure 6—source data 2. [file elife-98357-fig6-data2.zip › Figure 6-source data 2/6C-IP-TRIM11-HA-1.tif]

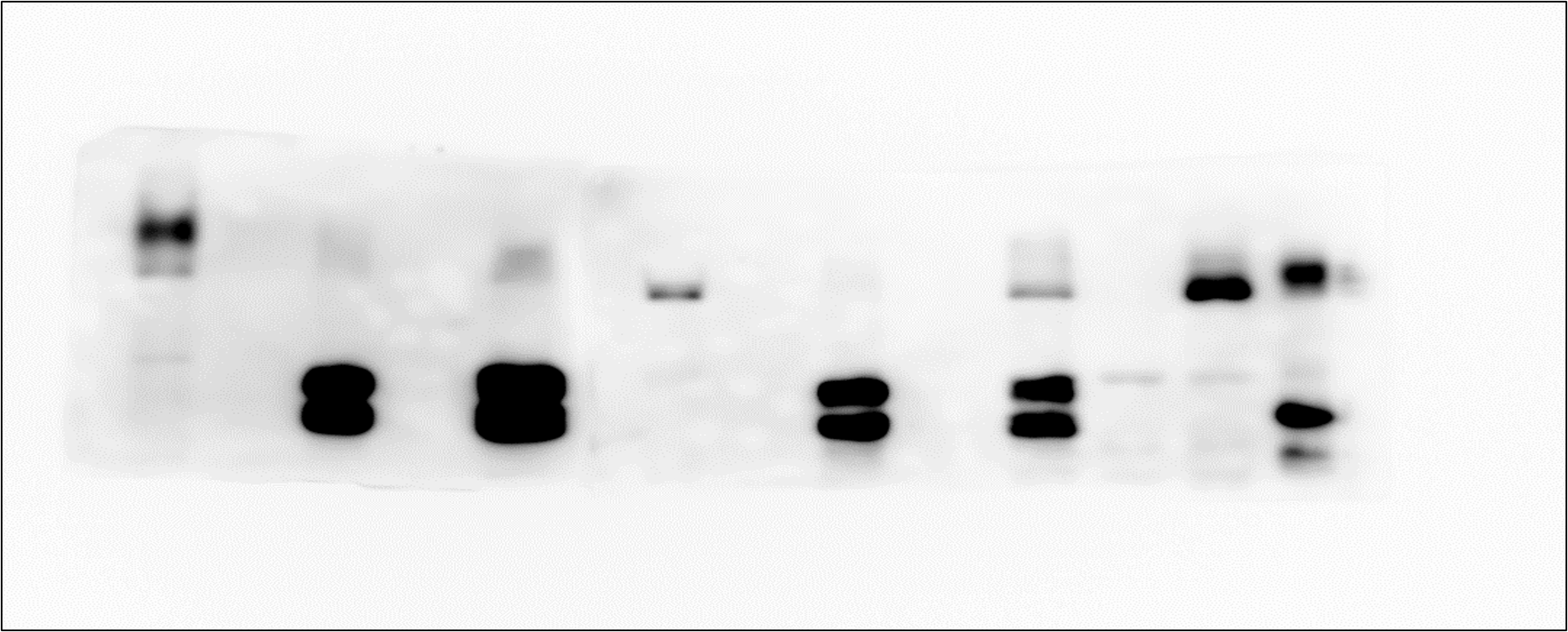

Supplement: Figure 6—source data 2. [file elife-98357-fig6-data2.zip › Figure 6-source data 2/6C-IP-TRIM11-Myc-1.tif]

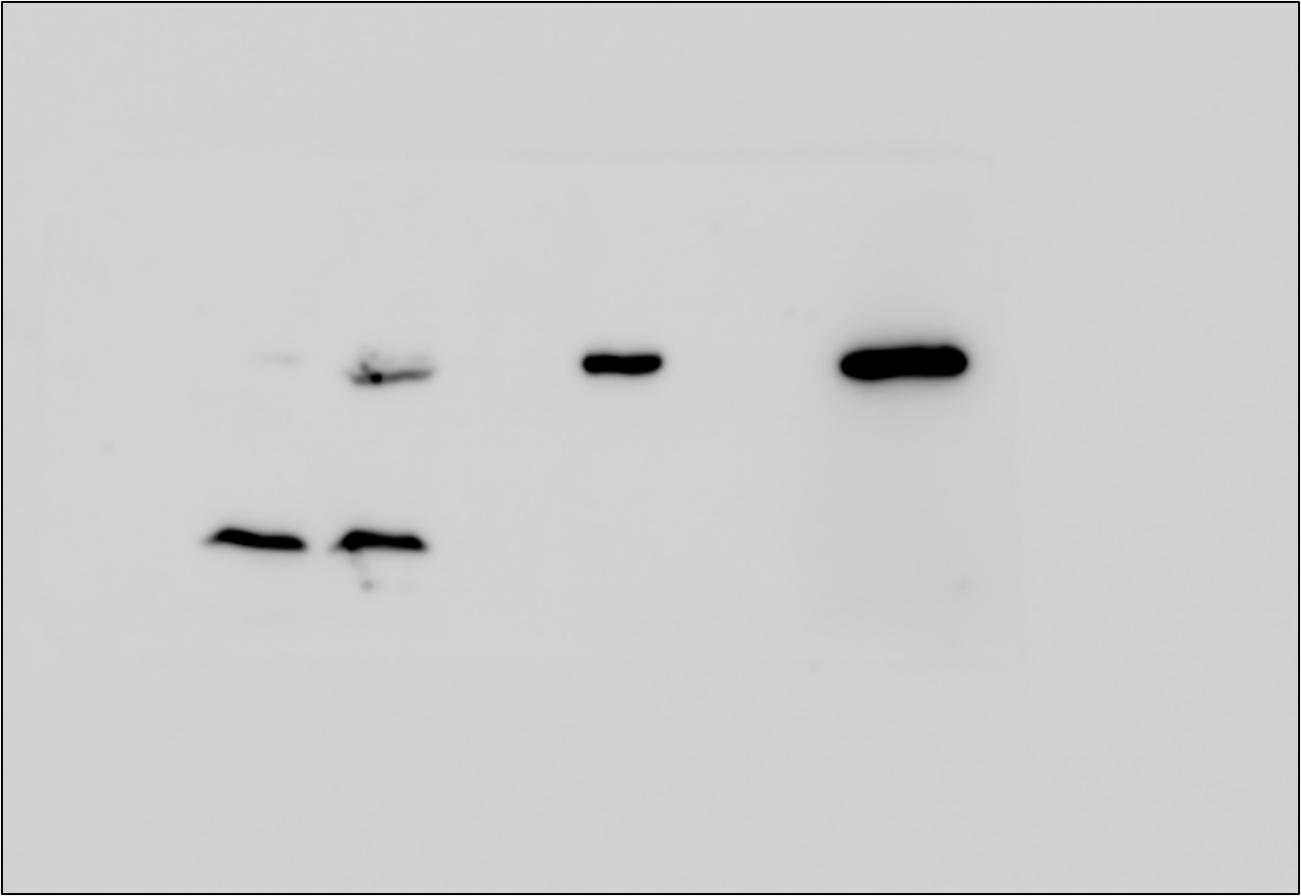

Supplement: Figure 6—source data 2. [file elife-98357-fig6-data2.zip › Figure 6-source data 2/6C-WCL-Dtx4-Flag-1.tif]

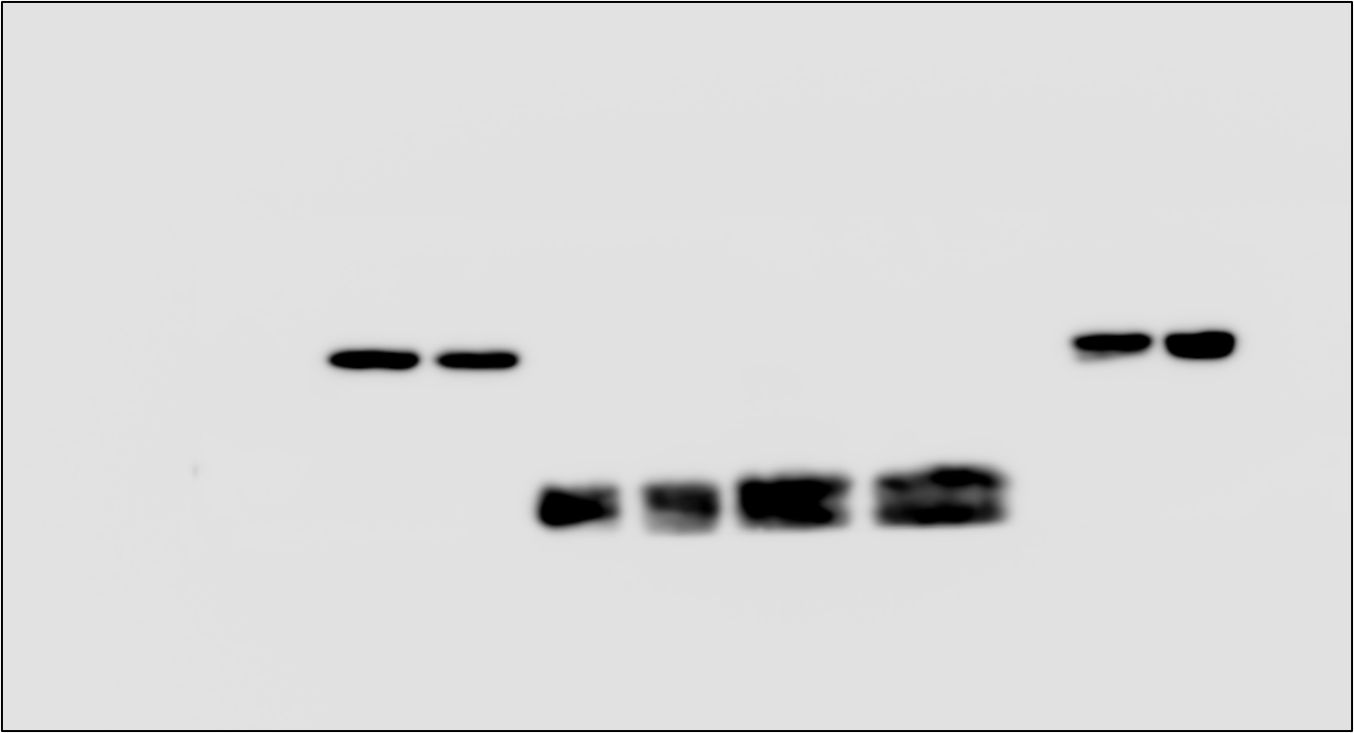

Supplement: Figure 6—source data 2. [file elife-98357-fig6-data2.zip › Figure 6-source data 2/6C-WCL-Dtx4-Myc-1.tif]

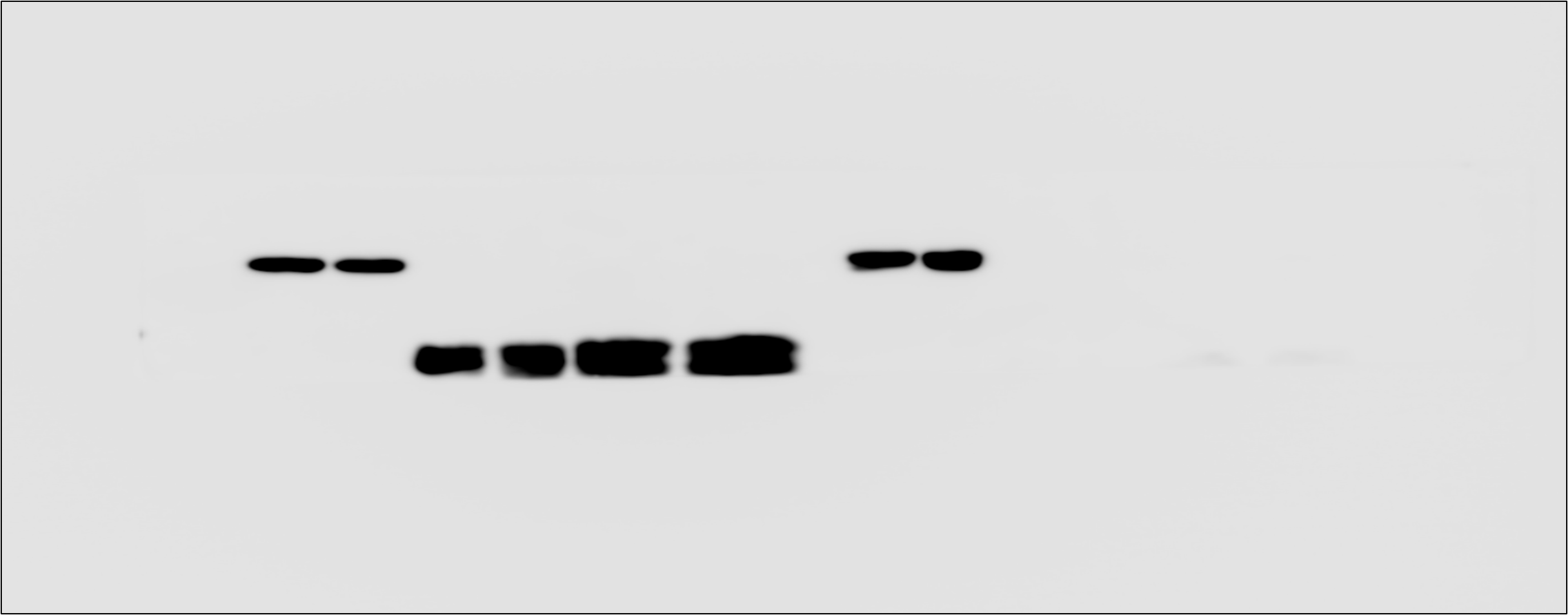

Supplement: Figure 6—source data 2. [file elife-98357-fig6-data2.zip › Figure 6-source data 2/6C-WCL-TRIM11-HA-1.tif]

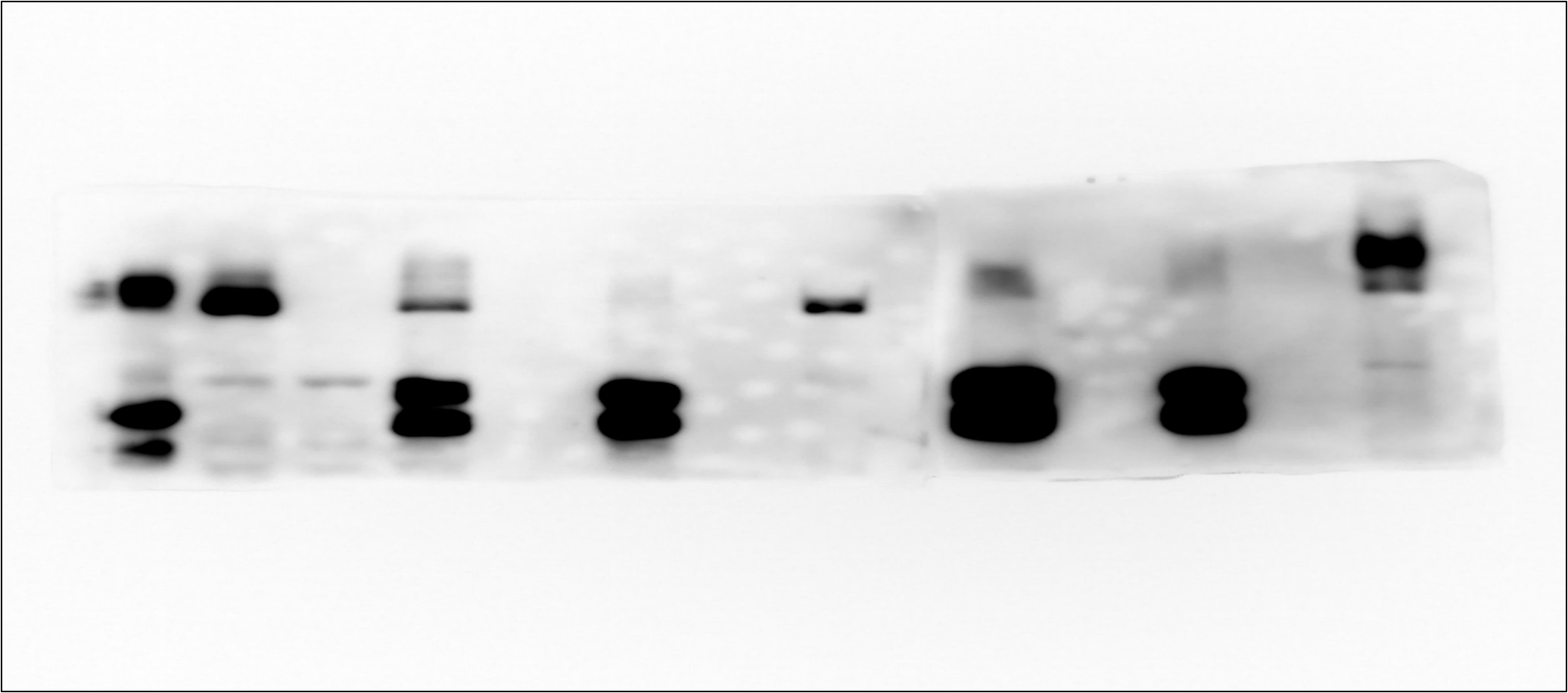

Supplement: Figure 6—source data 2. [file elife-98357-fig6-data2.zip › Figure 6-source data 2/6C-WCL-TRIM11-Myc-1.tif]

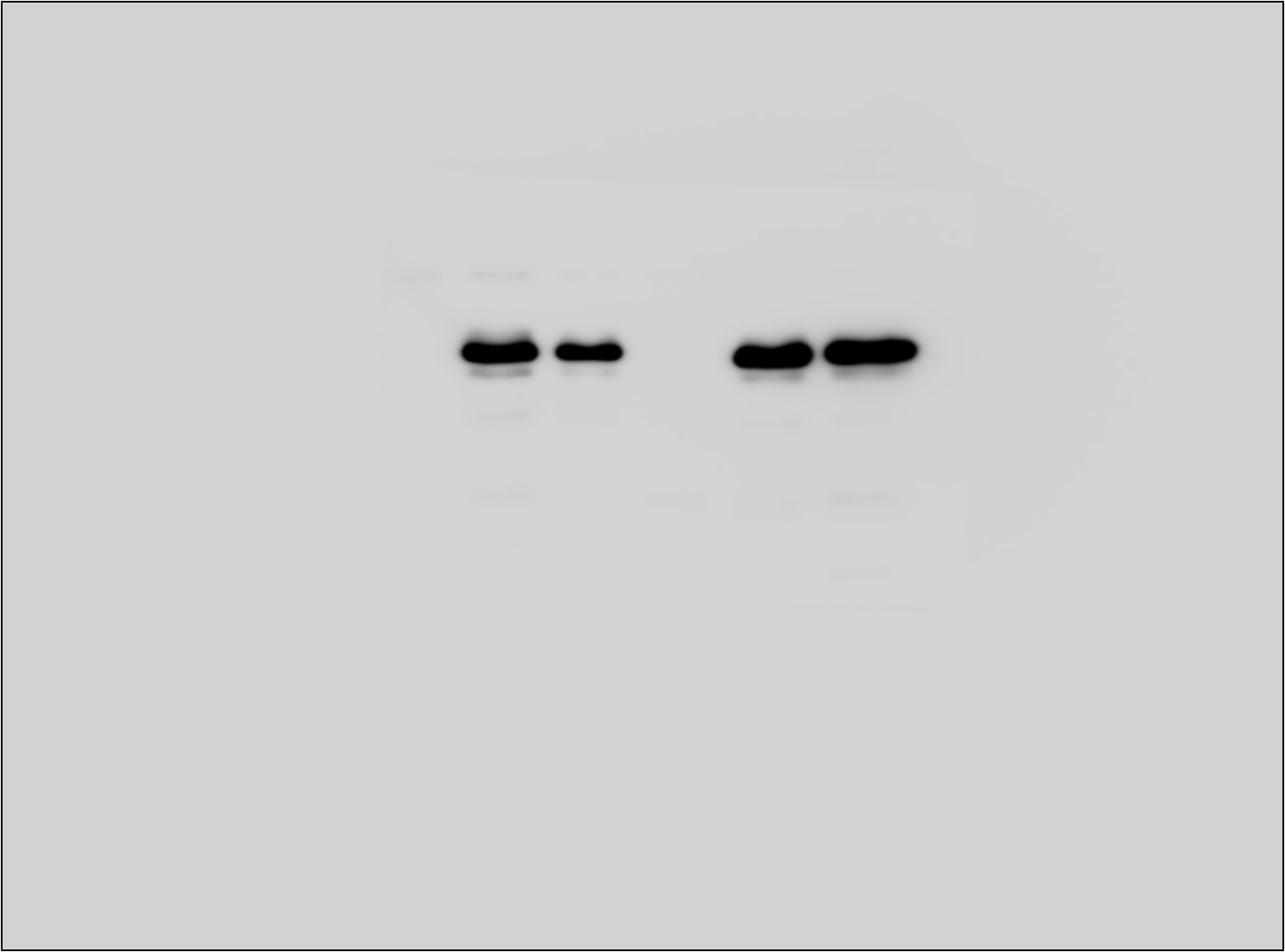

Supplement: Figure 6—source data 2. [file elife-98357-fig6-data2.zip › Figure 6-source data 2/6D-IP-CDK2-Flag-1.tif]

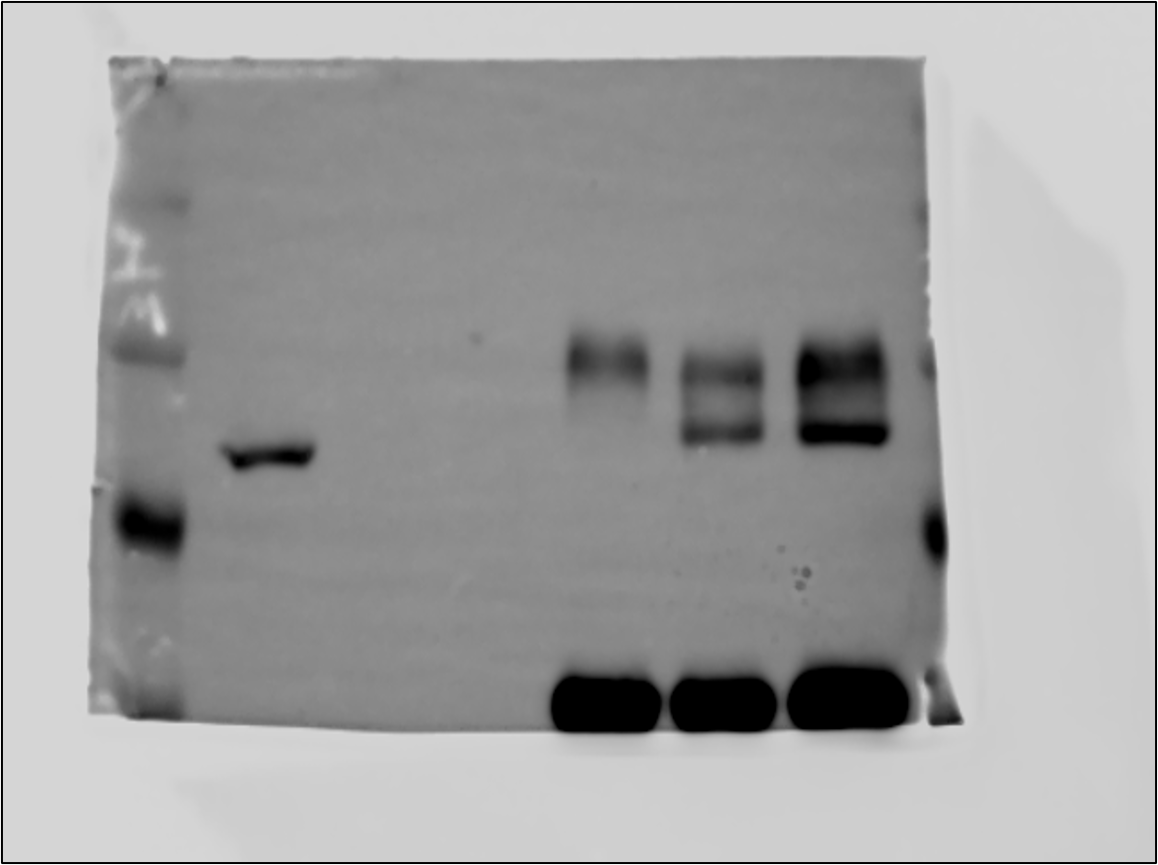

Supplement: Figure 6—source data 2. [file elife-98357-fig6-data2.zip › Figure 6-source data 2/6D-IP-CDK2-HA-1.tif]

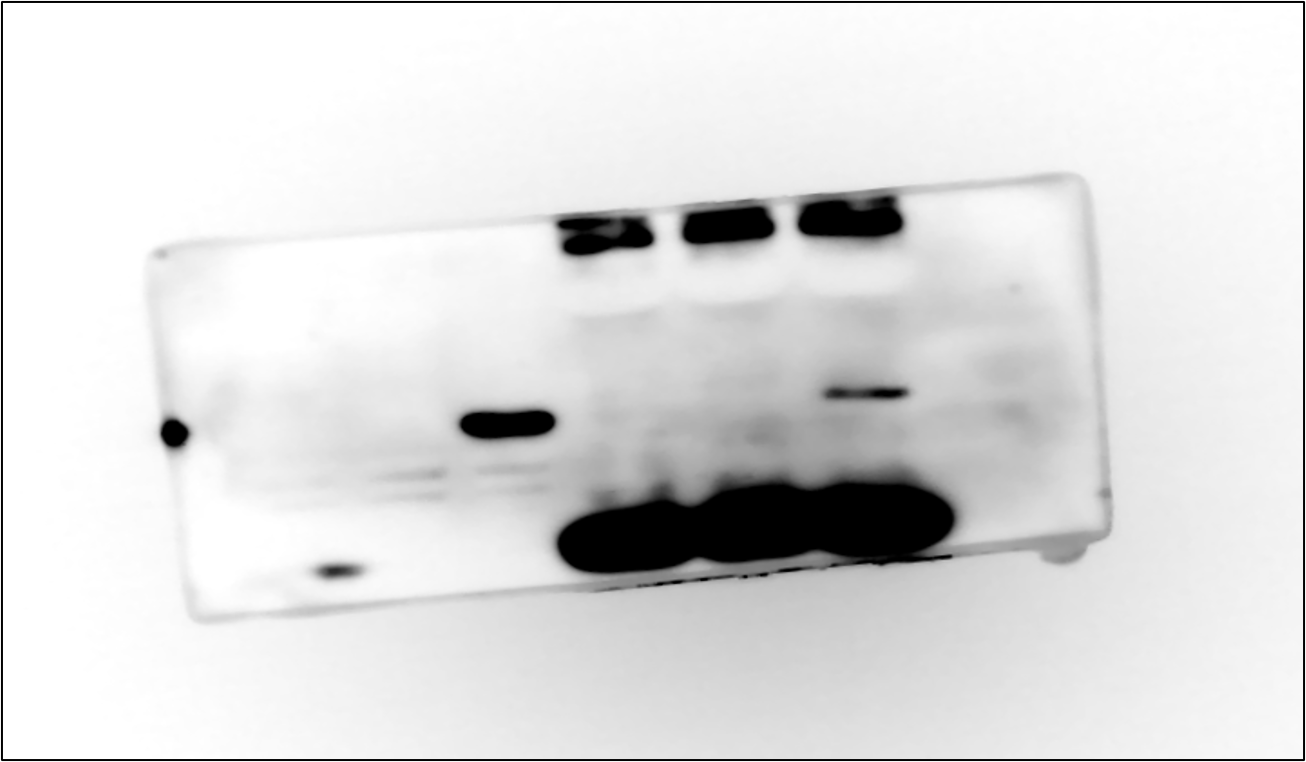

Supplement: Figure 6—source data 2. [file elife-98357-fig6-data2.zip › Figure 6-source data 2/6D-IP-CDK2-Myc-1.tif]

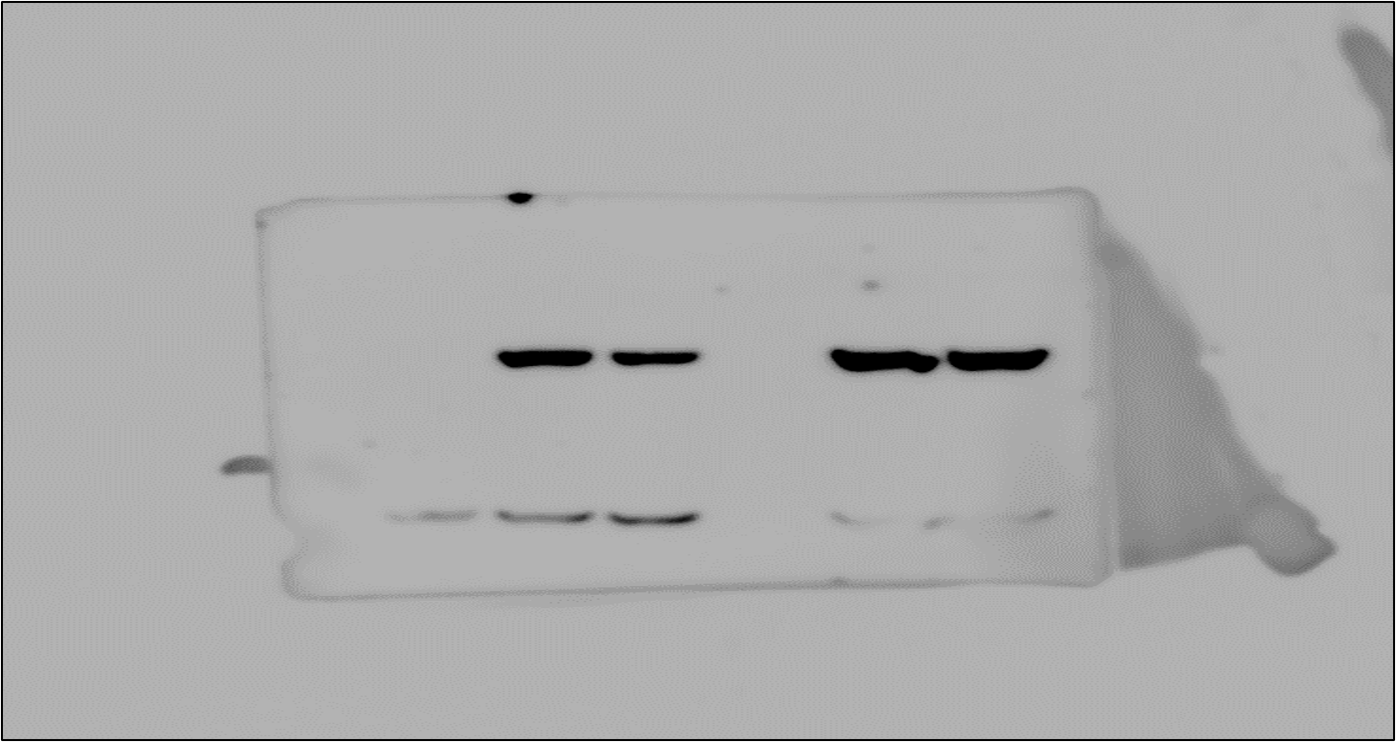

Supplement: Figure 6—source data 2. [file elife-98357-fig6-data2.zip › Figure 6-source data 2/6D-IP-shCDK2-Flag-1.tif]

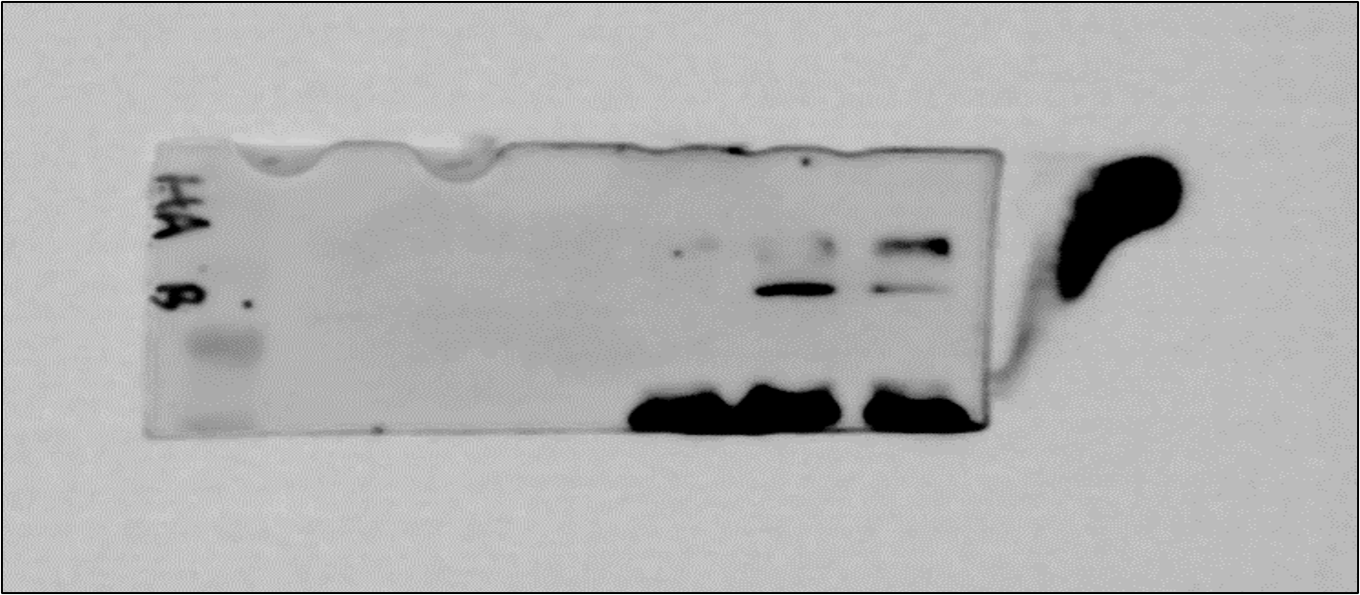

Supplement: Figure 6—source data 2. [file elife-98357-fig6-data2.zip › Figure 6-source data 2/6D-IP-shCDK2-HA-1.tif]

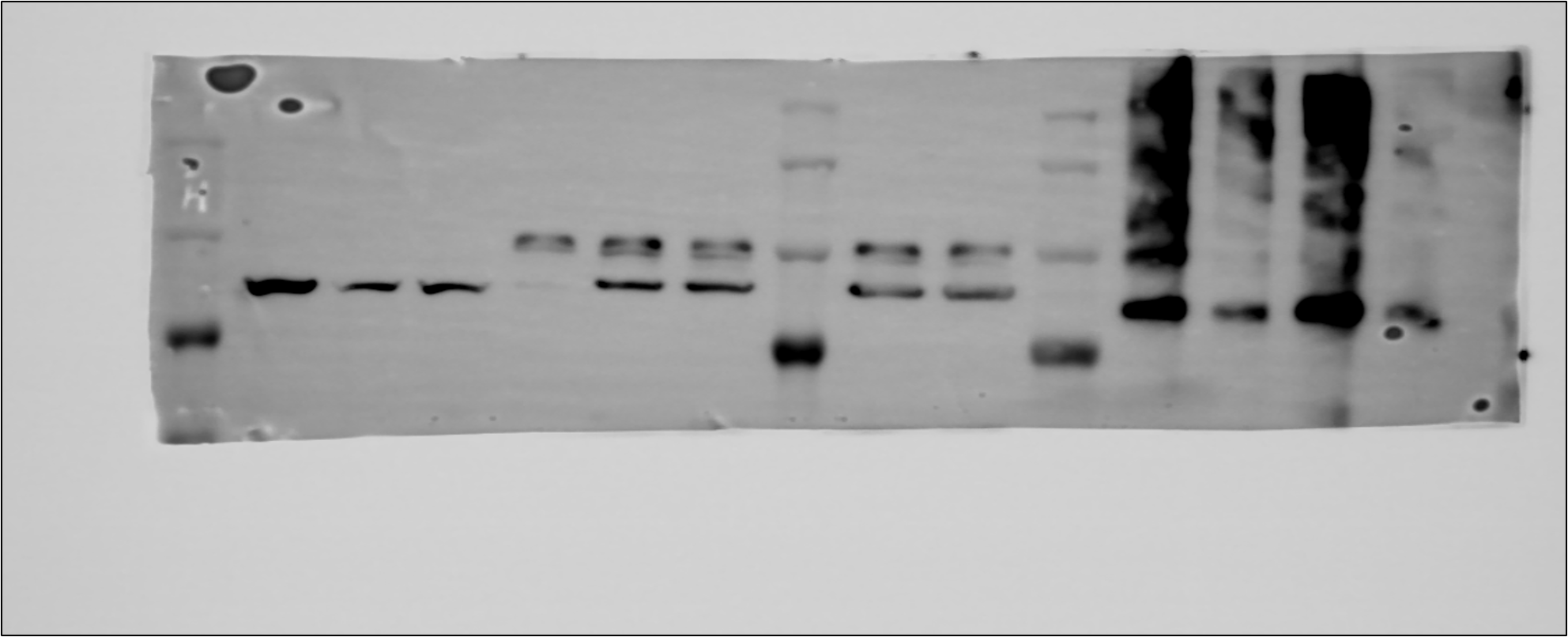

Supplement: Figure 6—source data 2. [file elife-98357-fig6-data2.zip › Figure 6-source data 2/6D-WCL-CDK2-HA-1.tif]

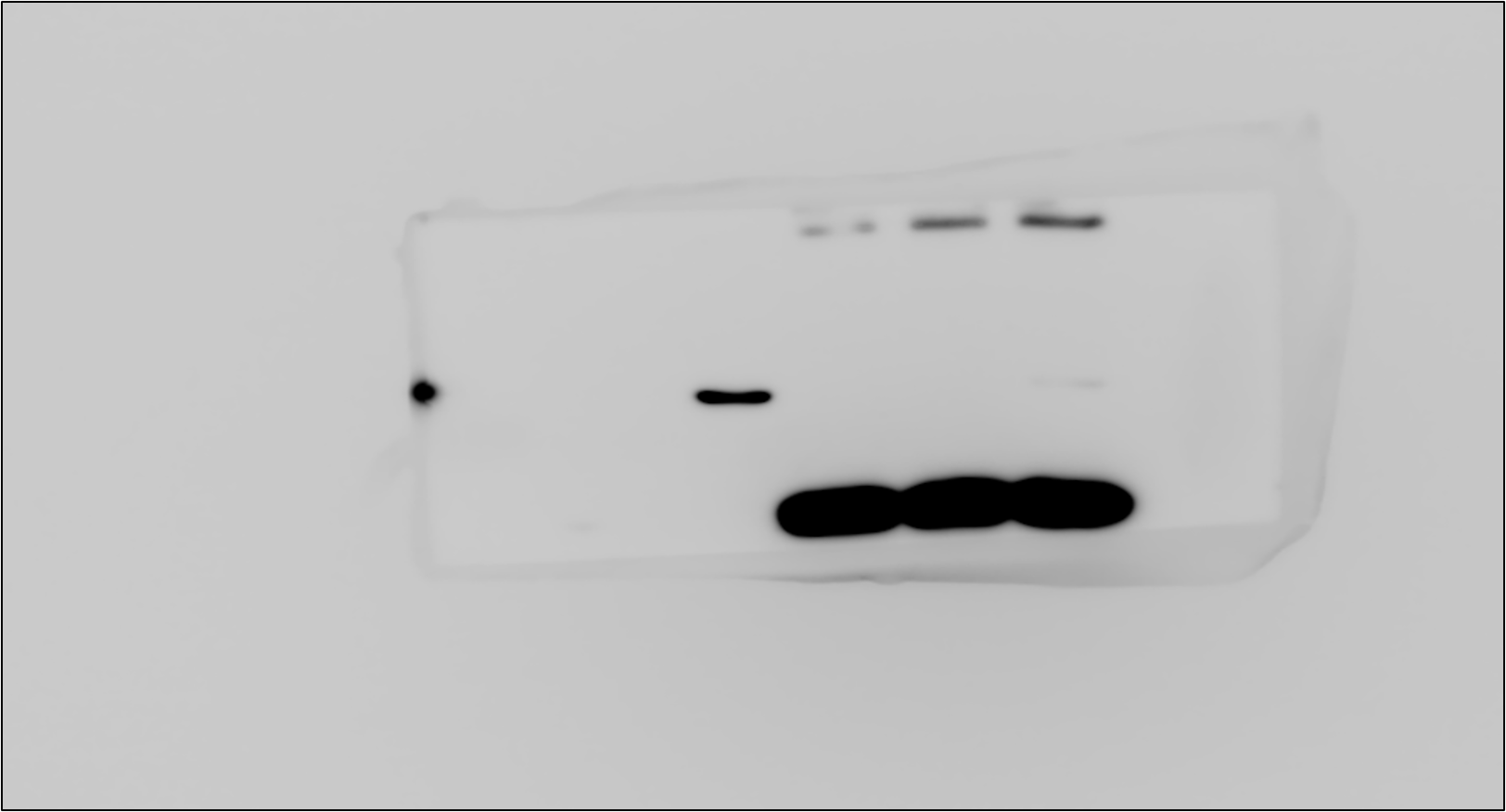

Supplement: Figure 6—source data 2. [file elife-98357-fig6-data2.zip › Figure 6-source data 2/6D-WCL-CDK2-Myc-1.tif]

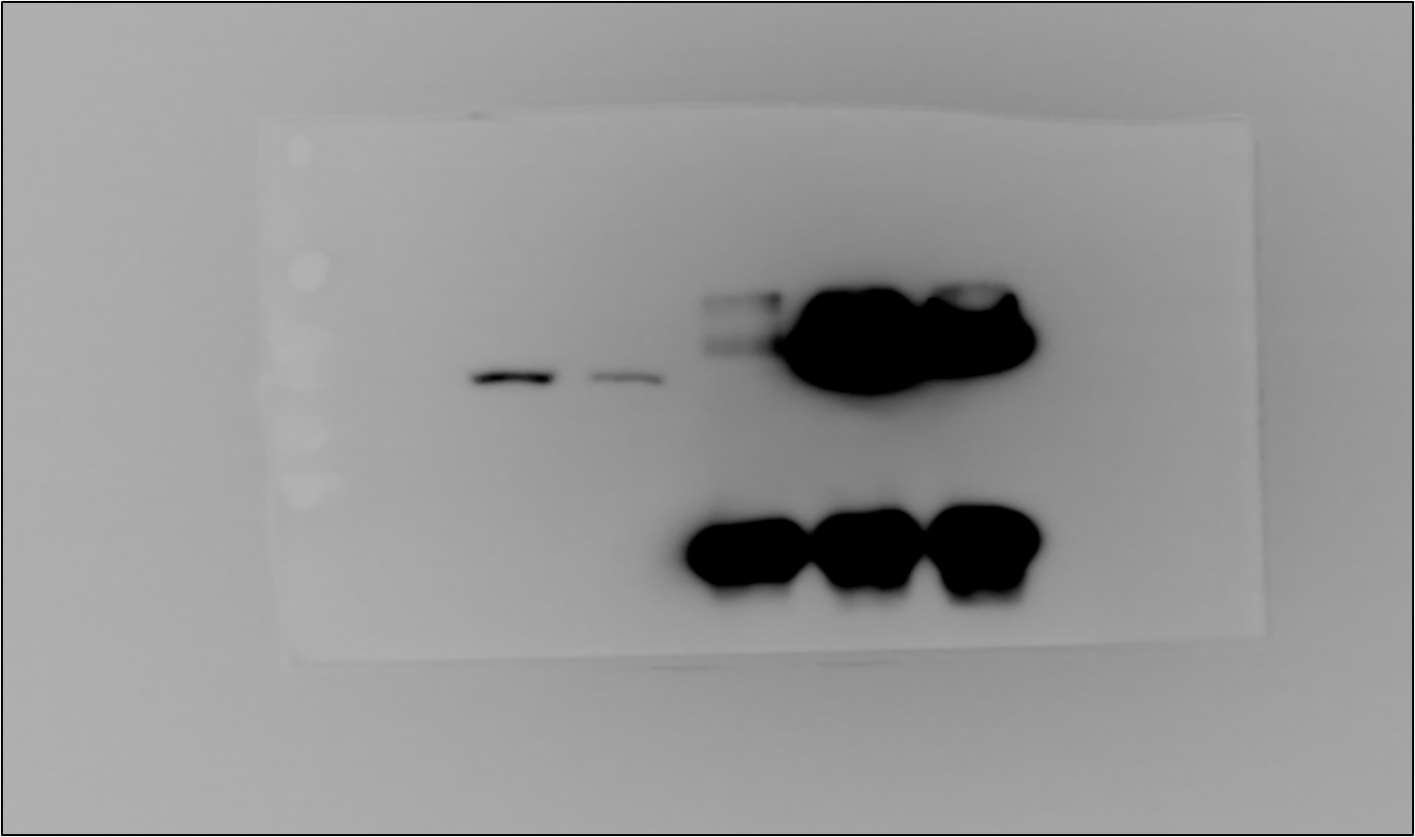

Supplement: Figure 6—source data 2. [file elife-98357-fig6-data2.zip › Figure 6-source data 2/6D-WCL-Flag-1.tif]

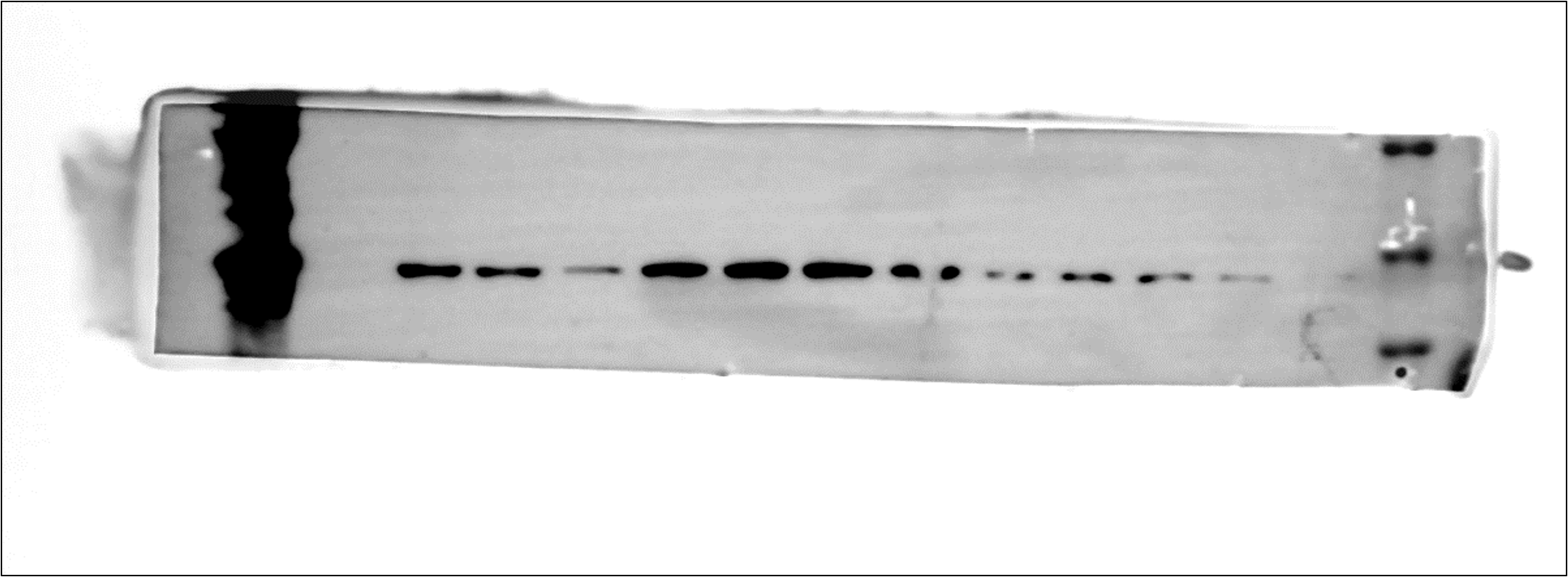

Supplement: Figure 6—source data 2. [file elife-98357-fig6-data2.zip › Figure 6-source data 2/6D-WCL-shCDK2-CDK2-1.tif]

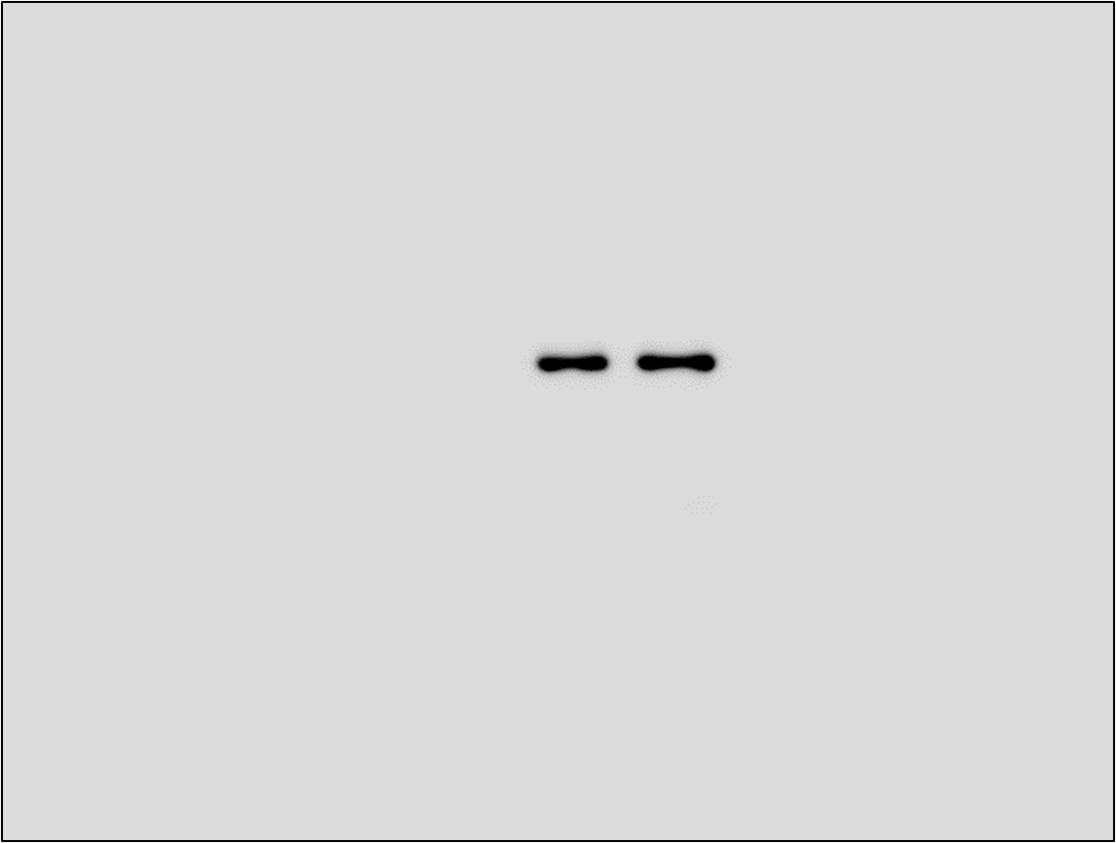

Supplement: Figure 6—source data 2. [file elife-98357-fig6-data2.zip › Figure 6-source data 2/6D-WCL-shCDK2-Flag-1.tif]

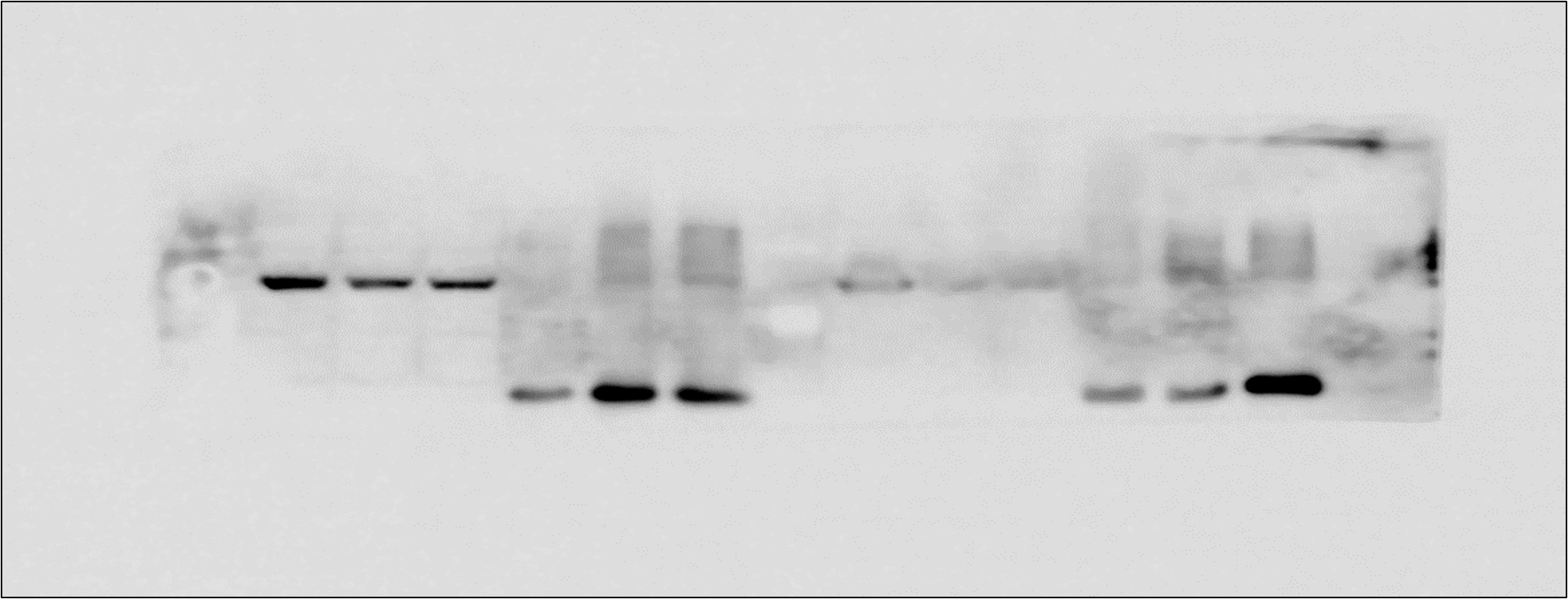

Supplement: Figure 6—source data 2. [file elife-98357-fig6-data2.zip › Figure 6-source data 2/6D-WCL-shCDK2-HA-1.tif]

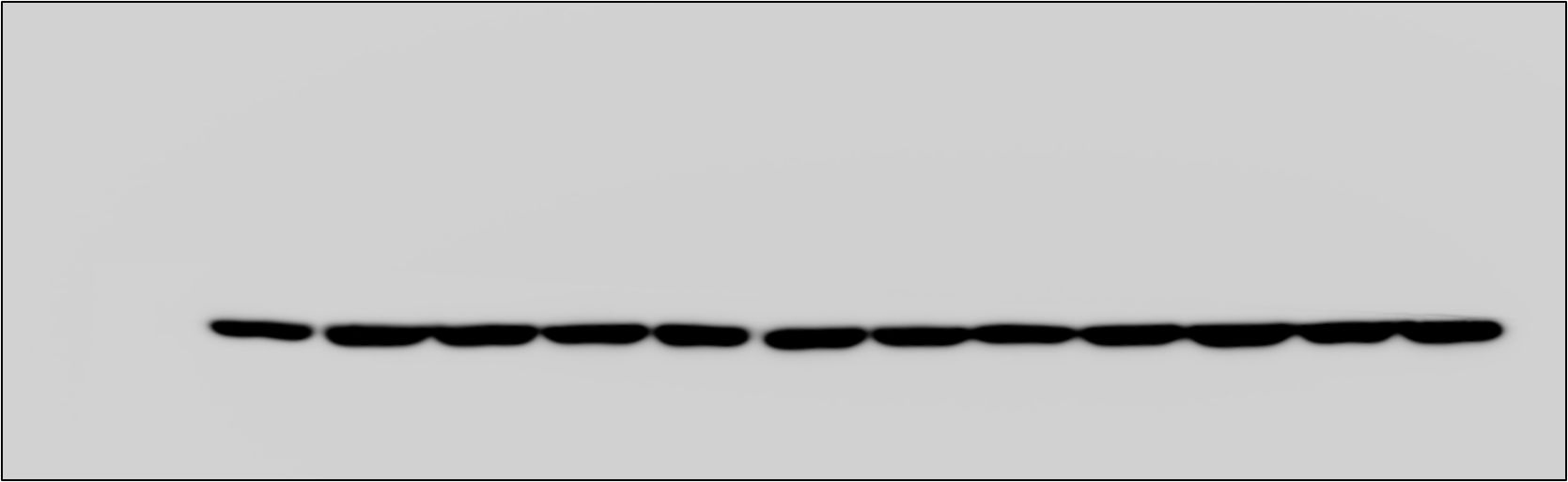

Supplement: Figure 6—source data 2. [file elife-98357-fig6-data2.zip › Figure 6-source data 2/6G-Actin-1.tif]

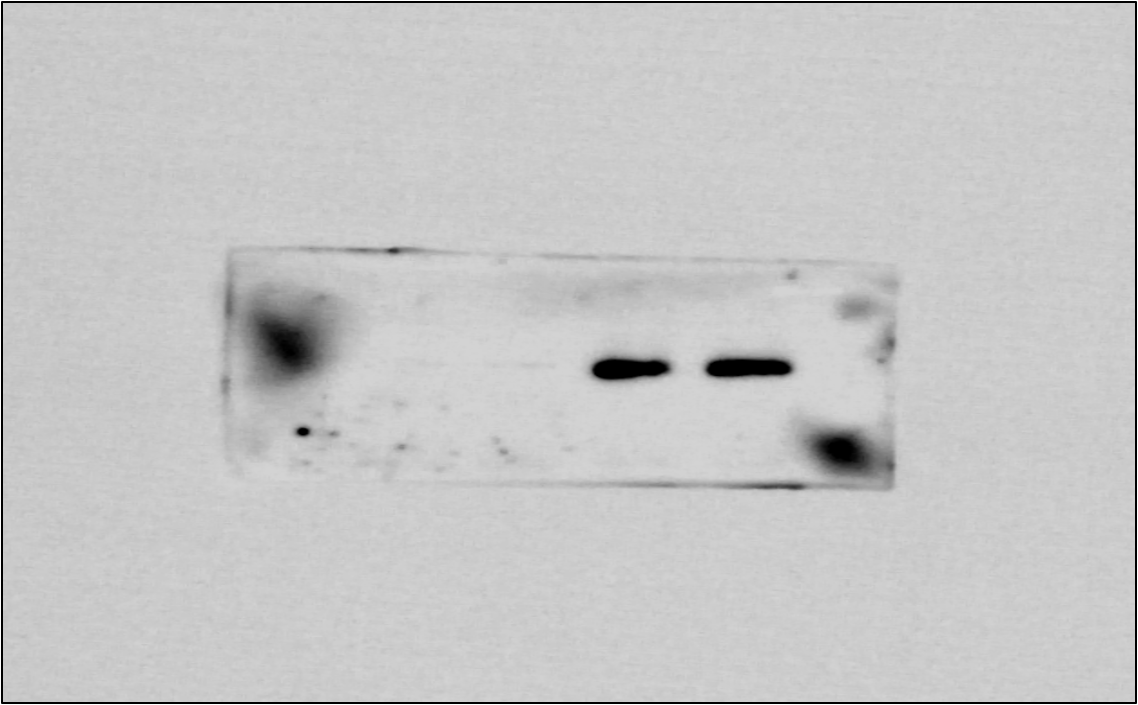

Supplement: Figure 6—source data 2. [file elife-98357-fig6-data2.zip › Figure 6-source data 2/6G-Flag-1.tif]

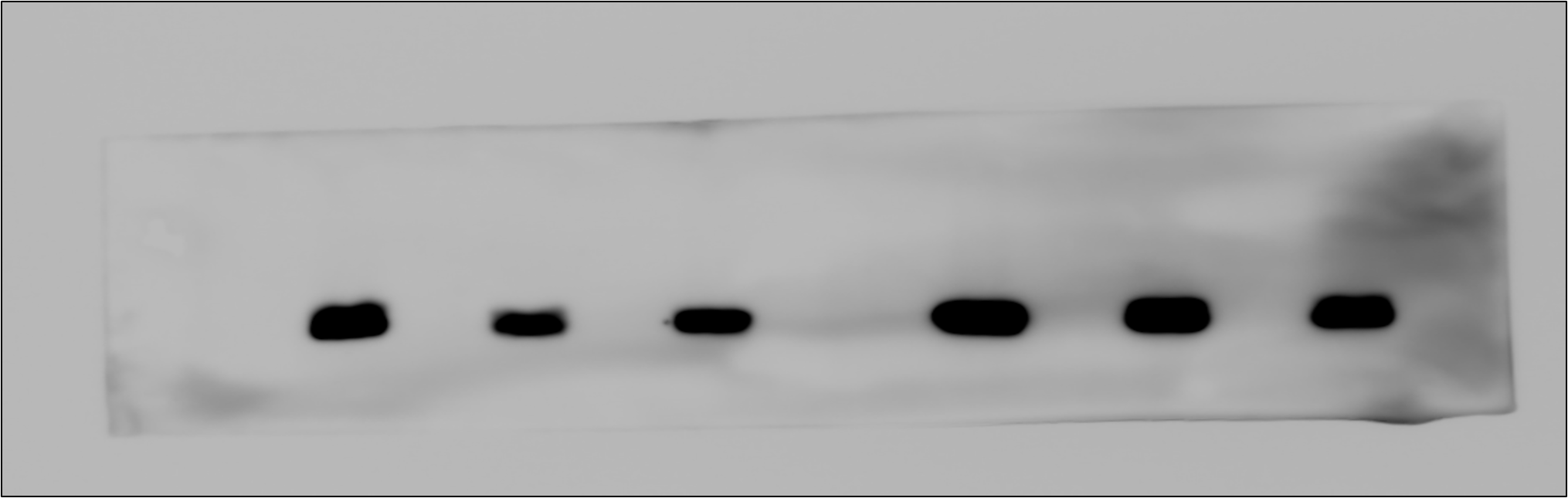

Supplement: Figure 6—source data 2. [file elife-98357-fig6-data2.zip › Figure 6-source data 2/6G-HA-1.tif]

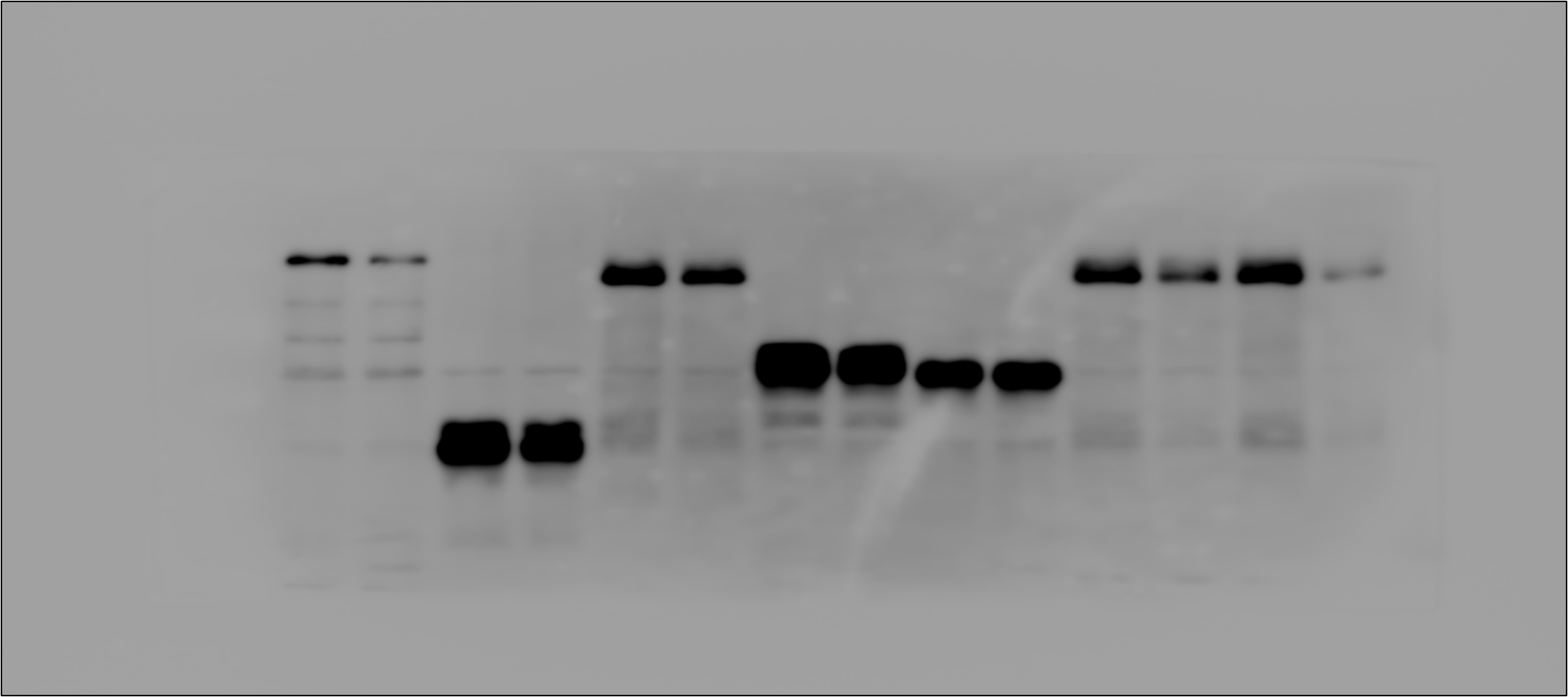

Supplement: Figure 6—source data 2. [file elife-98357-fig6-data2.zip › Figure 6-source data 2/6G-Myc-1.tif]

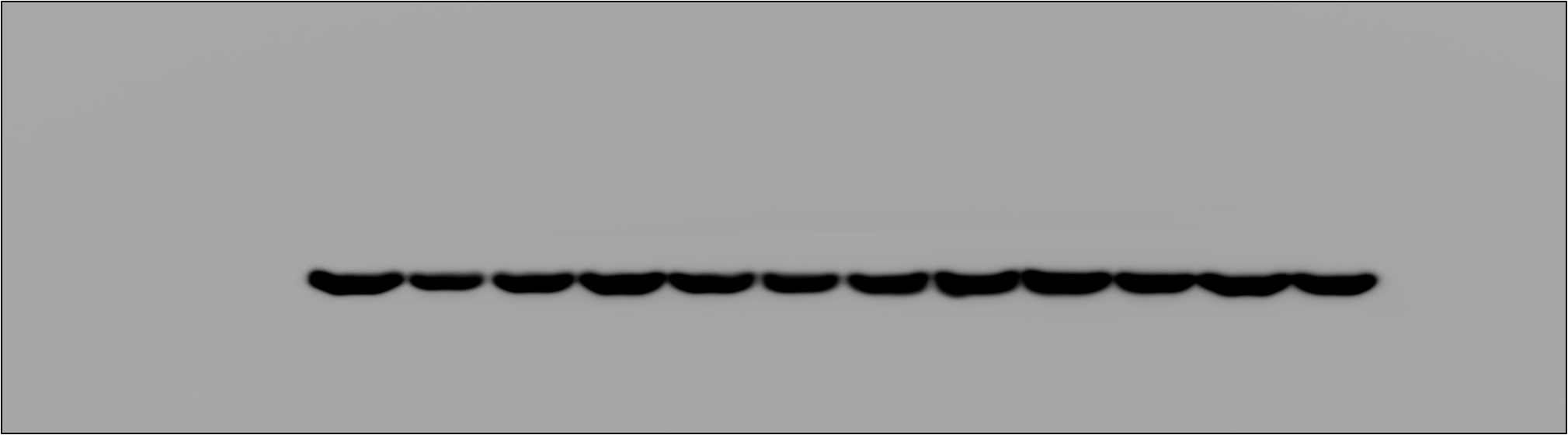

Supplement: Figure 6—source data 2. [file elife-98357-fig6-data2.zip › Figure 6-source data 2/6H-Actin-1.tif]

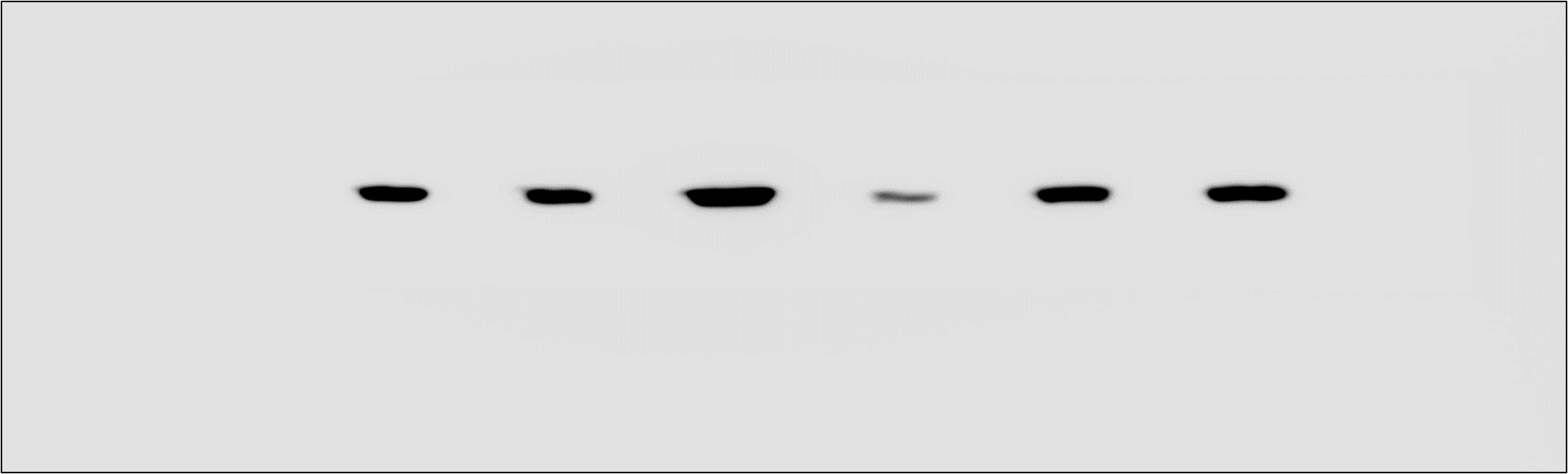

Supplement: Figure 6—source data 2. [file elife-98357-fig6-data2.zip › Figure 6-source data 2/6H-HA-1.tif]

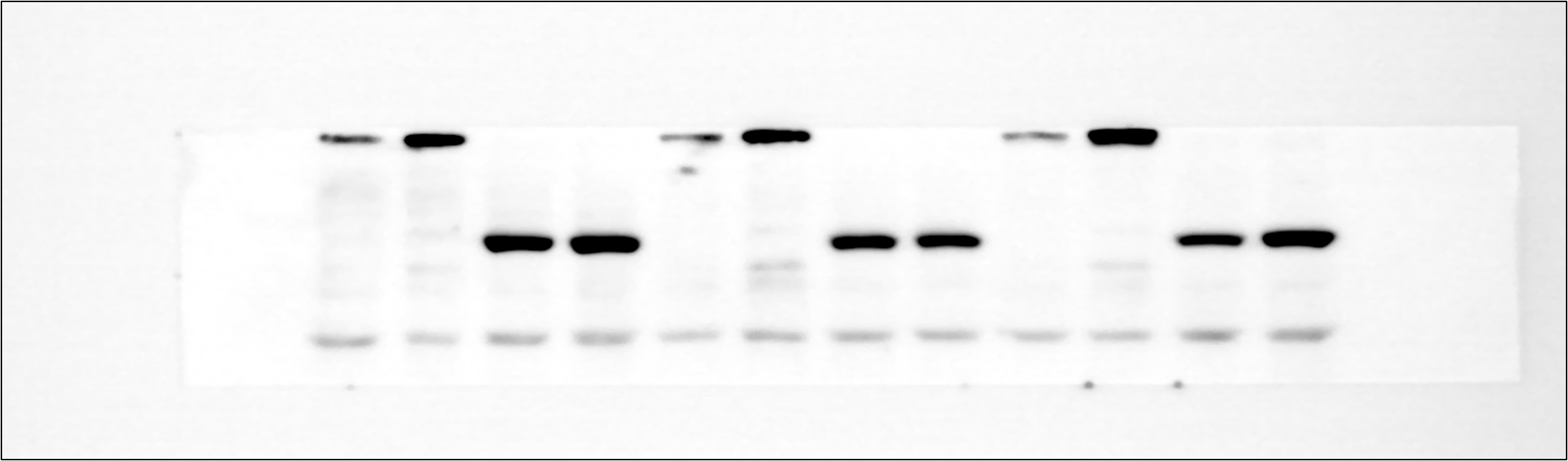

Supplement: Figure 6—source data 2. [file elife-98357-fig6-data2.zip › Figure 6-source data 2/6H-Myc-1.tif]

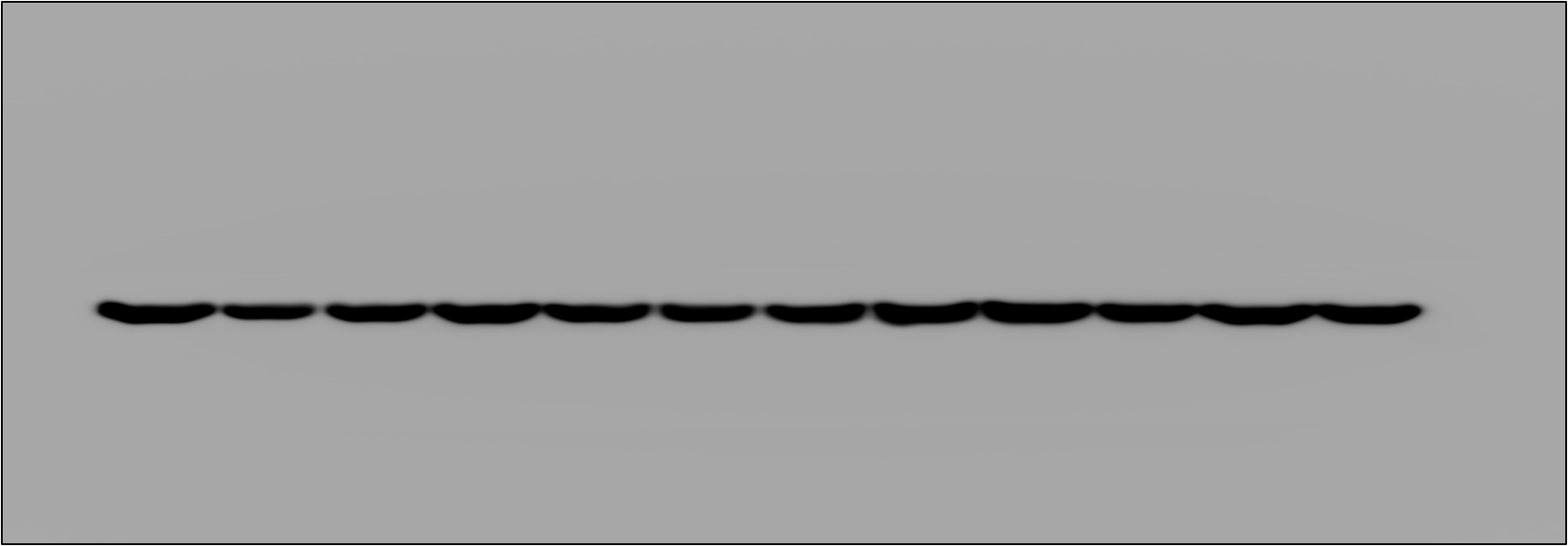

Supplement: Figure 6—source data 2. [file elife-98357-fig6-data2.zip › Figure 6-source data 2/6H-poly IC-Actin-1.tif]

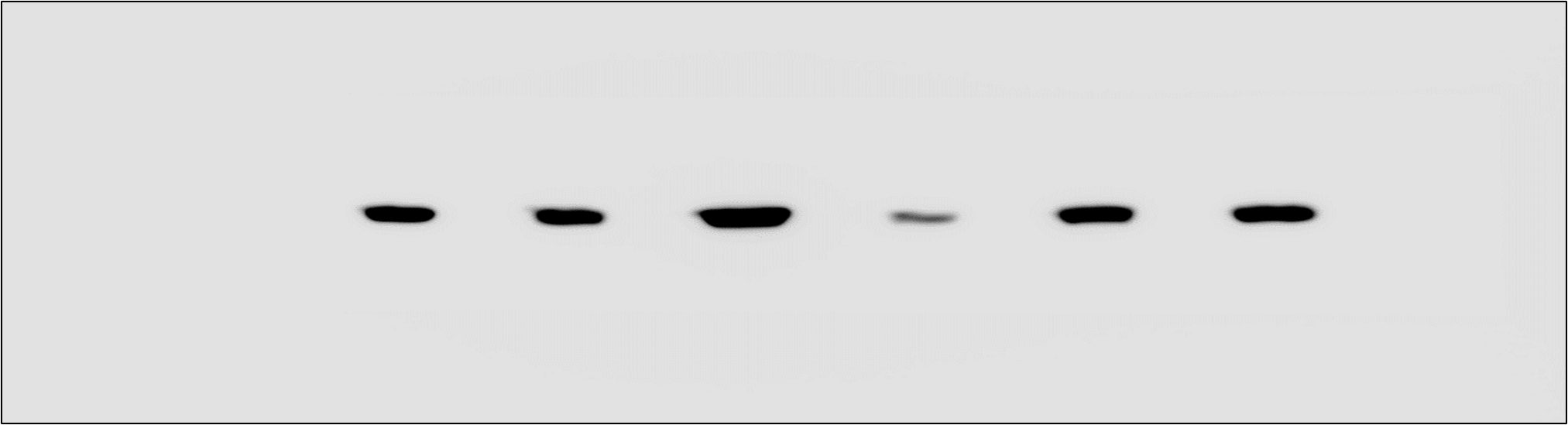

Supplement: Figure 6—source data 2. [file elife-98357-fig6-data2.zip › Figure 6-source data 2/6H-poly IC-HA-1.tif]

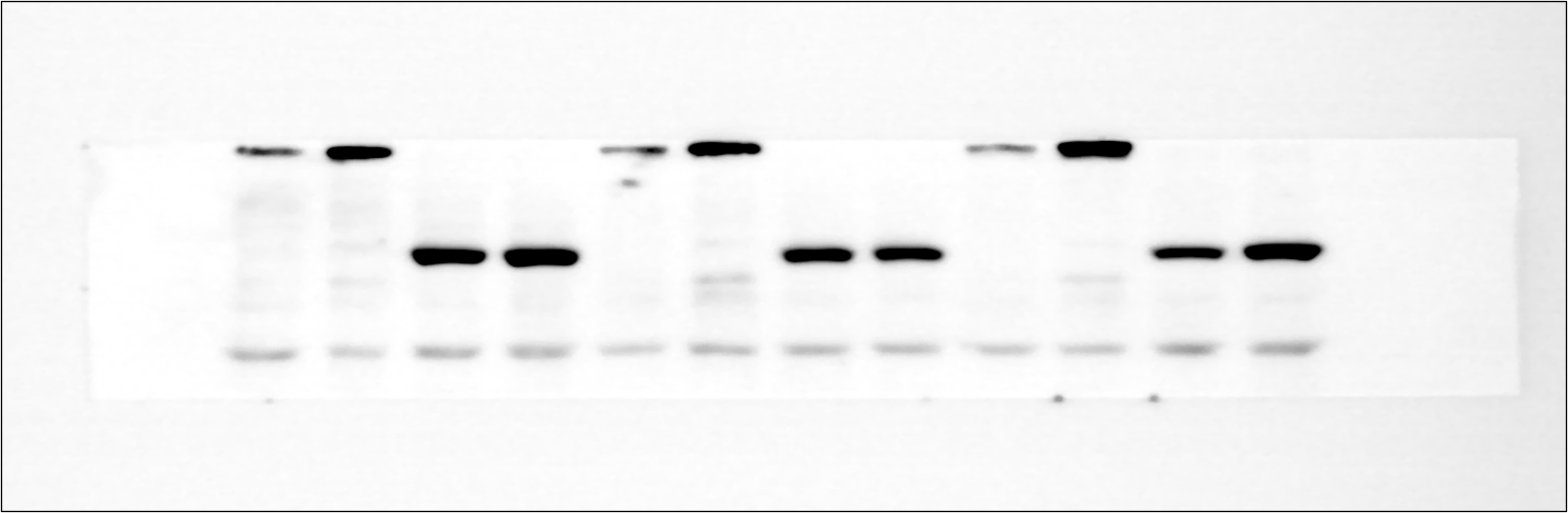

Supplement: Figure 6—source data 2. [file elife-98357-fig6-data2.zip › Figure 6-source data 2/6H-poly IC-Myc-.tif]

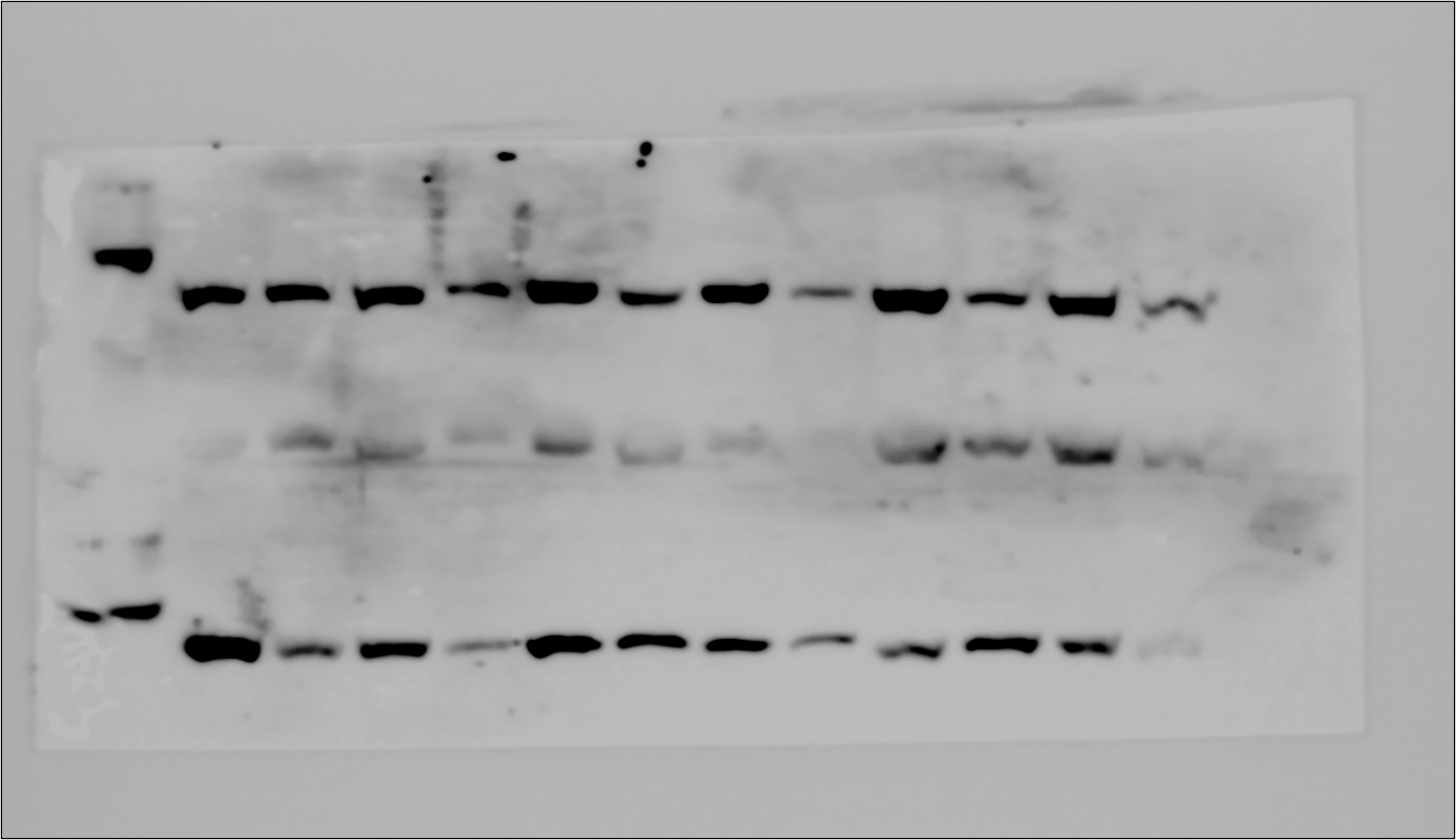

Supplement: Figure 6—source data 2. [file elife-98357-fig6-data2.zip › Figure 6-source data 2/6H-poly IC-TBK1-1.tif]

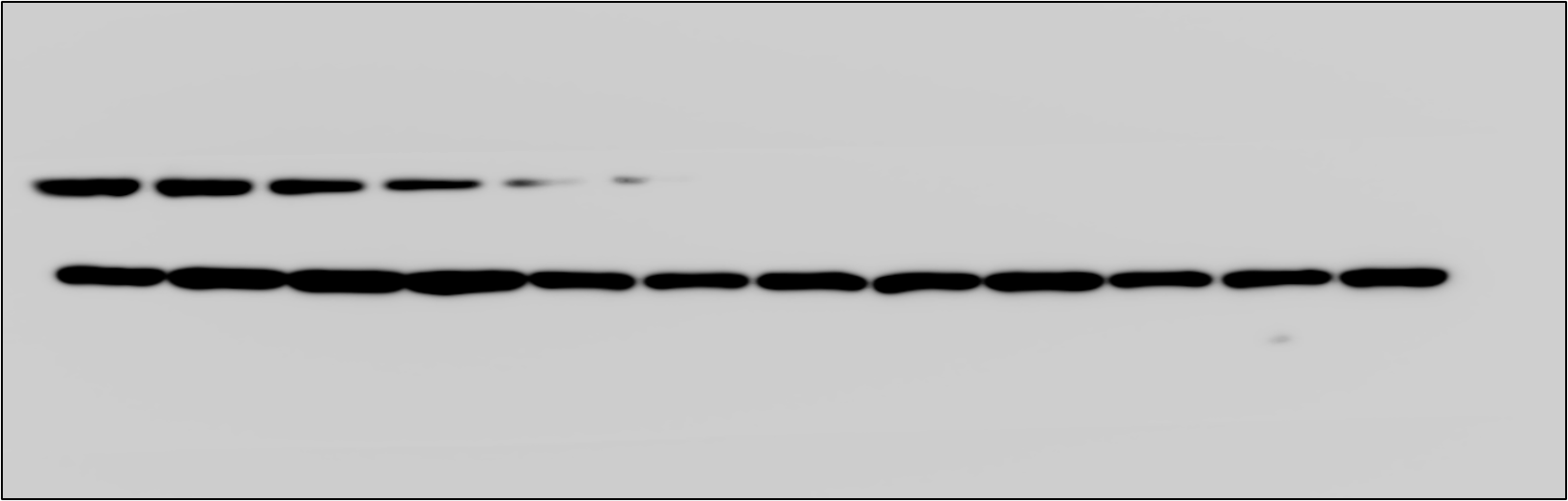

Supplement: Figure 6—source data 2. [file elife-98357-fig6-data2.zip › Figure 6-source data 2/6H-SVCV-Actin-1.tif]

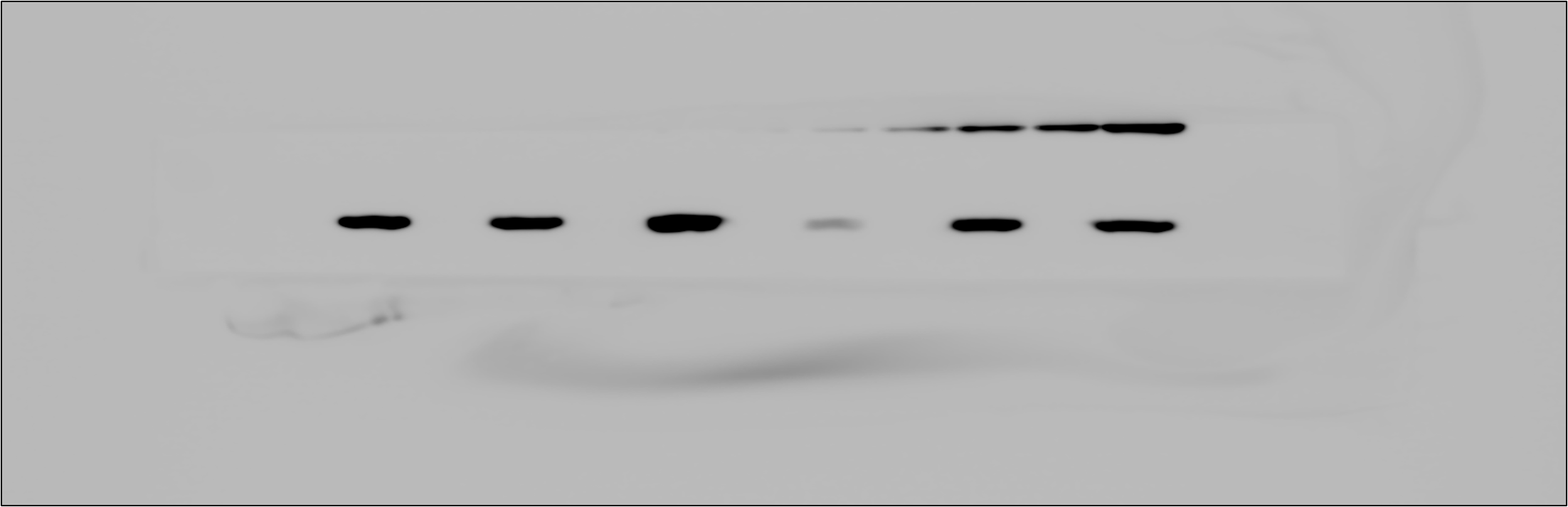

Supplement: Figure 6—source data 2. [file elife-98357-fig6-data2.zip › Figure 6-source data 2/6H-SVCV-HA-1.tif]

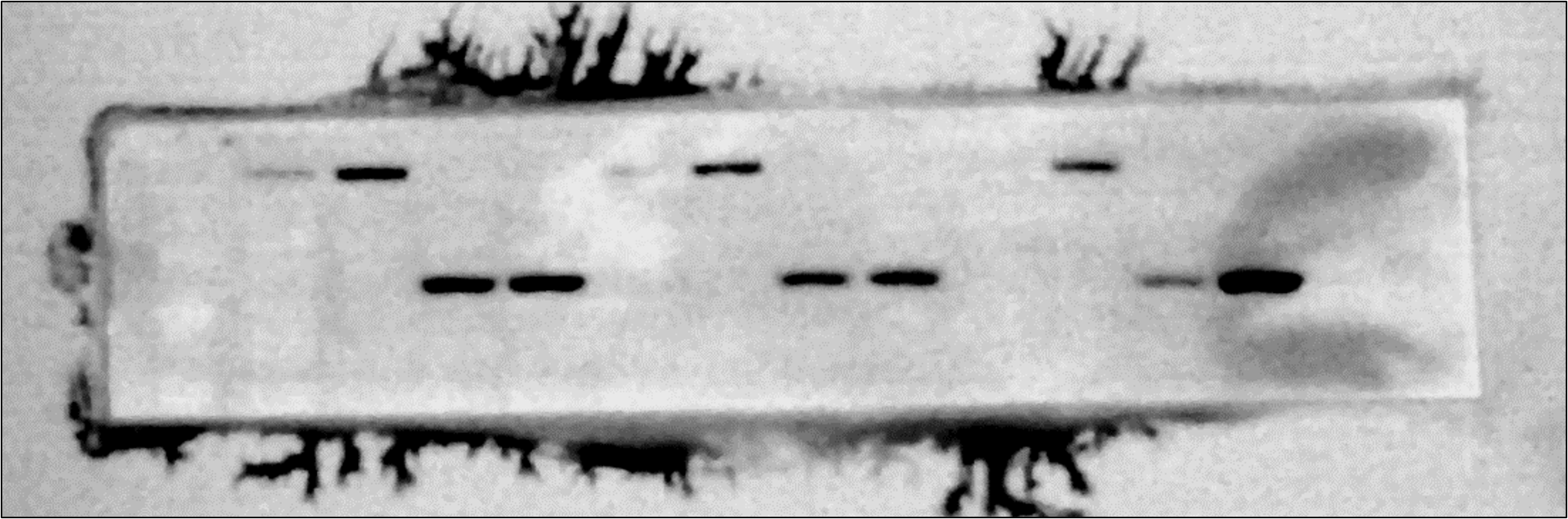

Supplement: Figure 6—source data 2. [file elife-98357-fig6-data2.zip › Figure 6-source data 2/6H-SVCV-Myc-1.tif]

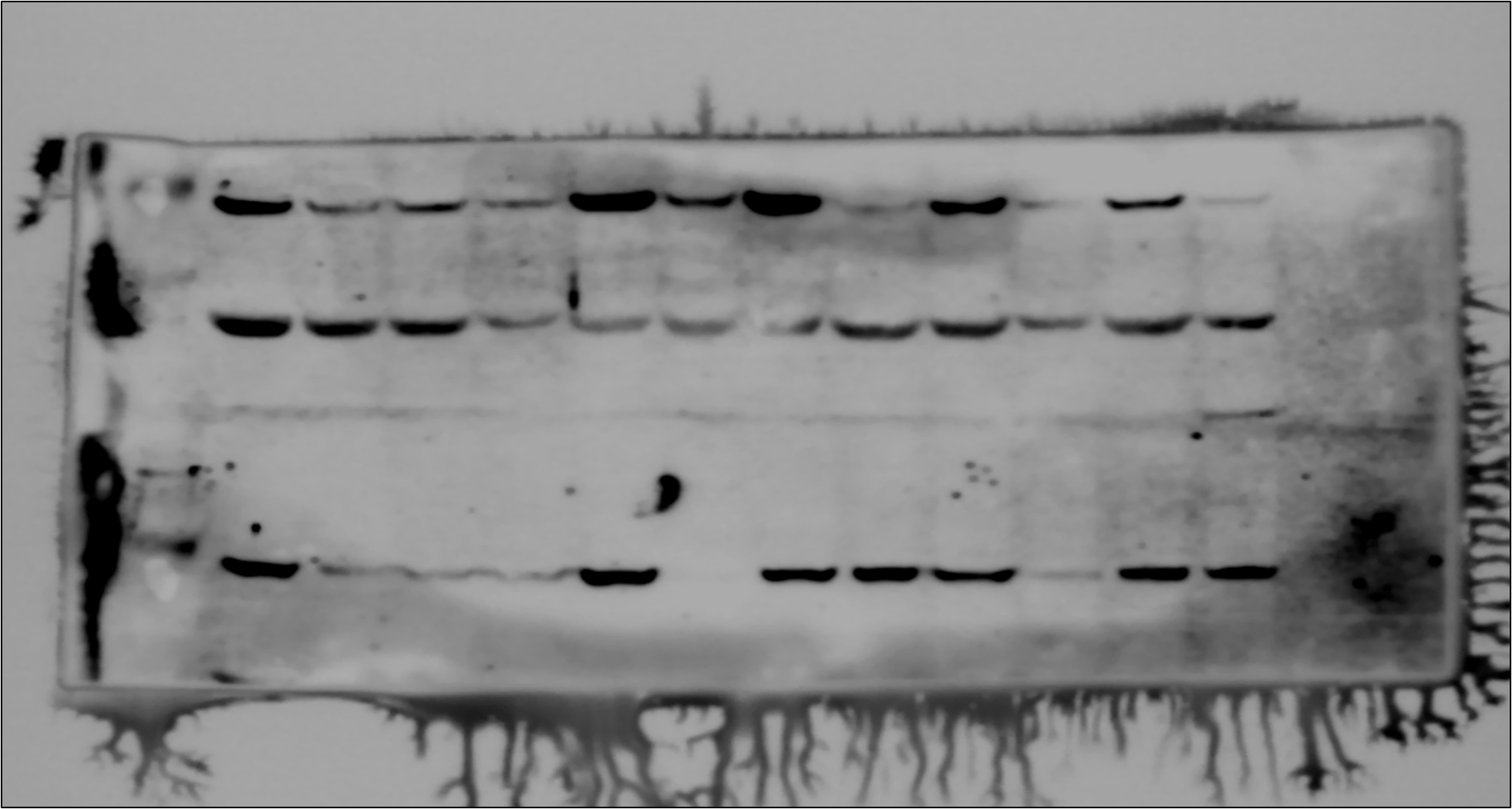

Supplement: Figure 6—source data 2. [file elife-98357-fig6-data2.zip › Figure 6-source data 2/6H-SVCV-TBK1-1.tif]

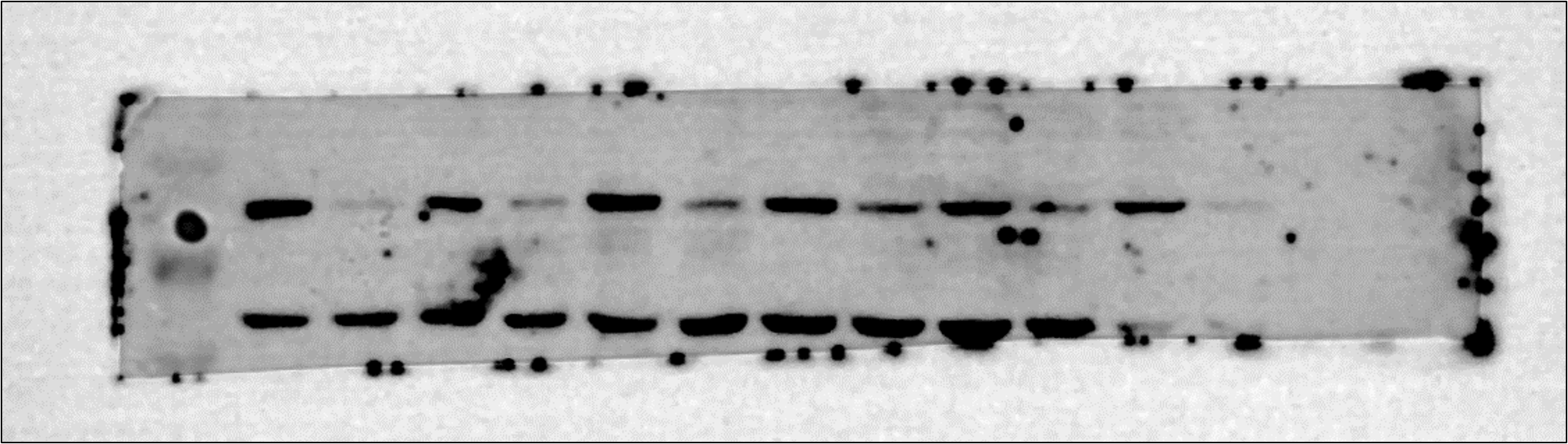

Supplement: Figure 6—source data 2. [file elife-98357-fig6-data2.zip › Figure 6-source data 2/6H-TBK1-1.tif]

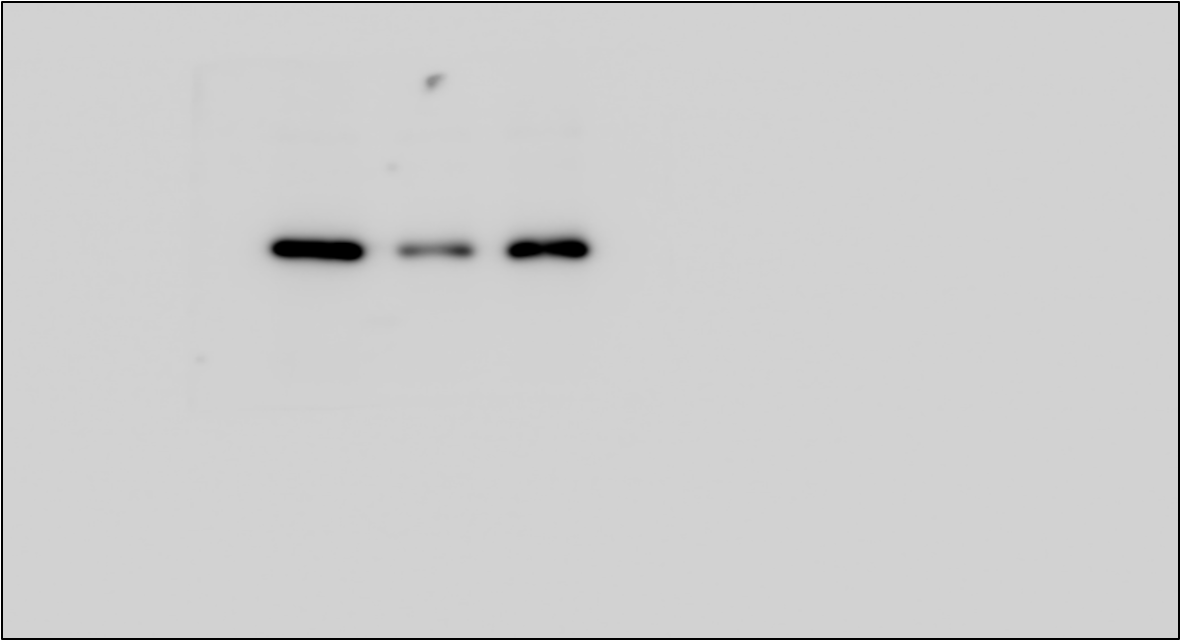

Supplement: Figure 6—source data 2. [file elife-98357-fig6-data2.zip › Figure 6-source data 2/6I-Ectopic-Flag-1.tif]

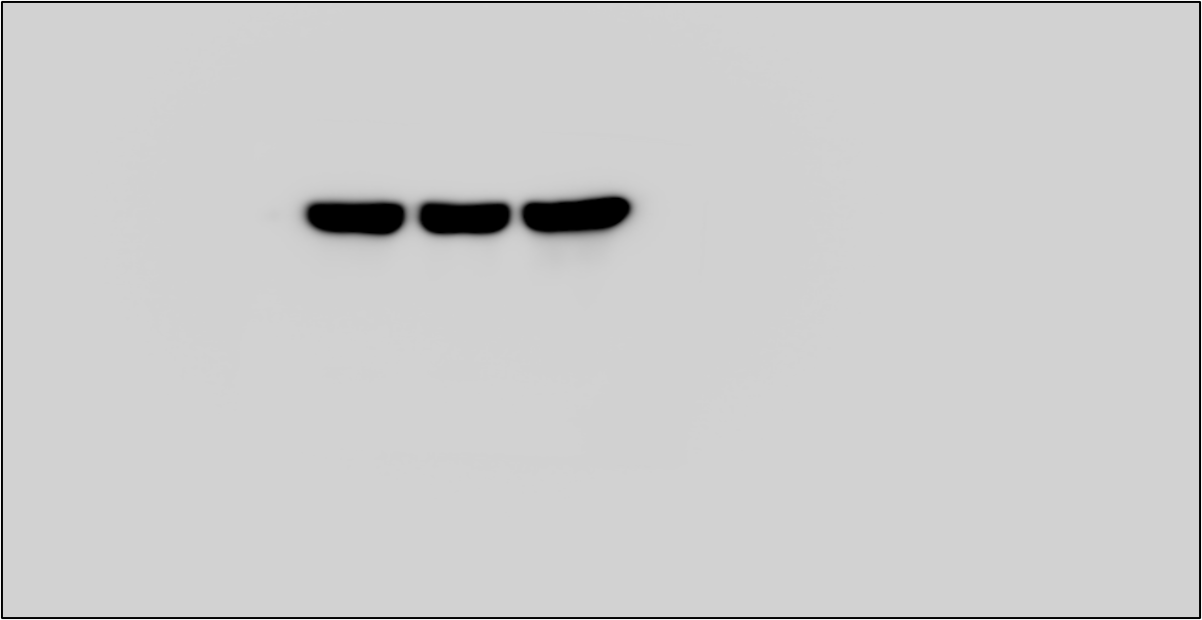

Supplement: Figure 6—source data 2. [file elife-98357-fig6-data2.zip › Figure 6-source data 2/6I-Ectopic-GAPDH-1.tif]

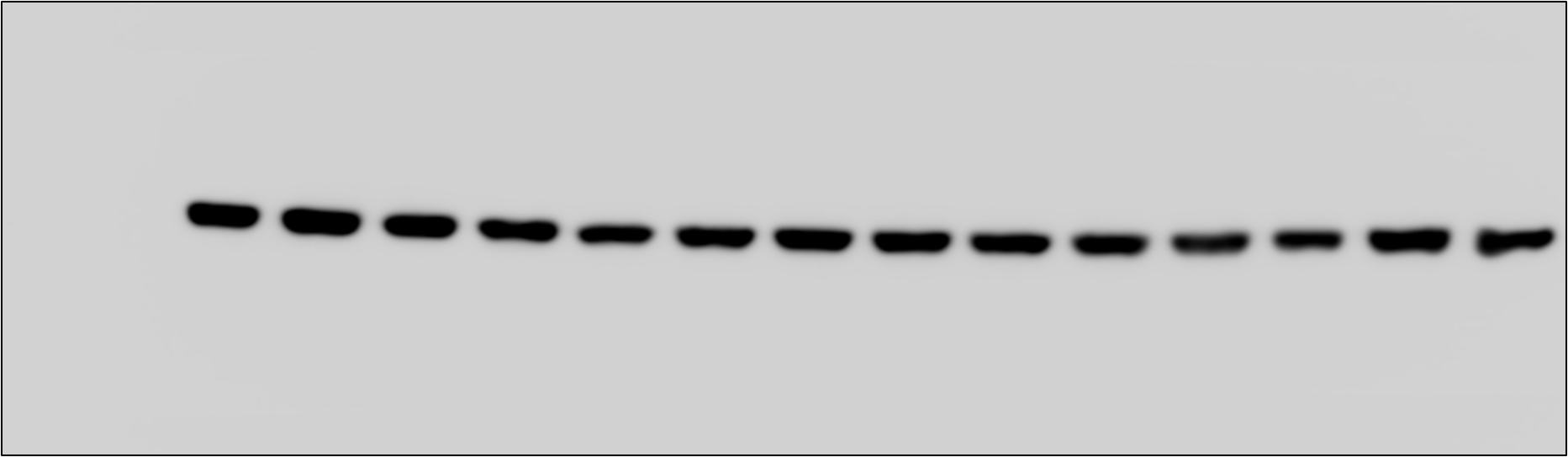

Supplement: Figure 6—source data 2. [file elife-98357-fig6-data2.zip › Figure 6-source data 2/6I-Endo-Actin-1.tif]

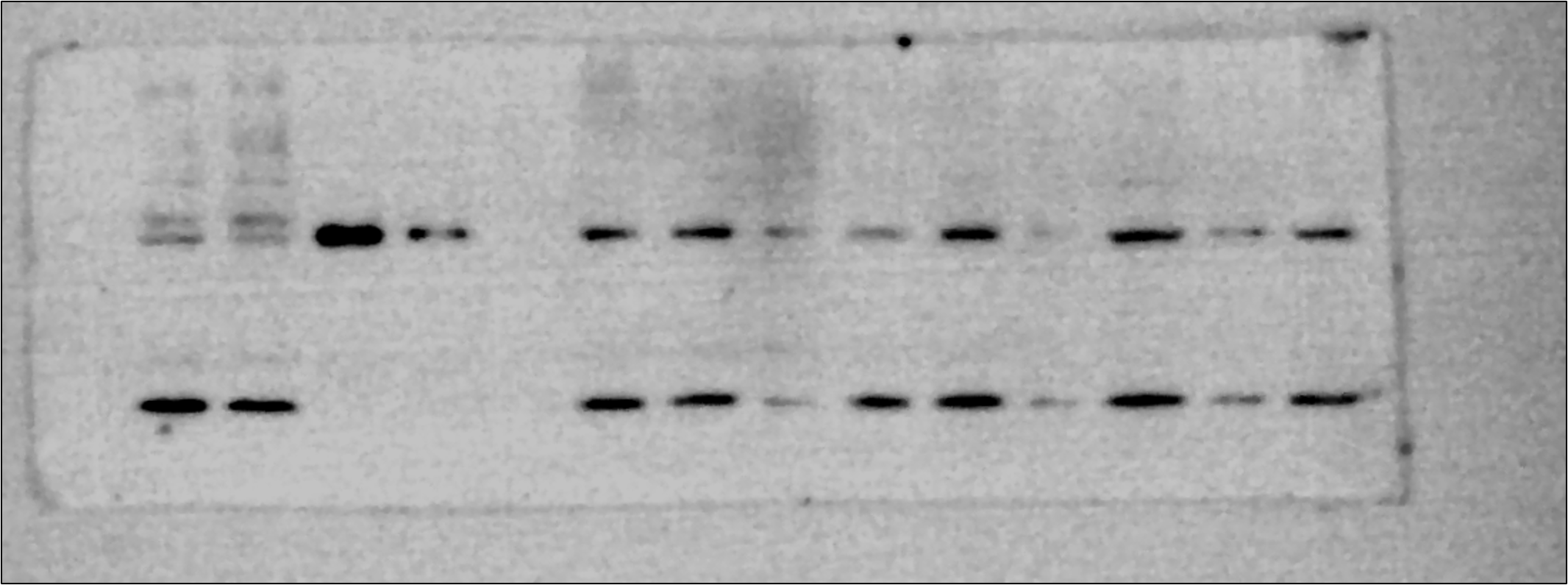

Supplement: Figure 6—source data 2. [file elife-98357-fig6-data2.zip › Figure 6-source data 2/6I-Endo-Dtx4-1.tif]

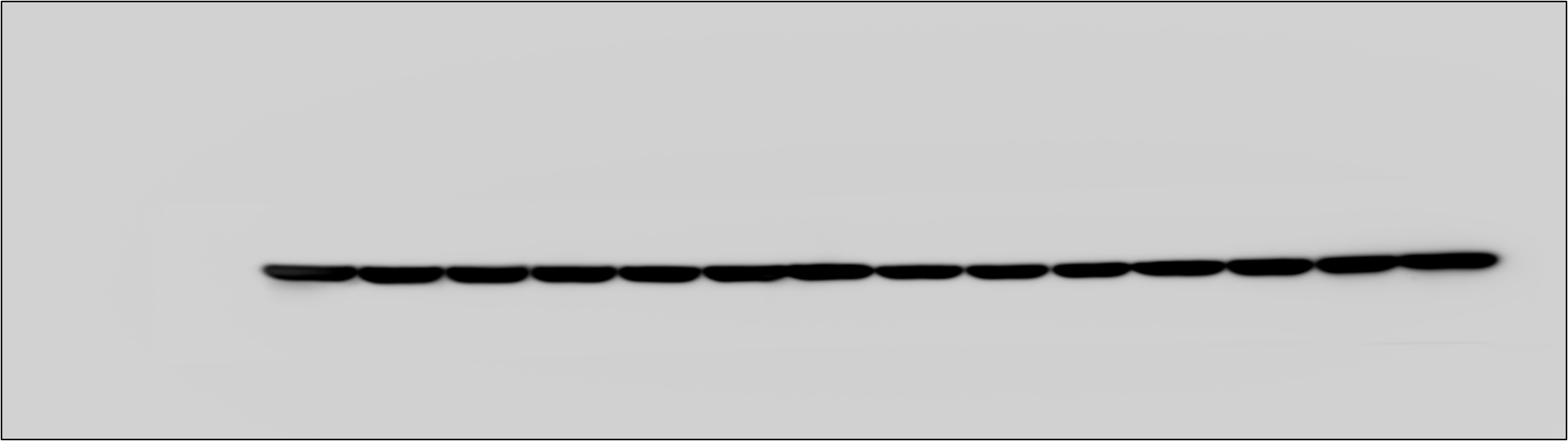

Supplement: Figure 6—source data 2. [file elife-98357-fig6-data2.zip › Figure 6-source data 2/6L-Actin-1.tif]

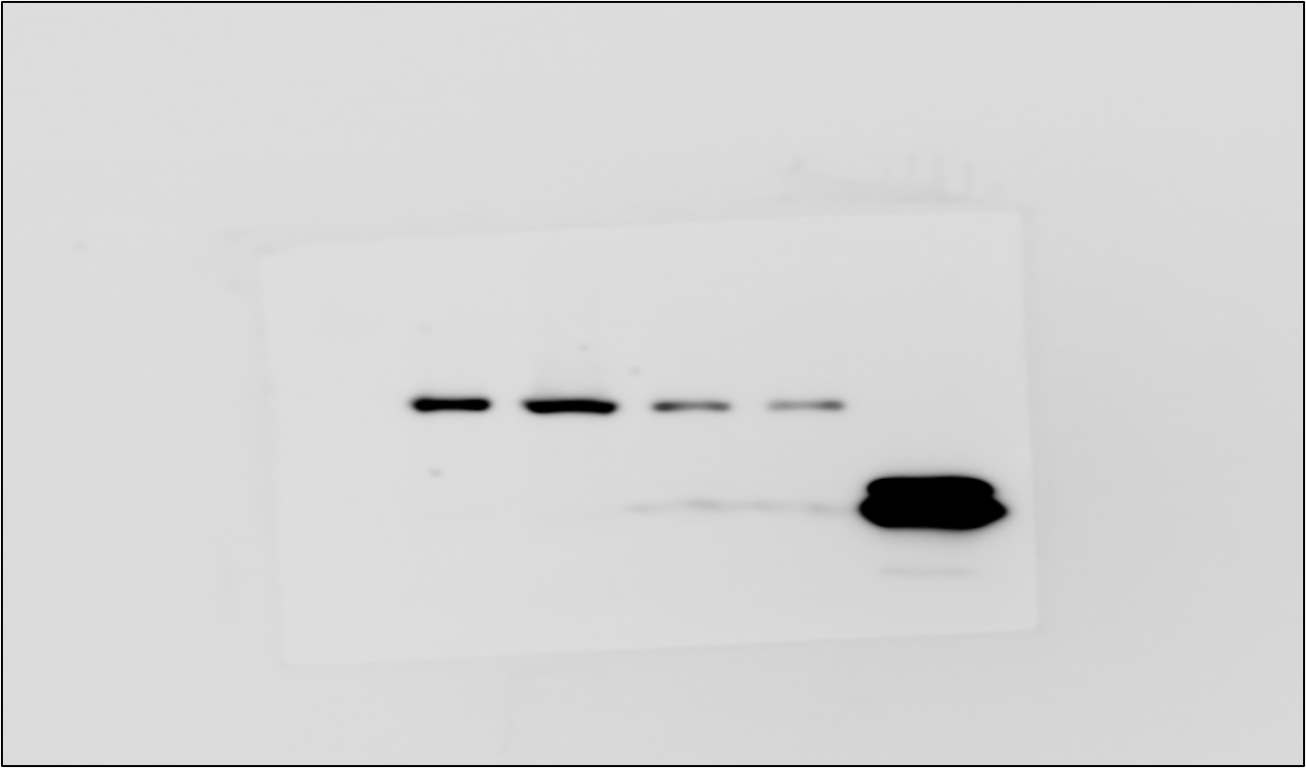

Supplement: Figure 6—source data 2. [file elife-98357-fig6-data2.zip › Figure 6-source data 2/6L-Dtx4-1.tif]

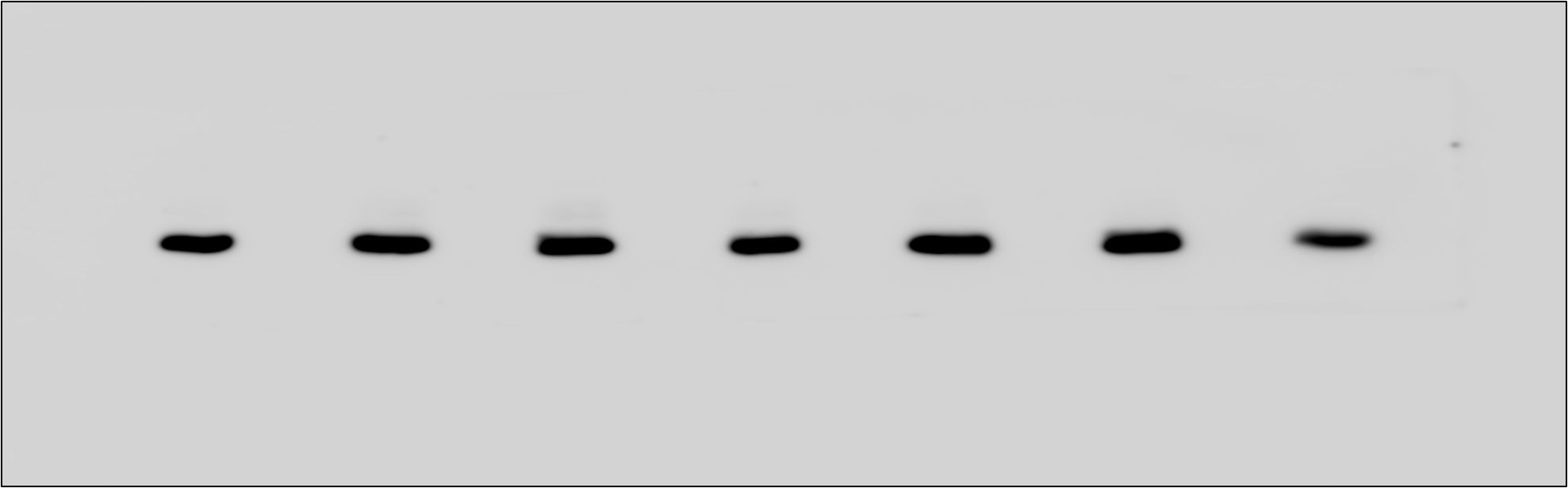

Supplement: Figure 6—source data 2. [file elife-98357-fig6-data2.zip › Figure 6-source data 2/6L-HA-1.tif]

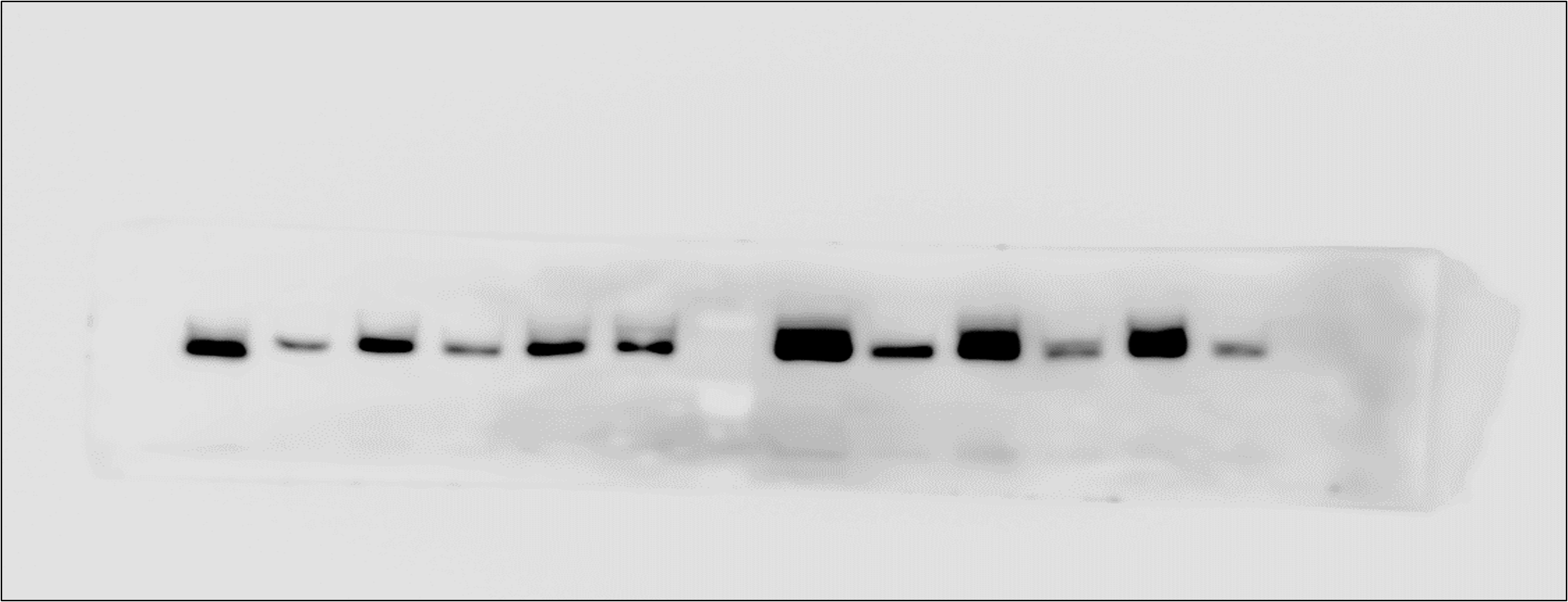

Supplement: Figure 6—source data 2. [file elife-98357-fig6-data2.zip › Figure 6-source data 2/6L-Myc-1.tif]

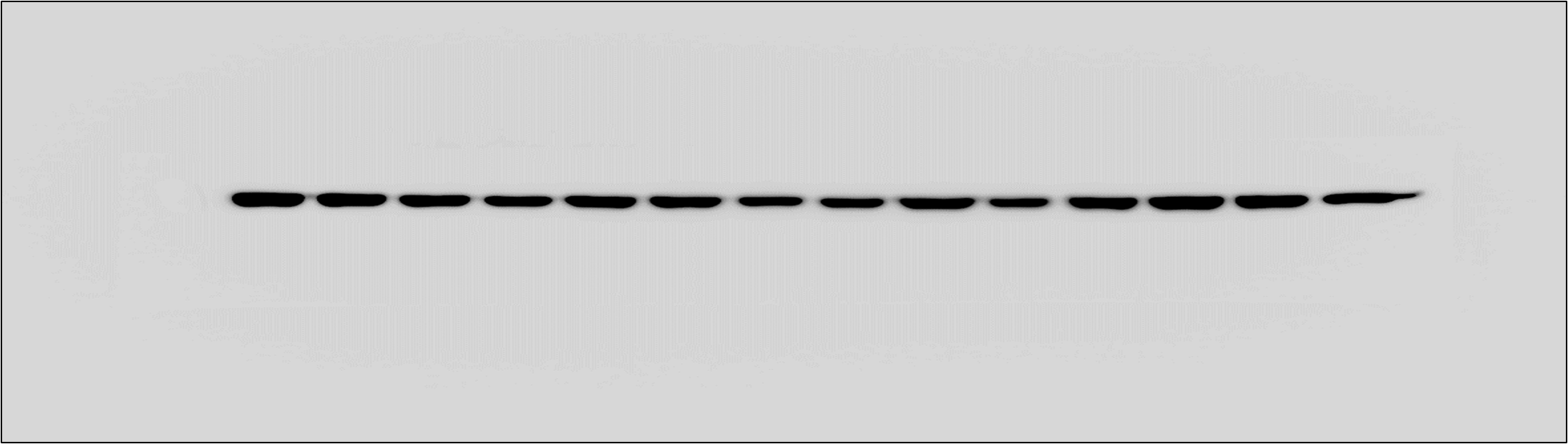

Supplement: Figure 6—source data 2. [file elife-98357-fig6-data2.zip › Figure 6-source data 2/6M-Actin-1.tif]

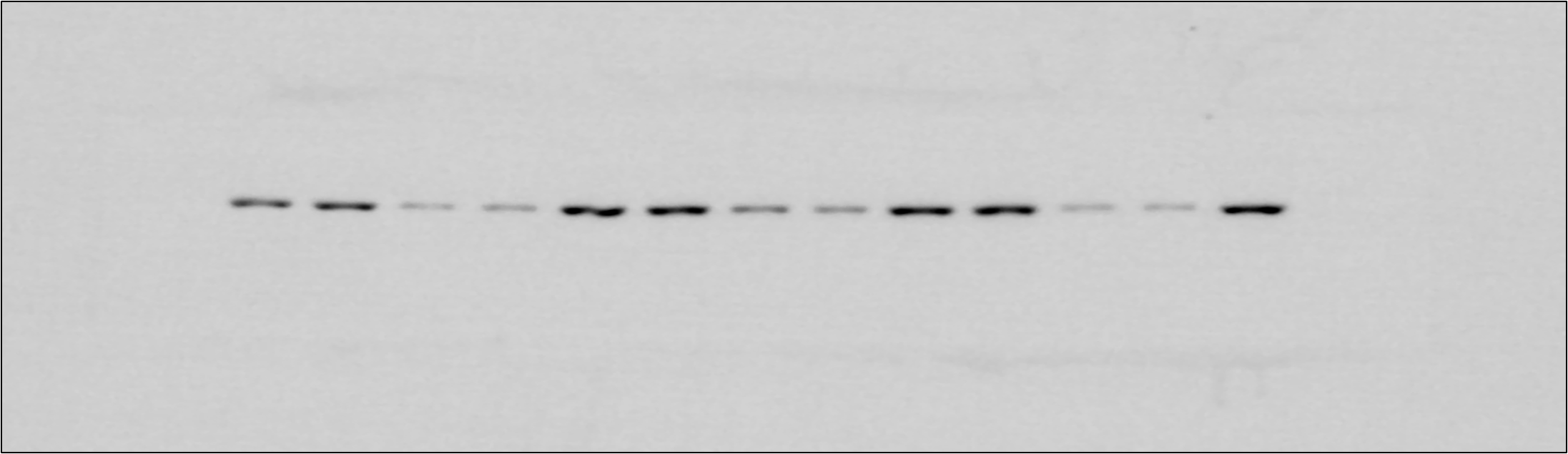

Supplement: Figure 6—source data 2. [file elife-98357-fig6-data2.zip › Figure 6-source data 2/6M-Dtx4-1.tif]

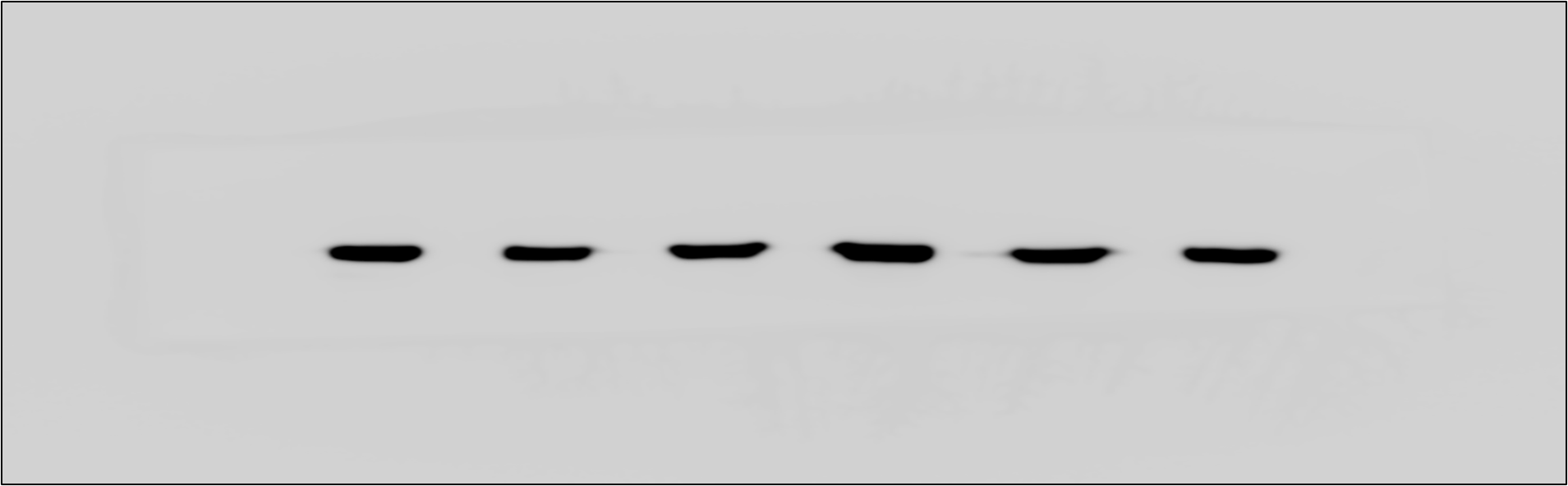

Supplement: Figure 6—source data 2. [file elife-98357-fig6-data2.zip › Figure 6-source data 2/6M-HA-1.tif]

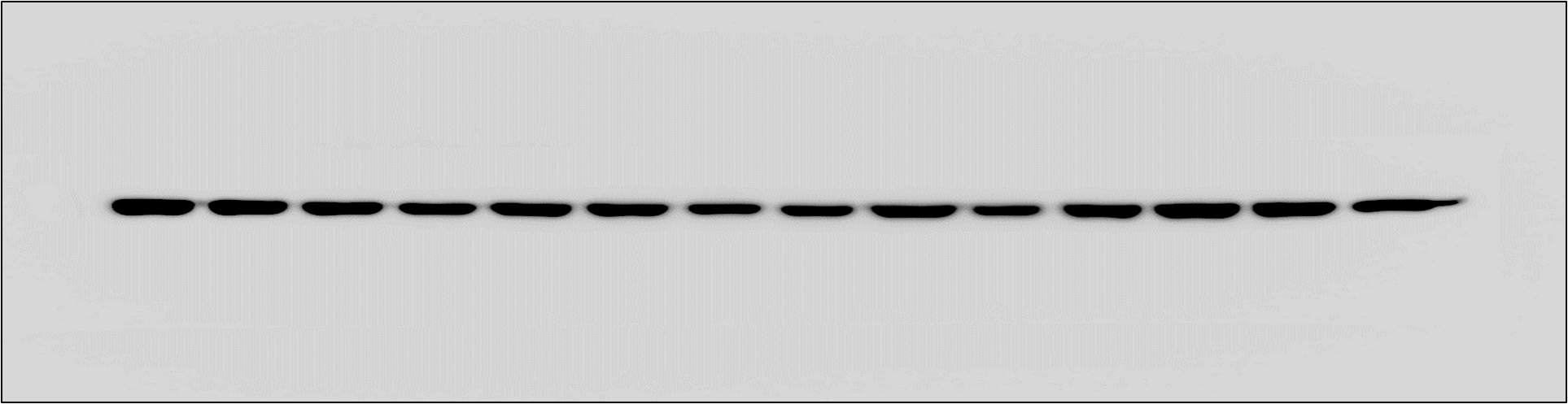

Supplement: Figure 6—source data 2. [file elife-98357-fig6-data2.zip › Figure 6-source data 2/6M-poly IC-Actin-1.tif]

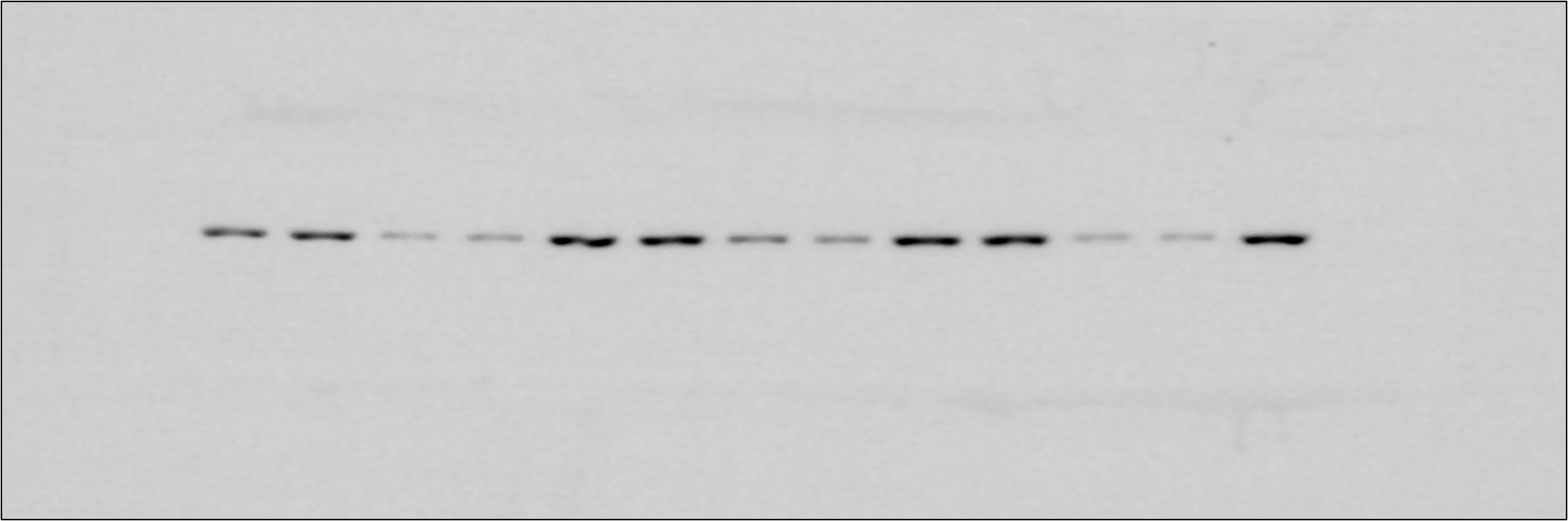

Supplement: Figure 6—source data 2. [file elife-98357-fig6-data2.zip › Figure 6-source data 2/6M-poly IC-Dtx4-1.tif]

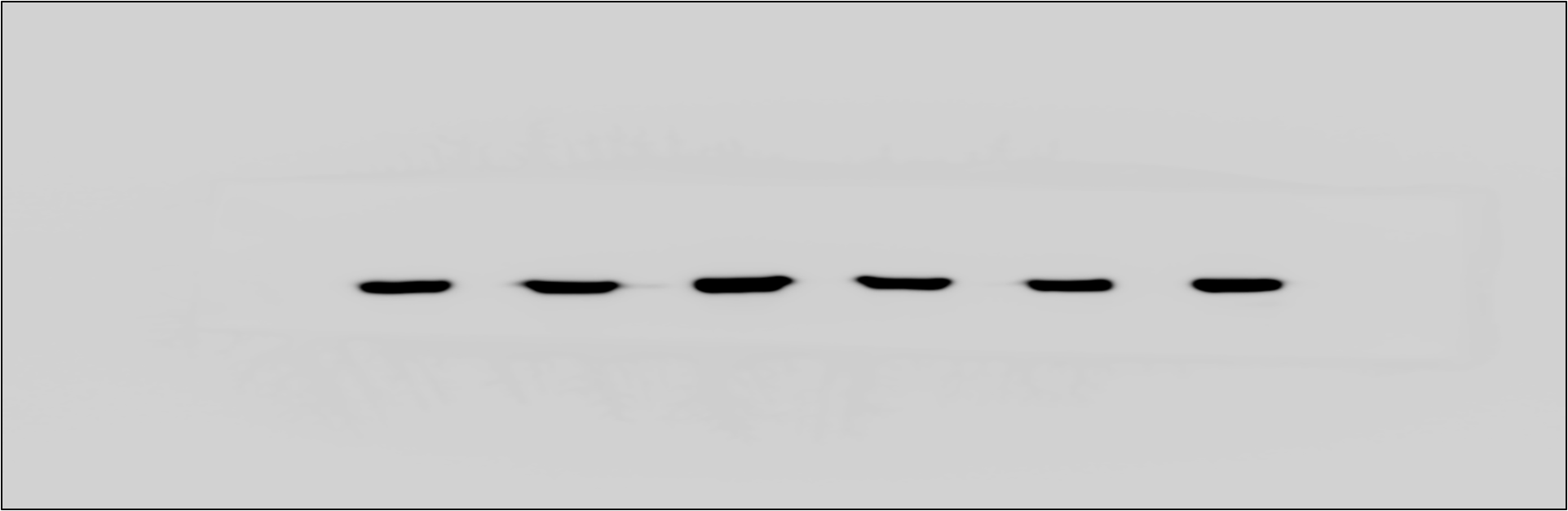

Supplement: Figure 6—source data 2. [file elife-98357-fig6-data2.zip › Figure 6-source data 2/6M-poly IC-HA-1.tif]

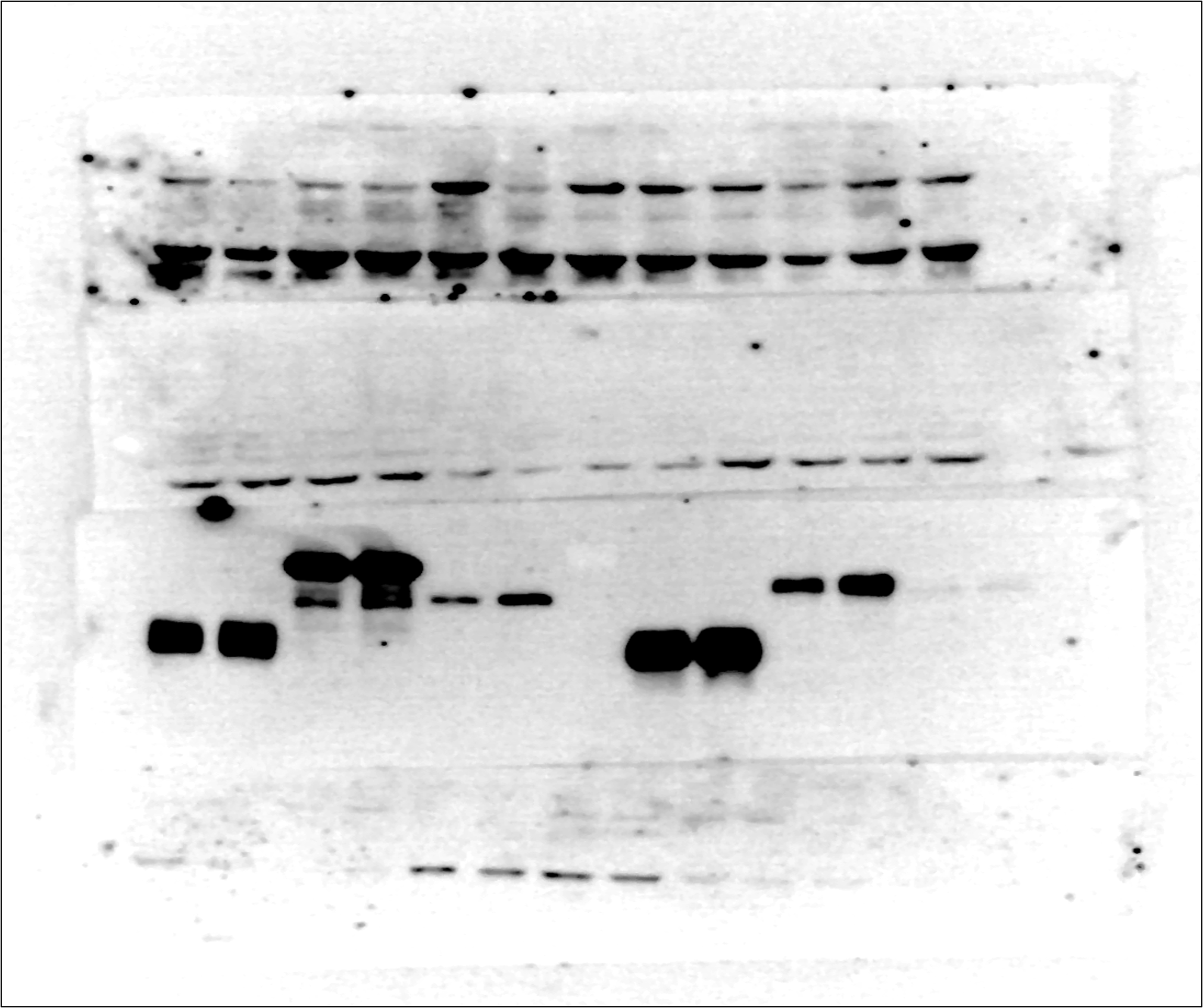

Supplement: Figure 6—source data 2. [file elife-98357-fig6-data2.zip › Figure 6-source data 2/6M-poly IC-TBK1-1.tif]

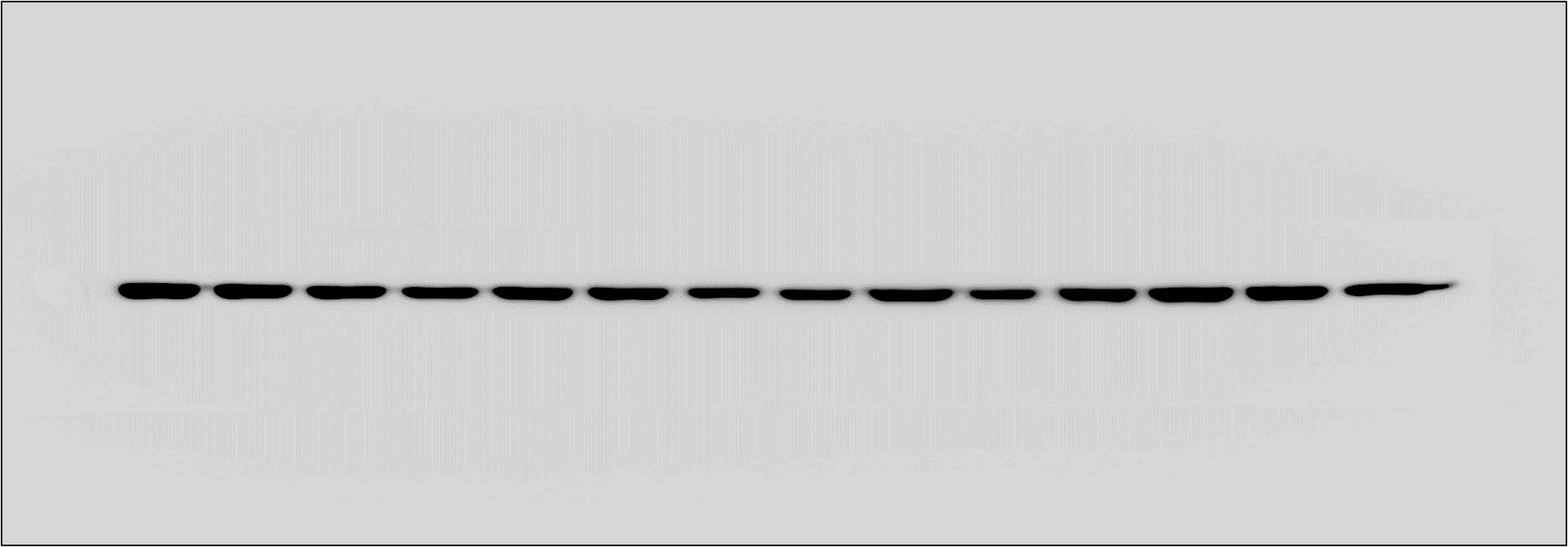

Supplement: Figure 6—source data 2. [file elife-98357-fig6-data2.zip › Figure 6-source data 2/6M-SVCV-Actin-1.tif]

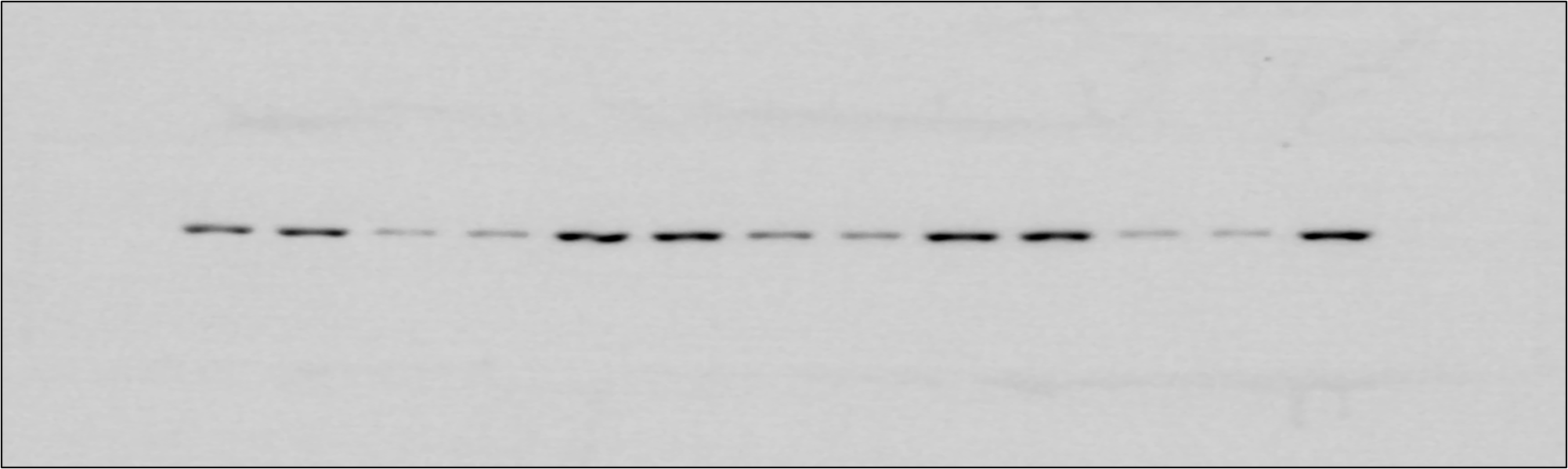

Supplement: Figure 6—source data 2. [file elife-98357-fig6-data2.zip › Figure 6-source data 2/6M-SVCV-Dtx4-1.tif]

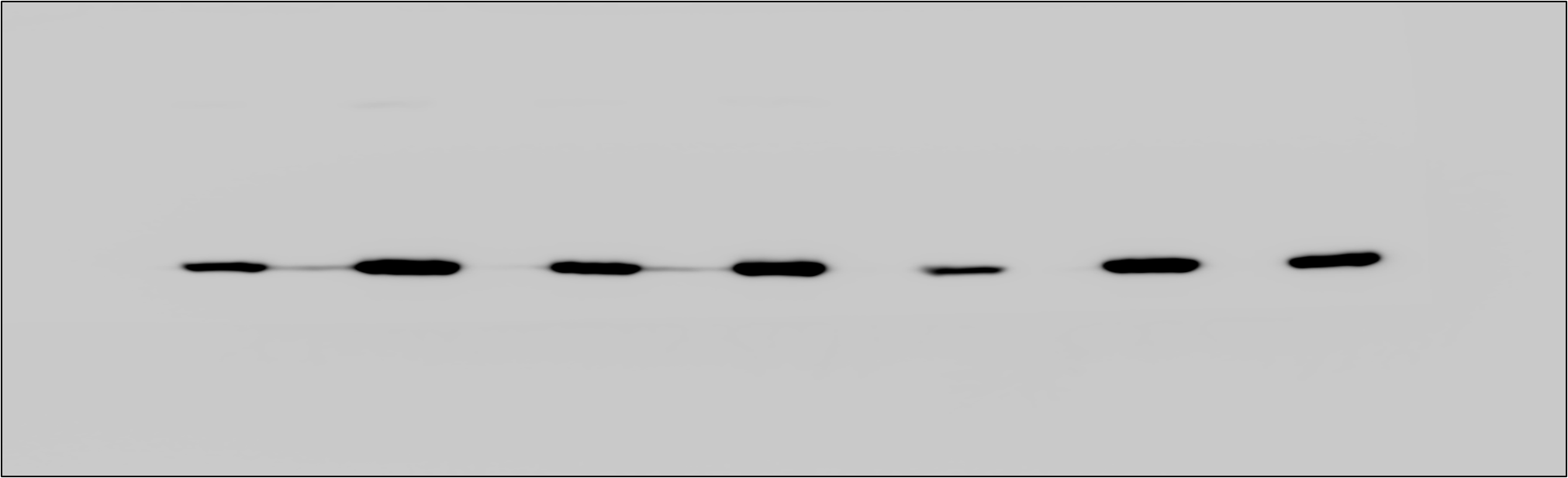

Supplement: Figure 6—source data 2. [file elife-98357-fig6-data2.zip › Figure 6-source data 2/6M-SVCV-HA-1.tif]

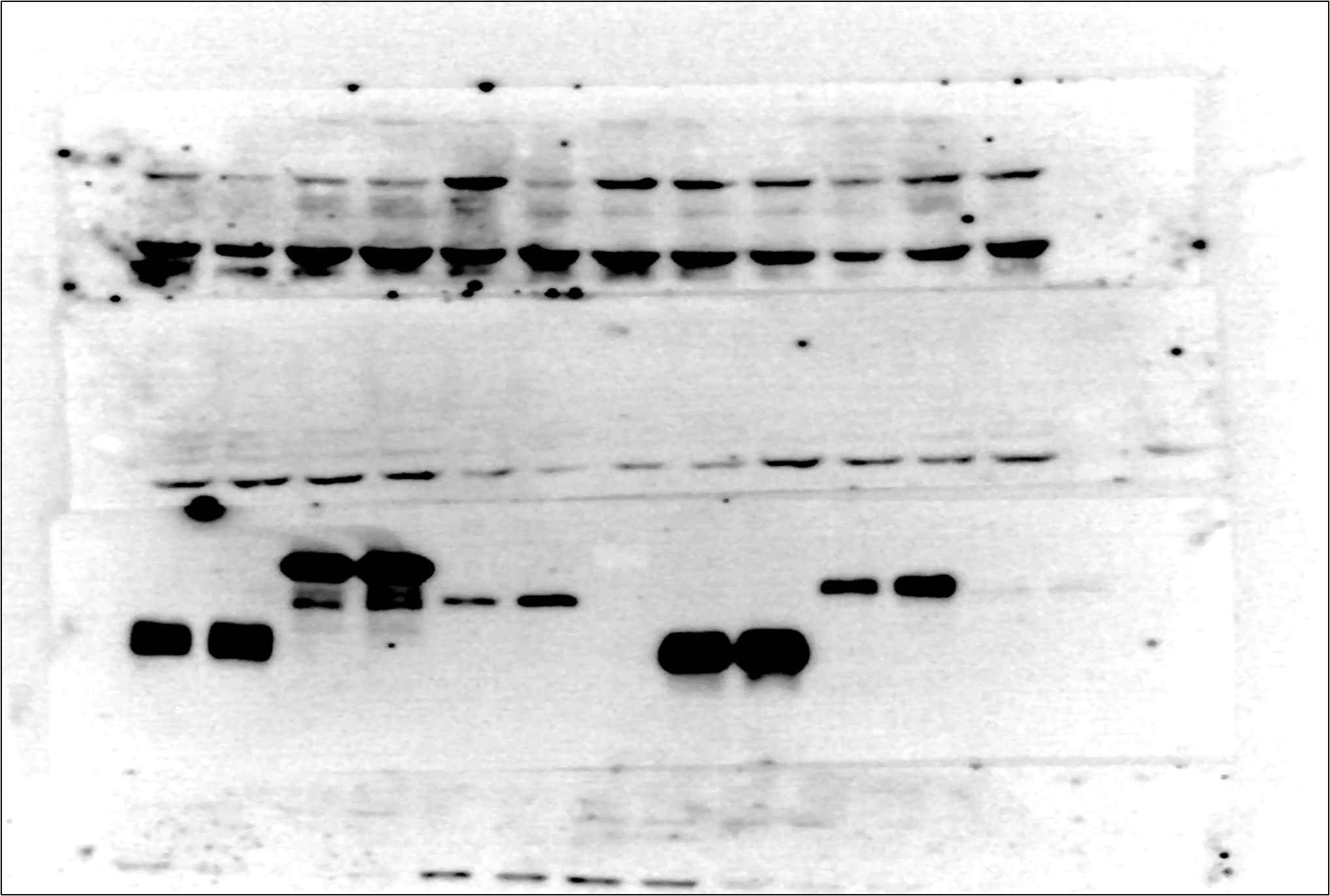

Supplement: Figure 6—source data 2. [file elife-98357-fig6-data2.zip › Figure 6-source data 2/6M-SVCV-TBK1-1.tif]

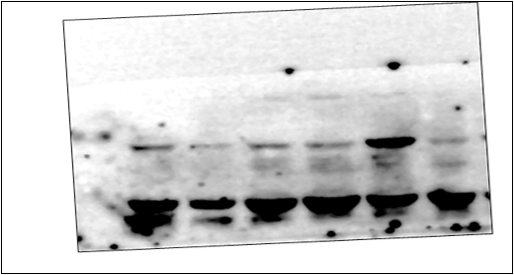

Supplement: Figure 6—source data 2. [file elife-98357-fig6-data2.zip › Figure 6-source data 2/6M-TBK1-1.tif]

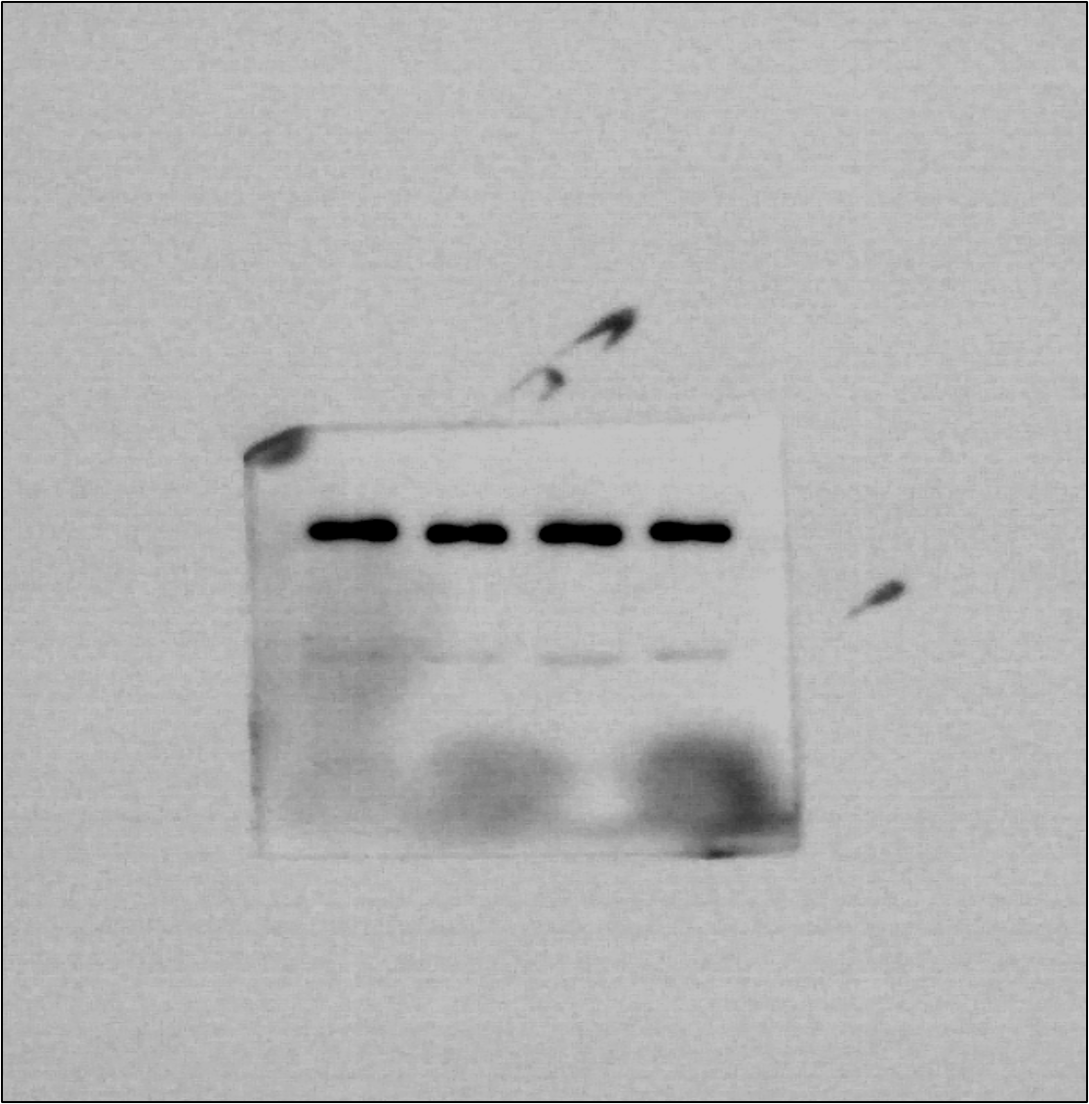

Supplement: Figure 7—source data 2. [file elife-98357-fig7-data2.zip › Figure 7-source data 2/7A-IP-Myc-1.tif]

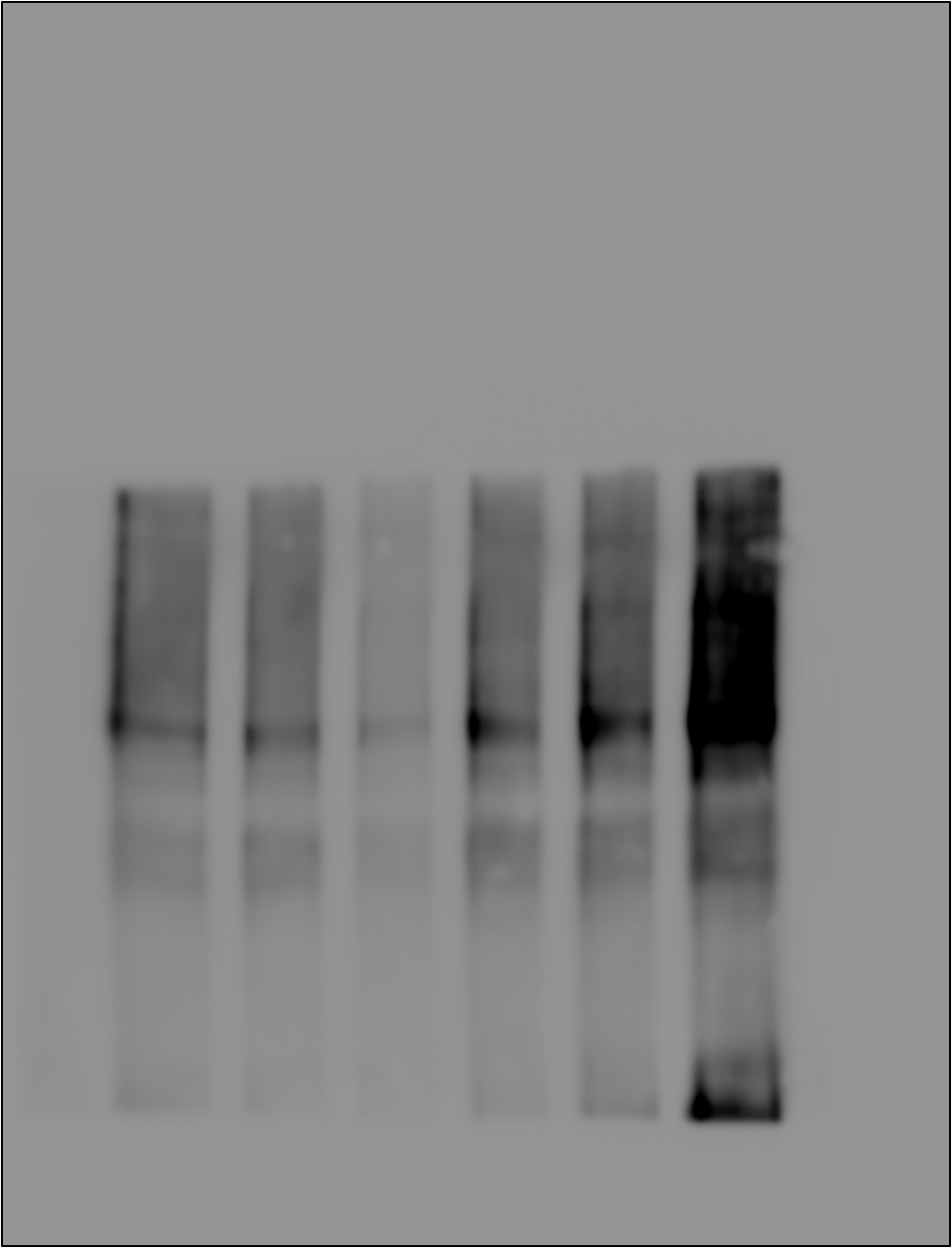

Supplement: Figure 7—source data 2. [file elife-98357-fig7-data2.zip › Figure 7-source data 2/7A-IP-TBK1-HA-Ub-1.tif]

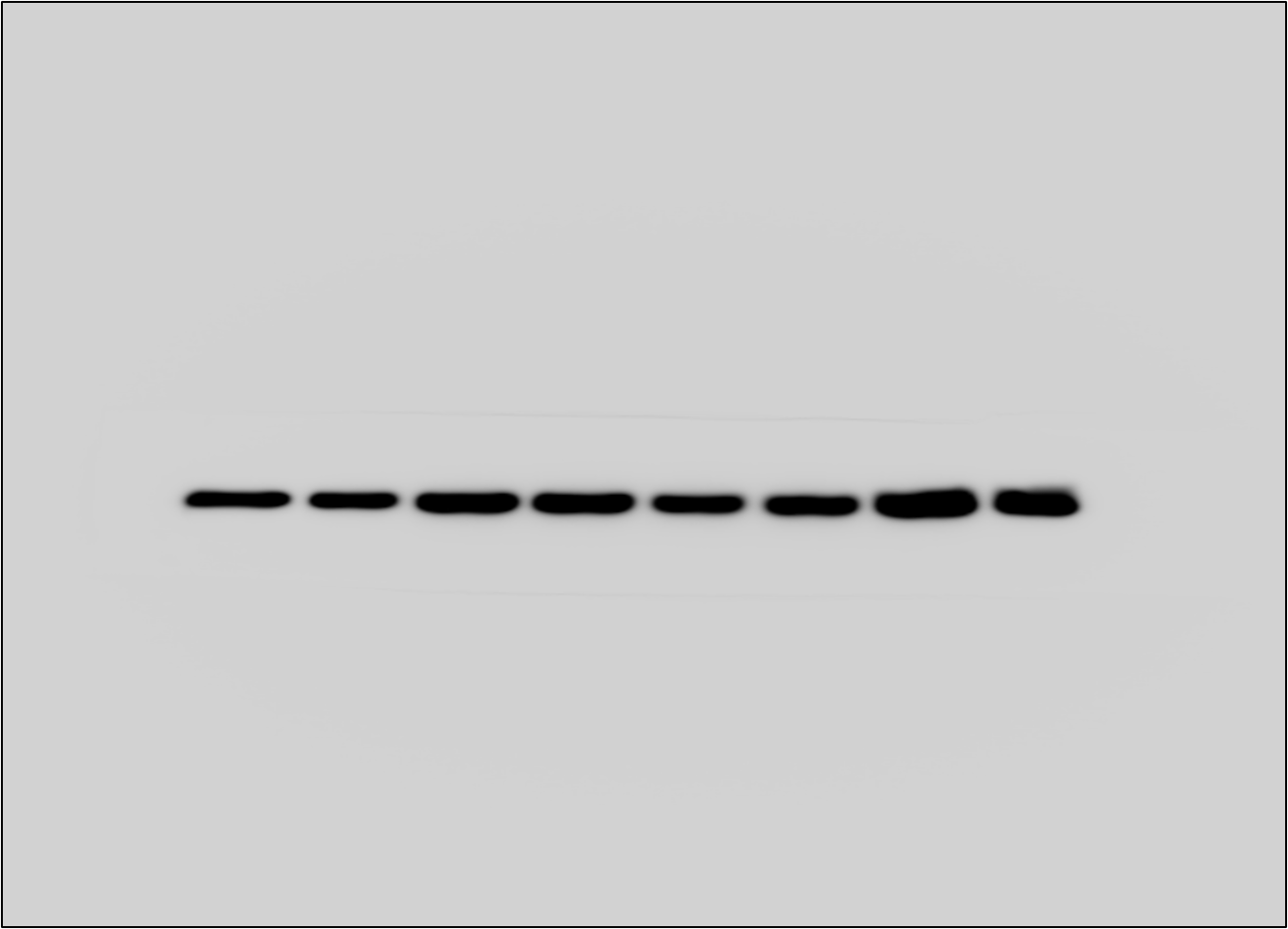

Supplement: Figure 7—source data 2. [file elife-98357-fig7-data2.zip › Figure 7-source data 2/7A-WCL-Actin-1.tif]

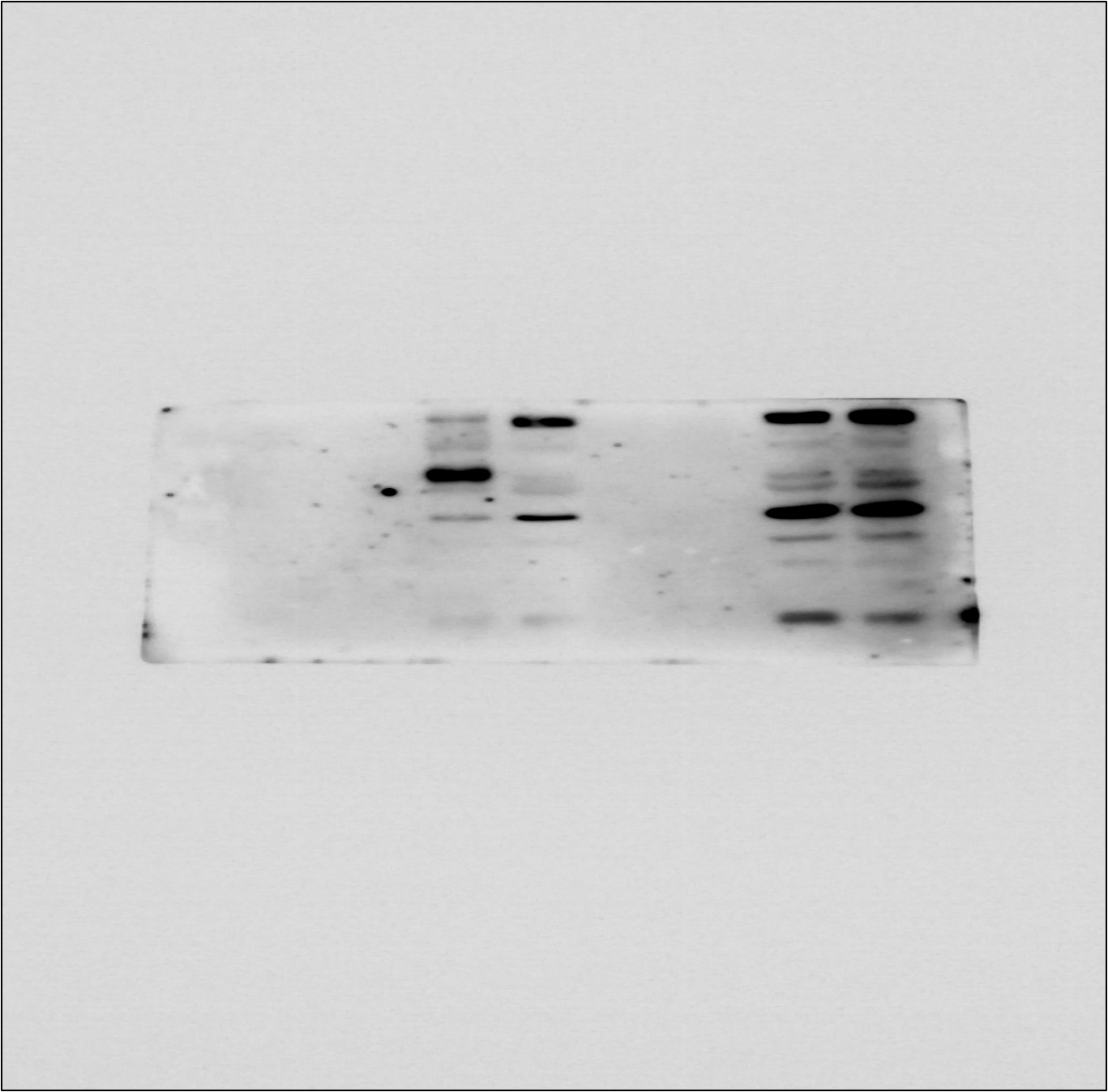

Supplement: Figure 7—source data 2. [file elife-98357-fig7-data2.zip › Figure 7-source data 2/7A-WCL-Flag-1.tif]

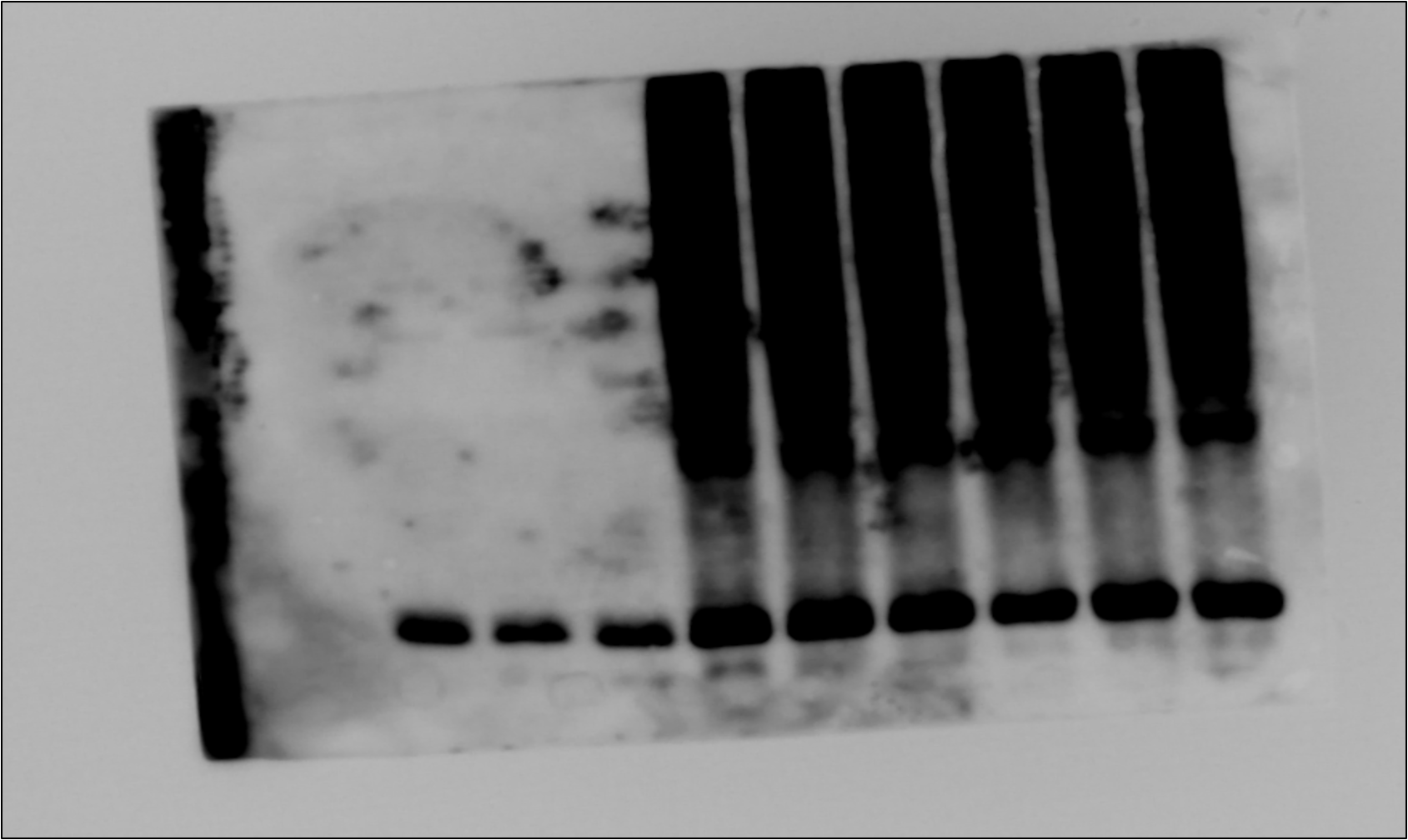

Supplement: Figure 7—source data 2. [file elife-98357-fig7-data2.zip › Figure 7-source data 2/7A-WCL-HA-1.tif]

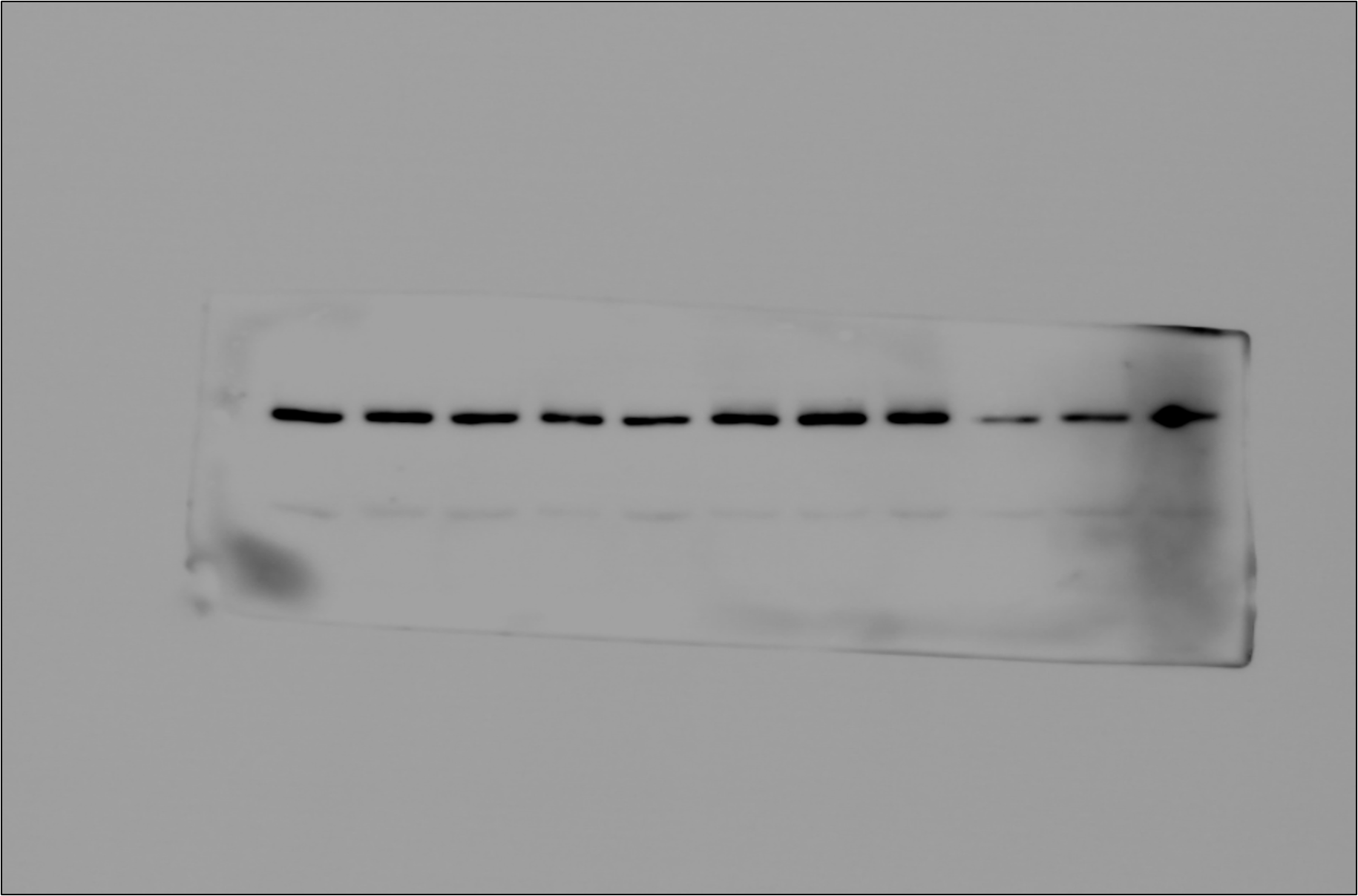

Supplement: Figure 7—source data 2. [file elife-98357-fig7-data2.zip › Figure 7-source data 2/7A-WCL-Myc-1.tif]

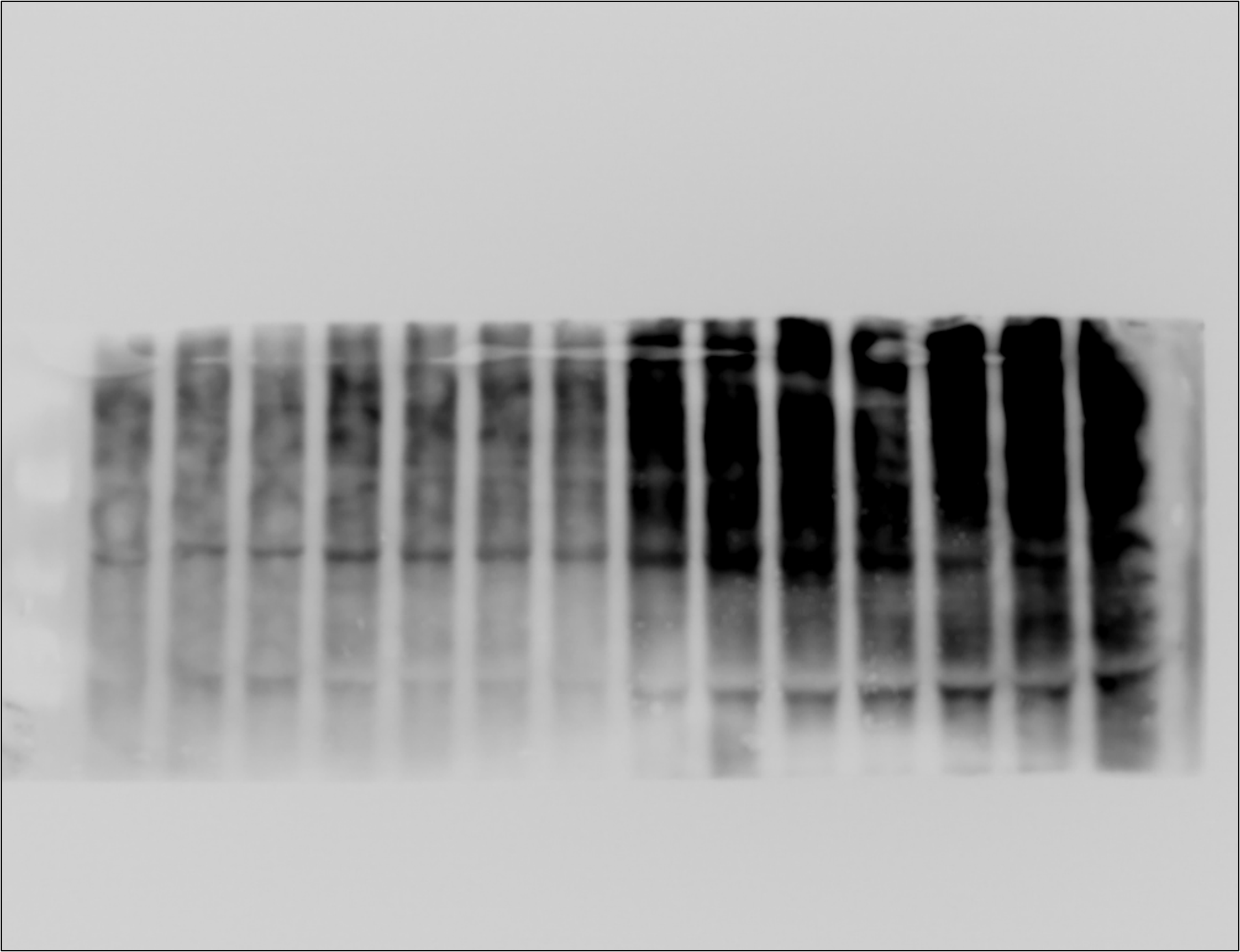

Supplement: Figure 7—source data 2. [file elife-98357-fig7-data2.zip › Figure 7-source data 2/7A-WCL-TBK1-HA-Ub-1.tif]

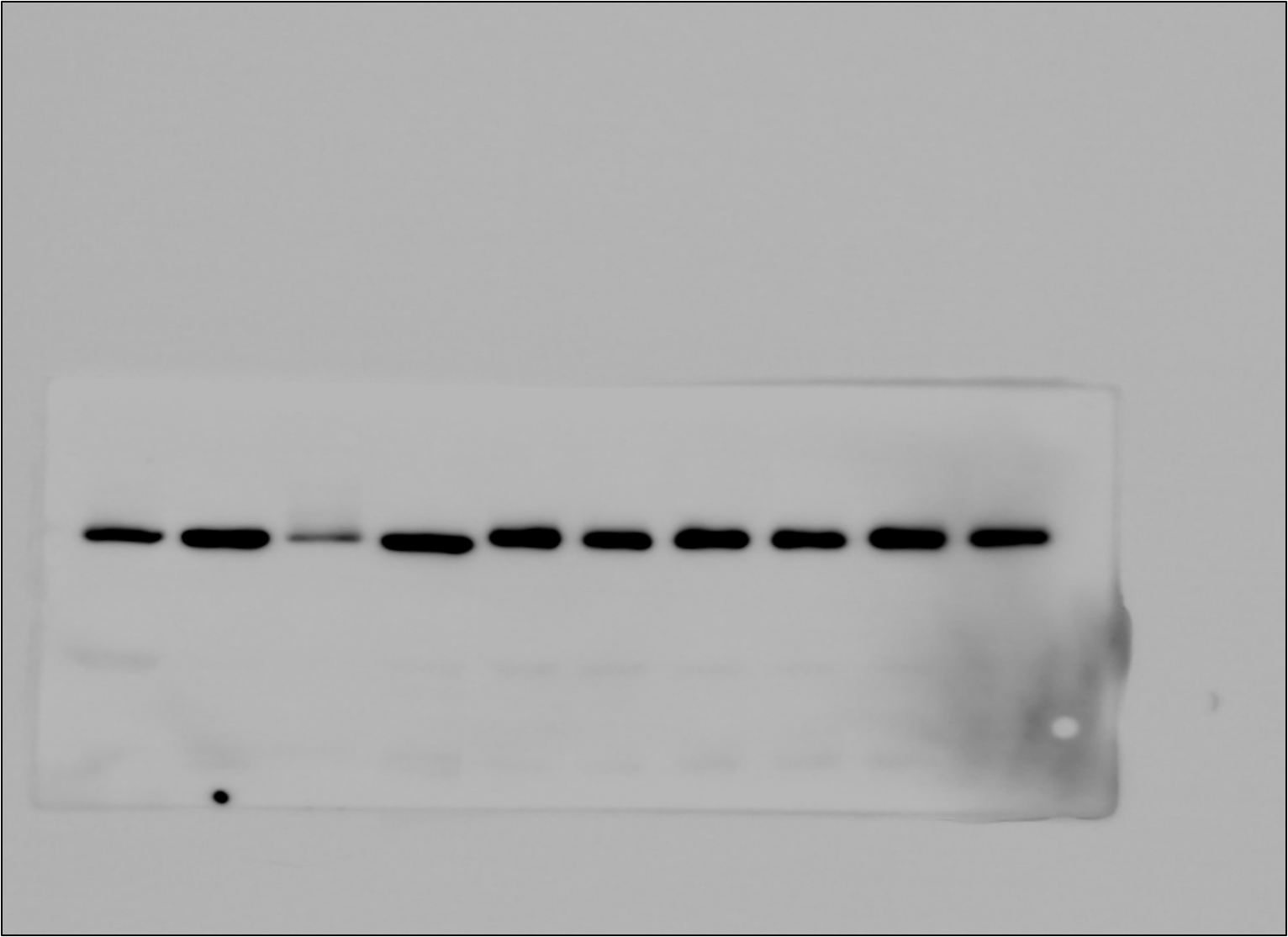

Supplement: Figure 7—source data 2. [file elife-98357-fig7-data2.zip › Figure 7-source data 2/7B-IP-Myc-1.tif]

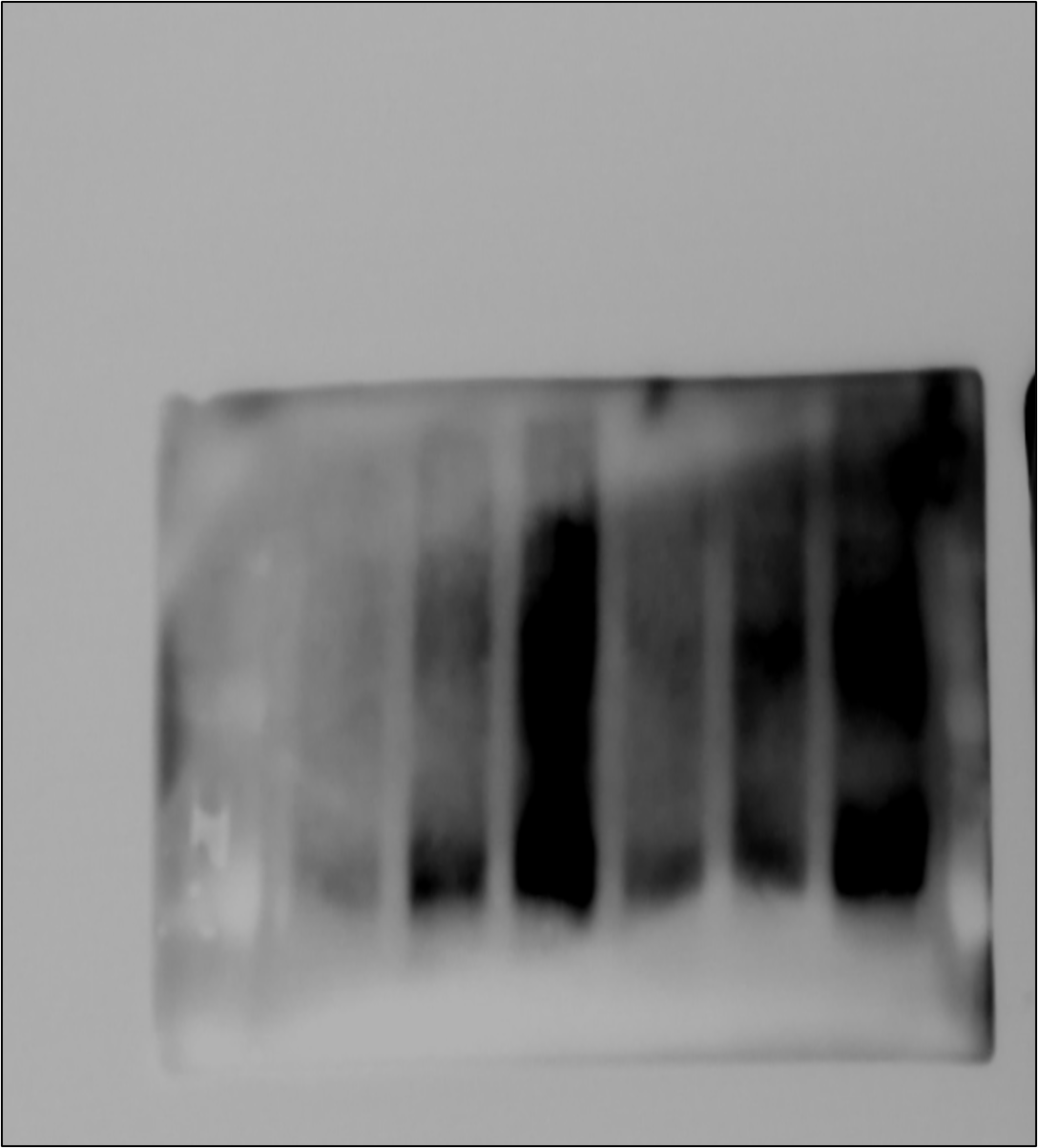

Supplement: Figure 7—source data 2. [file elife-98357-fig7-data2.zip › Figure 7-source data 2/7B-IP-TBK1-HA-Ub-1.tif]

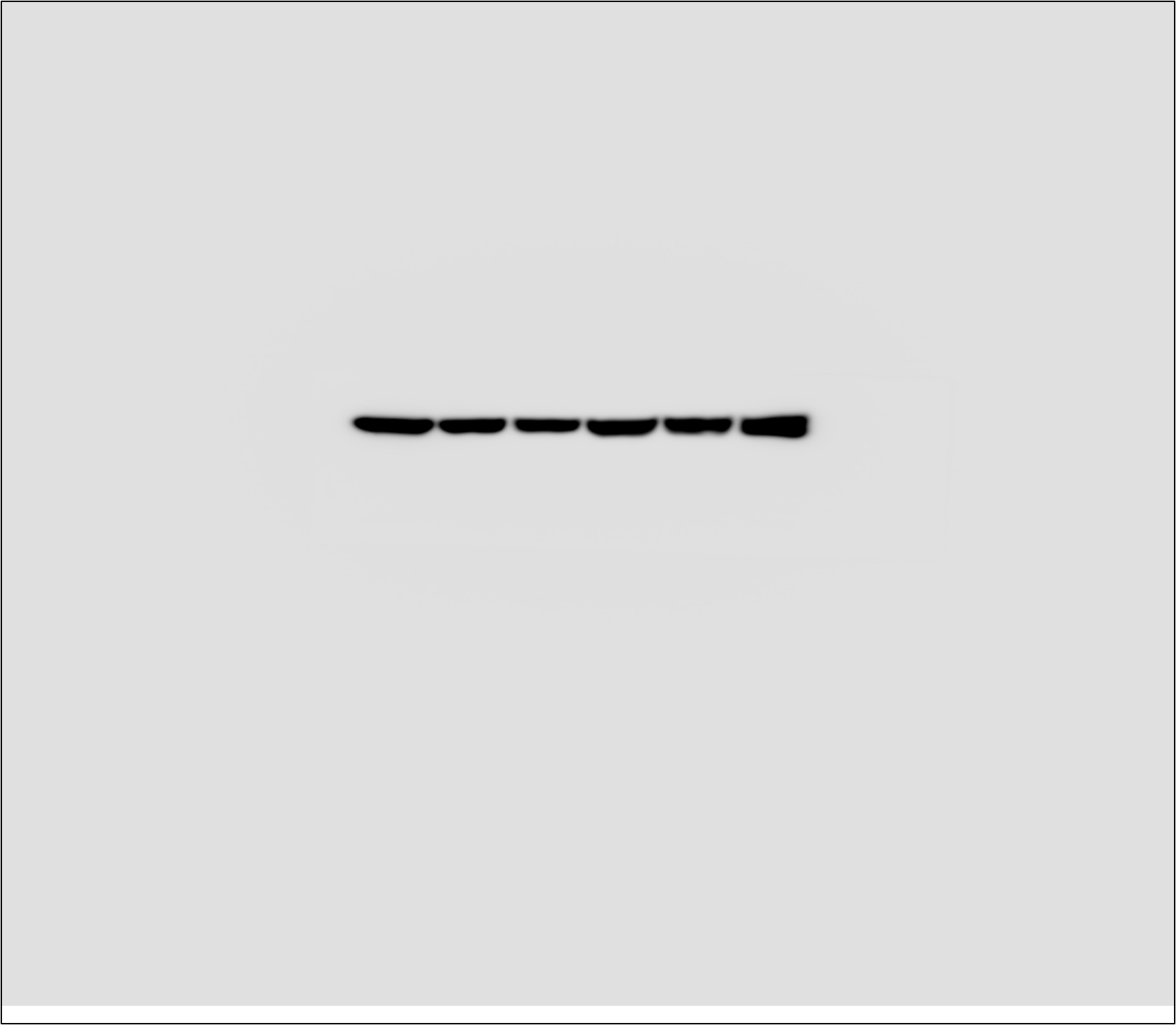

Supplement: Figure 7—source data 2. [file elife-98357-fig7-data2.zip › Figure 7-source data 2/7B-WCL-Actin-1.tif]

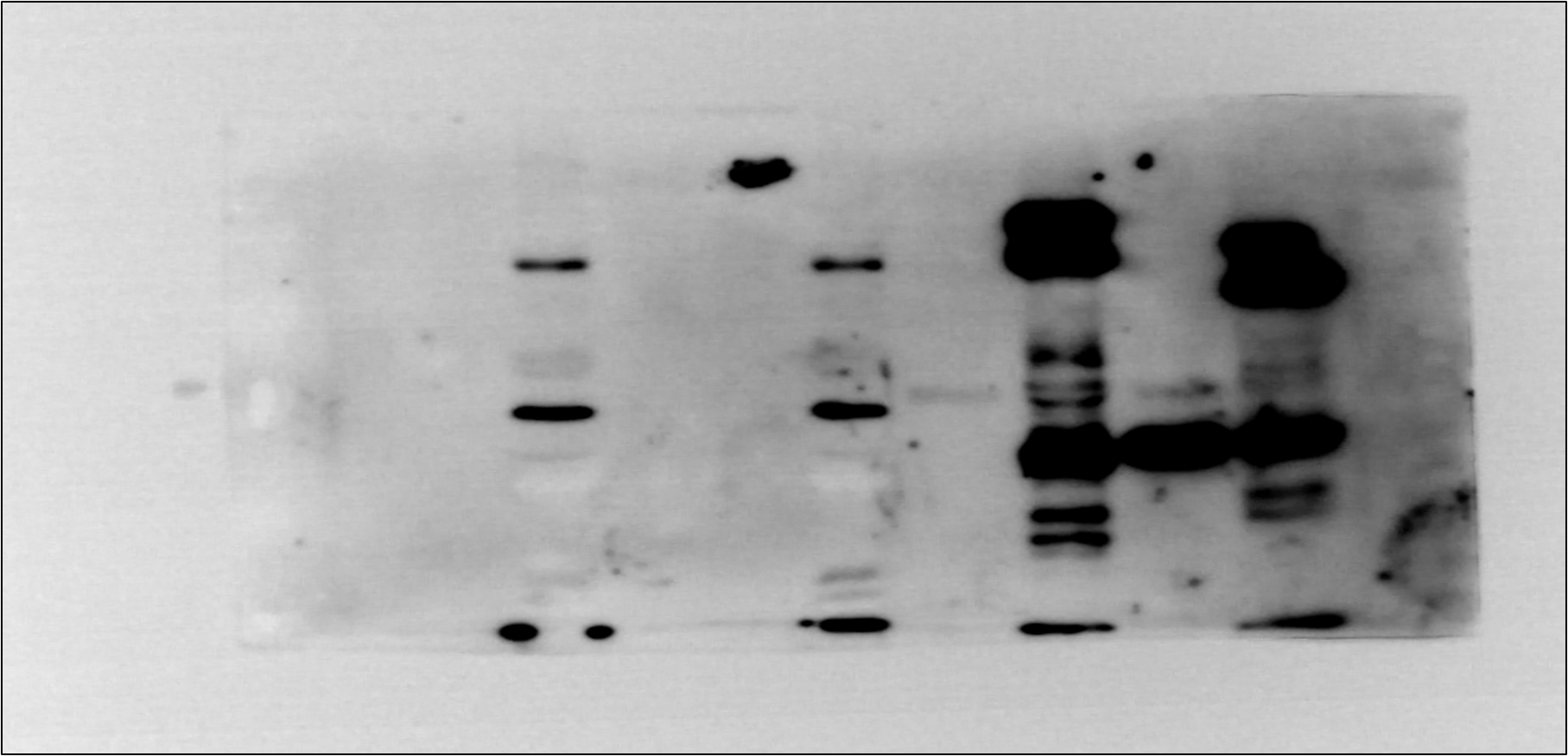

Supplement: Figure 7—source data 2. [file elife-98357-fig7-data2.zip › Figure 7-source data 2/7B-WCL-Flag-1.tif]

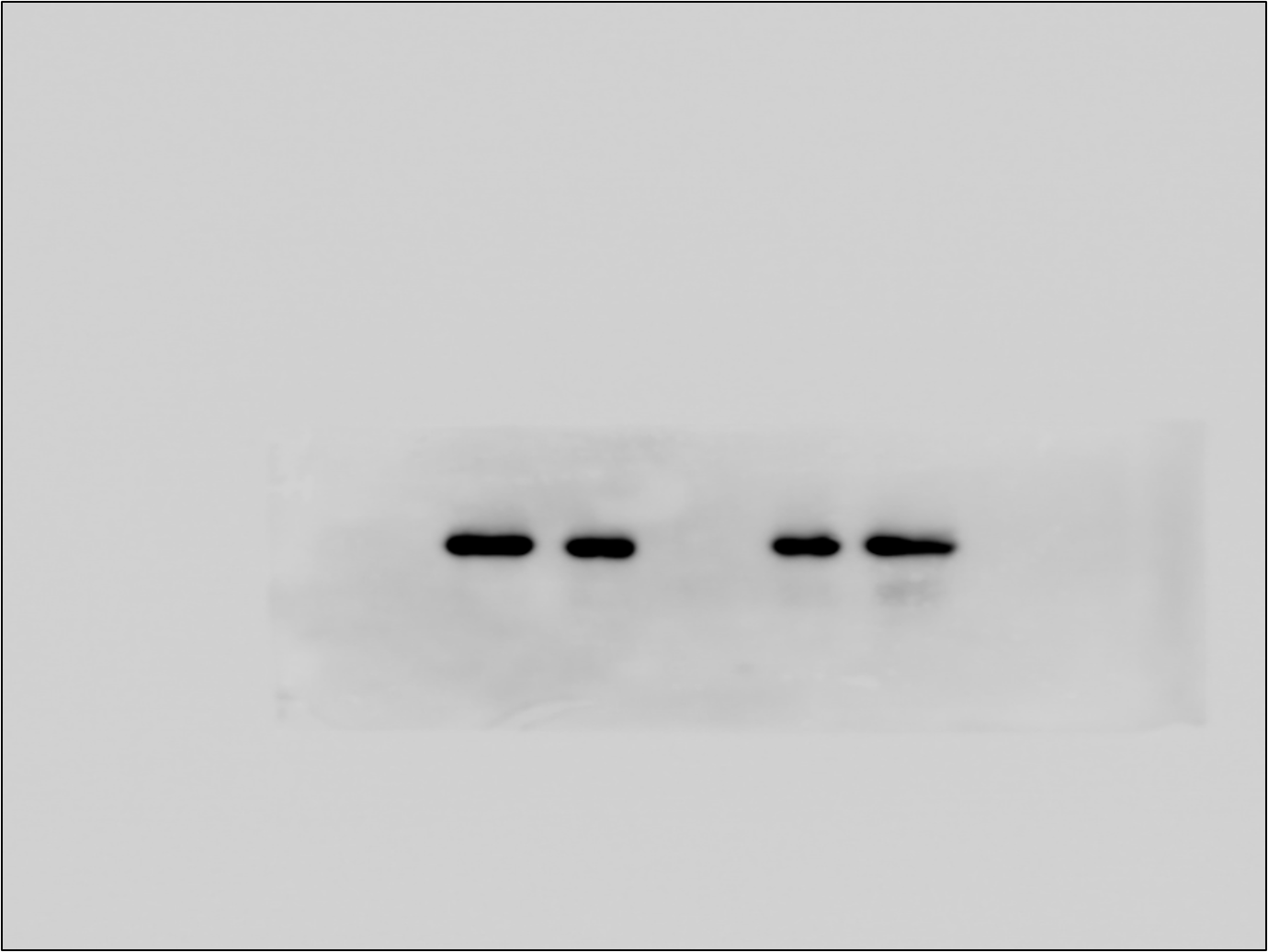

Supplement: Figure 7—source data 2. [file elife-98357-fig7-data2.zip › Figure 7-source data 2/7B-WCL-HA-1.tif]

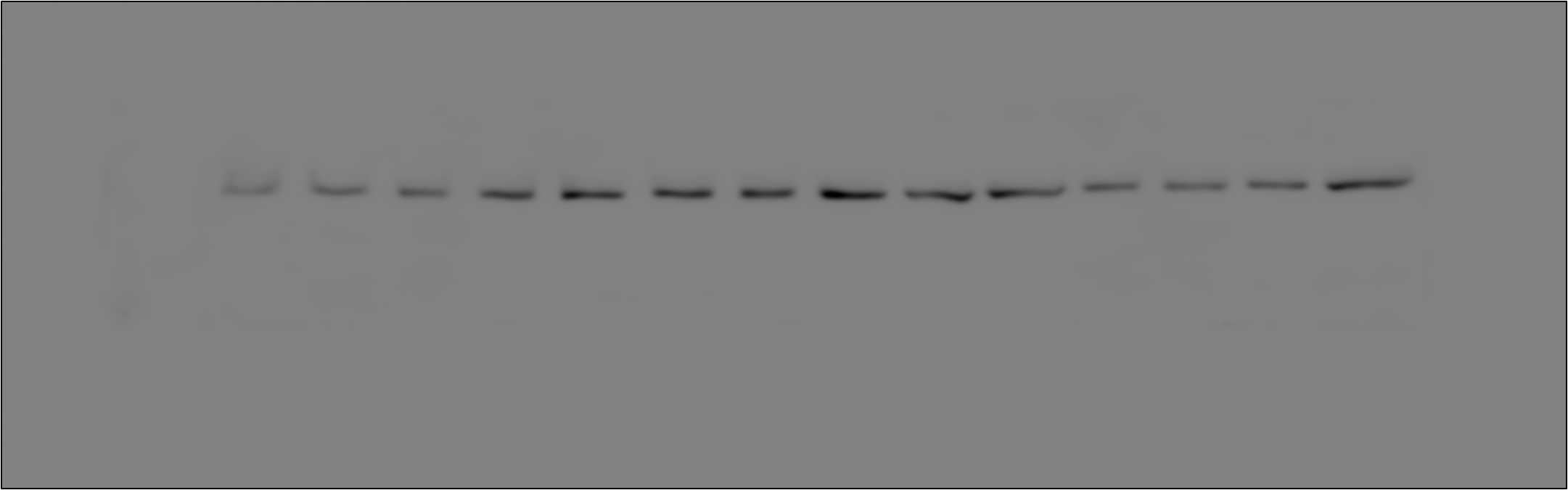

Supplement: Figure 7—source data 2. [file elife-98357-fig7-data2.zip › Figure 7-source data 2/7B-WCL-Myc-1.tif]

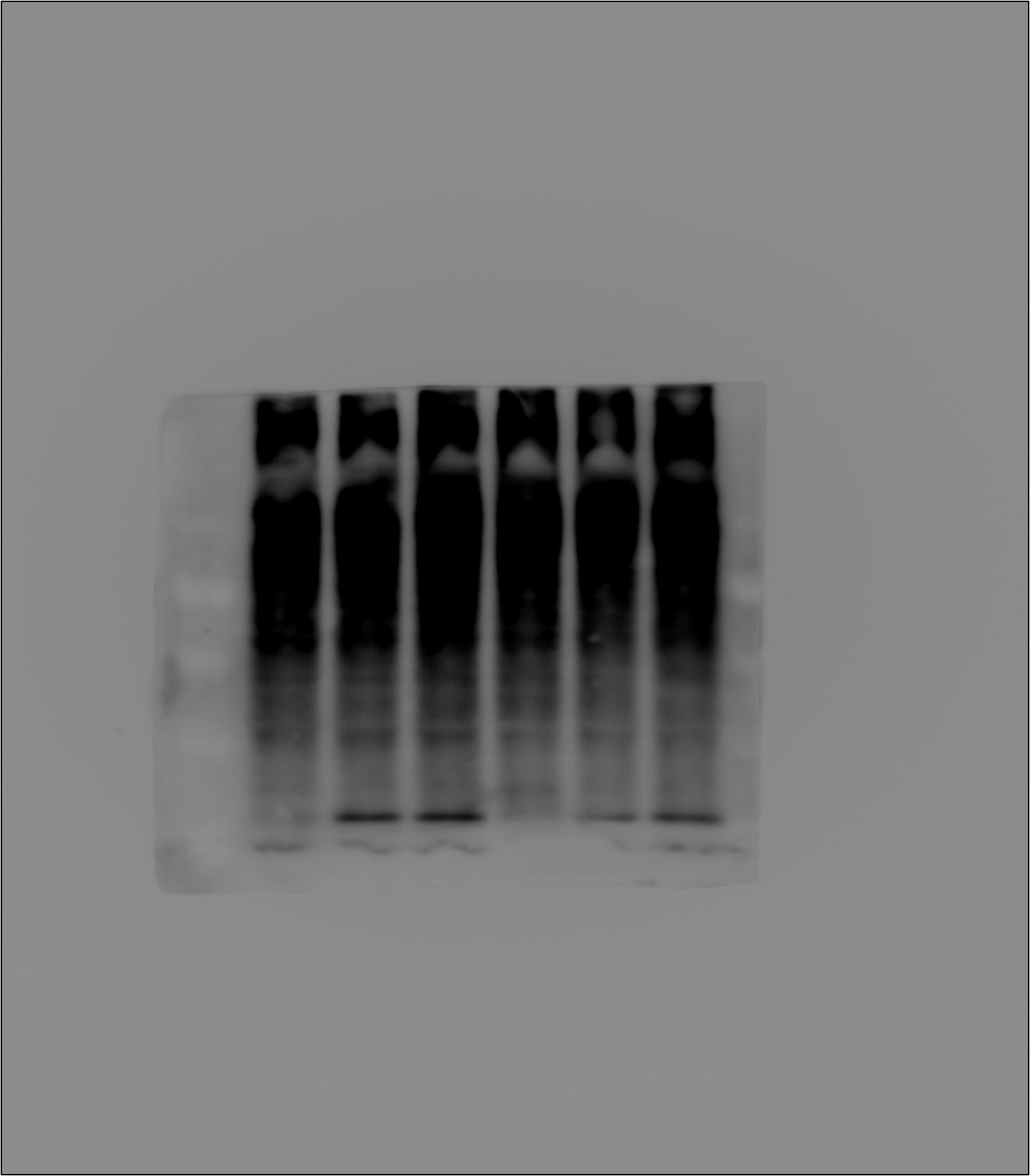

Supplement: Figure 7—source data 2. [file elife-98357-fig7-data2.zip › Figure 7-source data 2/7B-WCL-TBK1-HA-Ub-1.tif]

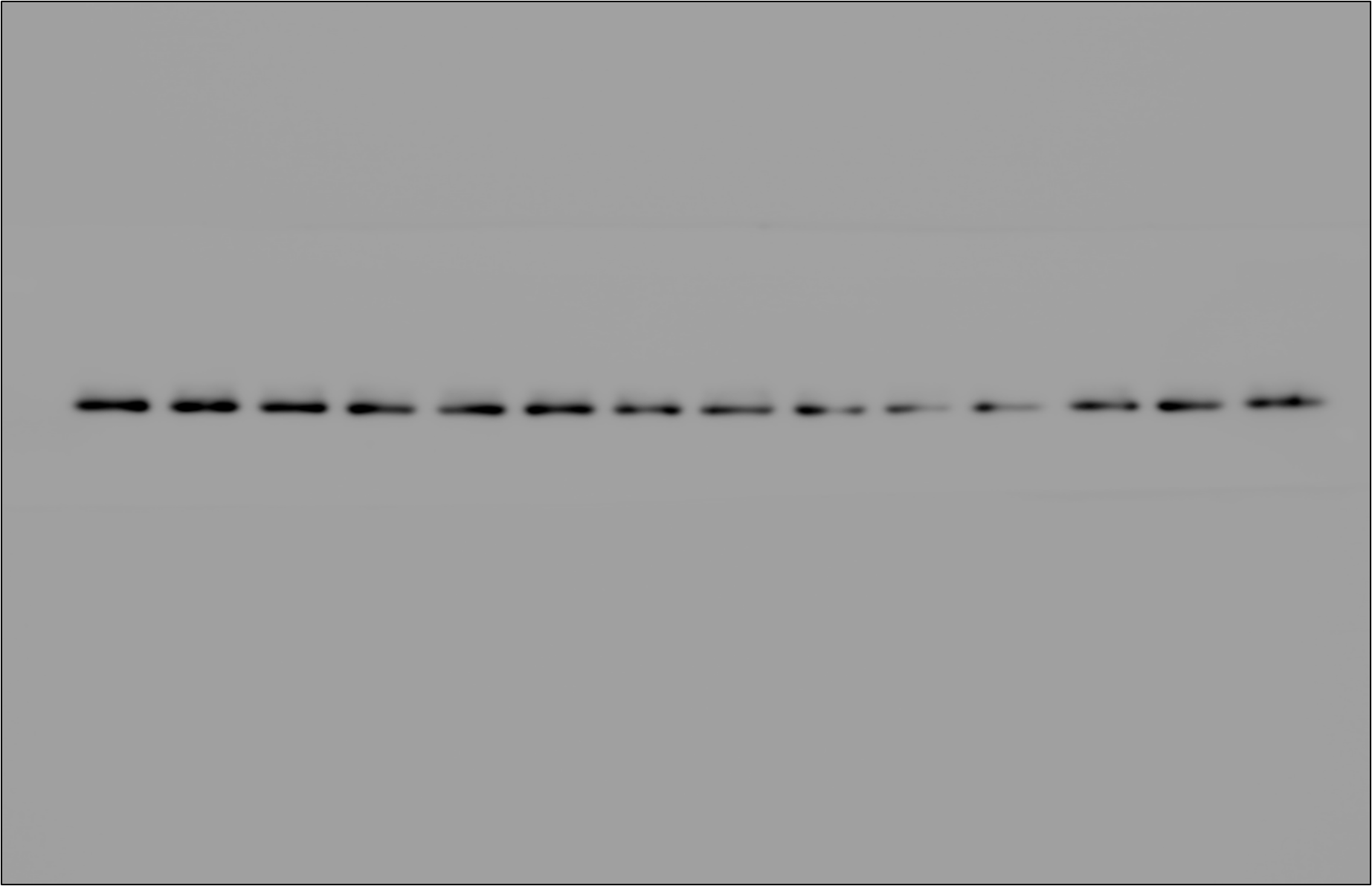

Supplement: Figure 7—source data 2. [file elife-98357-fig7-data2.zip › Figure 7-source data 2/7C-IP-Myc-1.tif]

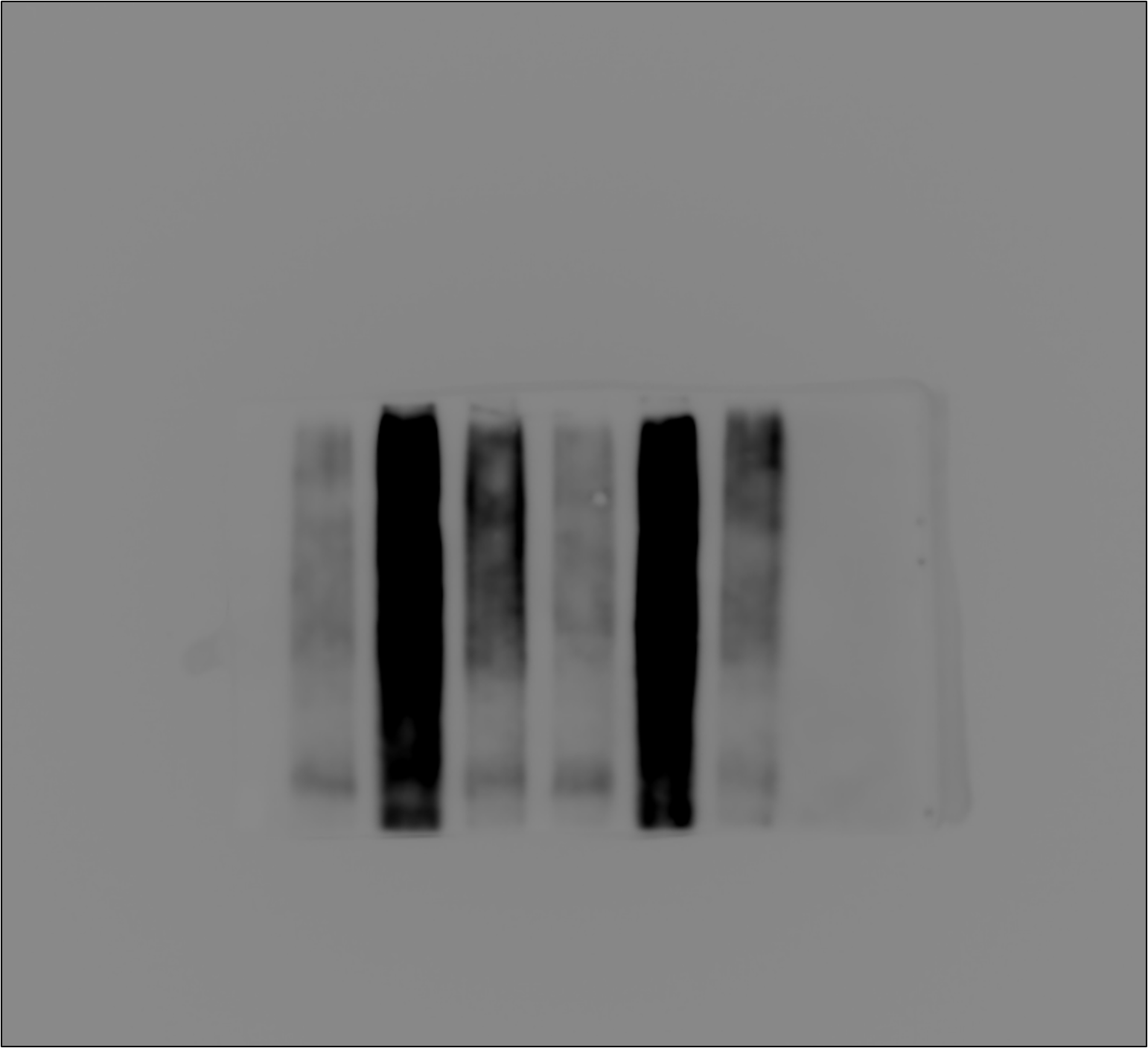

Supplement: Figure 7—source data 2. [file elife-98357-fig7-data2.zip › Figure 7-source data 2/7C-IP-TBK1-HA-Ub-1.tif]

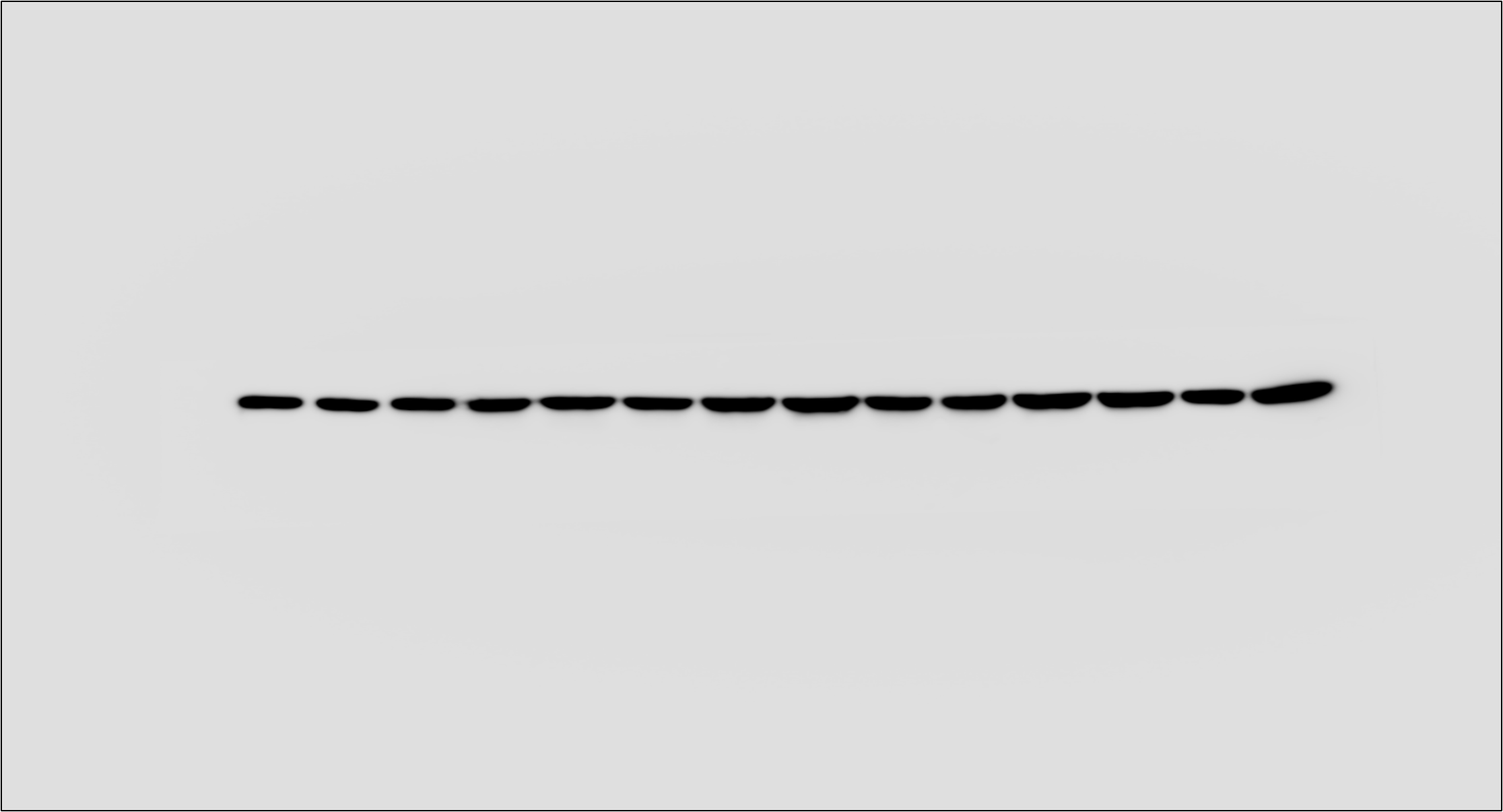

Supplement: Figure 7—source data 2. [file elife-98357-fig7-data2.zip › Figure 7-source data 2/7C-WCL-Actin-1.tif]

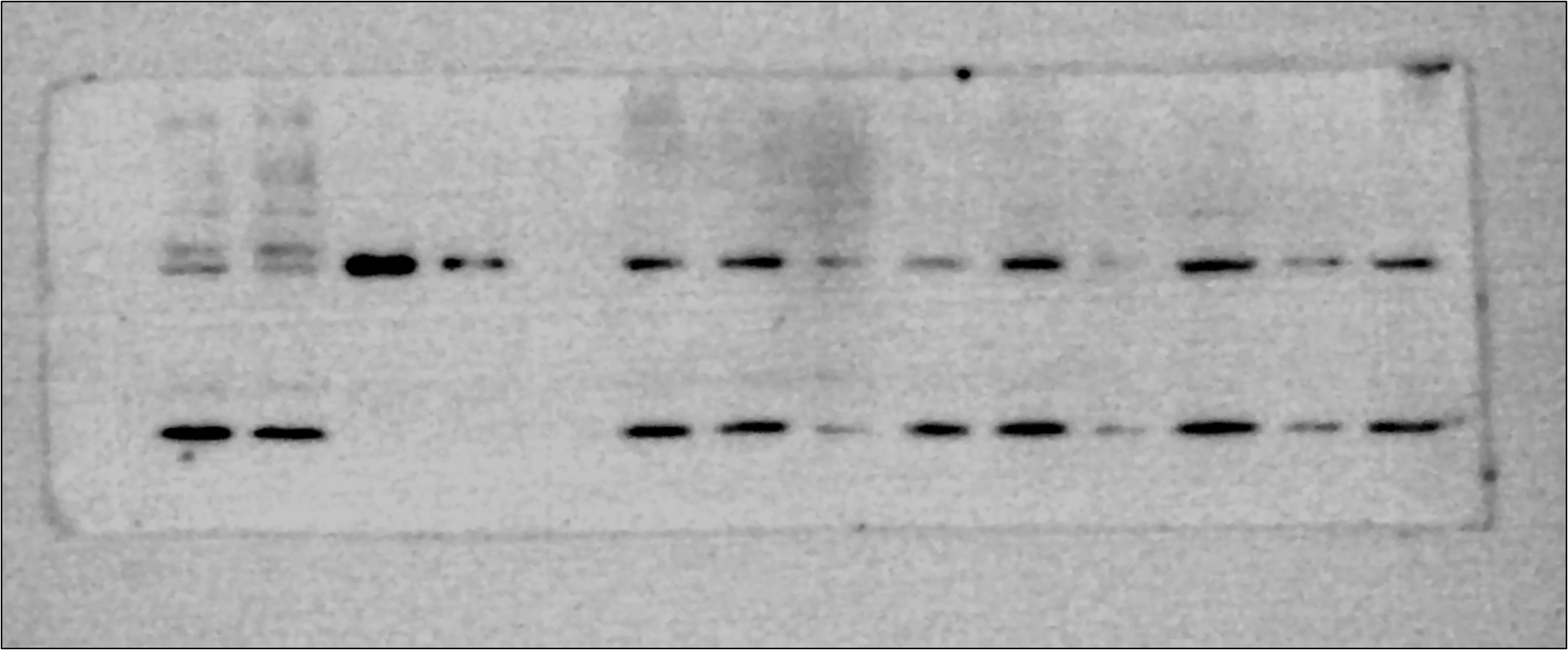

Supplement: Figure 7—source data 2. [file elife-98357-fig7-data2.zip › Figure 7-source data 2/7C-WCL-Dtx4-1.tif]

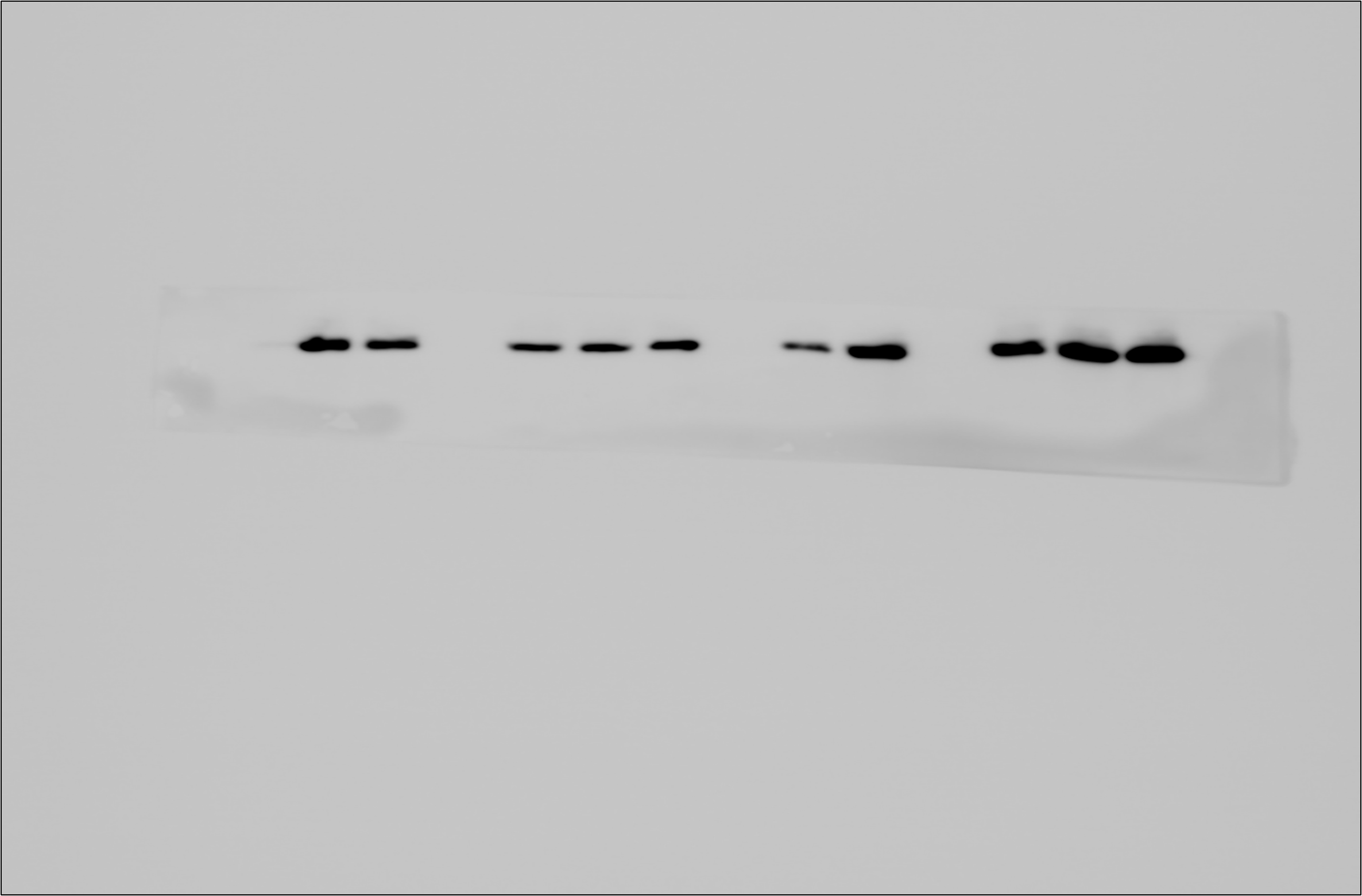

Supplement: Figure 7—source data 2. [file elife-98357-fig7-data2.zip › Figure 7-source data 2/7C-WCL-HA-1.tif]

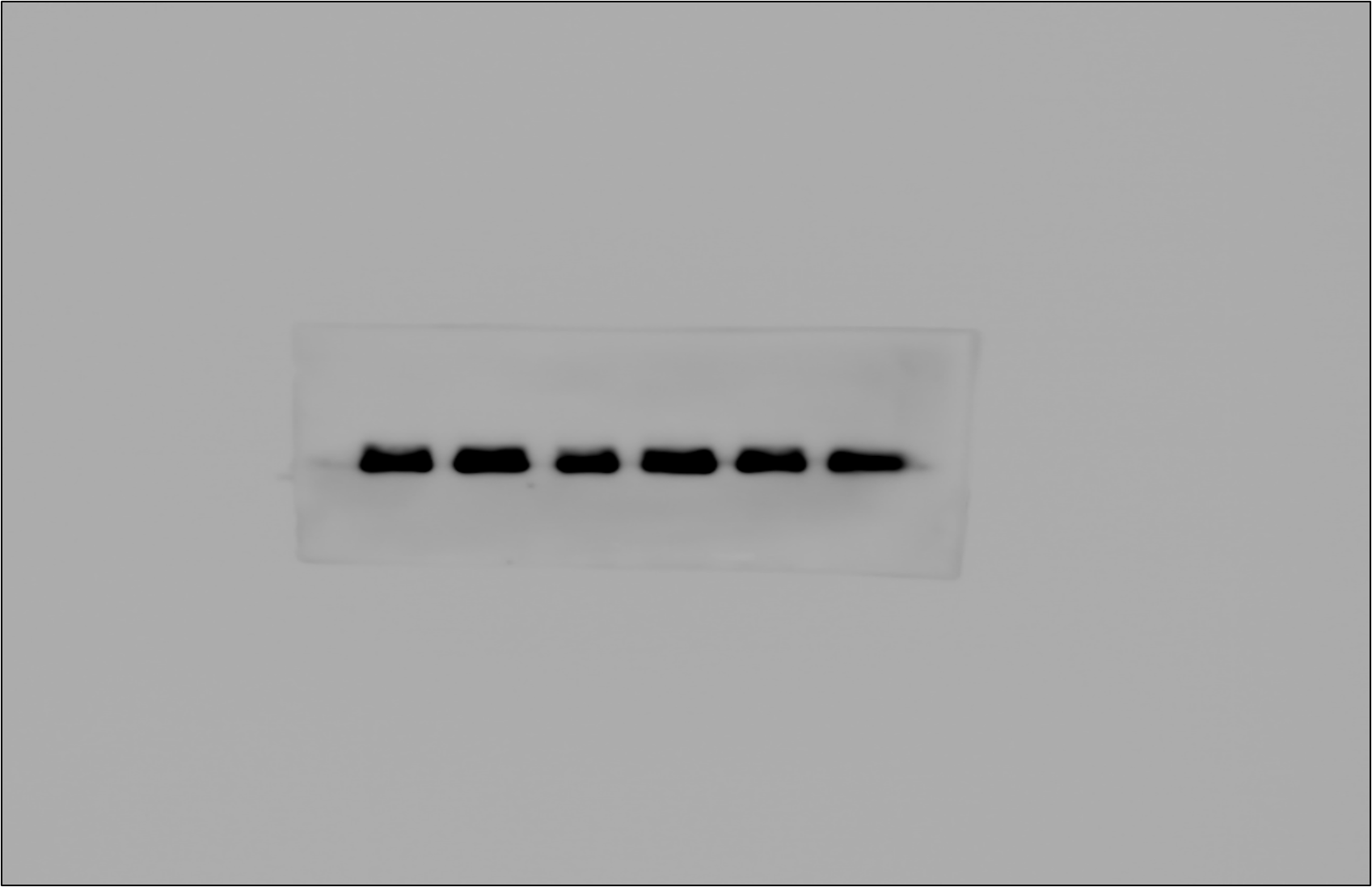

Supplement: Figure 7—source data 2. [file elife-98357-fig7-data2.zip › Figure 7-source data 2/7C-WCL-Myc-1.tif]

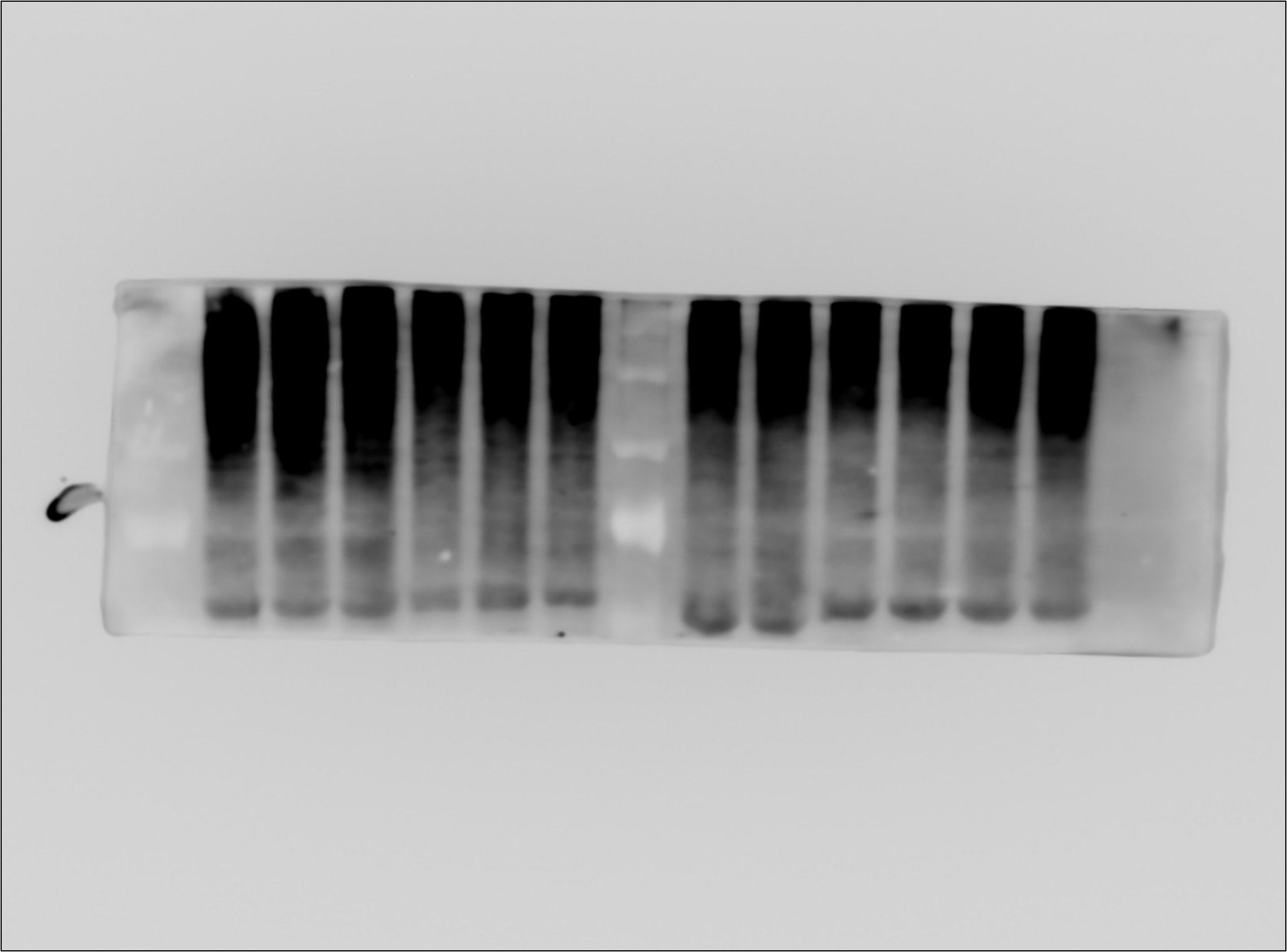

Supplement: Figure 7—source data 2. [file elife-98357-fig7-data2.zip › Figure 7-source data 2/7C-WCL-TBK1-HA-Ub-1.tif]

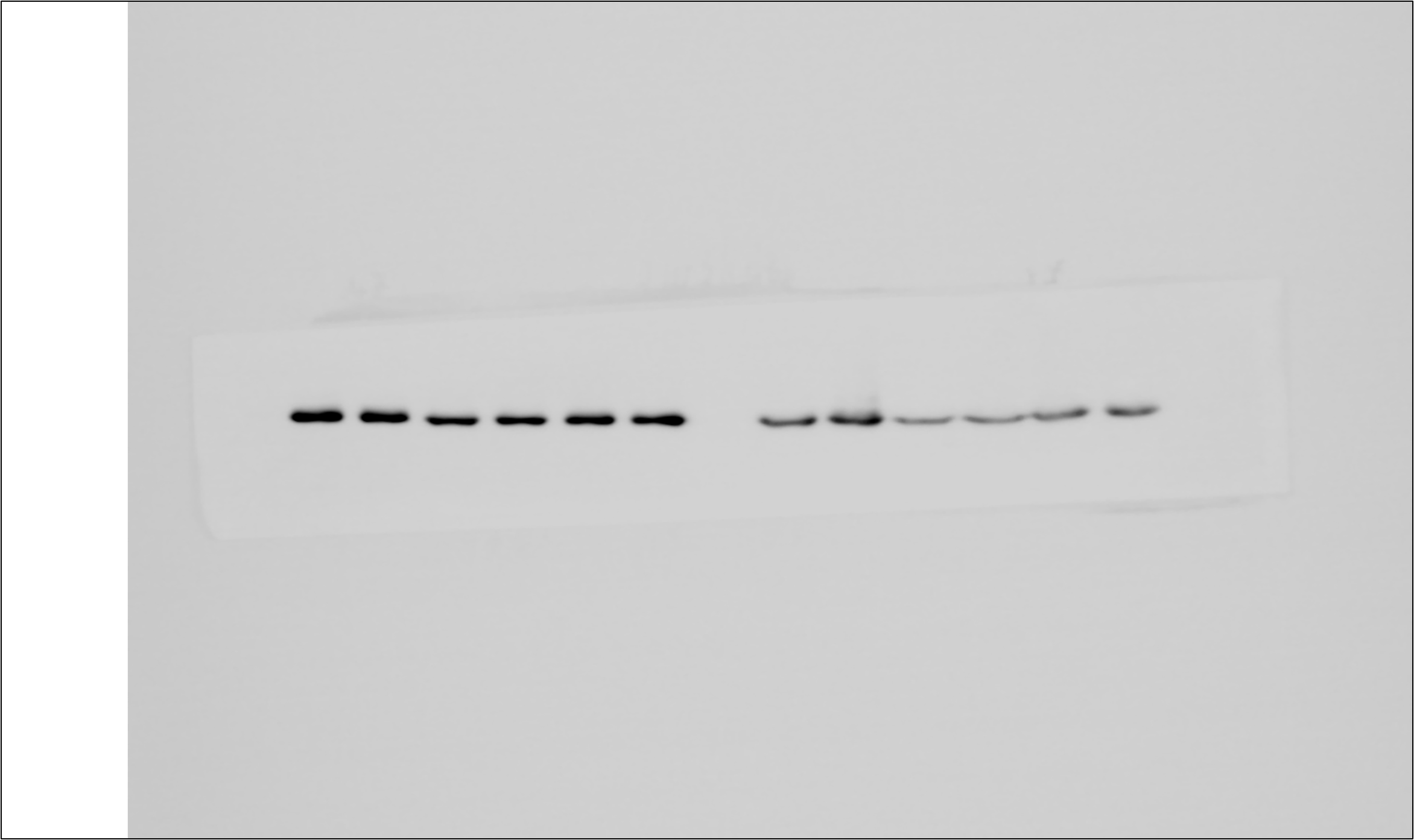

Supplement: Figure 7—source data 2. [file elife-98357-fig7-data2.zip › Figure 7-source data 2/7E-IP-Myc-1.tif]

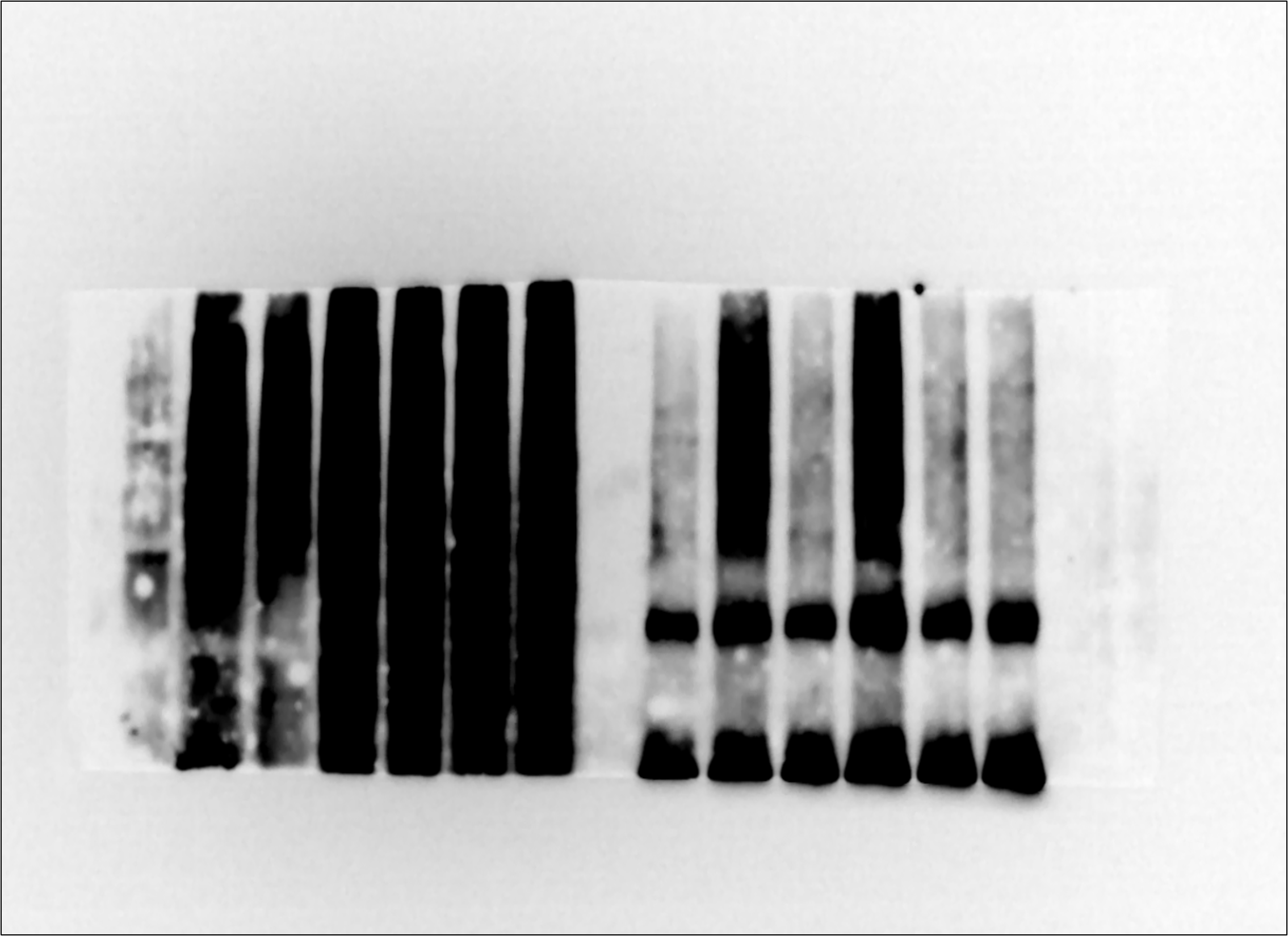

Supplement: Figure 7—source data 2. [file elife-98357-fig7-data2.zip › Figure 7-source data 2/7E-IP-TBK1-HA-Ub-1.tif]

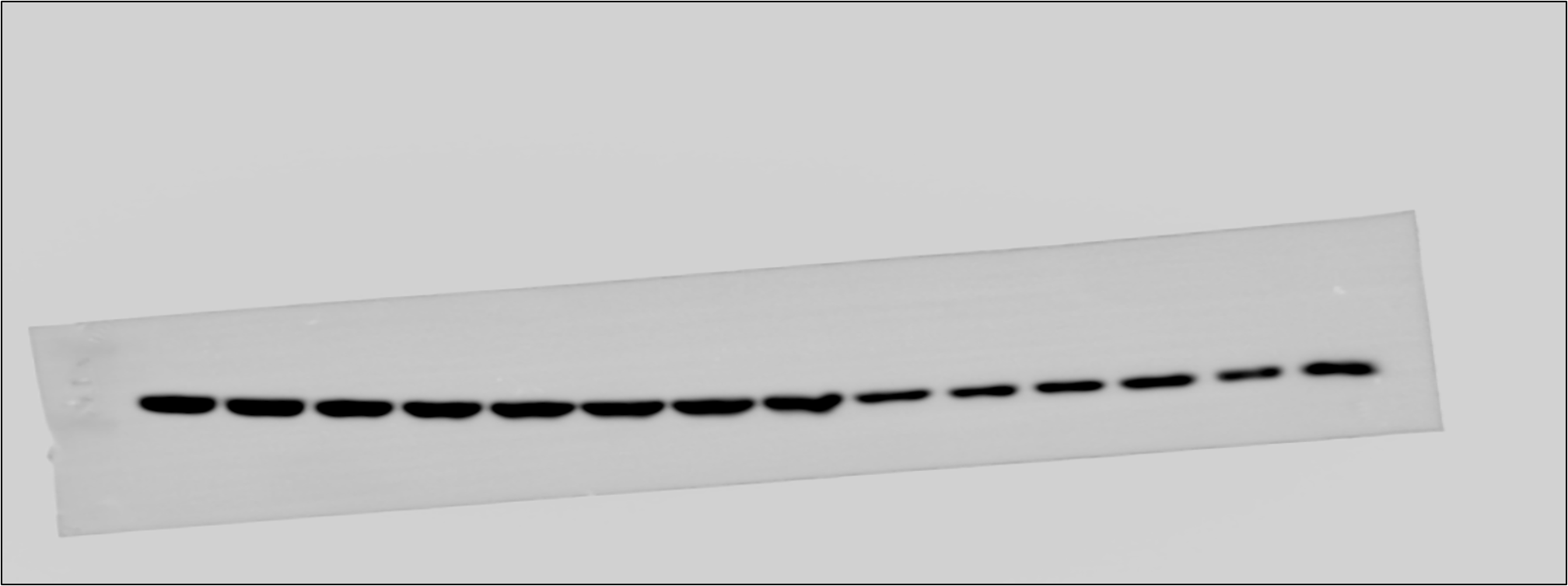

Supplement: Figure 7—source data 2. [file elife-98357-fig7-data2.zip › Figure 7-source data 2/7E-WCL-Actin-1.tif]

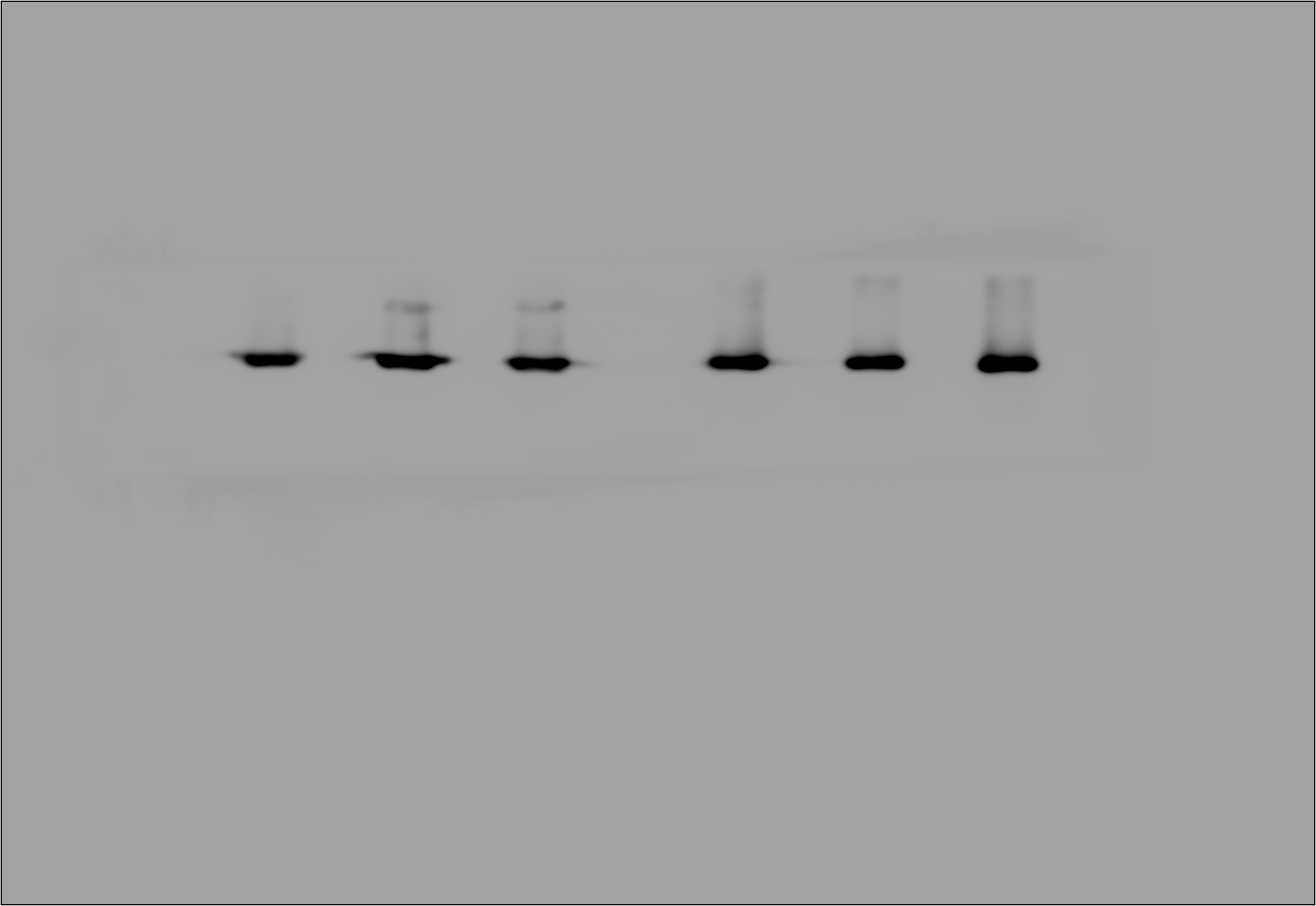

Supplement: Figure 7—source data 2. [file elife-98357-fig7-data2.zip › Figure 7-source data 2/7E-WCL-HA-1.tif]
