# Supplementary material for: Fish CDK2 recruits Dtx4 to degrade TBK1 through ubiquitination in the antiviral response
Source: eLife. 2026 Jan 14;13:RP98357. doi: 10.7554/eLife.98357 (PMC12803515; doi:10.7554/eLife.98357)
Supplement: Figure 7—source data 1. [file elife-98357-fig7-data1.zip › Figure 7-source data 1/Figure 7-source data.pdf]

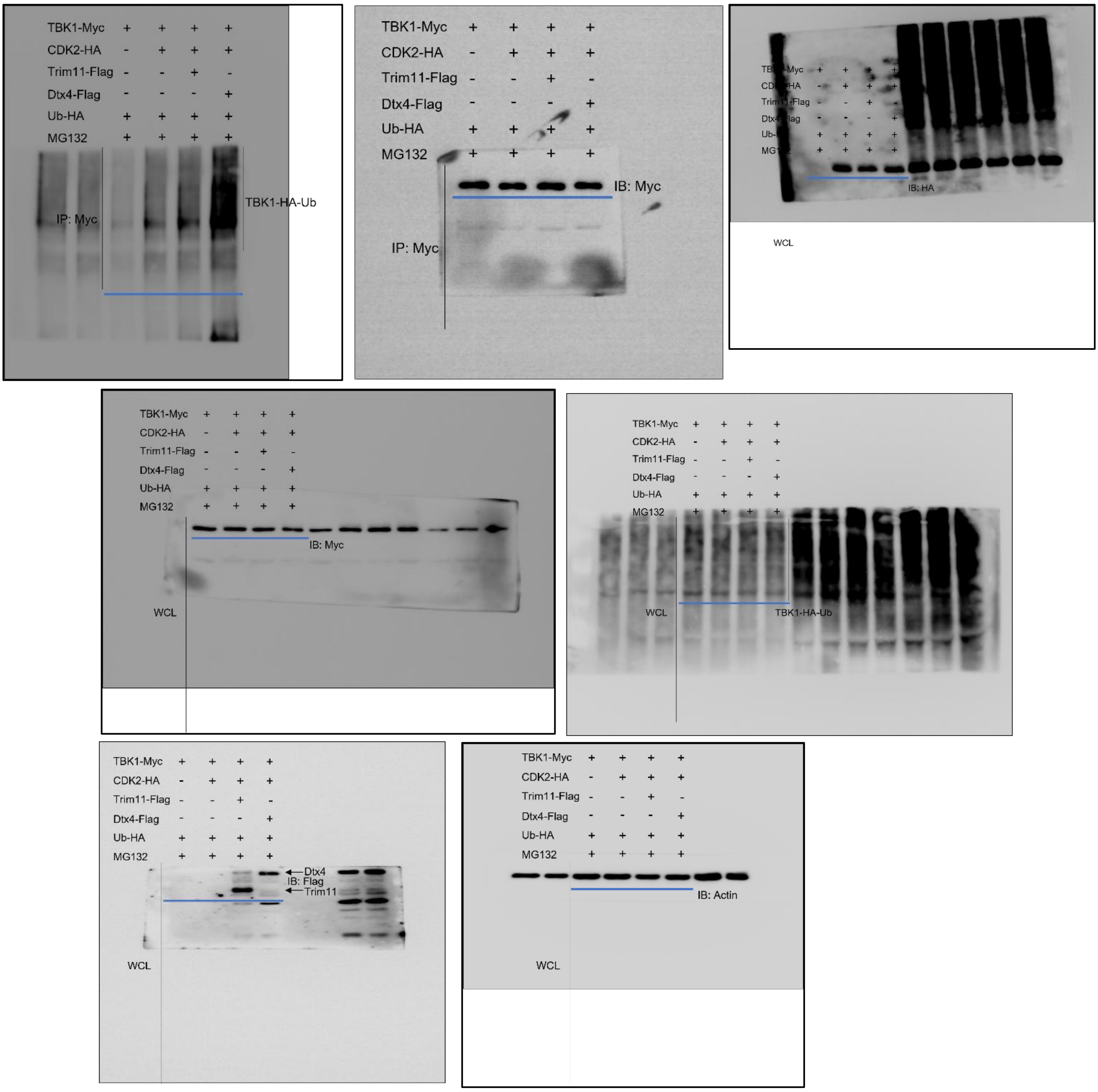

Figure 7, Source Data 1. Original membranes corresponding to Figure 7, panel A. Each membrane is labelled with the relevant information. The blue lines indicate the corresponding bands.

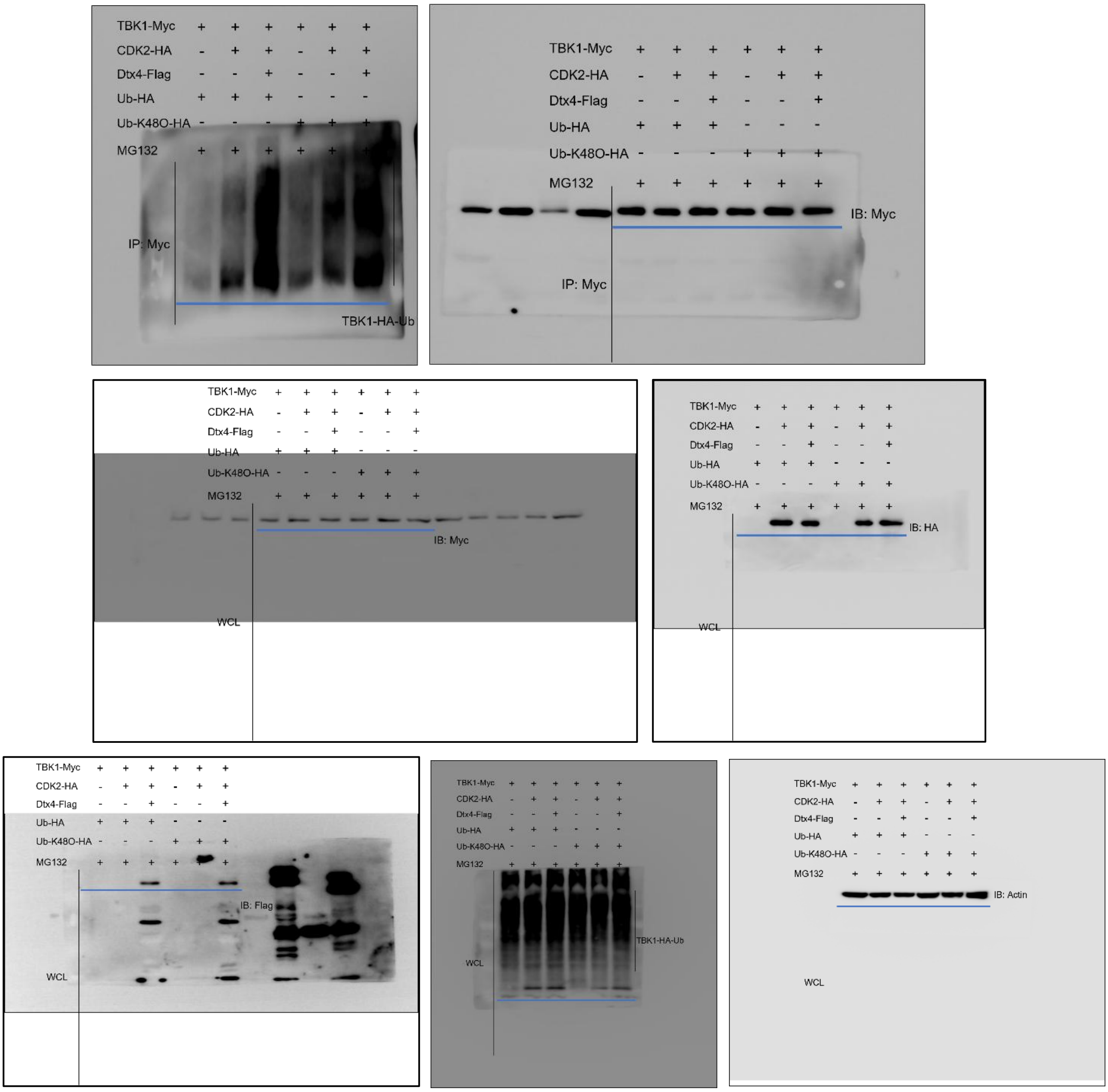

Figure 7, Source Data 1. Original membranes corresponding to Figure 7, panel B. Each membrane is labelled with the relevant information. The blue lines indicate the corresponding bands.

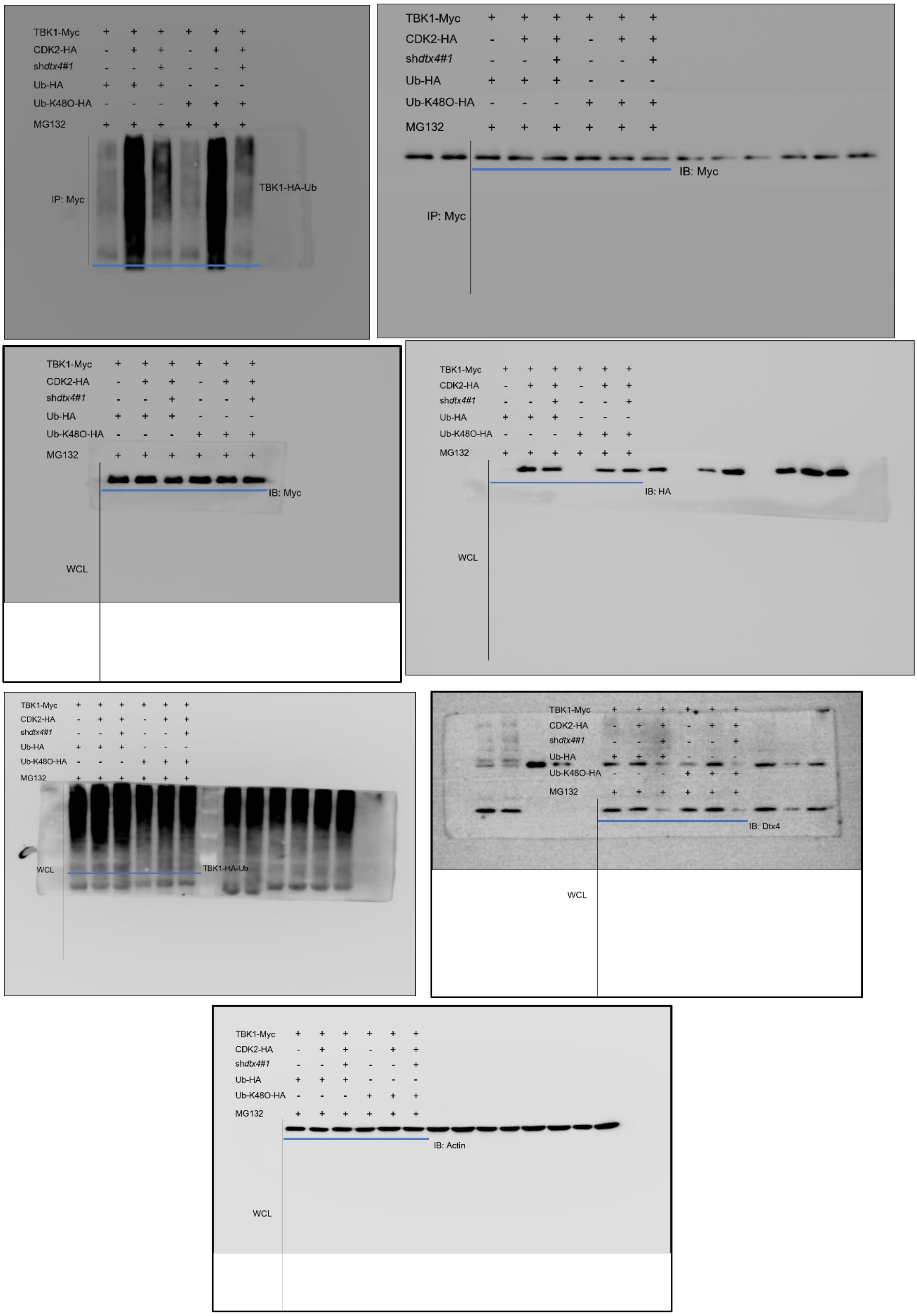

Figure 7, Source Data 1. Original membranes corresponding to Figure 7, panel C. Each membrane is labelled with the relevant information. The blue lines indicate the corresponding bands.

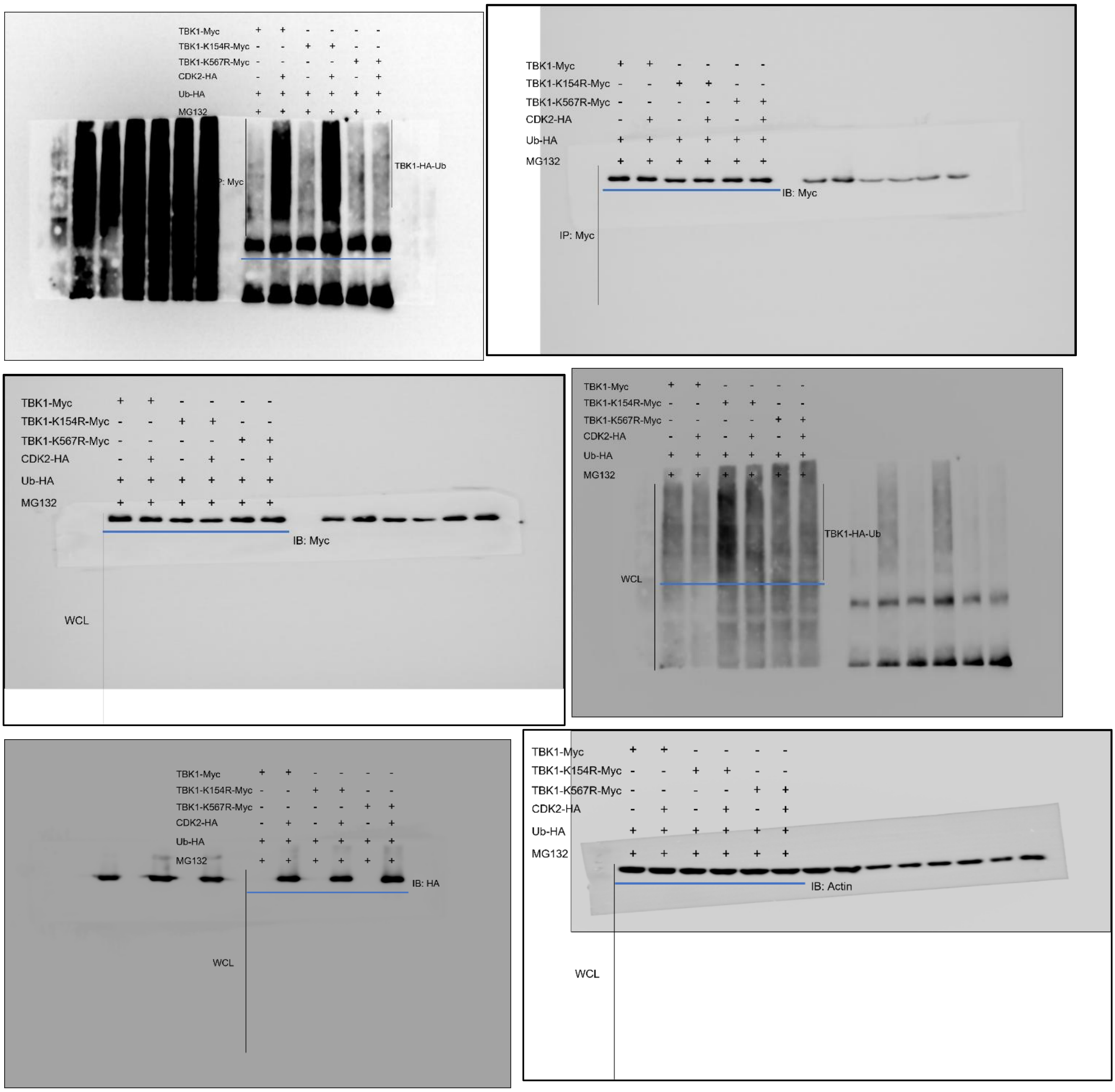

Figure 7, Source Data 1. Original membranes corresponding to Figure 7, panel E. Each membrane is labelled with the relevant information. The blue lines indicate the corresponding bands.

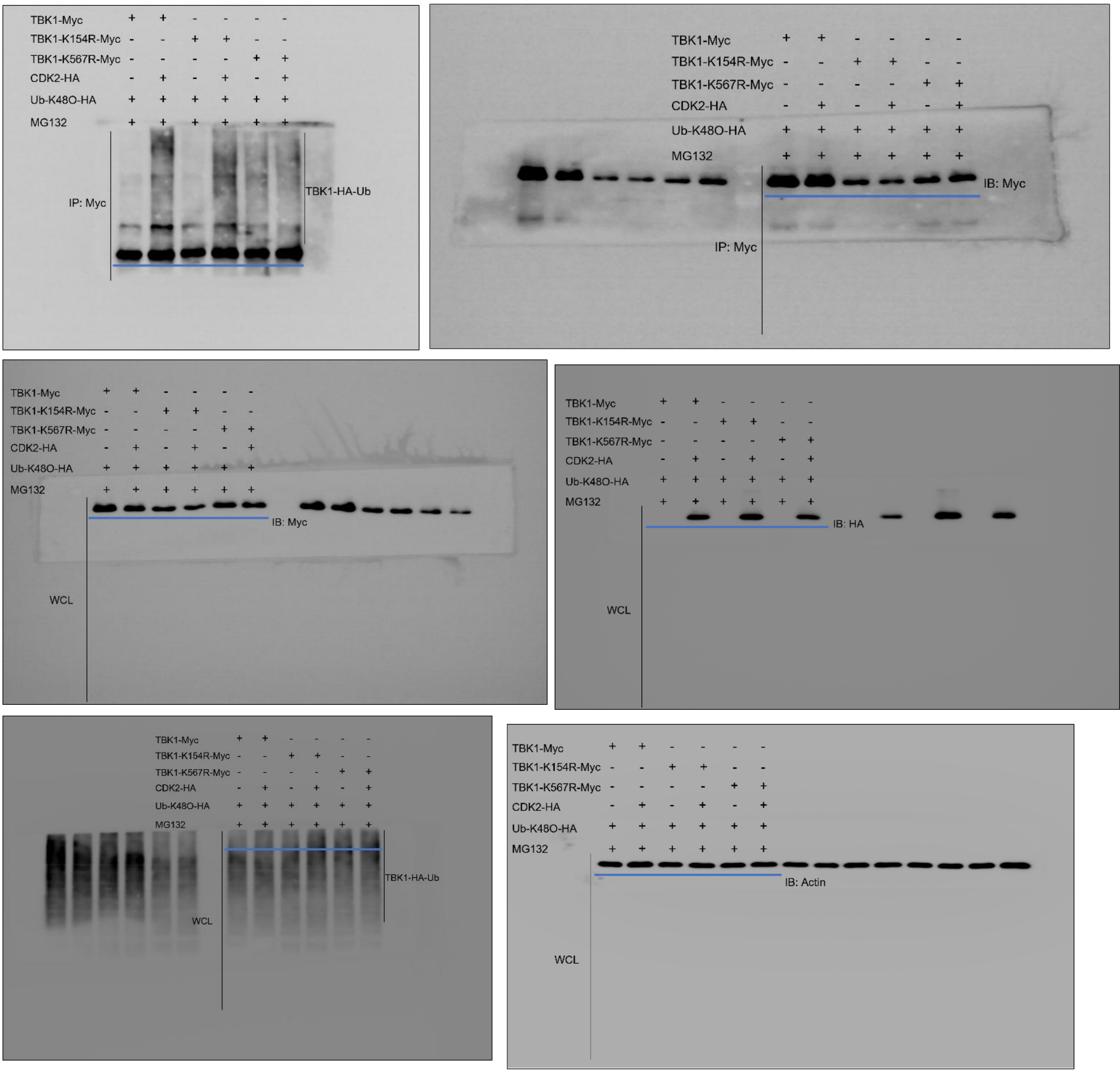

Figure 7, Source Data 1. Original membranes corresponding to Figure 7, panel F. Each membrane is labelled with the relevant information. The blue lines indicate the corresponding bands.

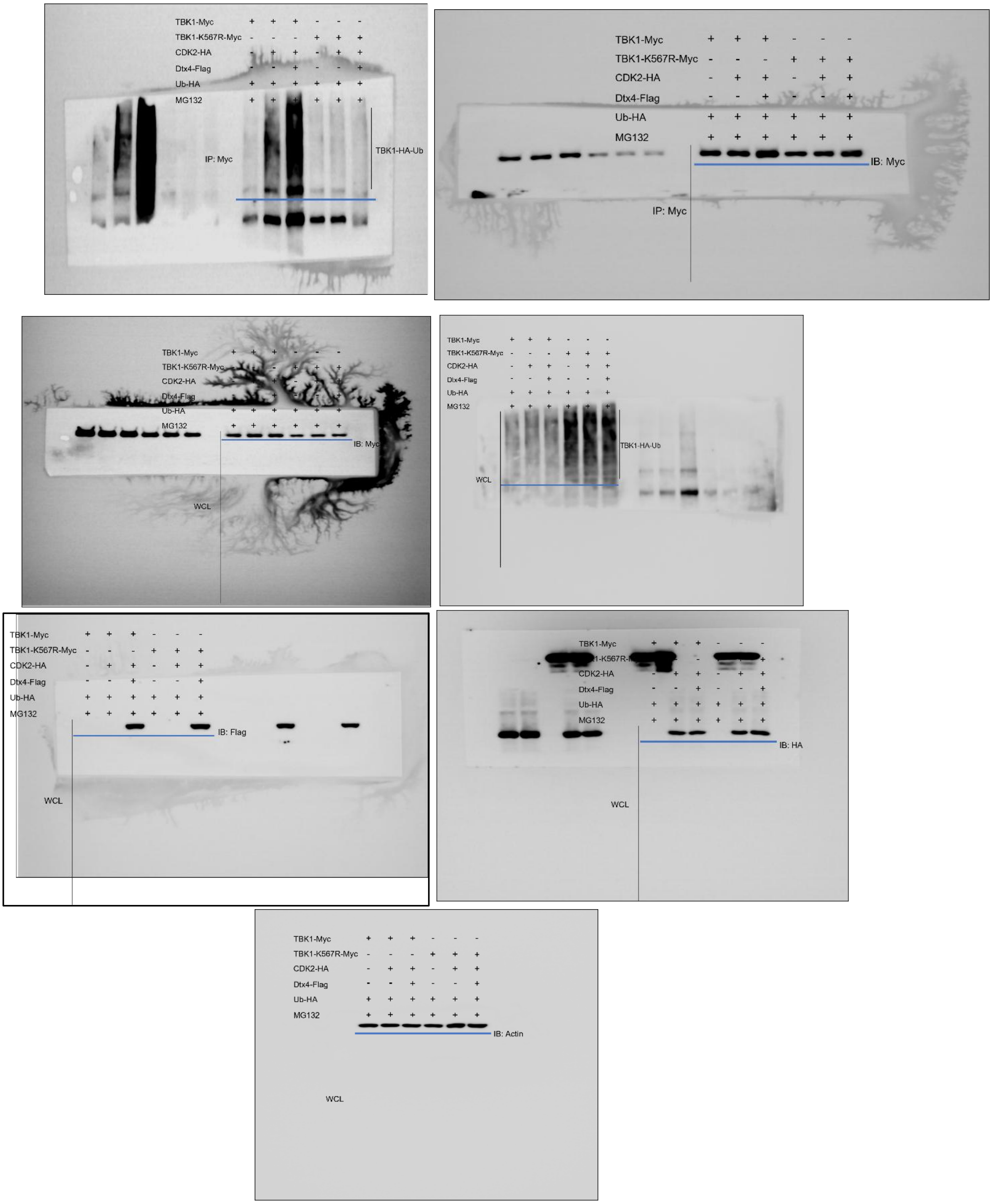

Figure 7, Source Data 1. Original membranes corresponding to Figure 7, panel G. Each membrane is labelled with the relevant information. The blue lines indicate the corresponding bands.

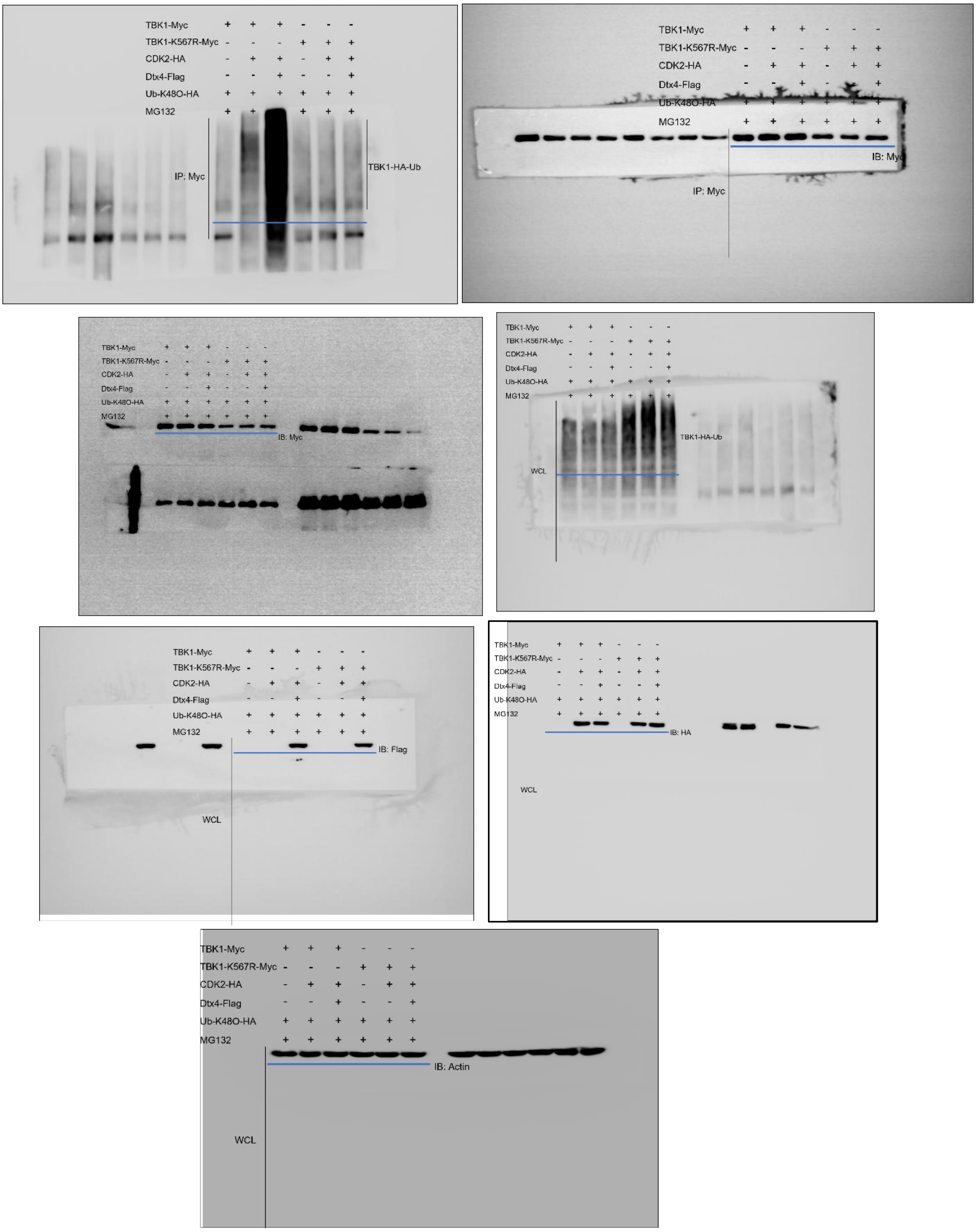

Figure 7, Source Data 1. Original membranes corresponding to Figure 7, panel H. Each membrane is labelled with the relevant information. The blue lines indicate the corresponding bands.

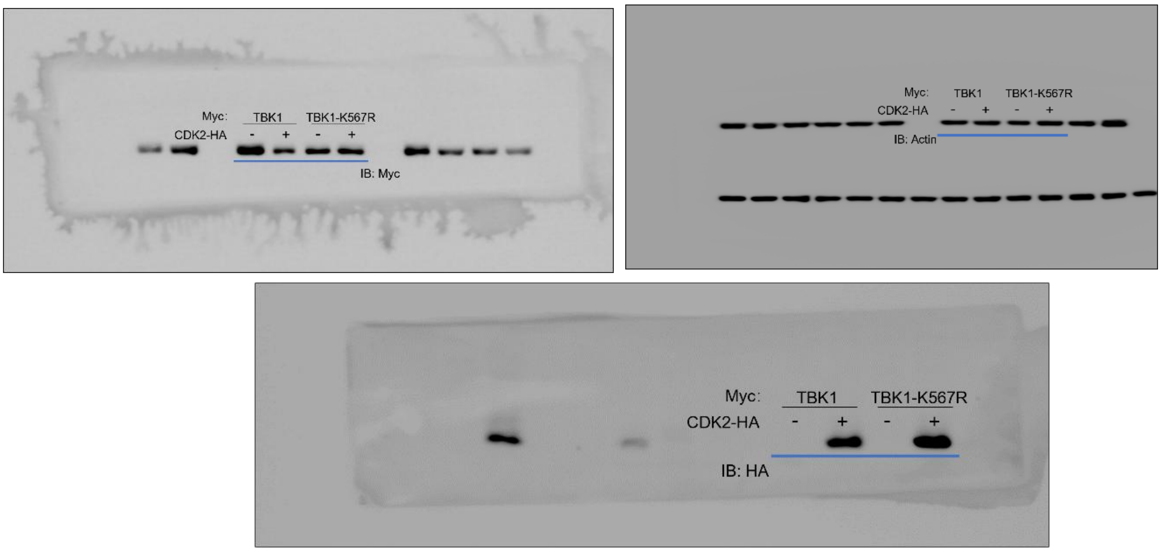

Figure 7, Source Data 1. Original membranes corresponding to Figure 7, panel I. Each membrane is labelled with the relevant information. The blue lines indicate the corresponding bands.

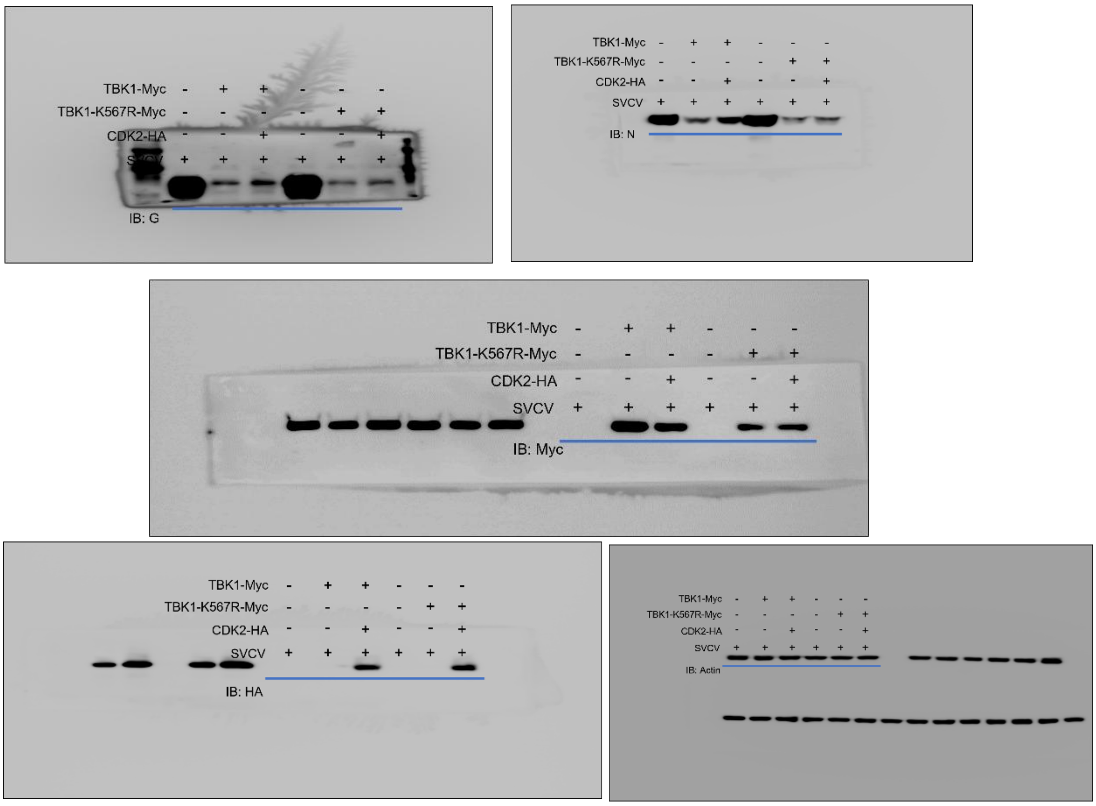

Figure 7, Source Data 1. Original membranes corresponding to Figure 7, panel M. Each membrane is labelled with the relevant information. The blue lines indicate the corresponding bands.

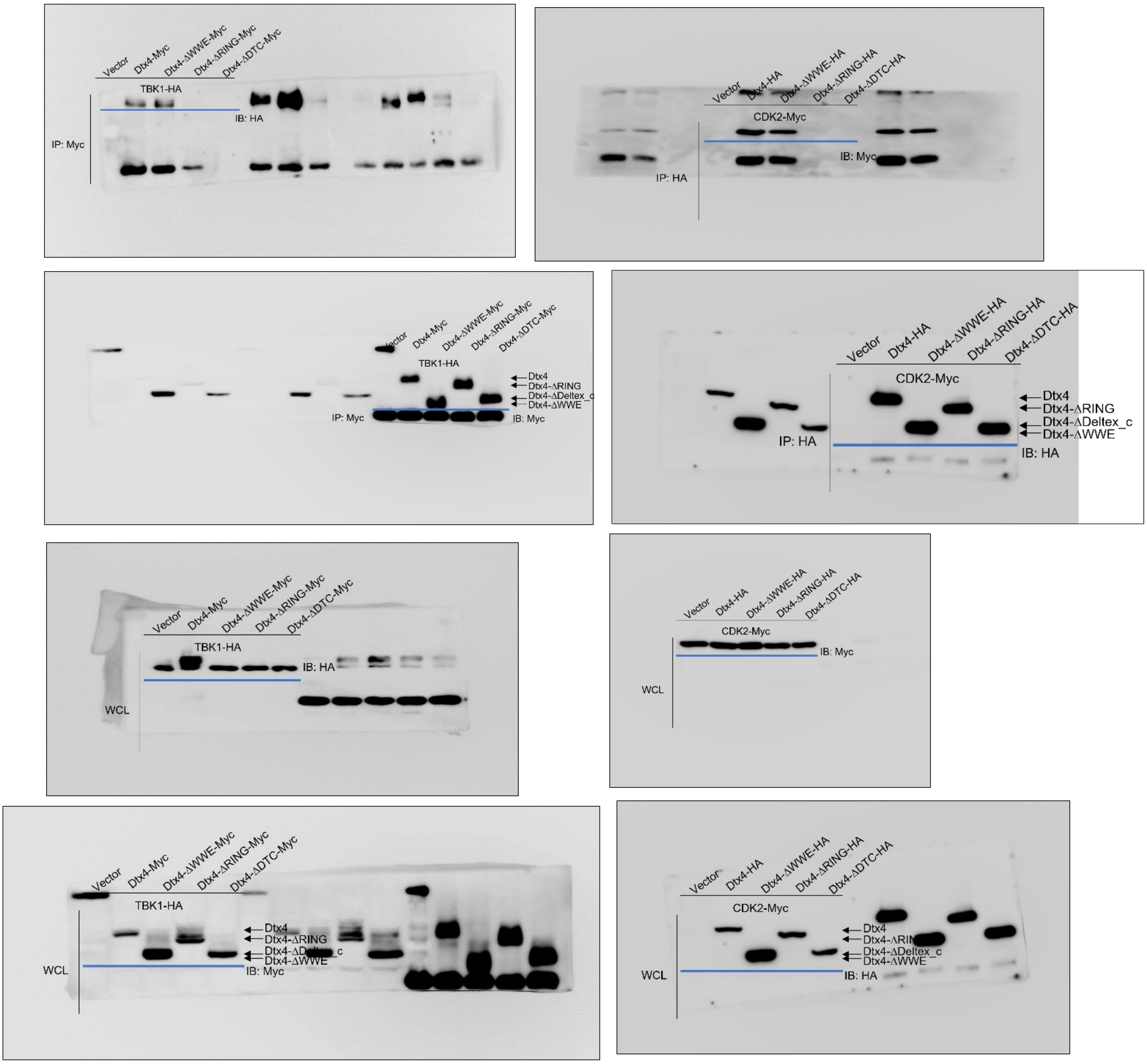

Figure 7, Source Data 1. Original membranes corresponding to Figure 7, panel Q. Each membrane is labelled with the relevant information. The blue lines indicate the corresponding bands.

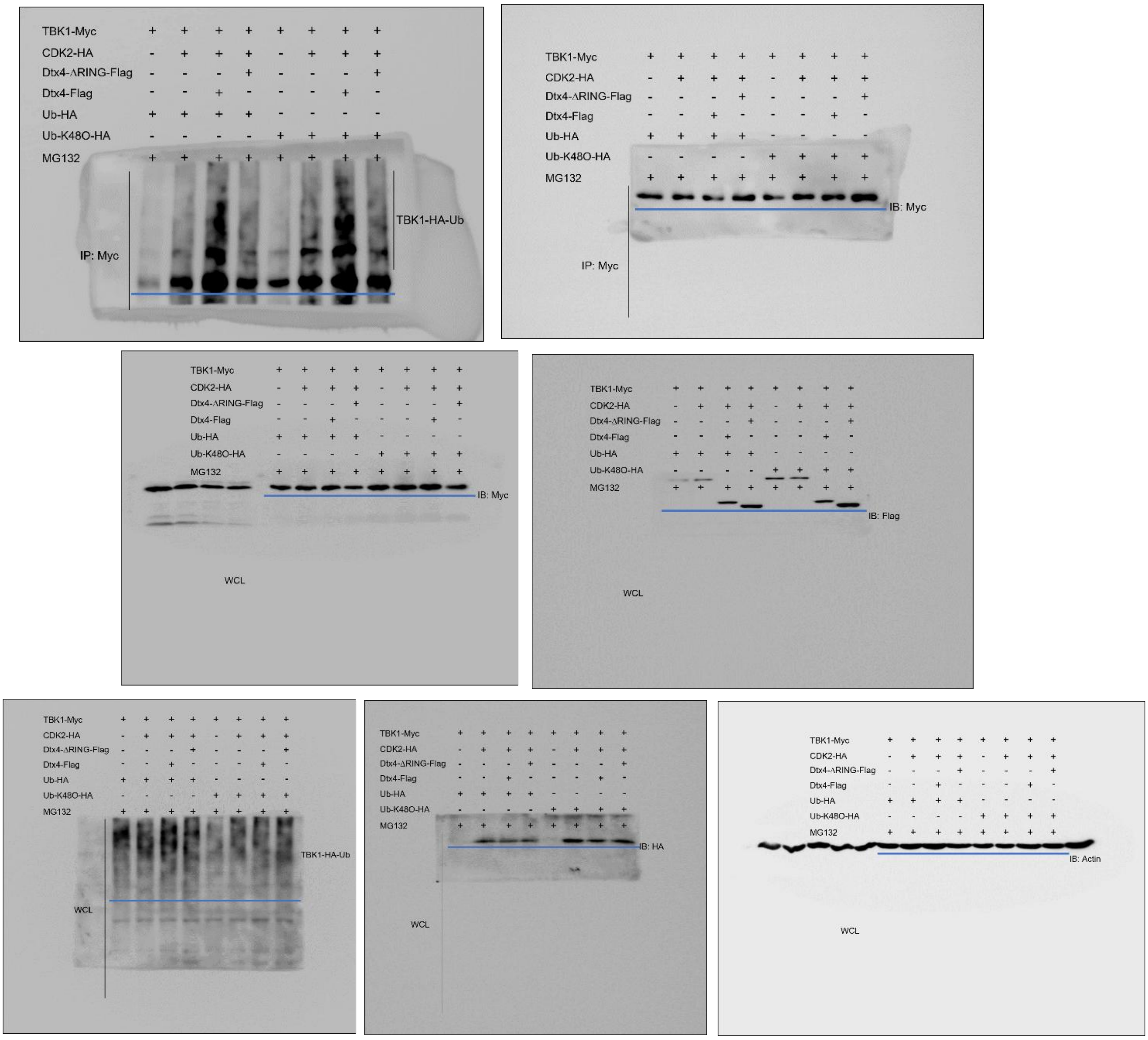

Figure 7, Source Data 1. Original membranes corresponding to Figure 7, panel R. Each membrane is labelled with the relevant information. The blue lines indicate the corresponding bands.
