# Supplementary figures and images for: Fish CDK2 recruits Dtx4 to degrade TBK1 through ubiquitination in the antiviral response (part 3 of 3)
Source: eLife. 2026 Jan 14;13:RP98357. doi: 10.7554/eLife.98357 (PMC12803515; doi:10.7554/eLife.98357)

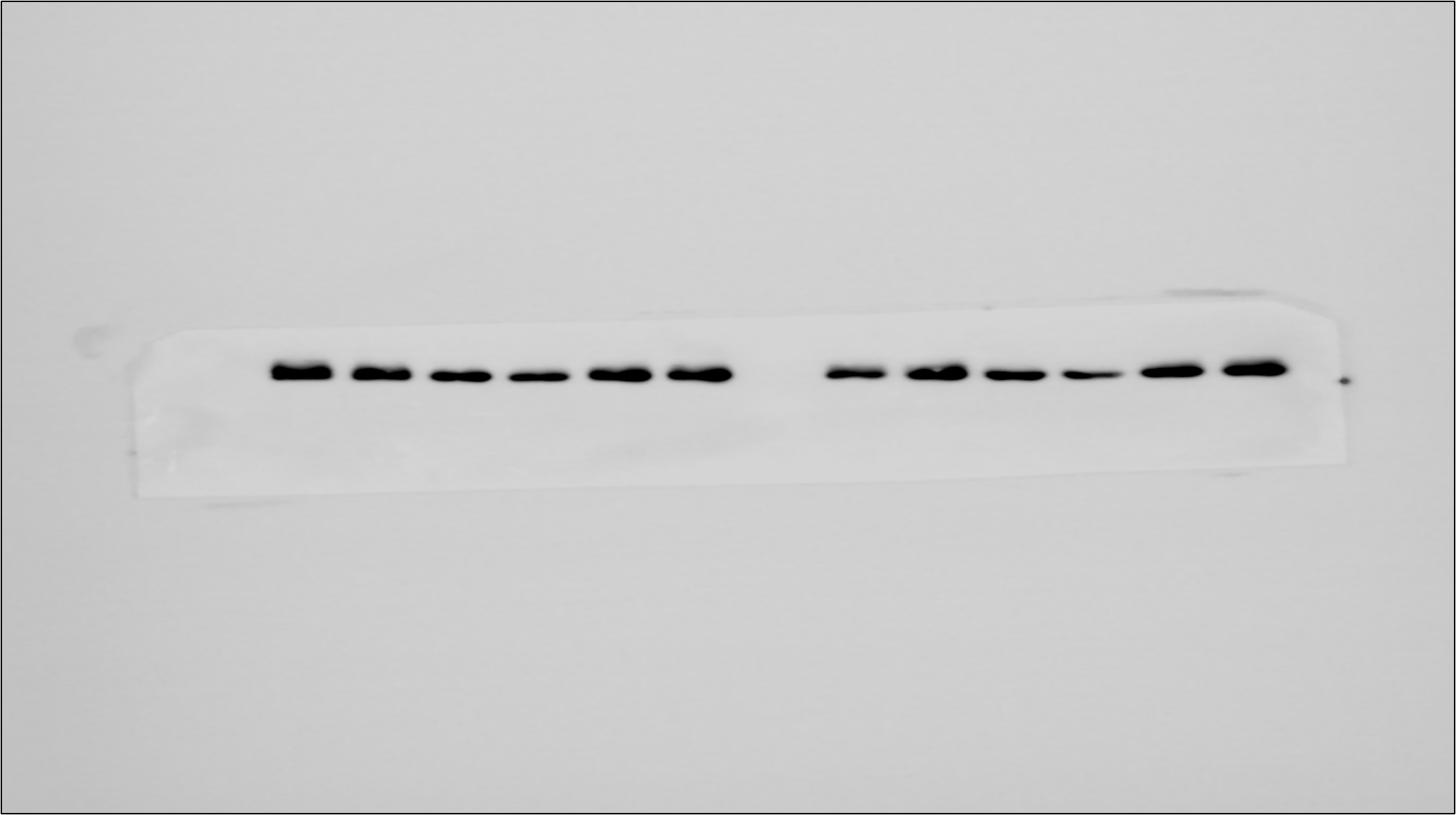

Supplement: Figure 7—source data 2. [file elife-98357-fig7-data2.zip › Figure 7-source data 2/7E-WCL-Myc-1.tif]

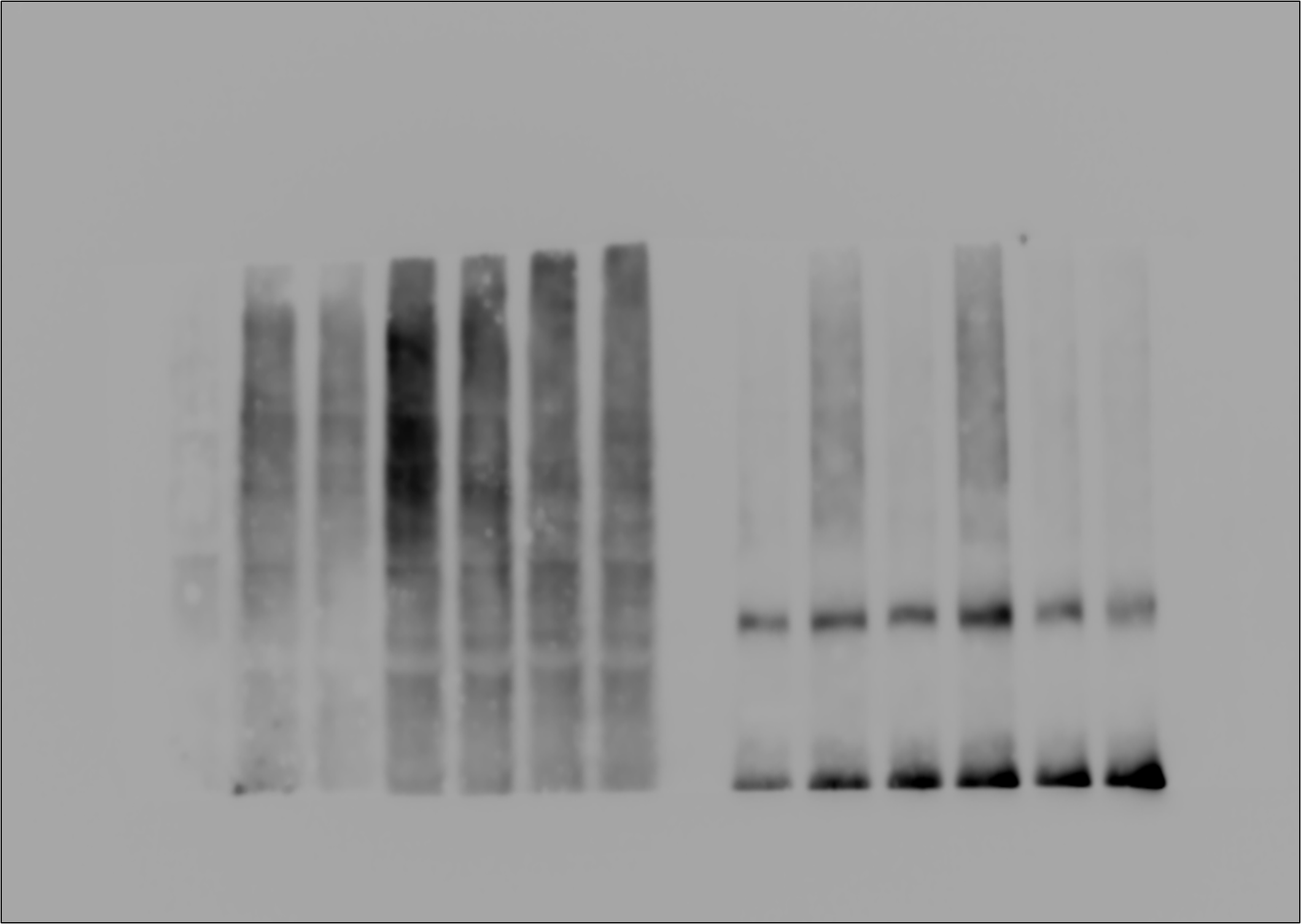

Supplement: Figure 7—source data 2. [file elife-98357-fig7-data2.zip › Figure 7-source data 2/7E-WCL-TBK1-HA-Ub-1.tif]

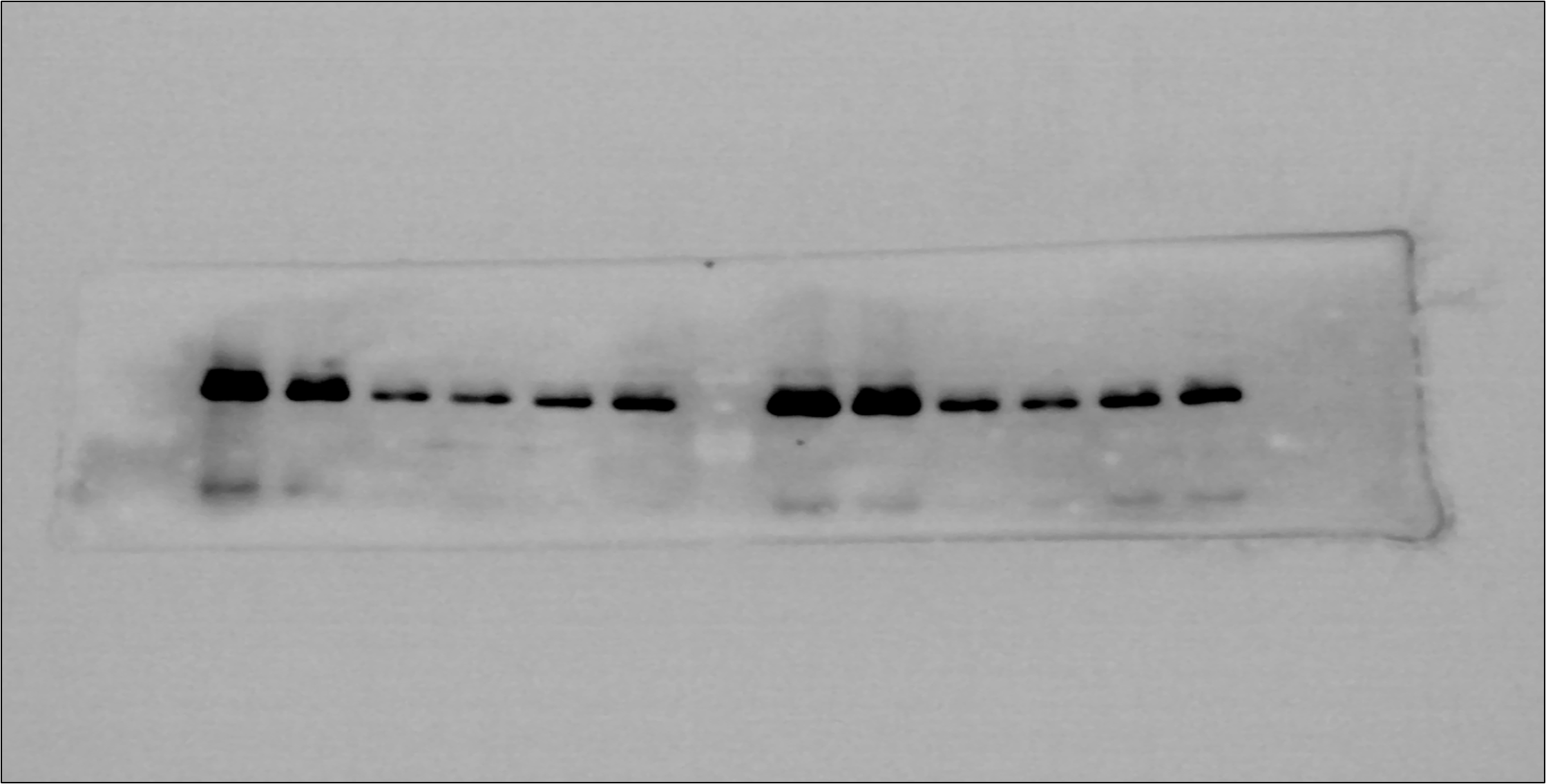

Supplement: Figure 7—source data 2. [file elife-98357-fig7-data2.zip › Figure 7-source data 2/7F-IP-Myc-1.tif]

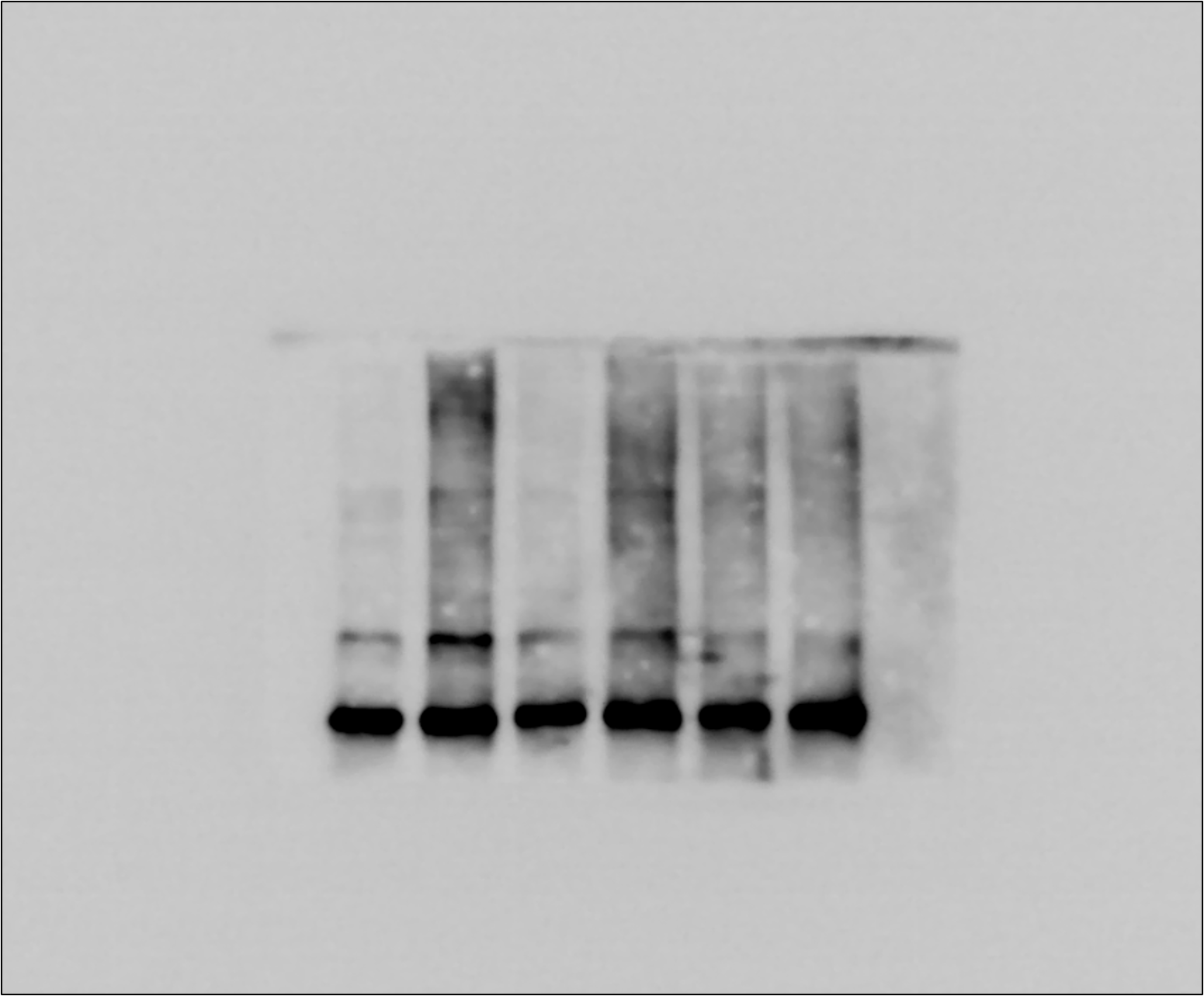

Supplement: Figure 7—source data 2. [file elife-98357-fig7-data2.zip › Figure 7-source data 2/7F-IP-TBK1-HA-Ub-1.tif]

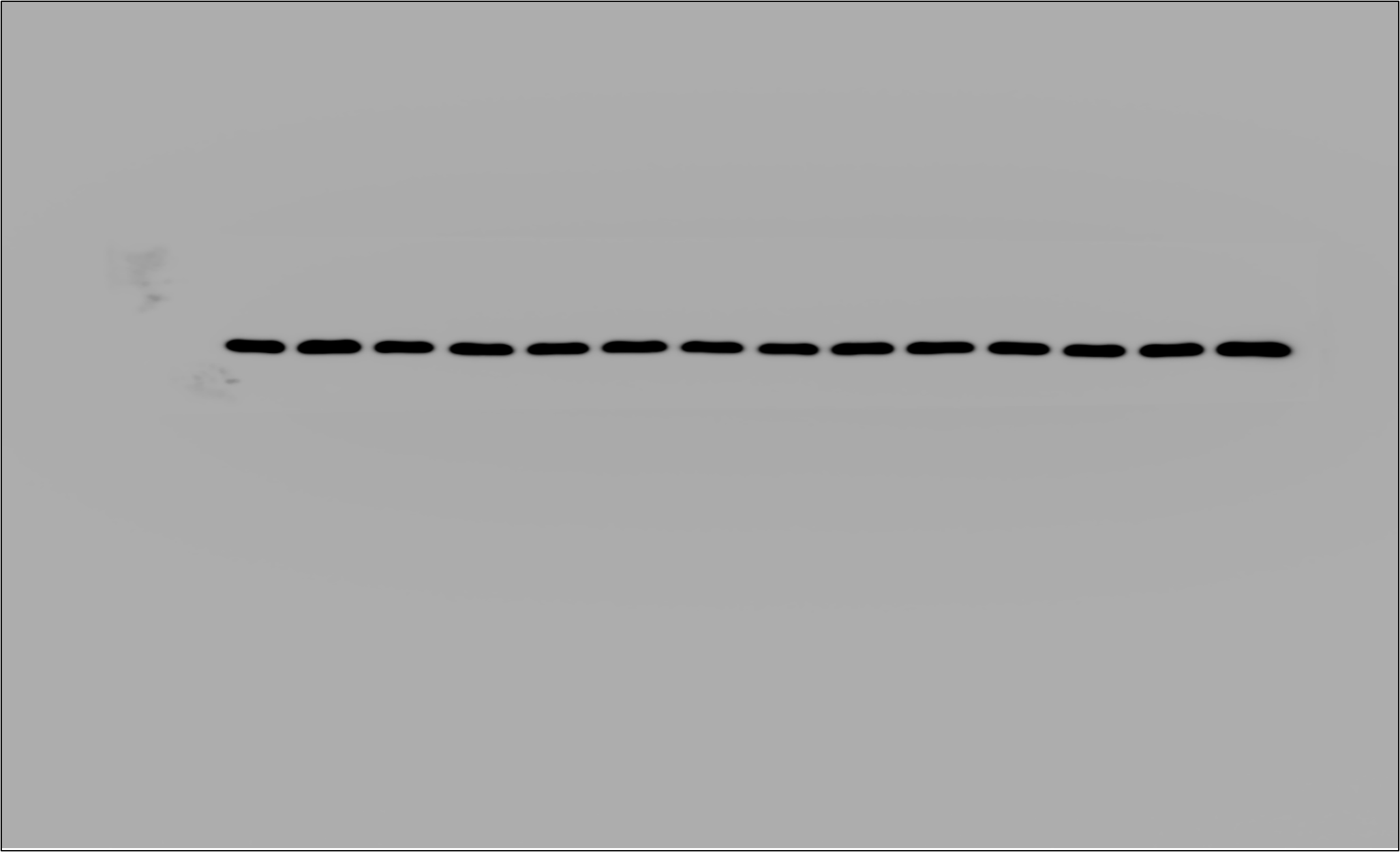

Supplement: Figure 7—source data 2. [file elife-98357-fig7-data2.zip › Figure 7-source data 2/7F-WCL-Actin-1.tif]

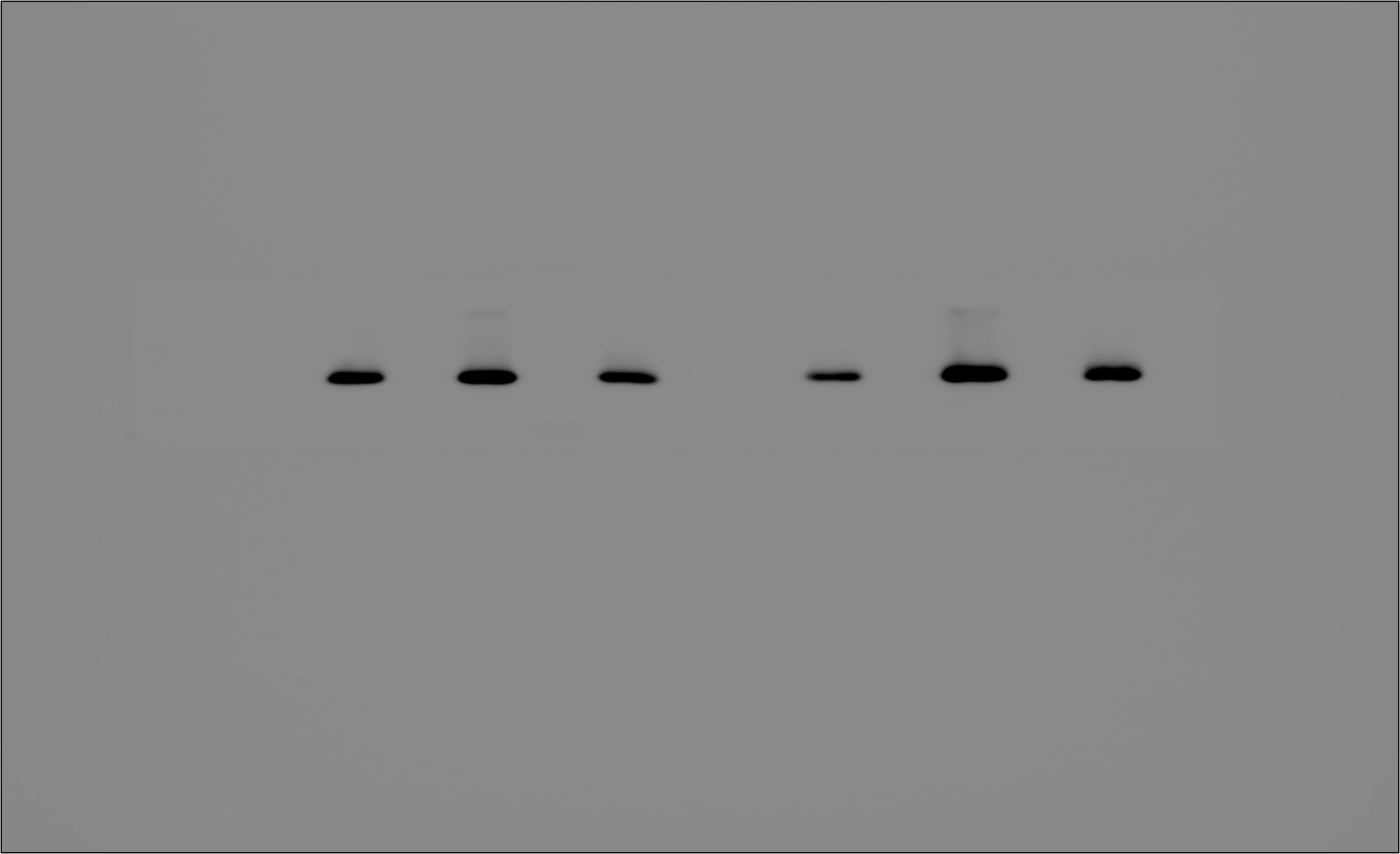

Supplement: Figure 7—source data 2. [file elife-98357-fig7-data2.zip › Figure 7-source data 2/7F-WCL-HA-1.tif]

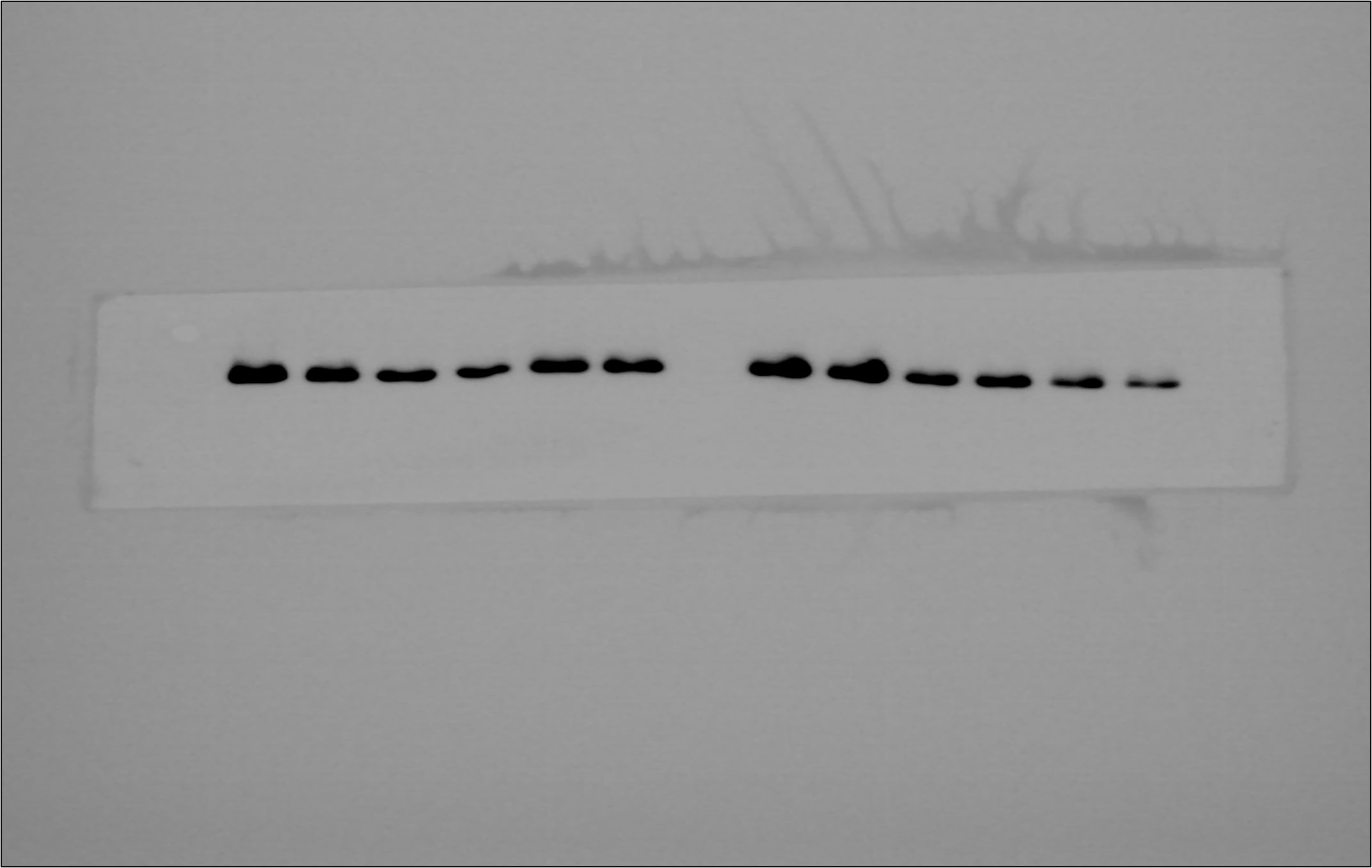

Supplement: Figure 7—source data 2. [file elife-98357-fig7-data2.zip › Figure 7-source data 2/7F-WCL-Myc-1.tif]

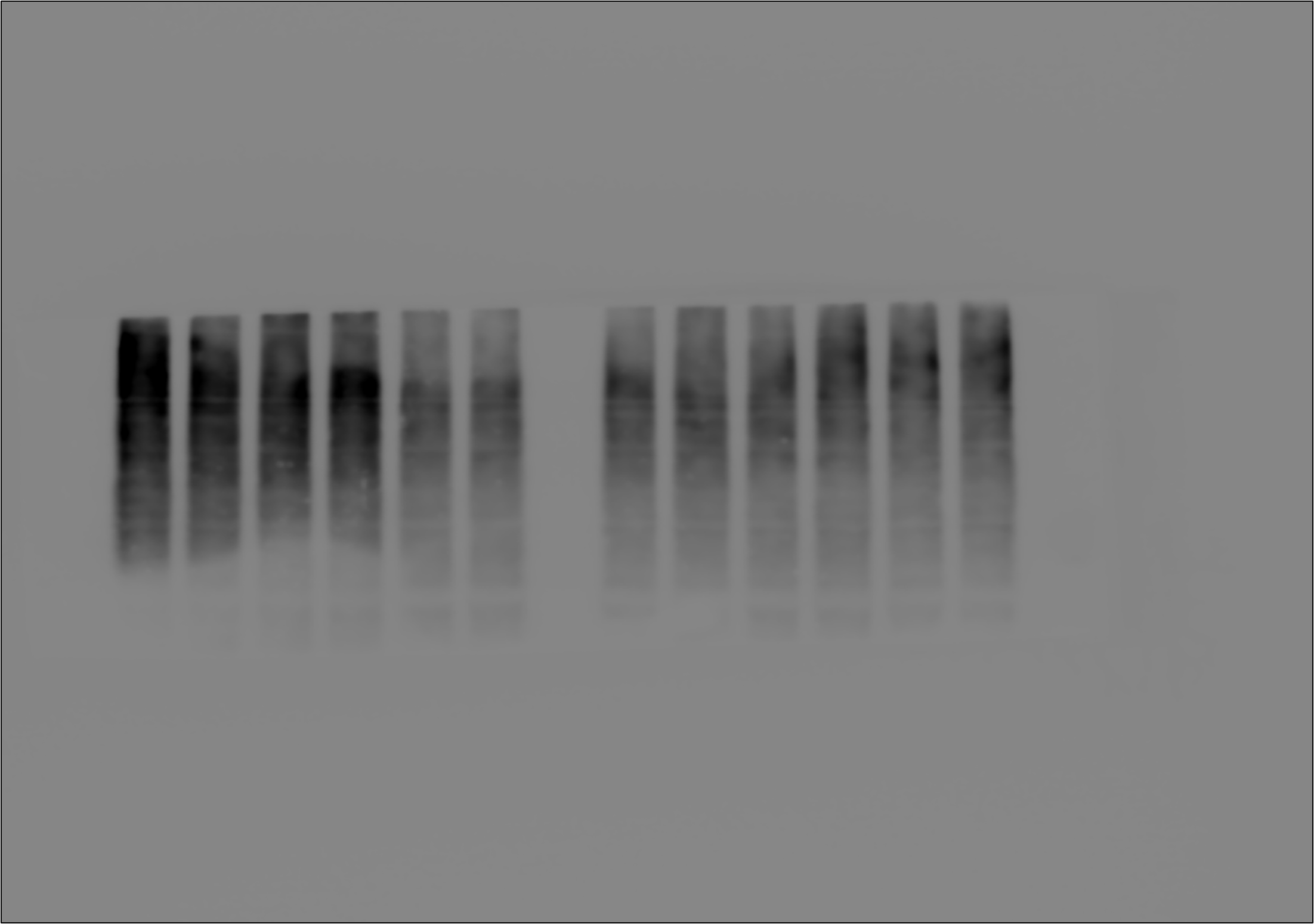

Supplement: Figure 7—source data 2. [file elife-98357-fig7-data2.zip › Figure 7-source data 2/7F-WCL-TBK1-HA-Ub-1.tif]

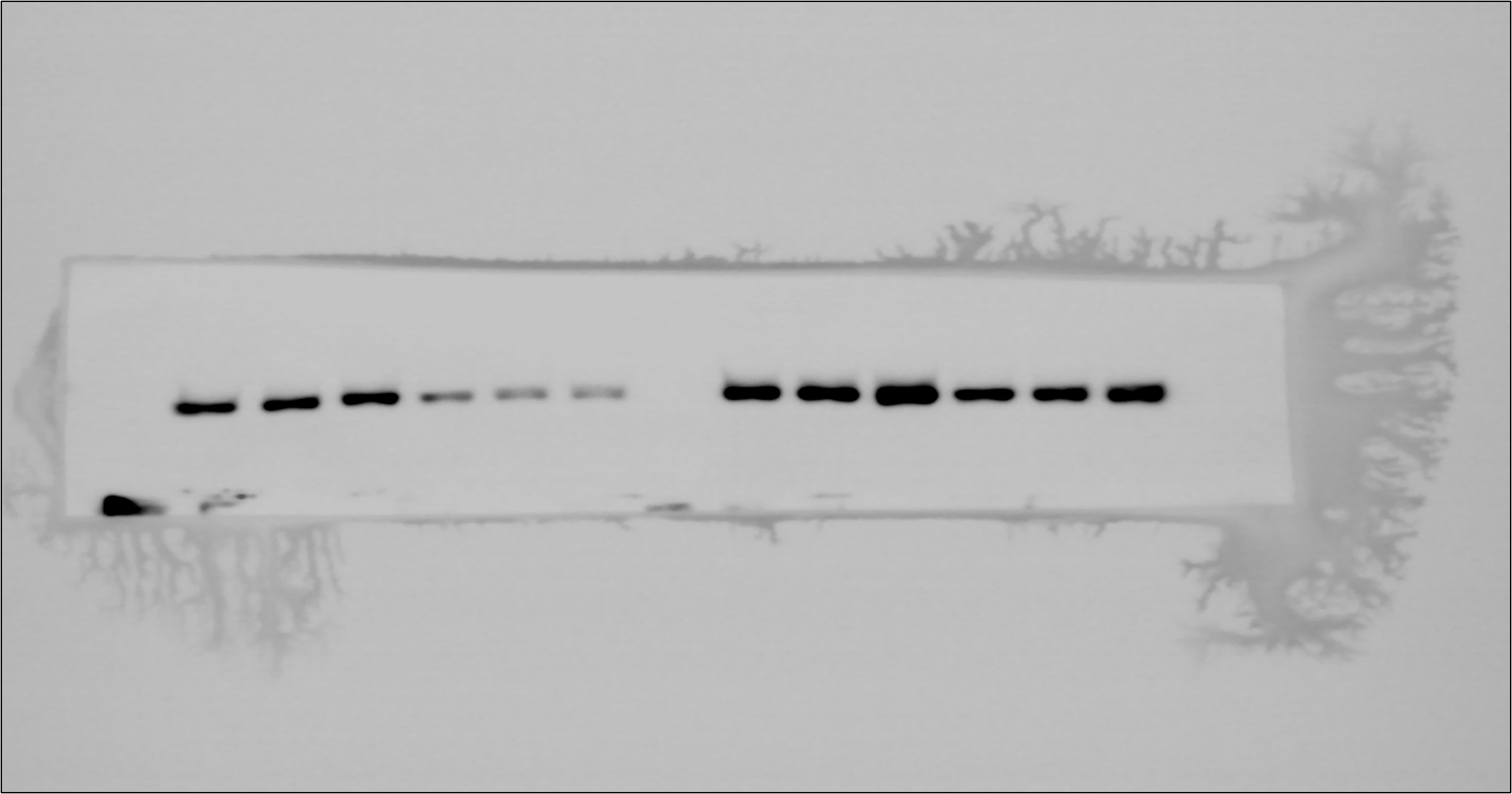

Supplement: Figure 7—source data 2. [file elife-98357-fig7-data2.zip › Figure 7-source data 2/7G-IP-Myc-1.tif]

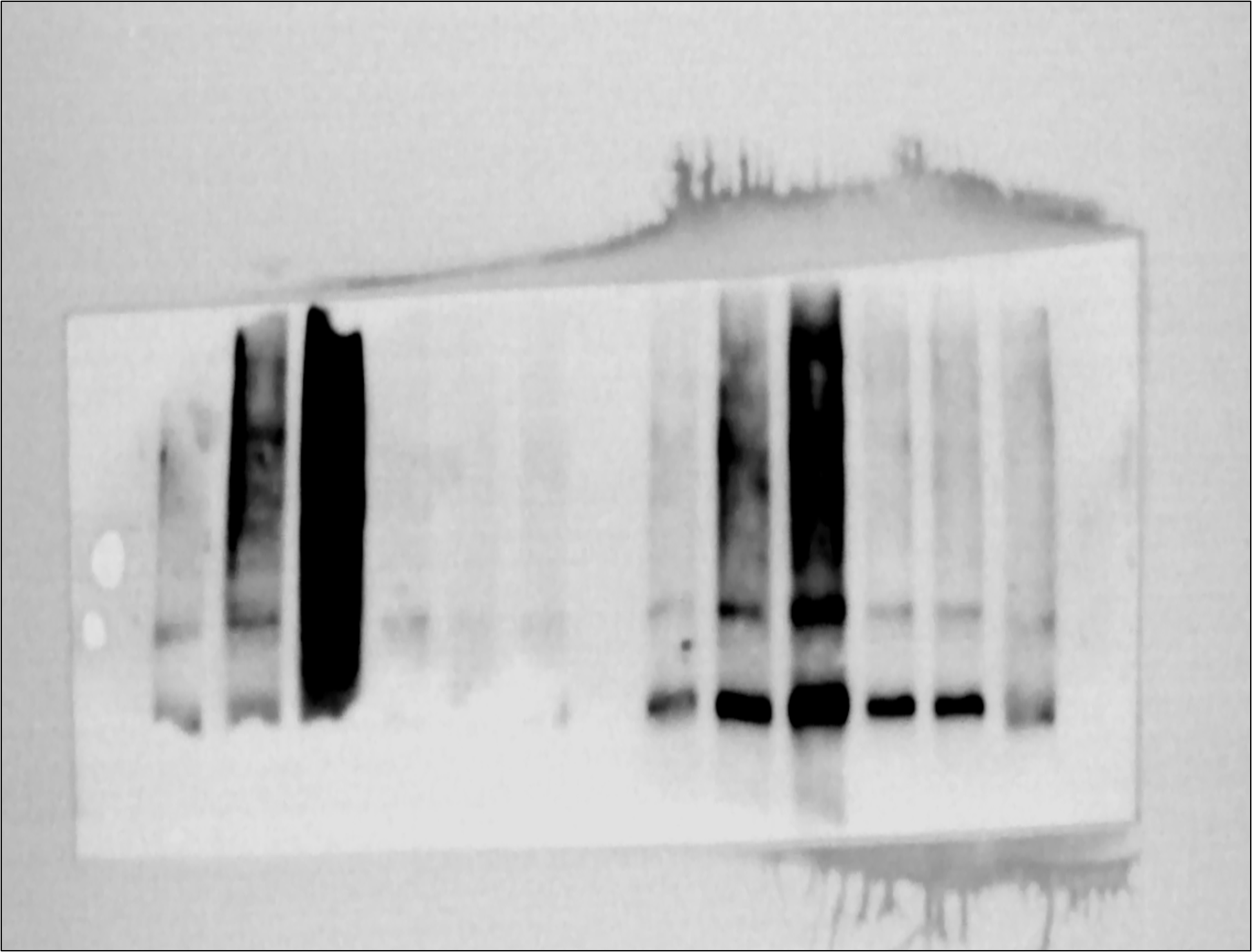

Supplement: Figure 7—source data 2. [file elife-98357-fig7-data2.zip › Figure 7-source data 2/7G-IP-TBK1-HA-Ub-1.tif]

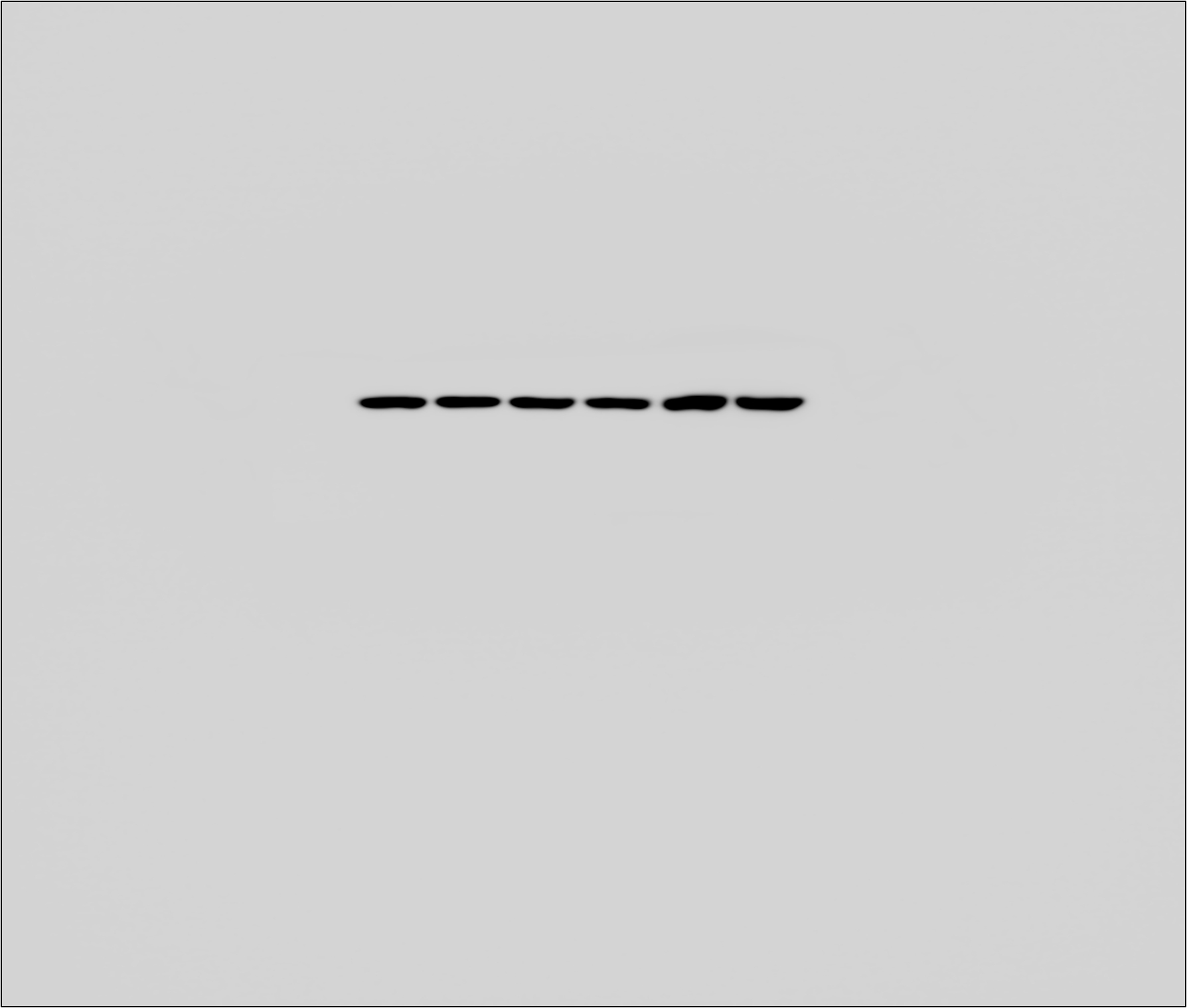

Supplement: Figure 7—source data 2. [file elife-98357-fig7-data2.zip › Figure 7-source data 2/7G-WCL-Actin-1.tif]

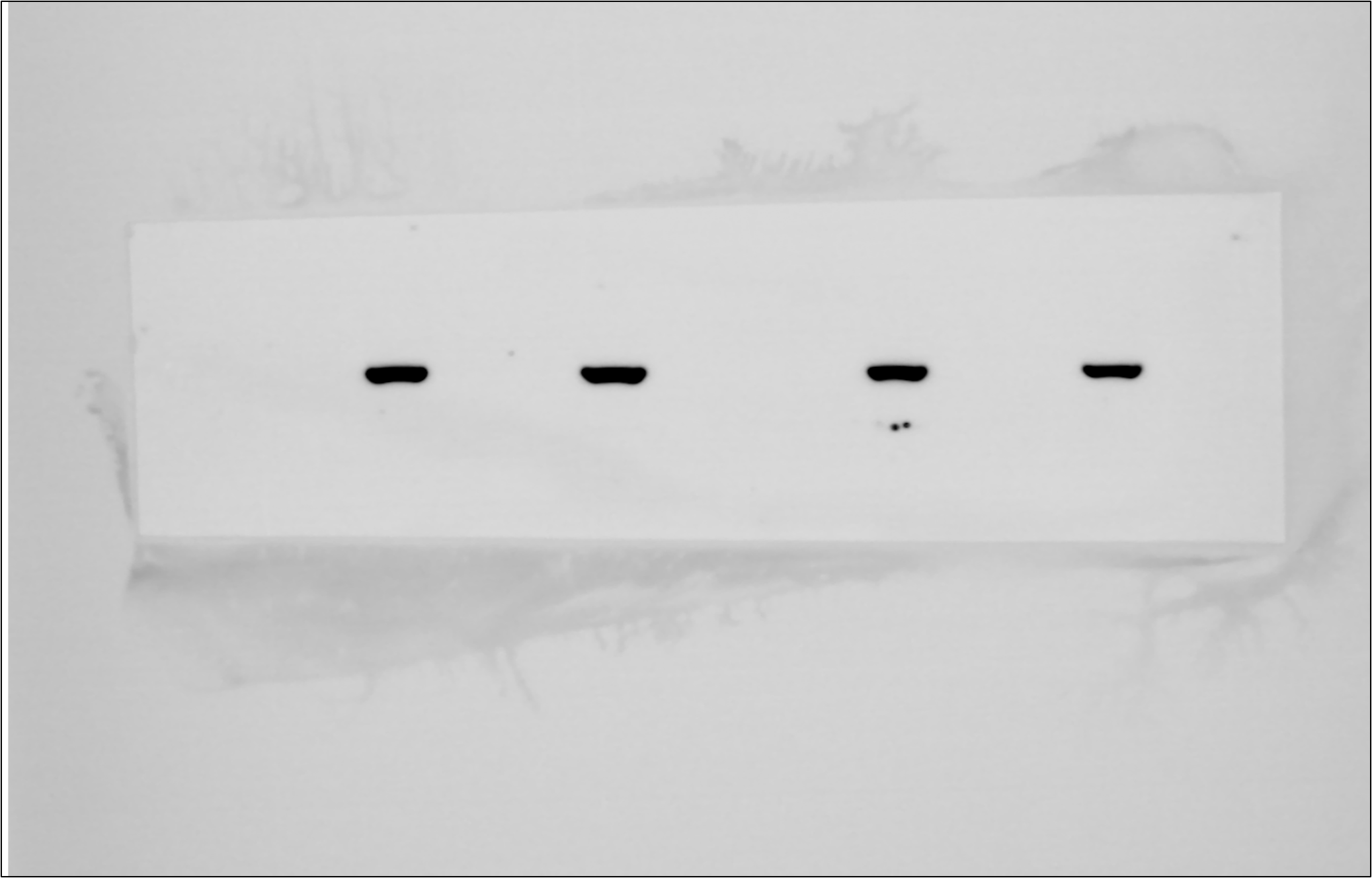

Supplement: Figure 7—source data 2. [file elife-98357-fig7-data2.zip › Figure 7-source data 2/7G-WCL-Flag-1.tif]

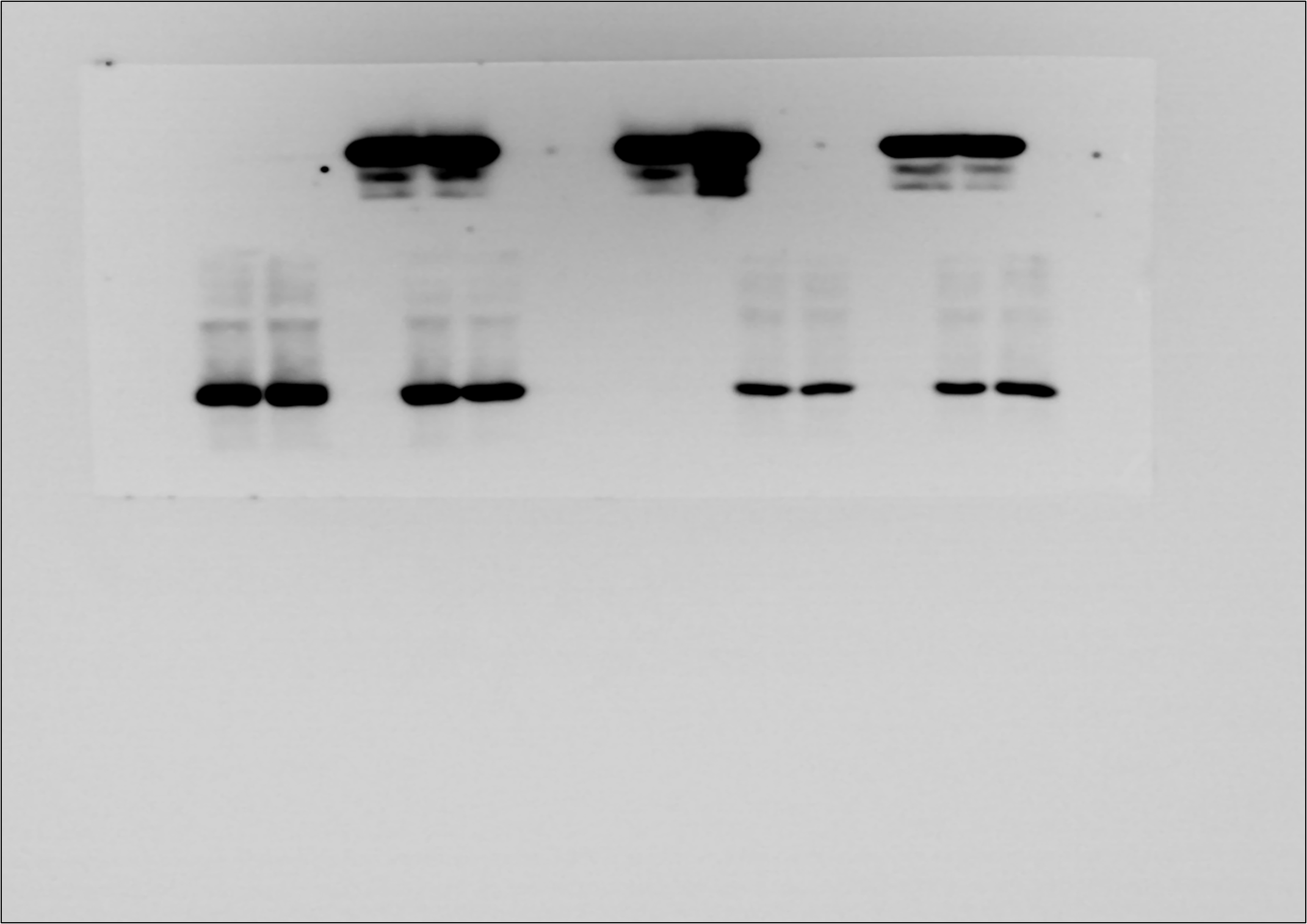

Supplement: Figure 7—source data 2. [file elife-98357-fig7-data2.zip › Figure 7-source data 2/7G-WCL-HA-1.tif]

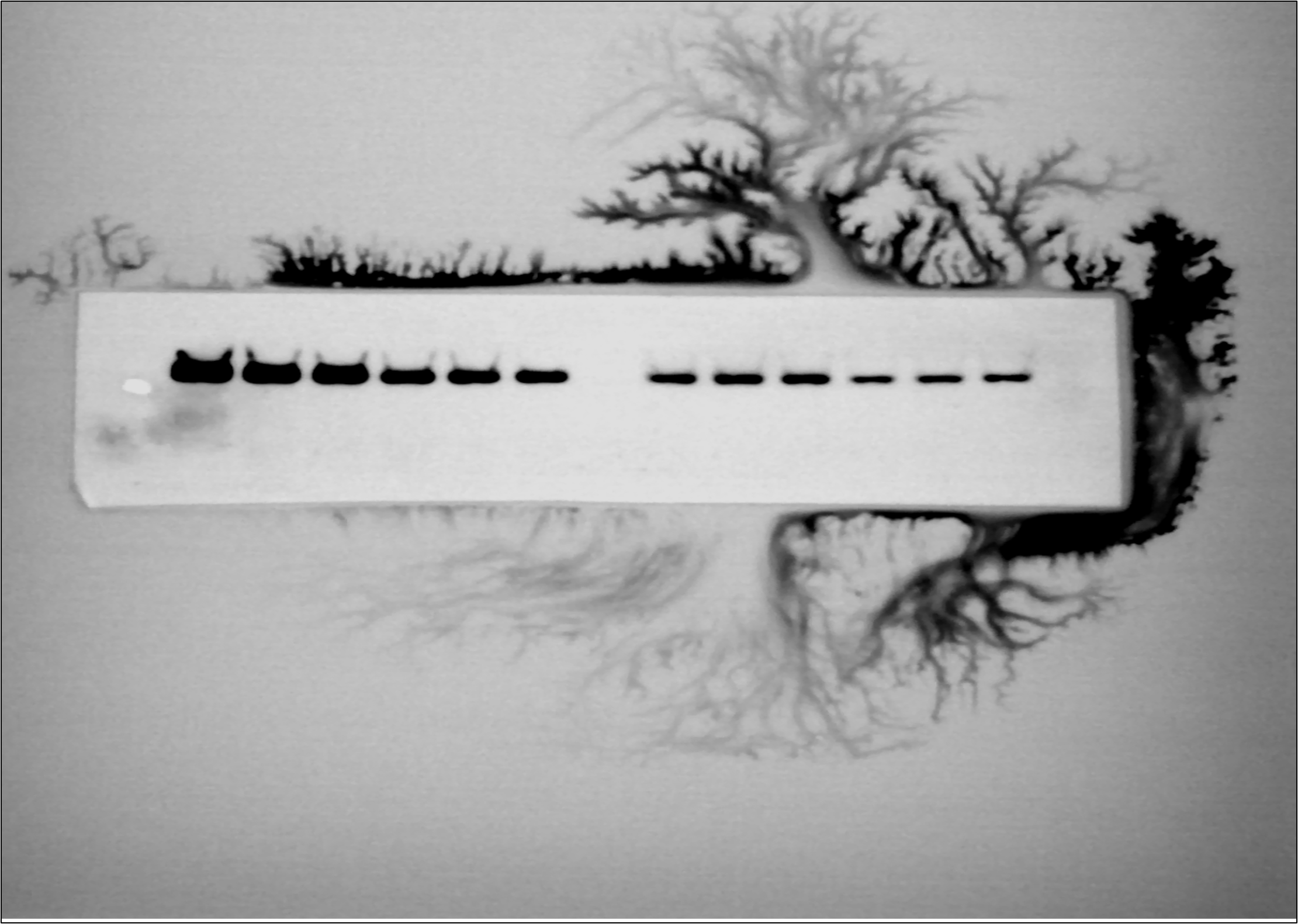

Supplement: Figure 7—source data 2. [file elife-98357-fig7-data2.zip › Figure 7-source data 2/7G-WCL-Myc-1.tif]

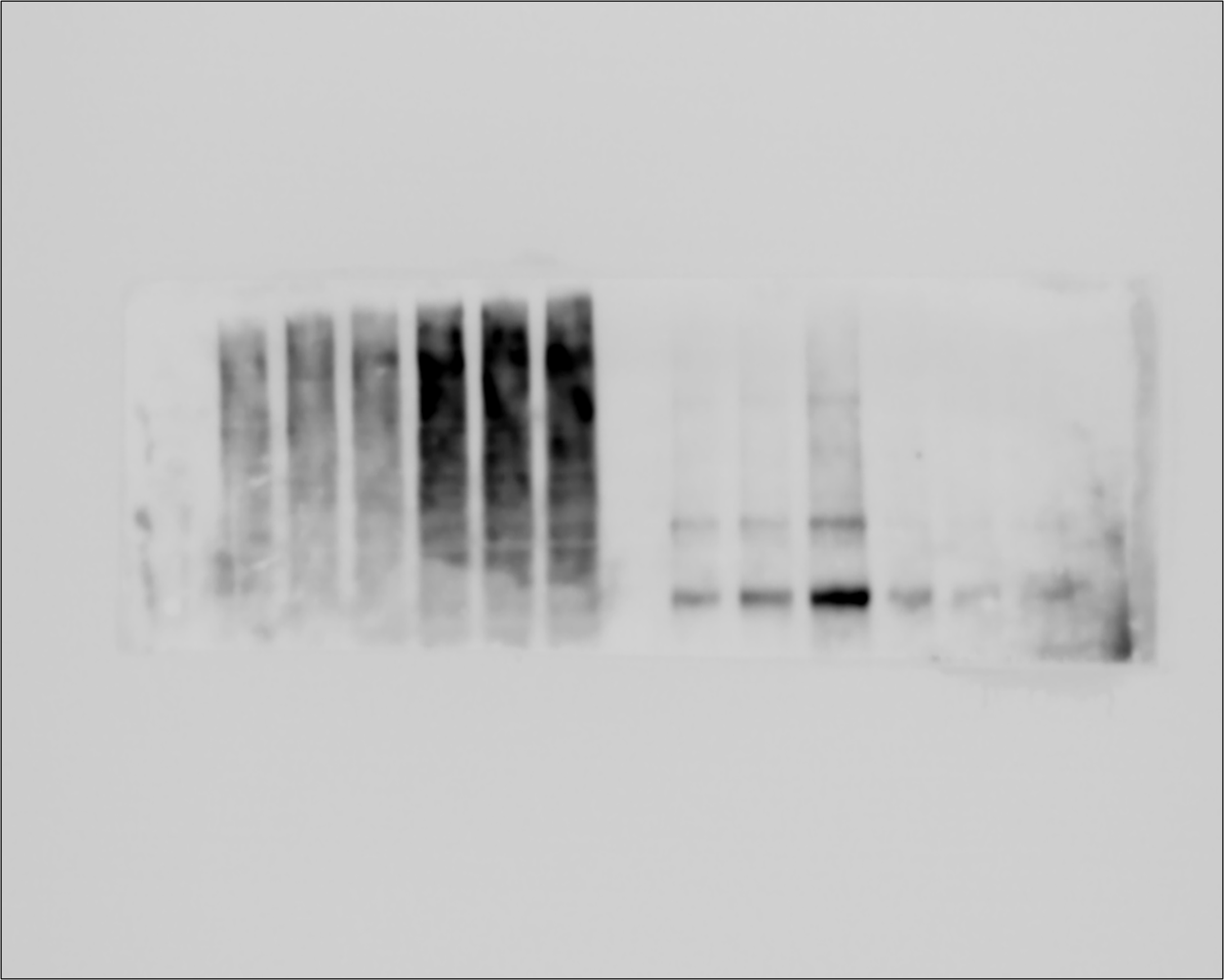

Supplement: Figure 7—source data 2. [file elife-98357-fig7-data2.zip › Figure 7-source data 2/7G-WCL-TBK1-HA-Ub-1.tif]

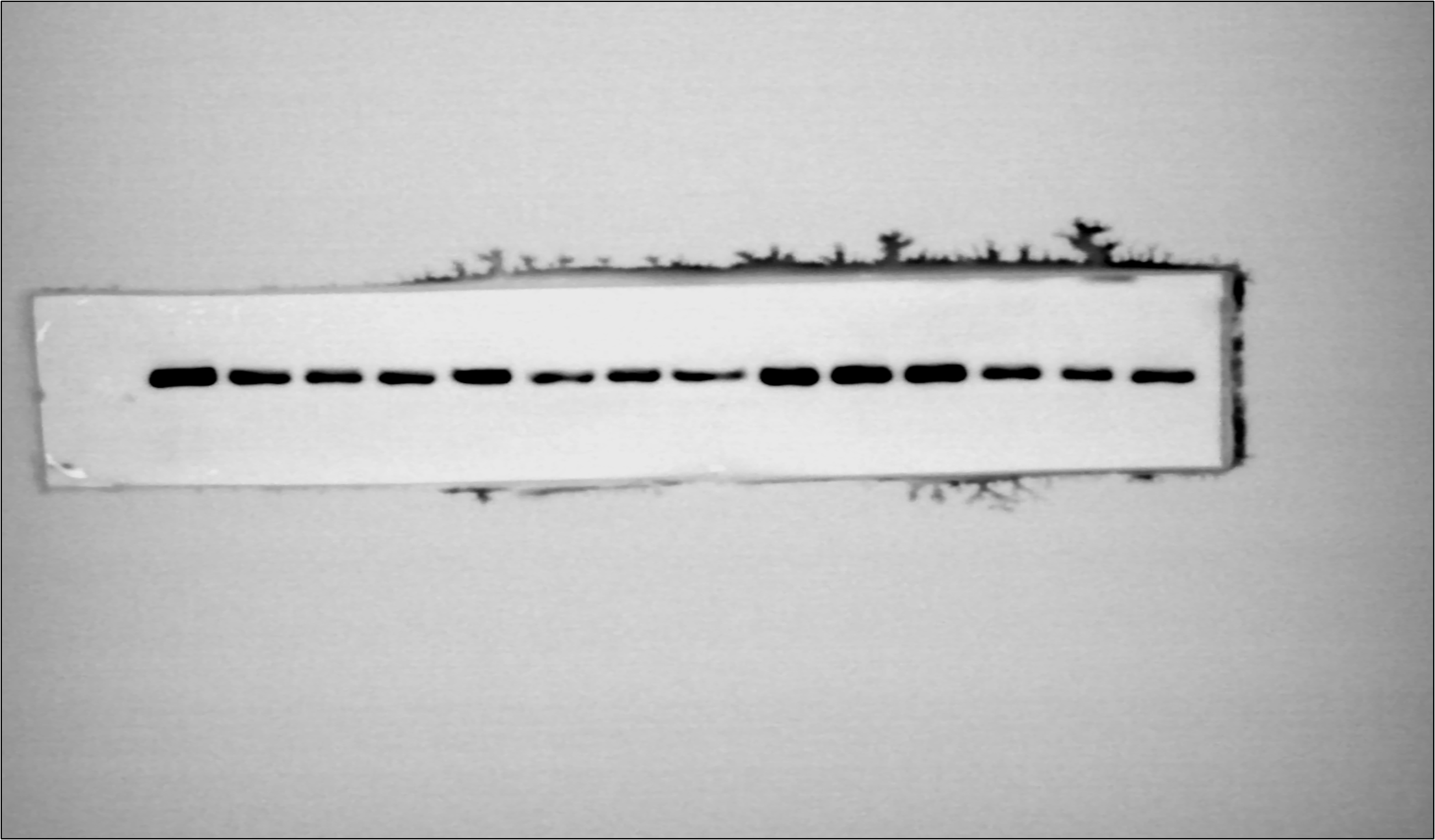

Supplement: Figure 7—source data 2. [file elife-98357-fig7-data2.zip › Figure 7-source data 2/7H-IP-Myc-1.tif]

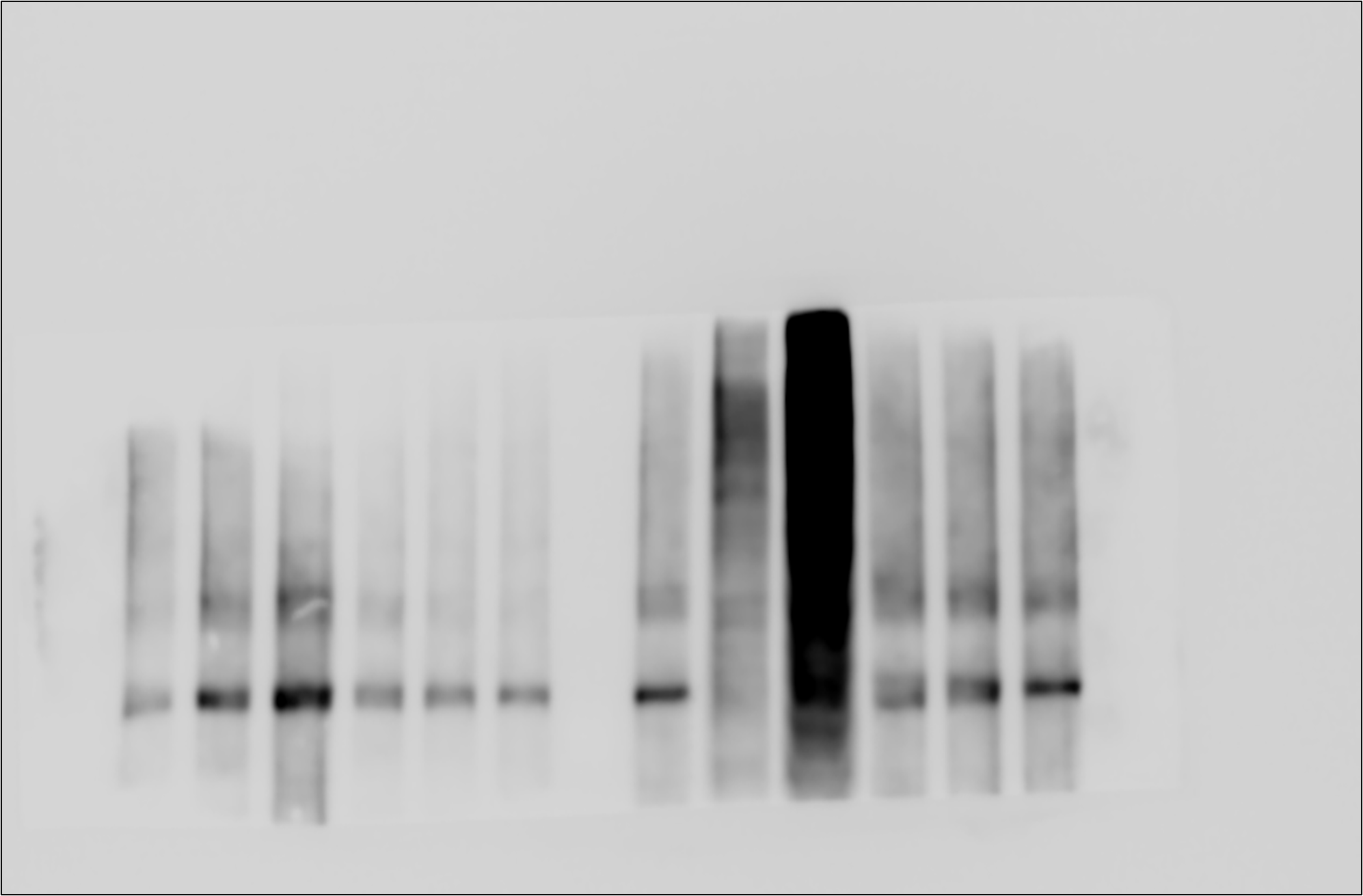

Supplement: Figure 7—source data 2. [file elife-98357-fig7-data2.zip › Figure 7-source data 2/7H-IP-TBK1-HA-Ub-1.tif]

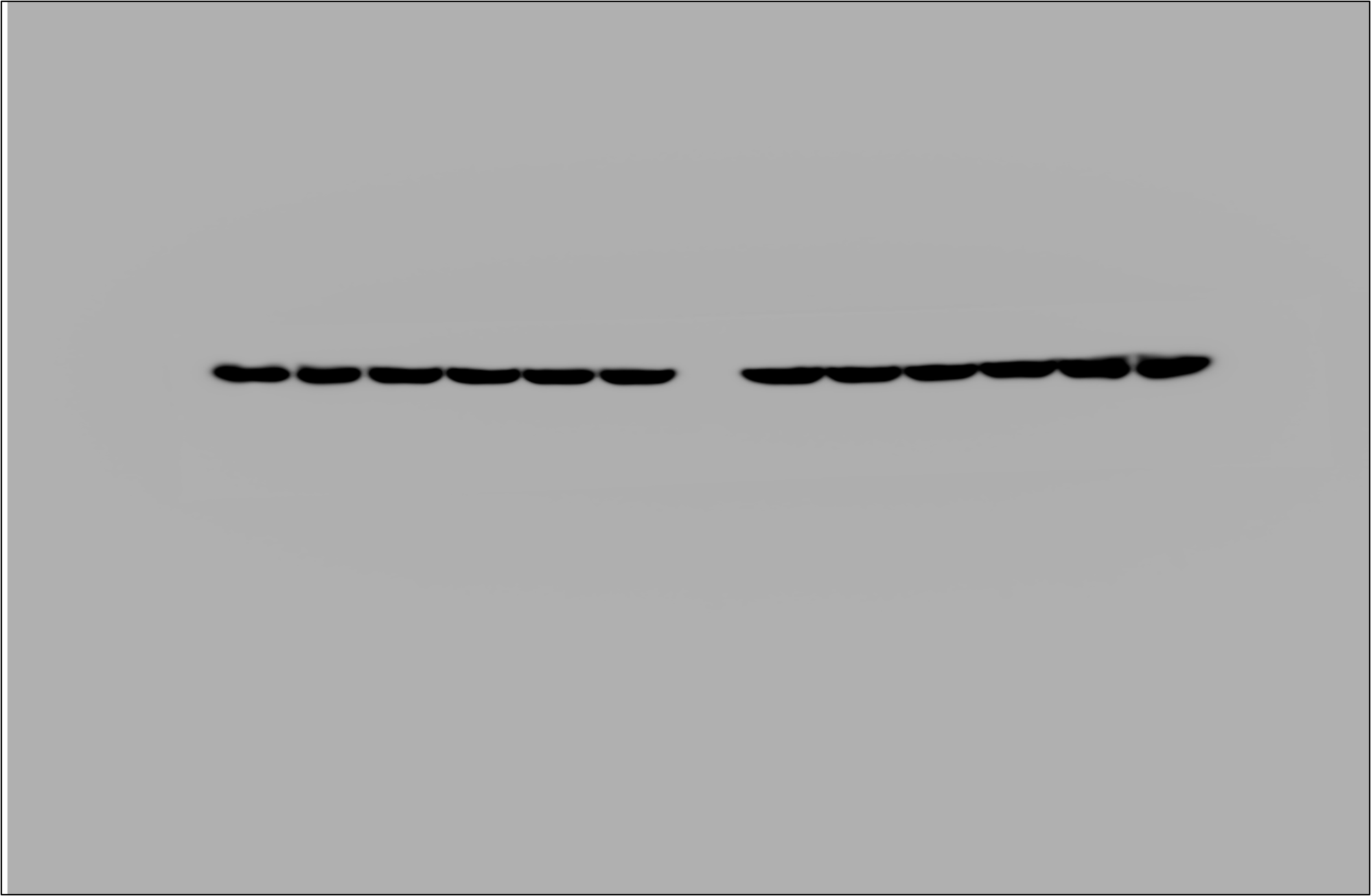

Supplement: Figure 7—source data 2. [file elife-98357-fig7-data2.zip › Figure 7-source data 2/7H-WCL-Actin-1.tif]

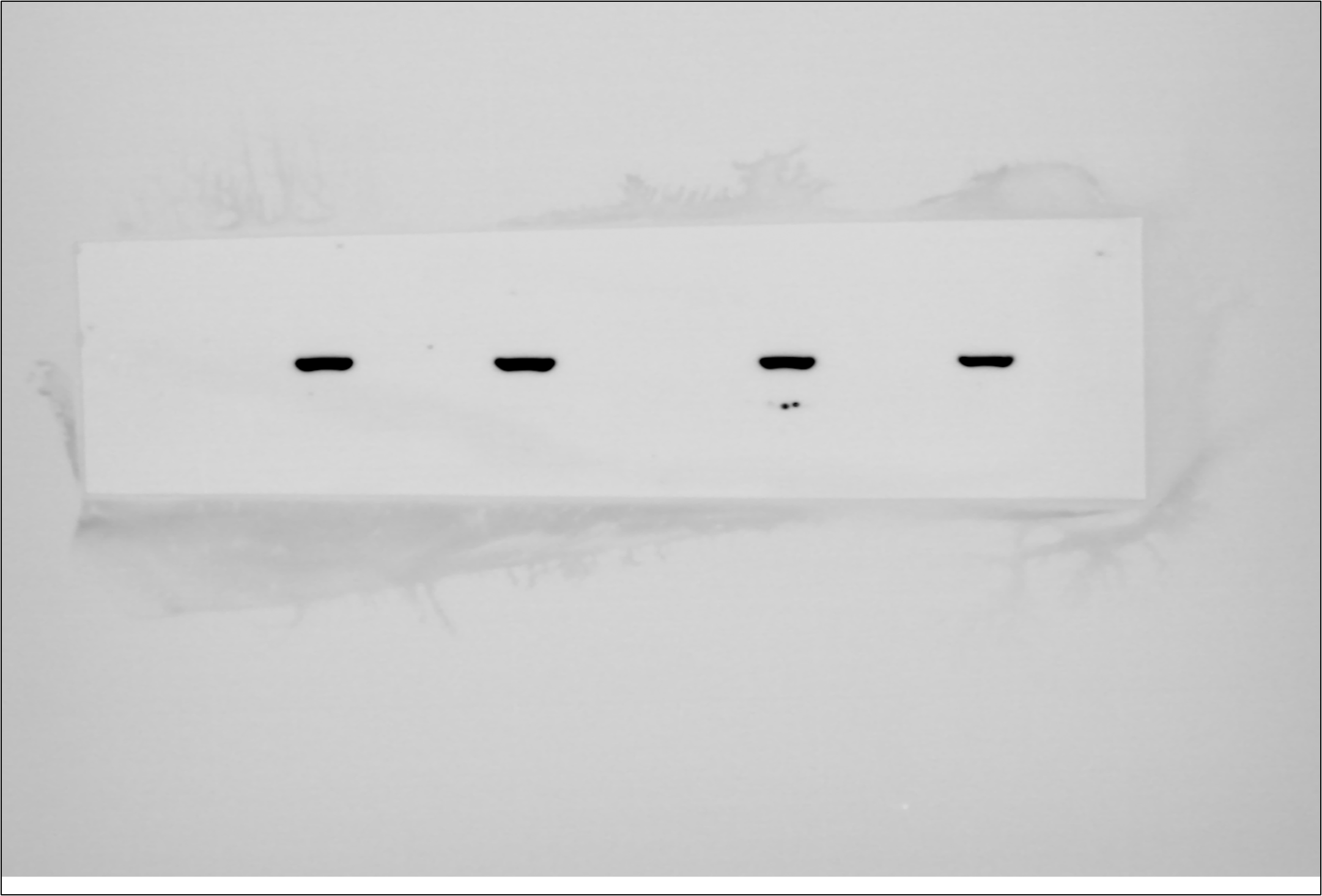

Supplement: Figure 7—source data 2. [file elife-98357-fig7-data2.zip › Figure 7-source data 2/7H-WCL-Flag-1.tif]

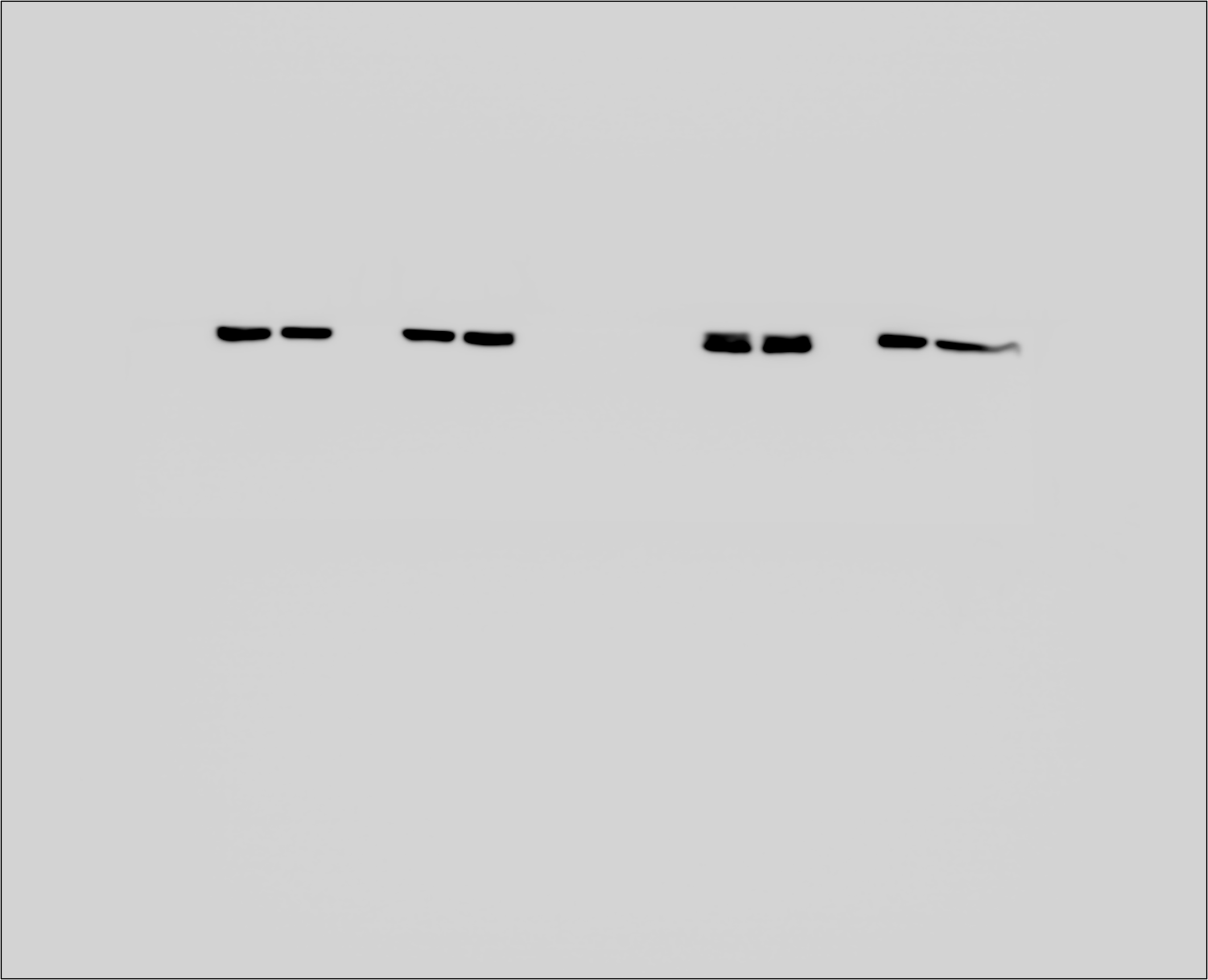

Supplement: Figure 7—source data 2. [file elife-98357-fig7-data2.zip › Figure 7-source data 2/7H-WCL-HA-1.tif]

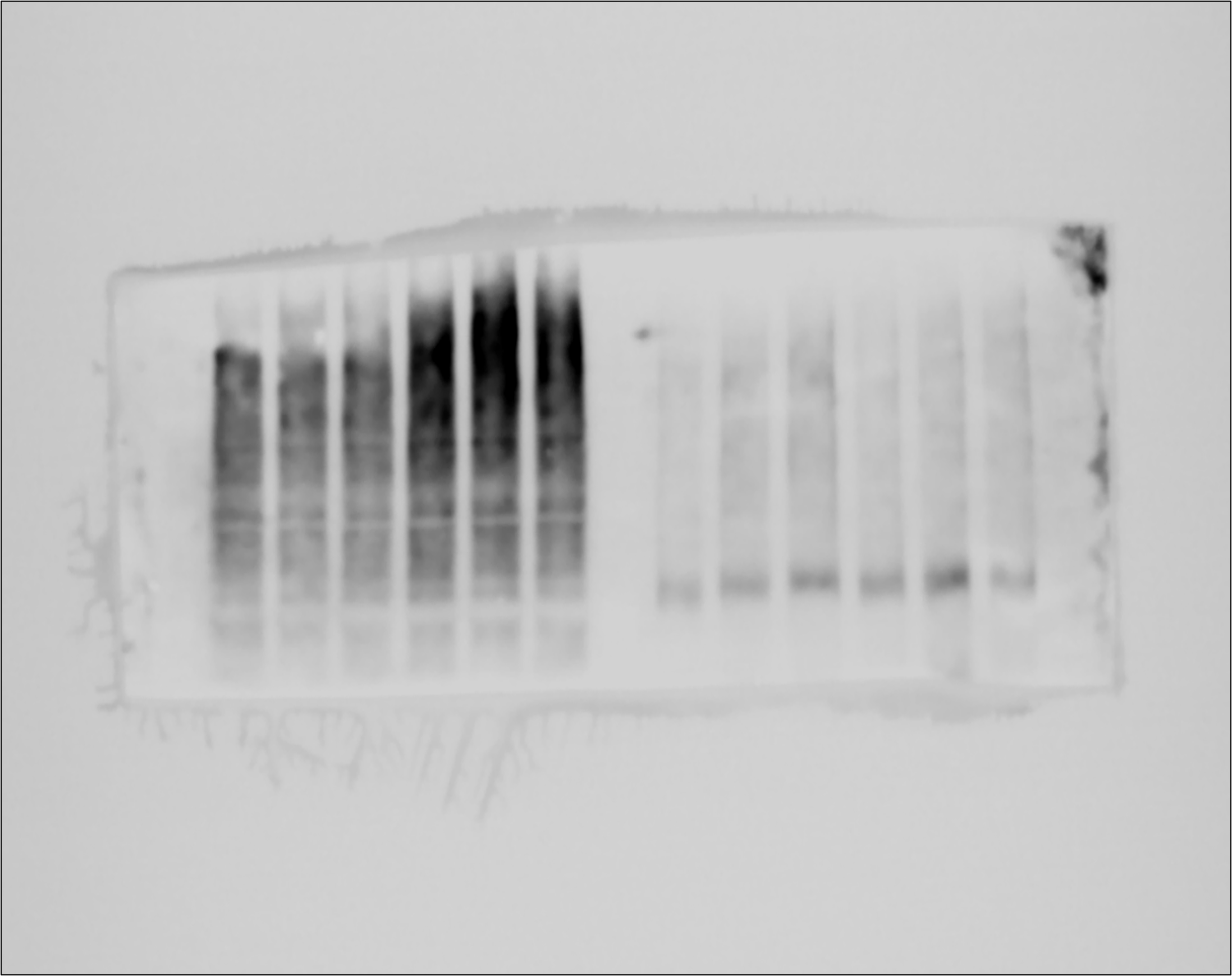

Supplement: Figure 7—source data 2. [file elife-98357-fig7-data2.zip › Figure 7-source data 2/7H-WCL-TBK1-HA-Ub-1.tif]

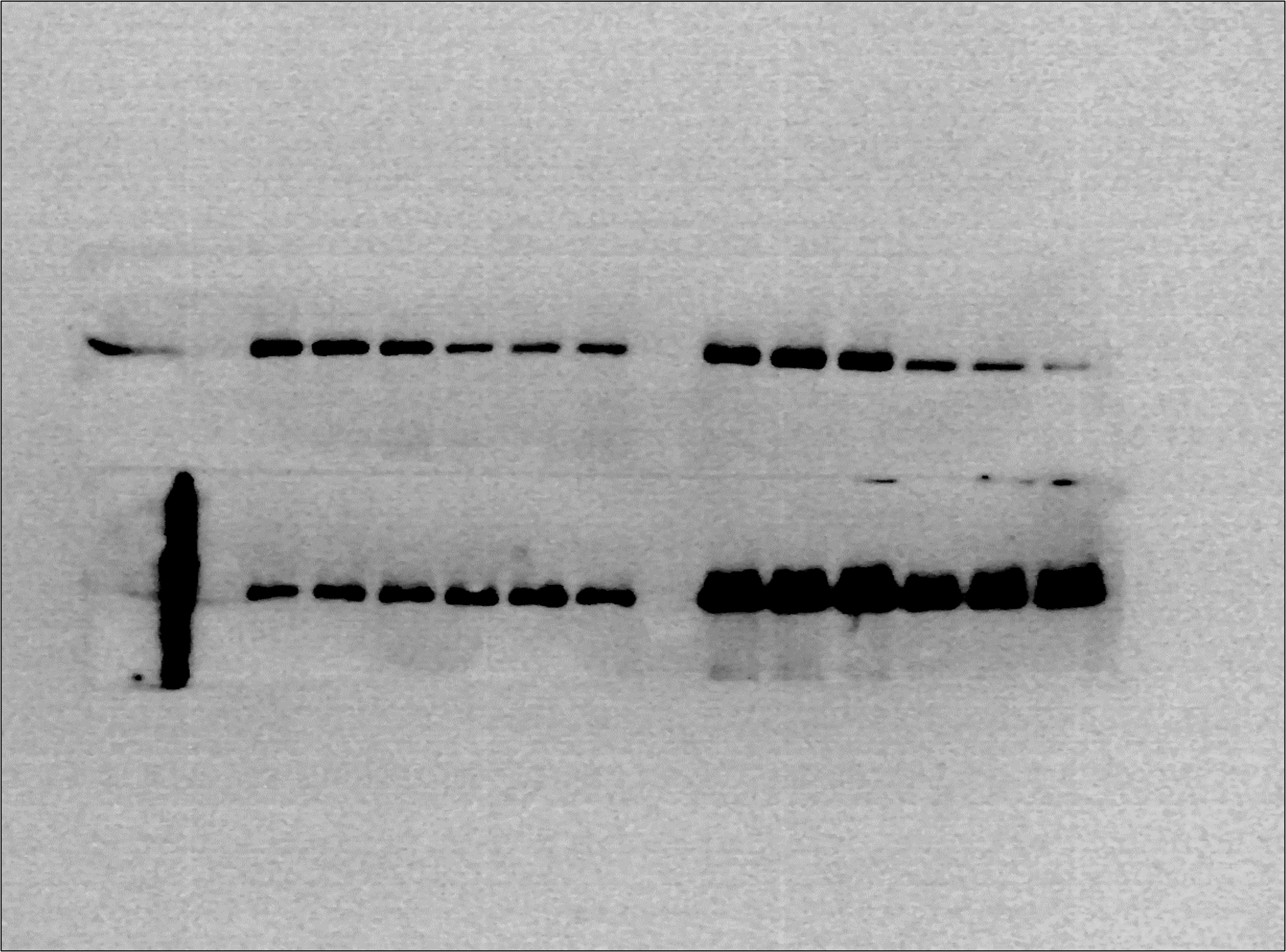

Supplement: Figure 7—source data 2. [file elife-98357-fig7-data2.zip › Figure 7-source data 2/7H-WCL-TBK1-Myc-1.tif]

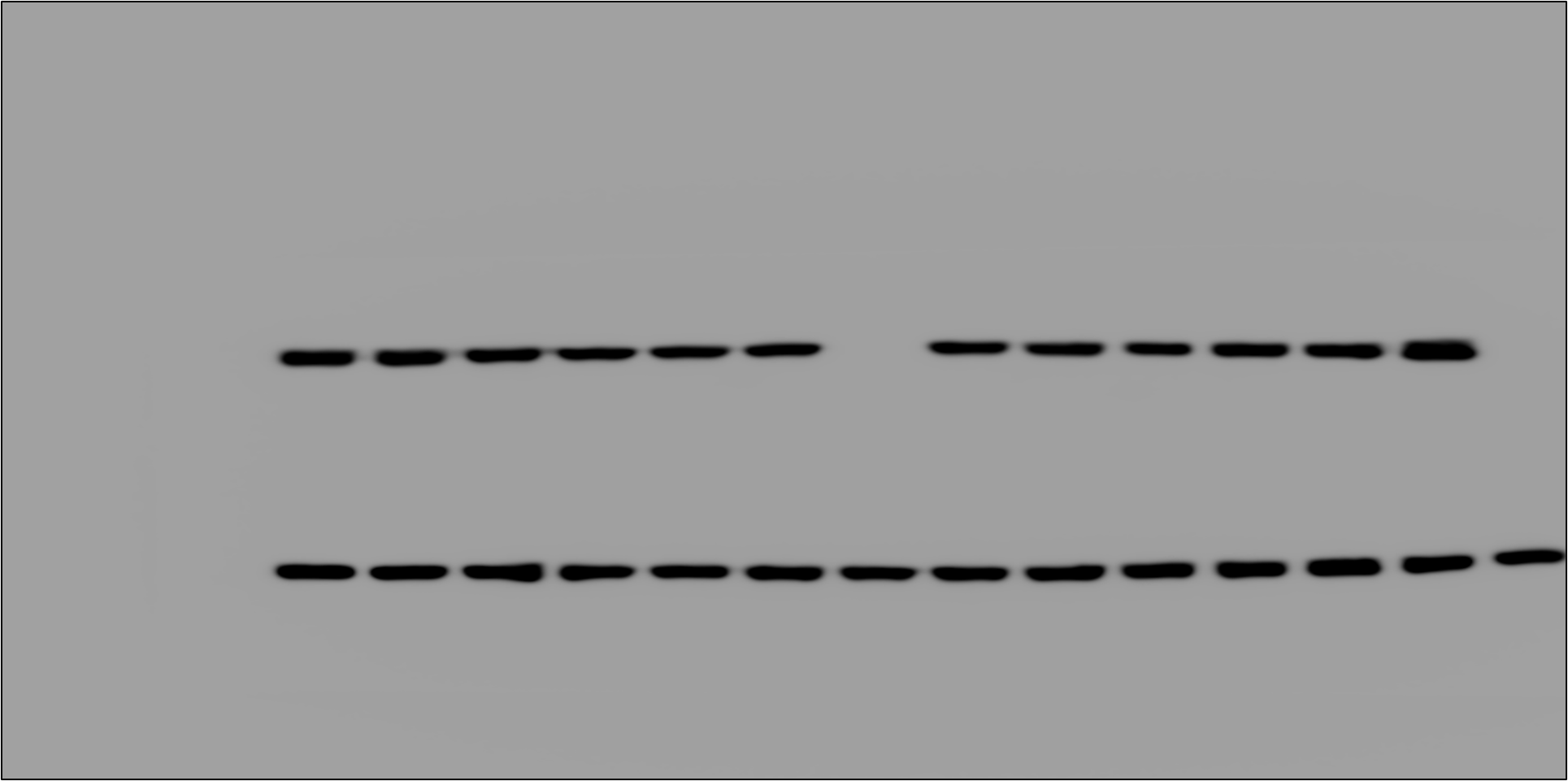

Supplement: Figure 7—source data 2. [file elife-98357-fig7-data2.zip › Figure 7-source data 2/7I-Actin-1.tif]

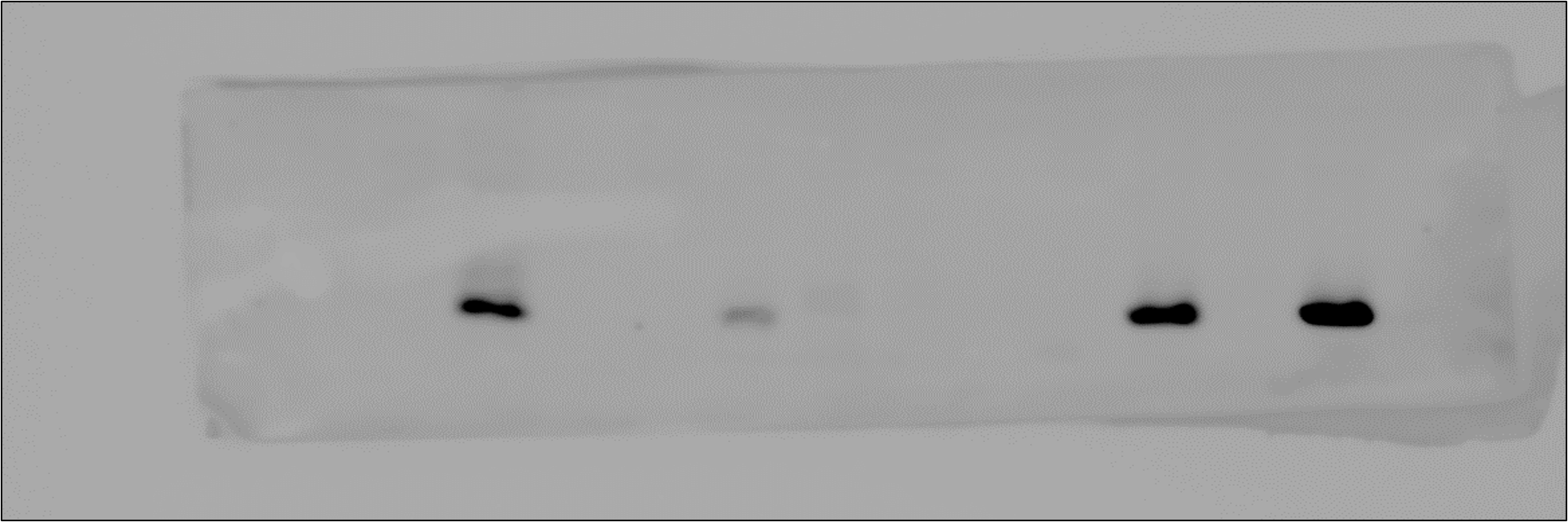

Supplement: Figure 7—source data 2. [file elife-98357-fig7-data2.zip › Figure 7-source data 2/7I-HA-1.tif]

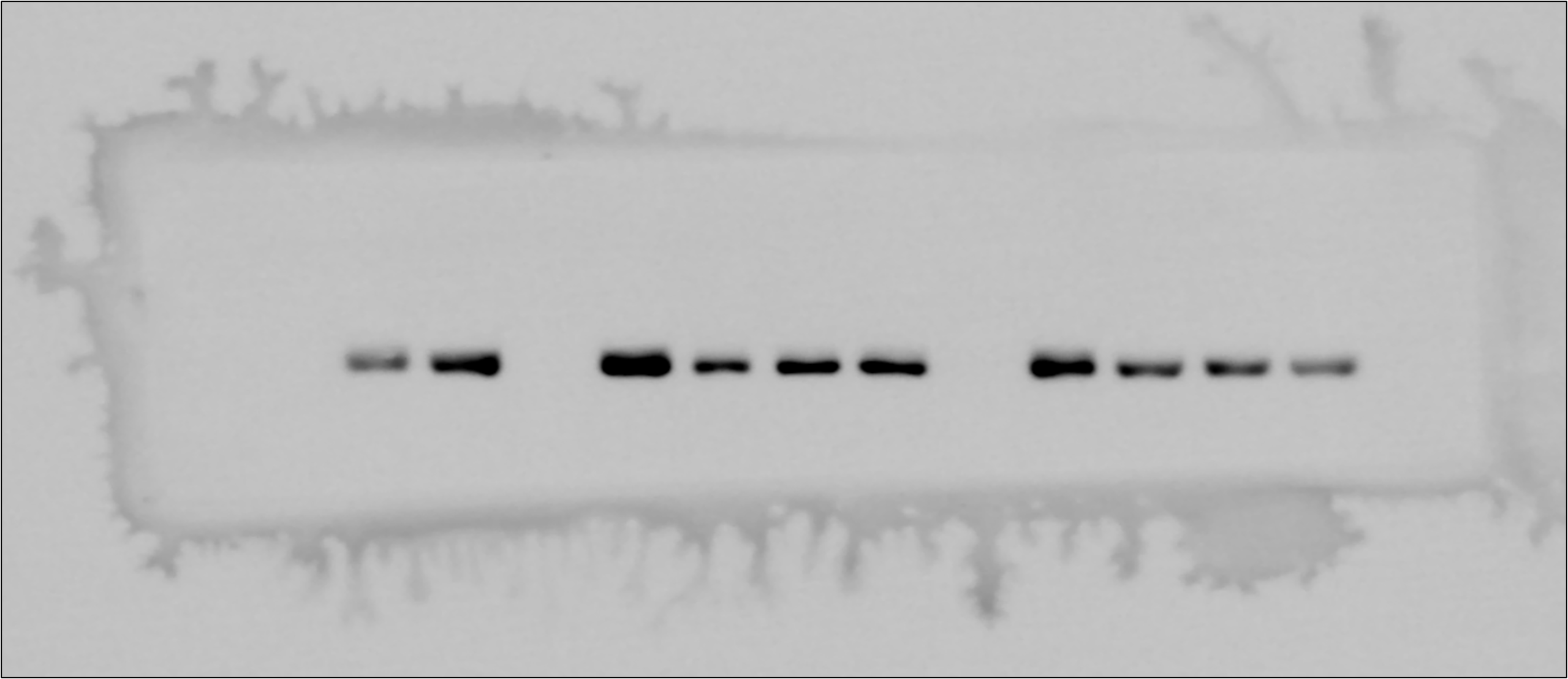

Supplement: Figure 7—source data 2. [file elife-98357-fig7-data2.zip › Figure 7-source data 2/7I-Myc-1.tif]

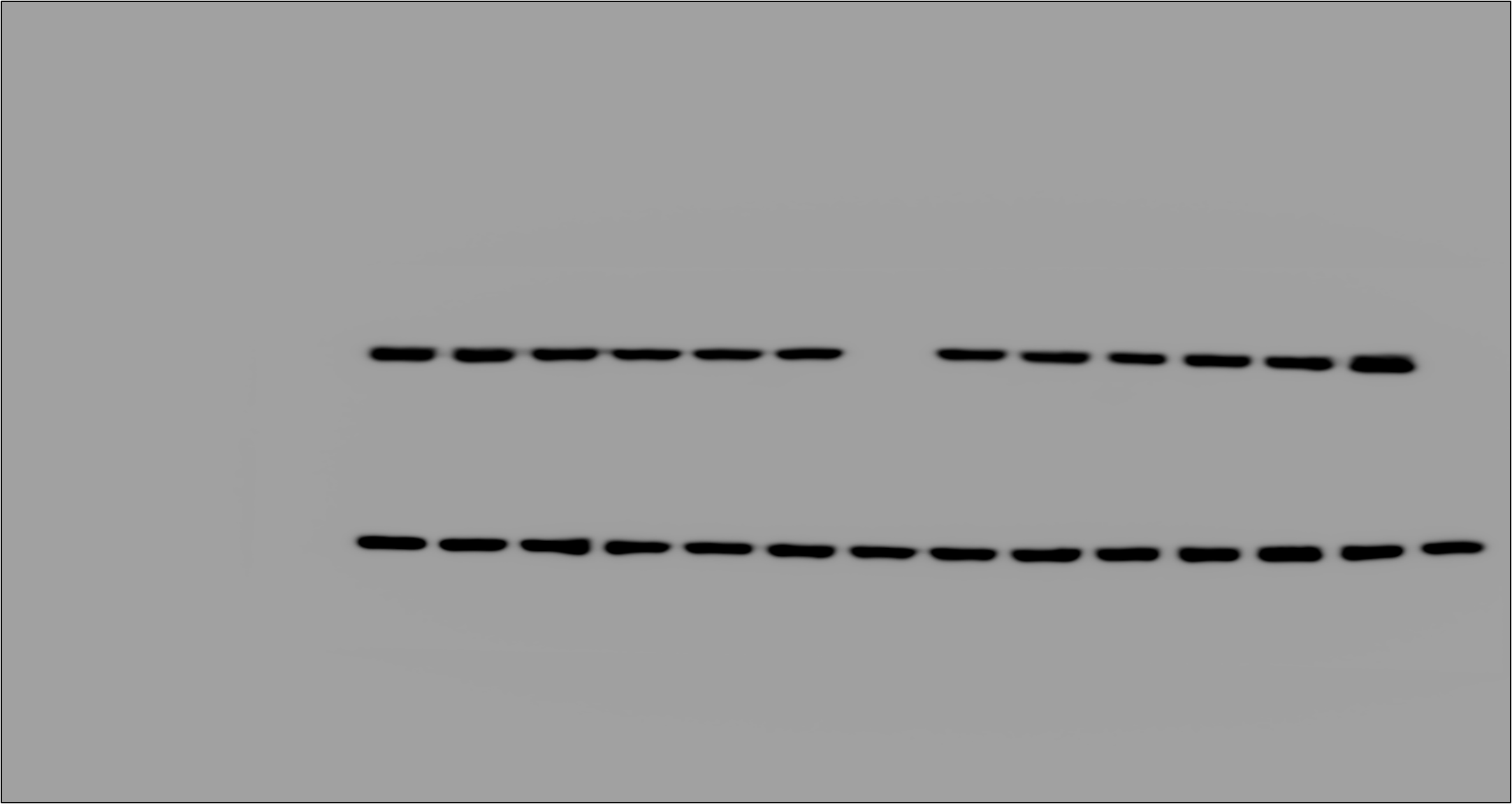

Supplement: Figure 7—source data 2. [file elife-98357-fig7-data2.zip › Figure 7-source data 2/7M-Actin-1.tif]

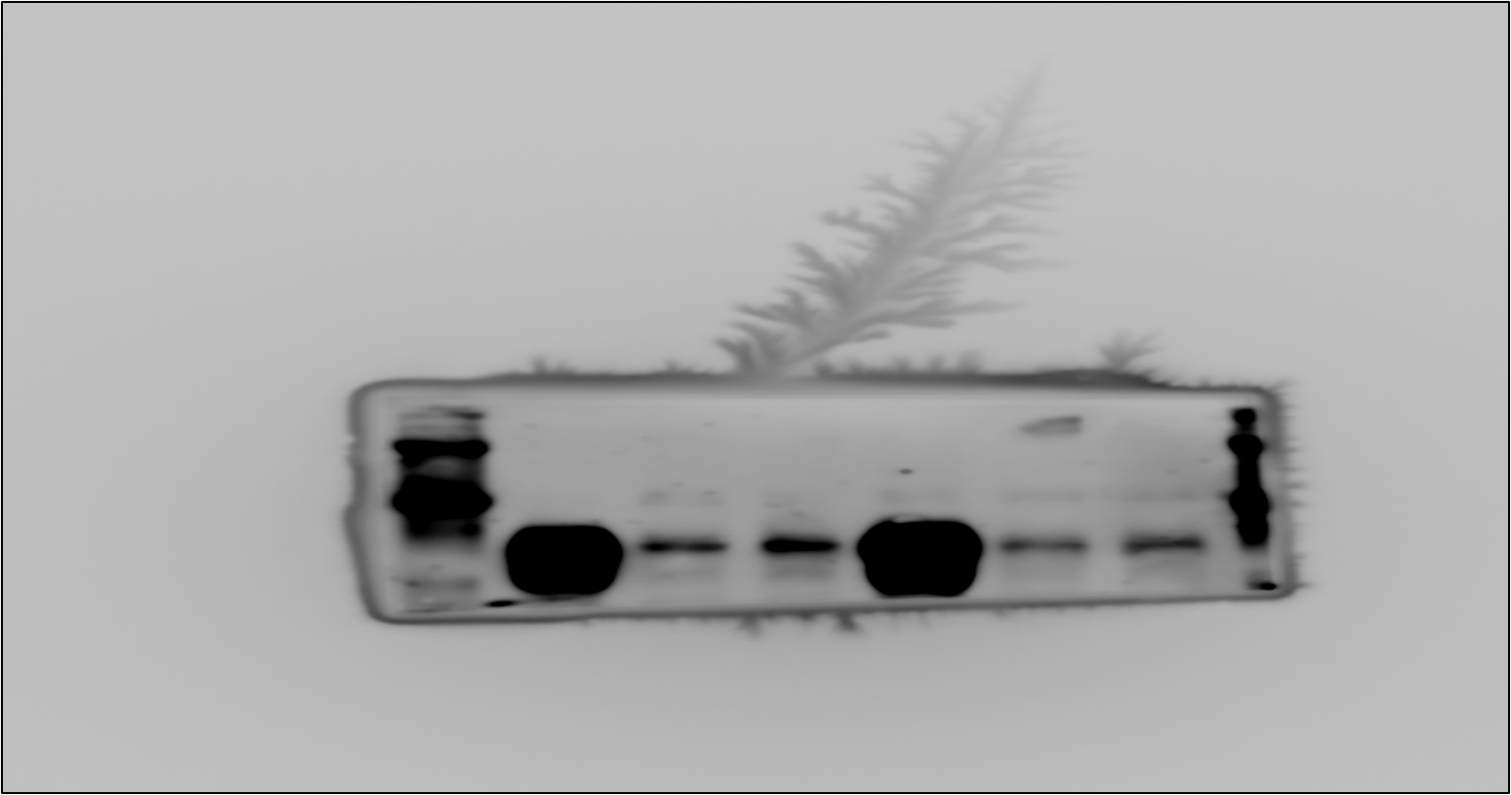

Supplement: Figure 7—source data 2. [file elife-98357-fig7-data2.zip › Figure 7-source data 2/7M-G-1.tif]

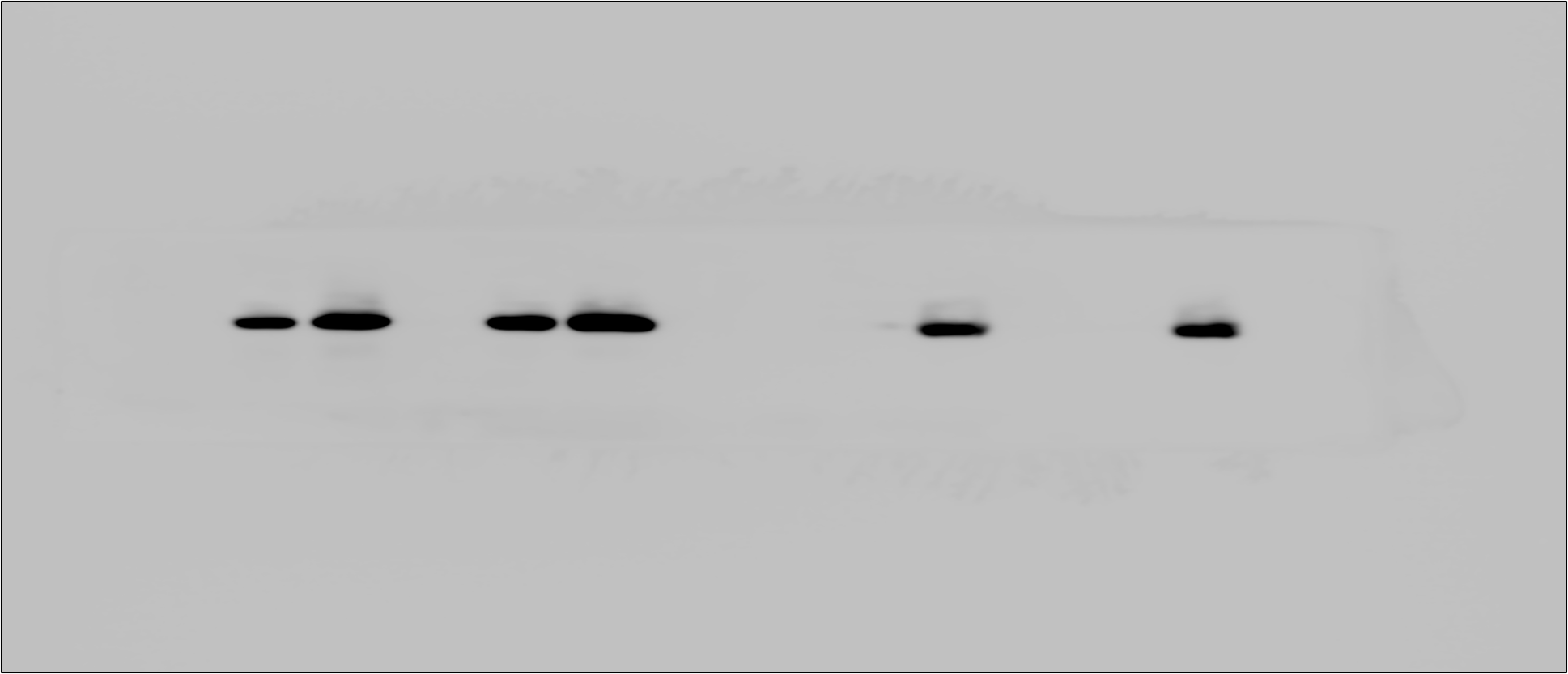

Supplement: Figure 7—source data 2. [file elife-98357-fig7-data2.zip › Figure 7-source data 2/7M-HA-1.tif]

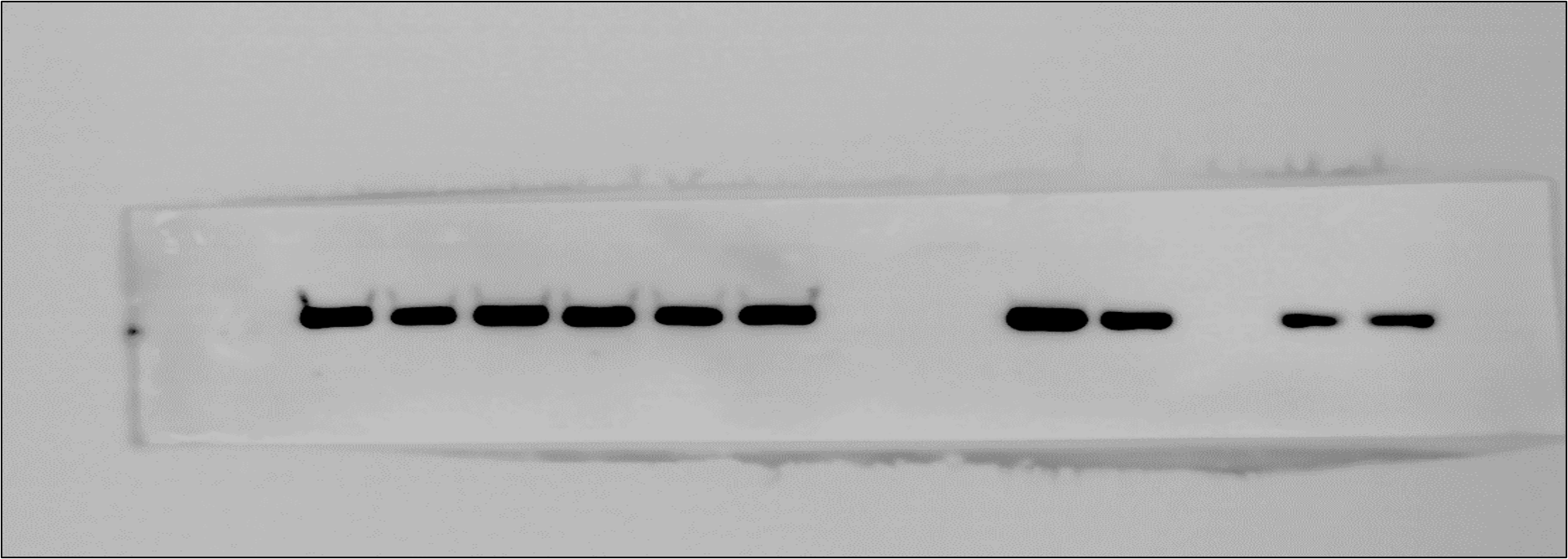

Supplement: Figure 7—source data 2. [file elife-98357-fig7-data2.zip › Figure 7-source data 2/7M-Myc-1.tif]

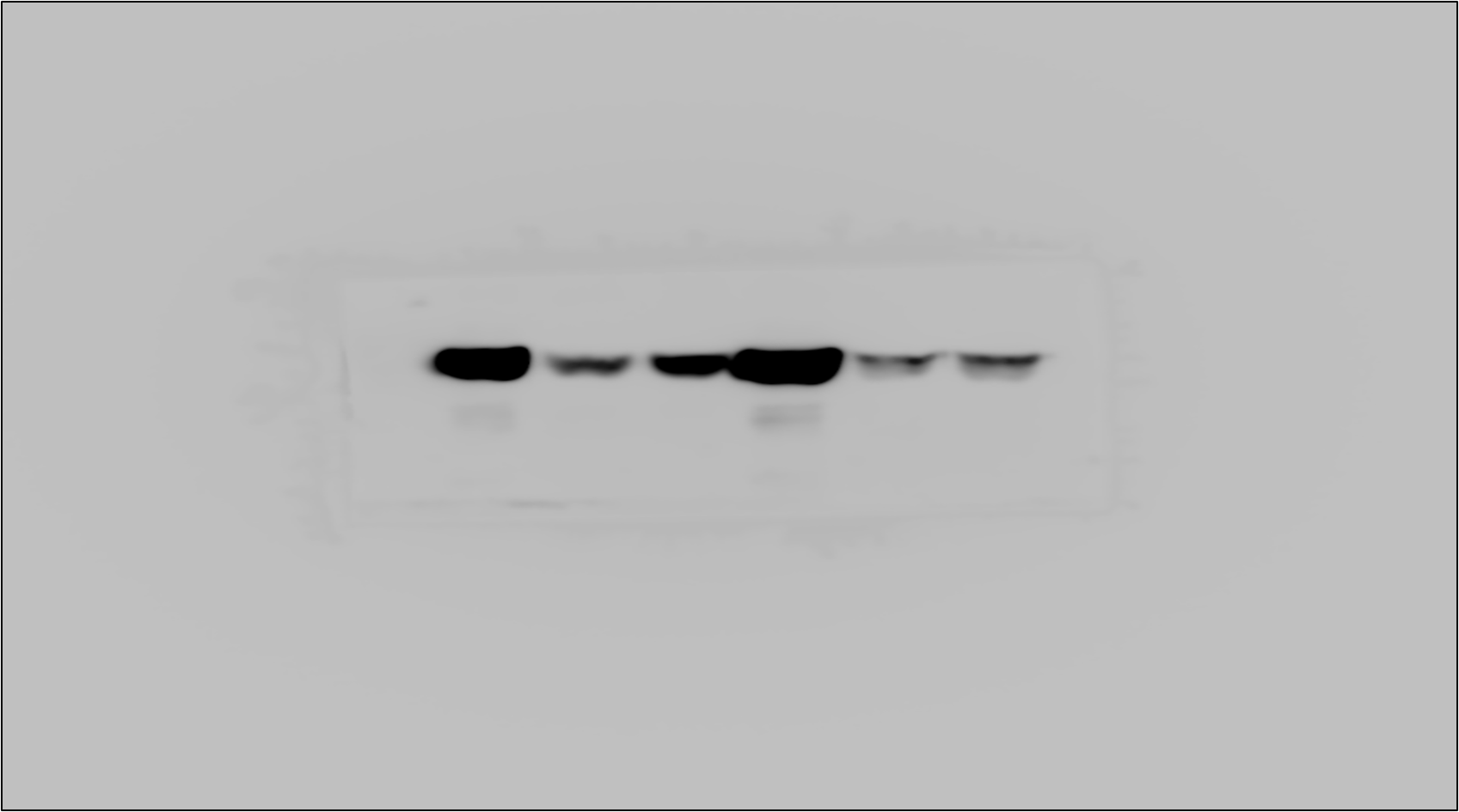

Supplement: Figure 7—source data 2. [file elife-98357-fig7-data2.zip › Figure 7-source data 2/7M-N-1.tif]

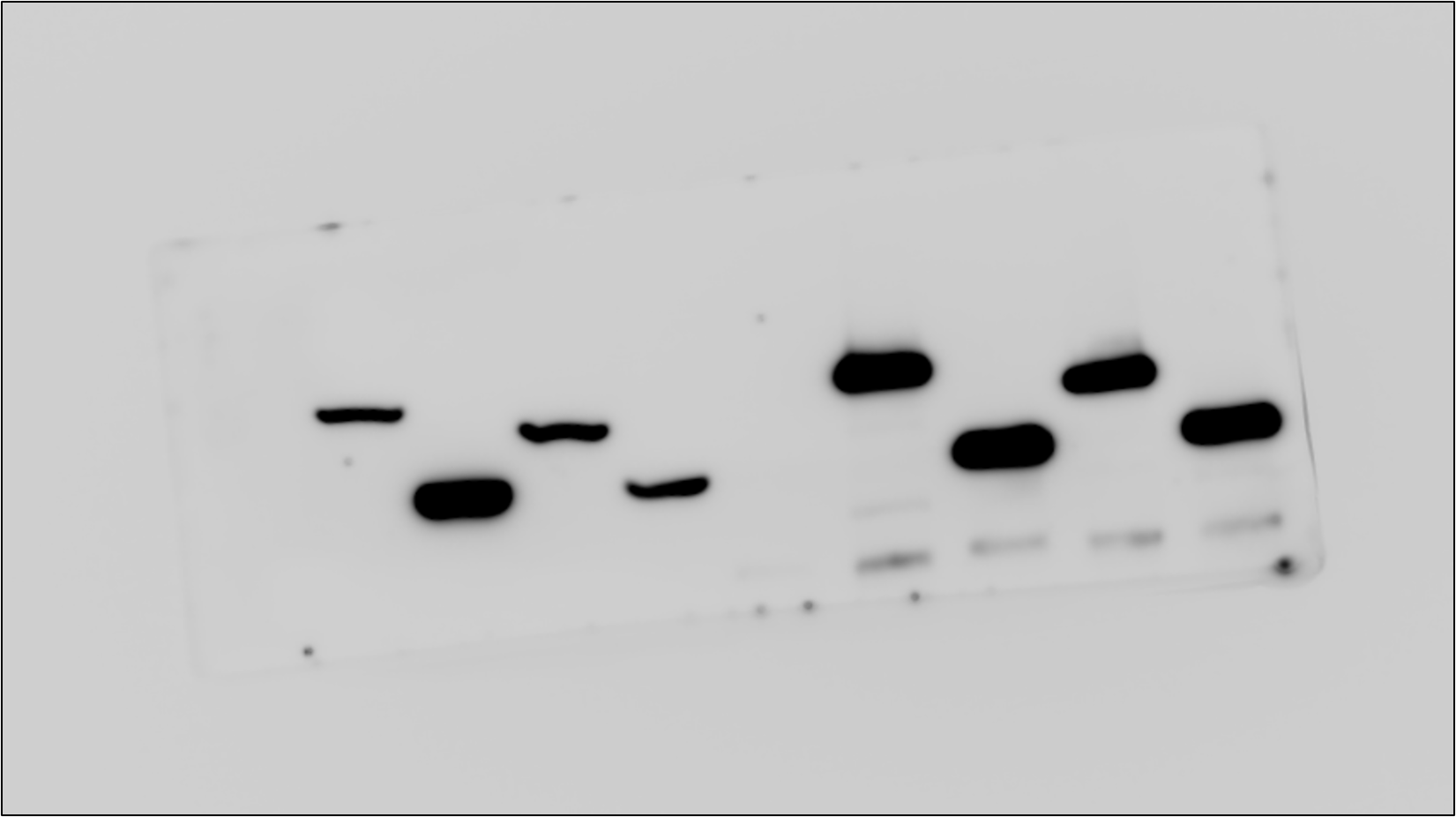

Supplement: Figure 7—source data 2. [file elife-98357-fig7-data2.zip › Figure 7-source data 2/7Q-Input-CDK2-HA-1.tif]

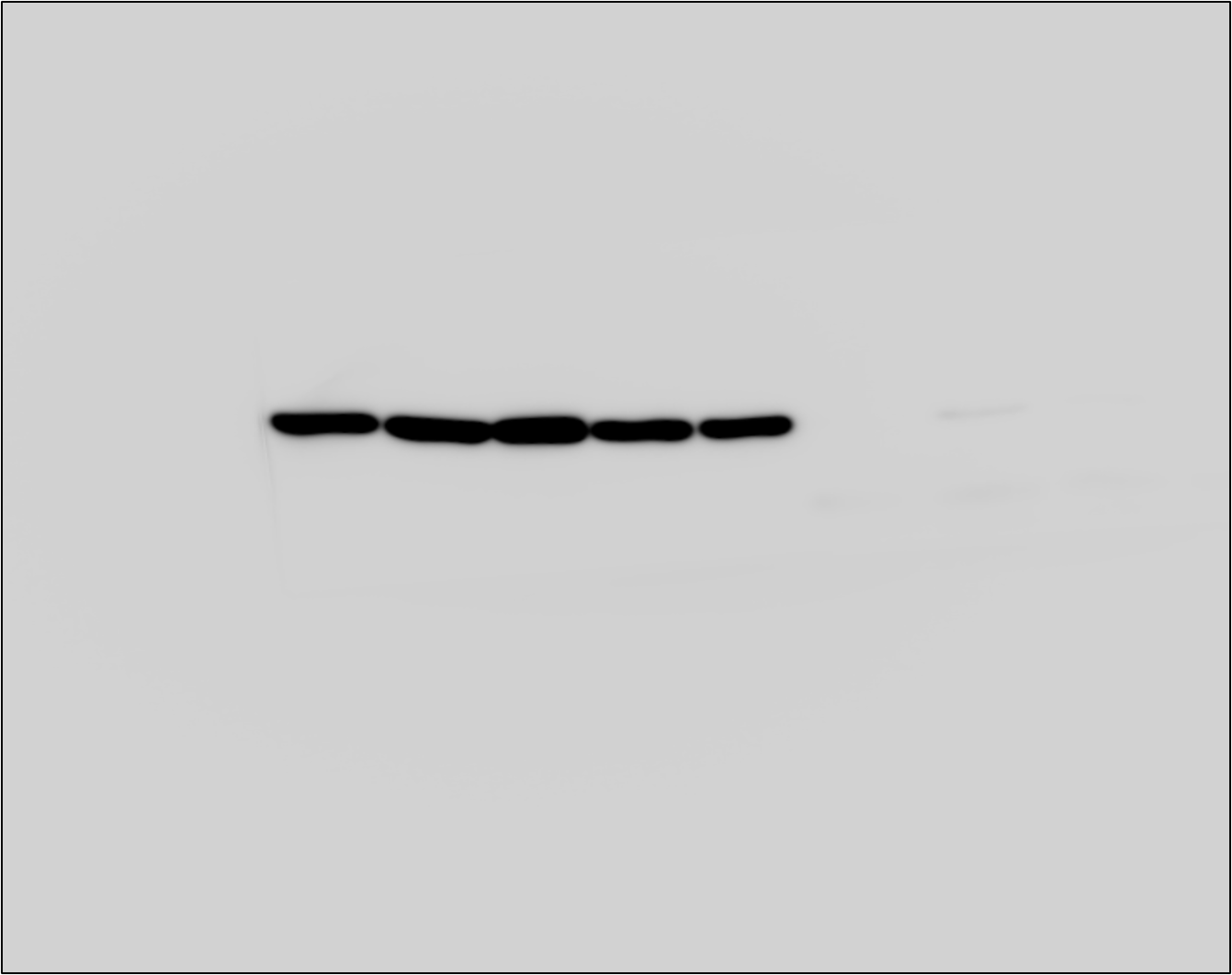

Supplement: Figure 7—source data 2. [file elife-98357-fig7-data2.zip › Figure 7-source data 2/7Q-Input-CDK2-Myc-1.tif]

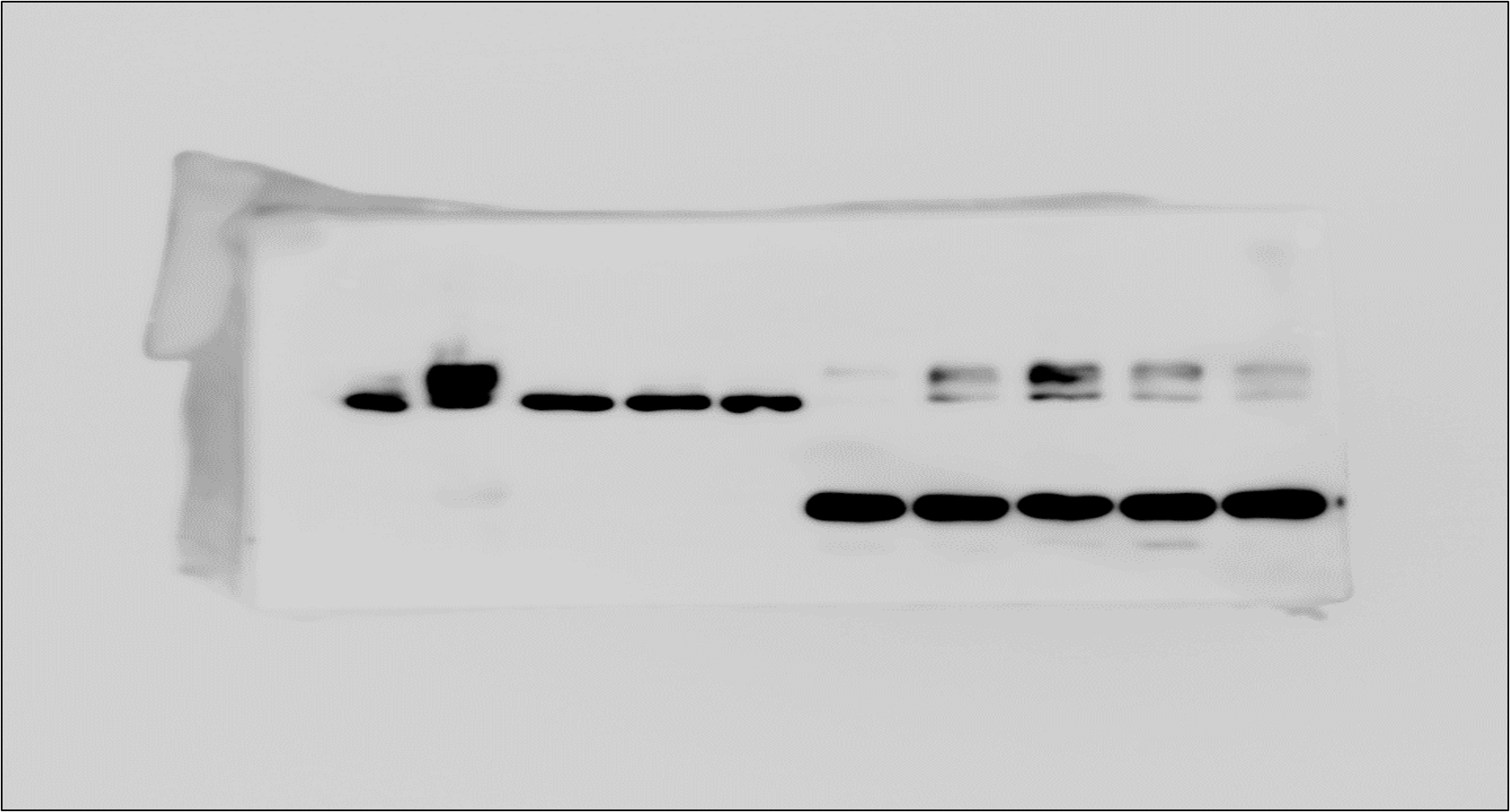

Supplement: Figure 7—source data 2. [file elife-98357-fig7-data2.zip › Figure 7-source data 2/7Q-Input-HA-1.tif]

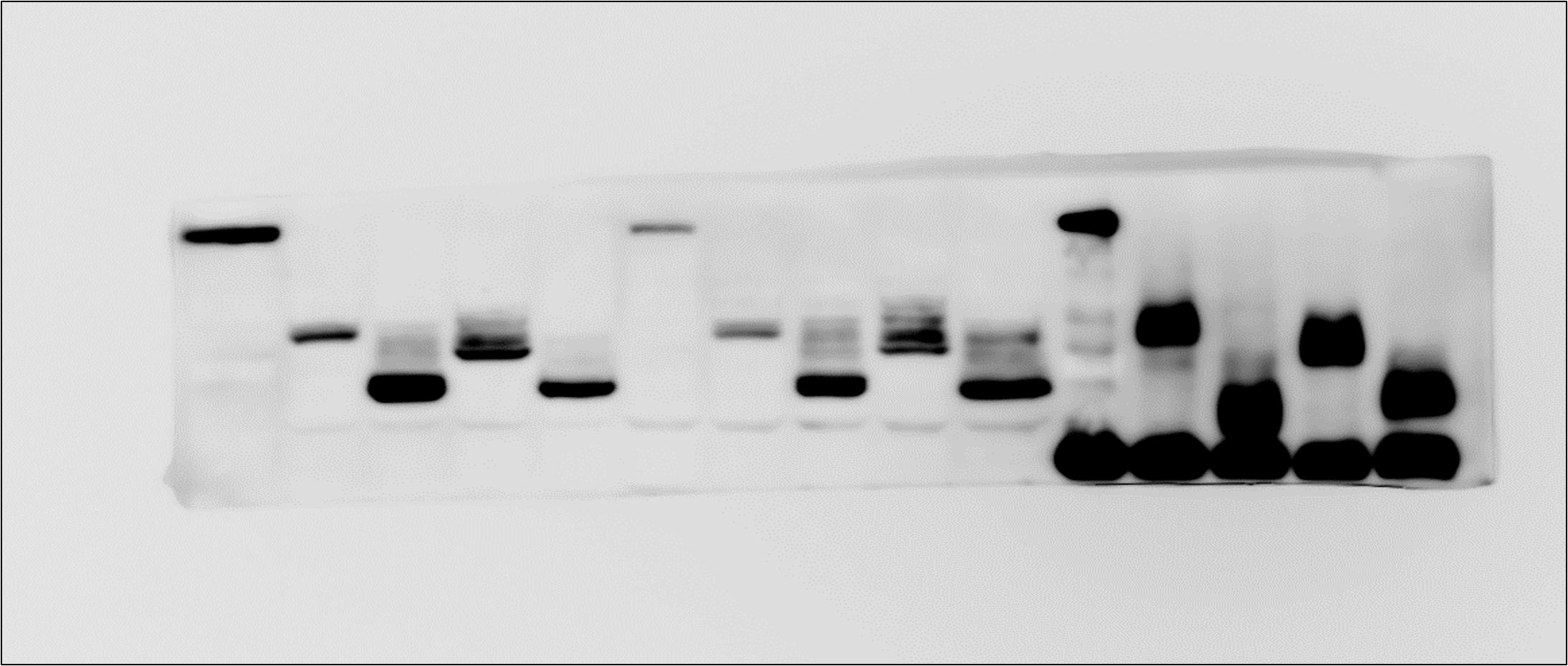

Supplement: Figure 7—source data 2. [file elife-98357-fig7-data2.zip › Figure 7-source data 2/7Q-Input-TBK1-Myc-1.tif]

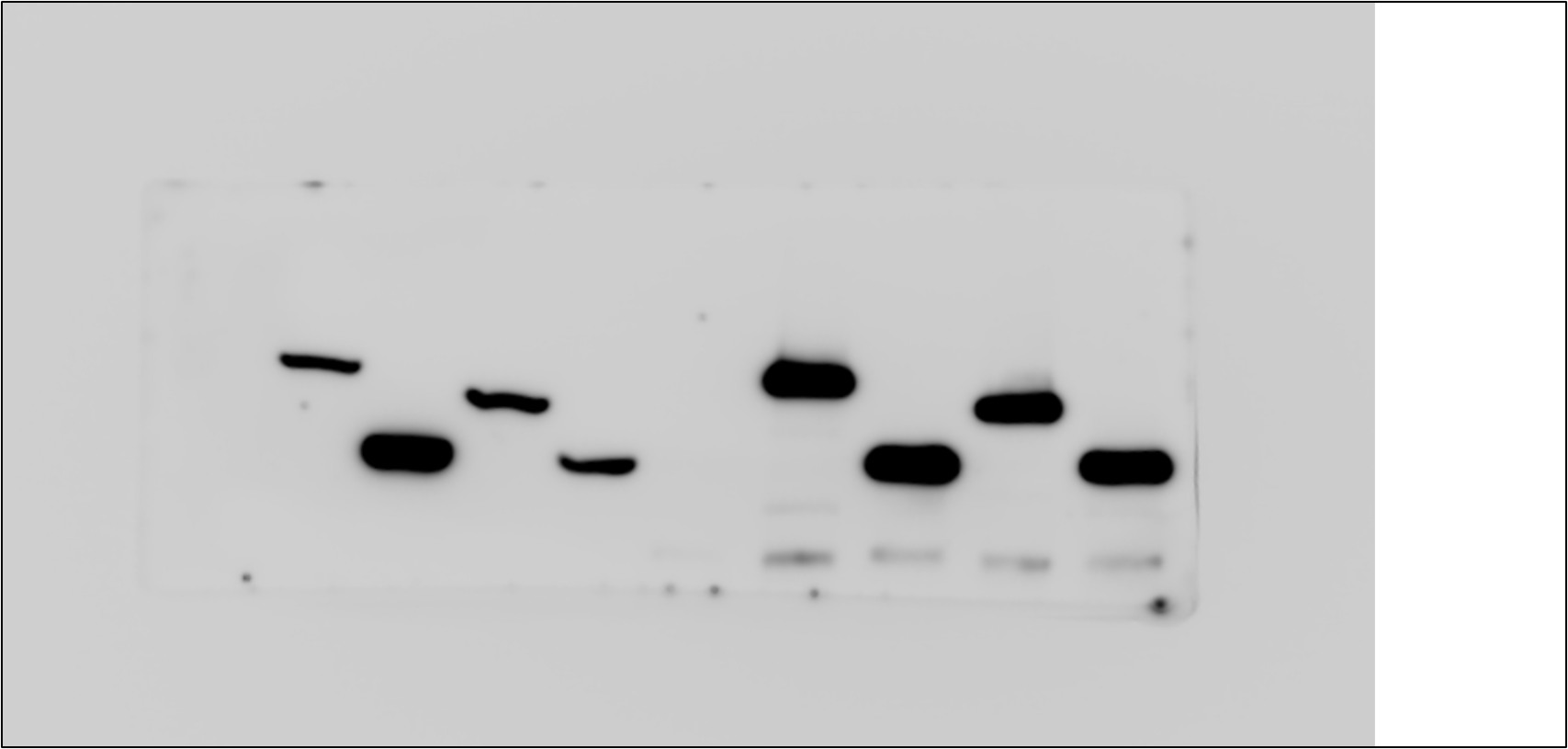

Supplement: Figure 7—source data 2. [file elife-98357-fig7-data2.zip › Figure 7-source data 2/7Q-IP-CDK2-HA-1.tif]

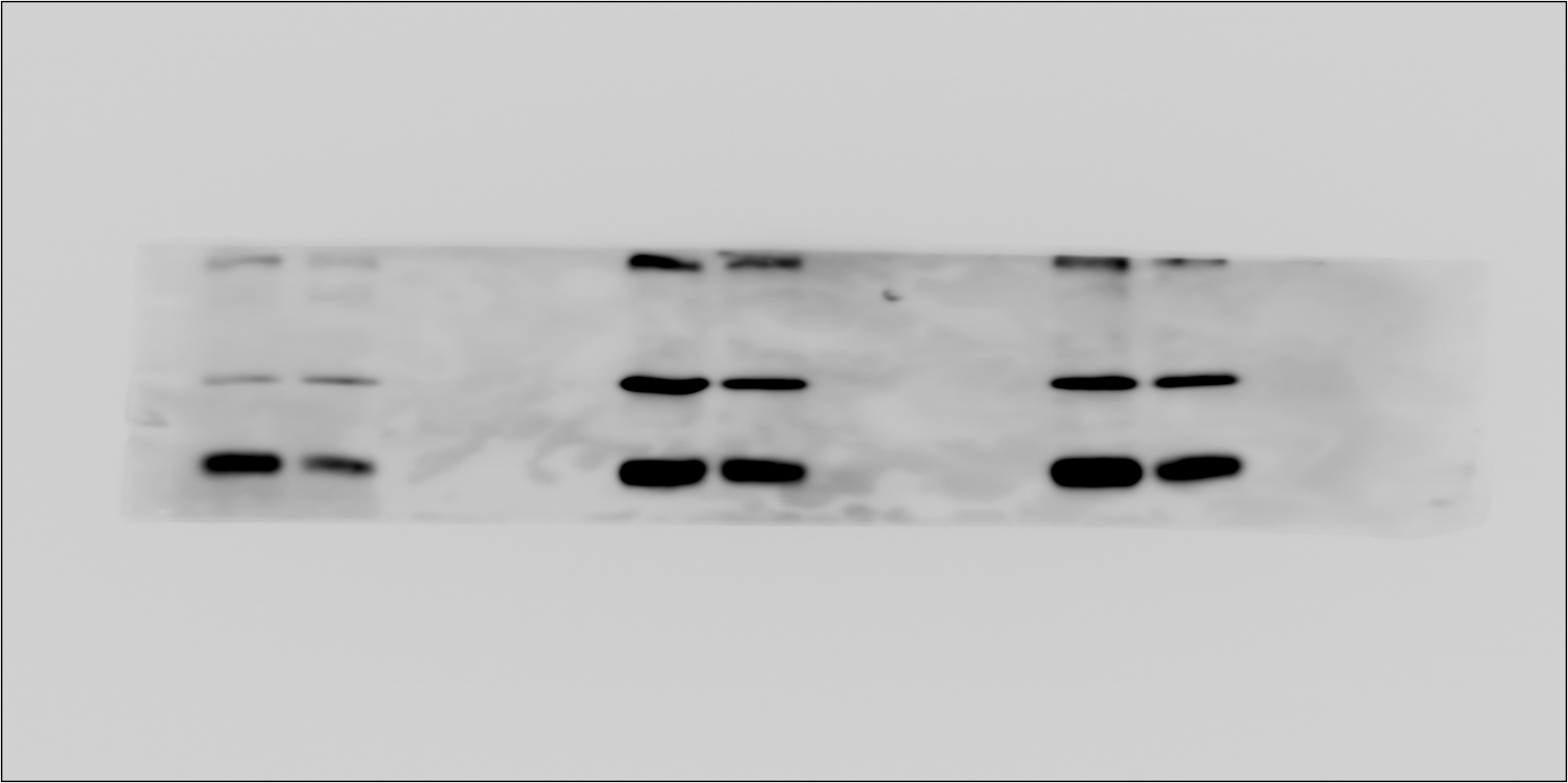

Supplement: Figure 7—source data 2. [file elife-98357-fig7-data2.zip › Figure 7-source data 2/7Q-IP-CDK2-Myc-1.tif]

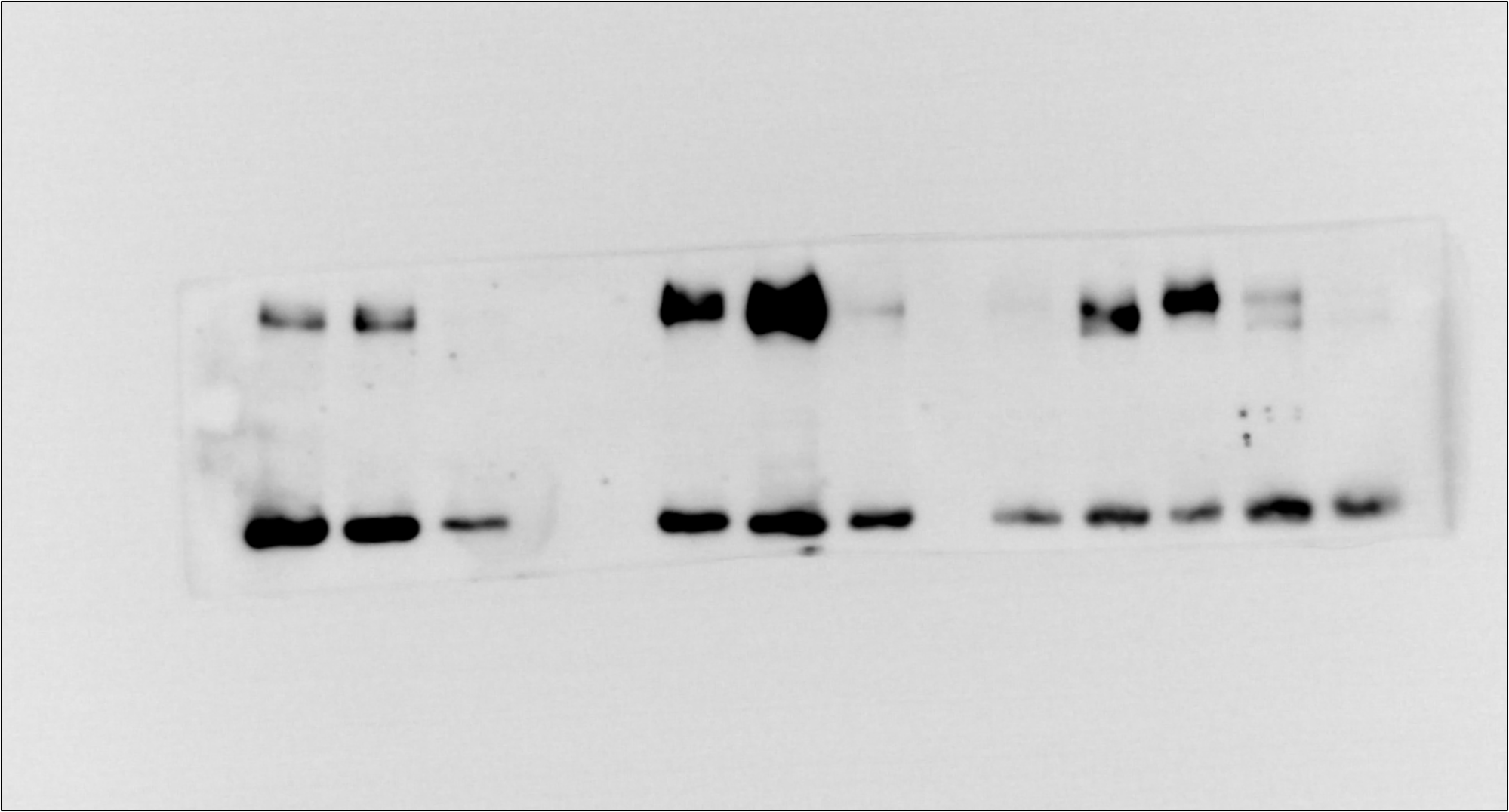

Supplement: Figure 7—source data 2. [file elife-98357-fig7-data2.zip › Figure 7-source data 2/7Q-IP-TBK1-HA-1.tif]

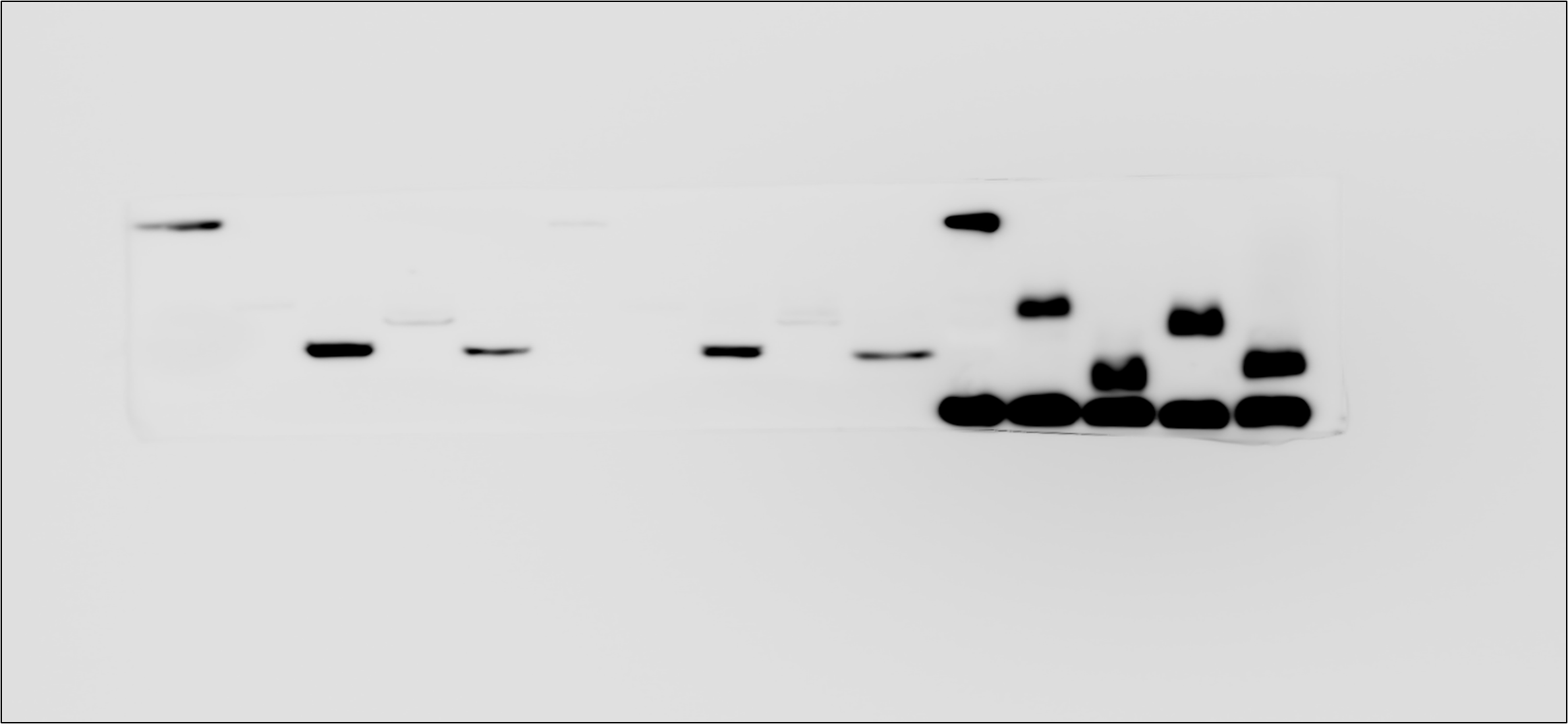

Supplement: Figure 7—source data 2. [file elife-98357-fig7-data2.zip › Figure 7-source data 2/7Q-IP-TBK1-Myc-1.tif]

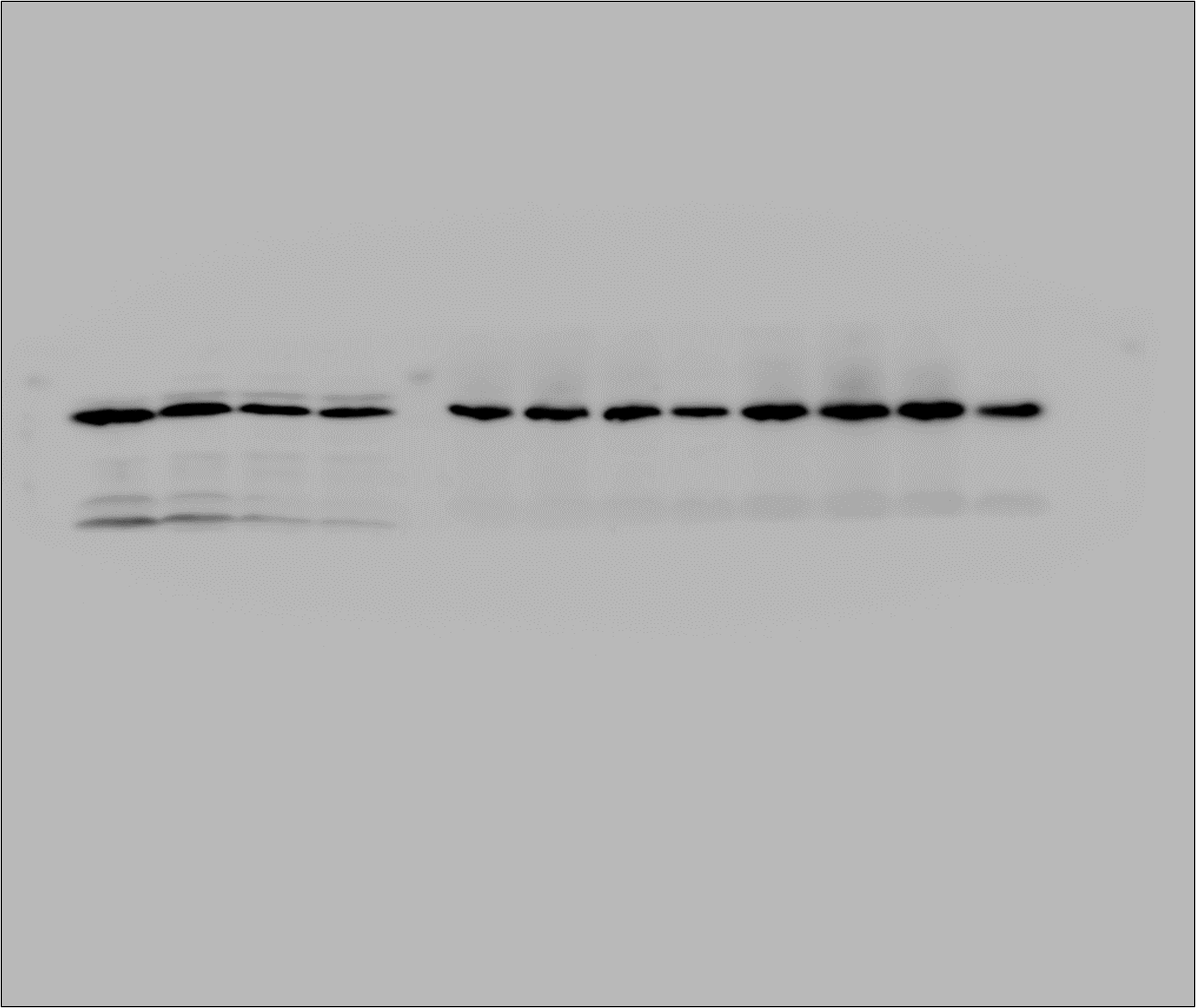

Supplement: Figure 7—source data 2. [file elife-98357-fig7-data2.zip › Figure 7-source data 2/7R-Input-Myc-1.tif]

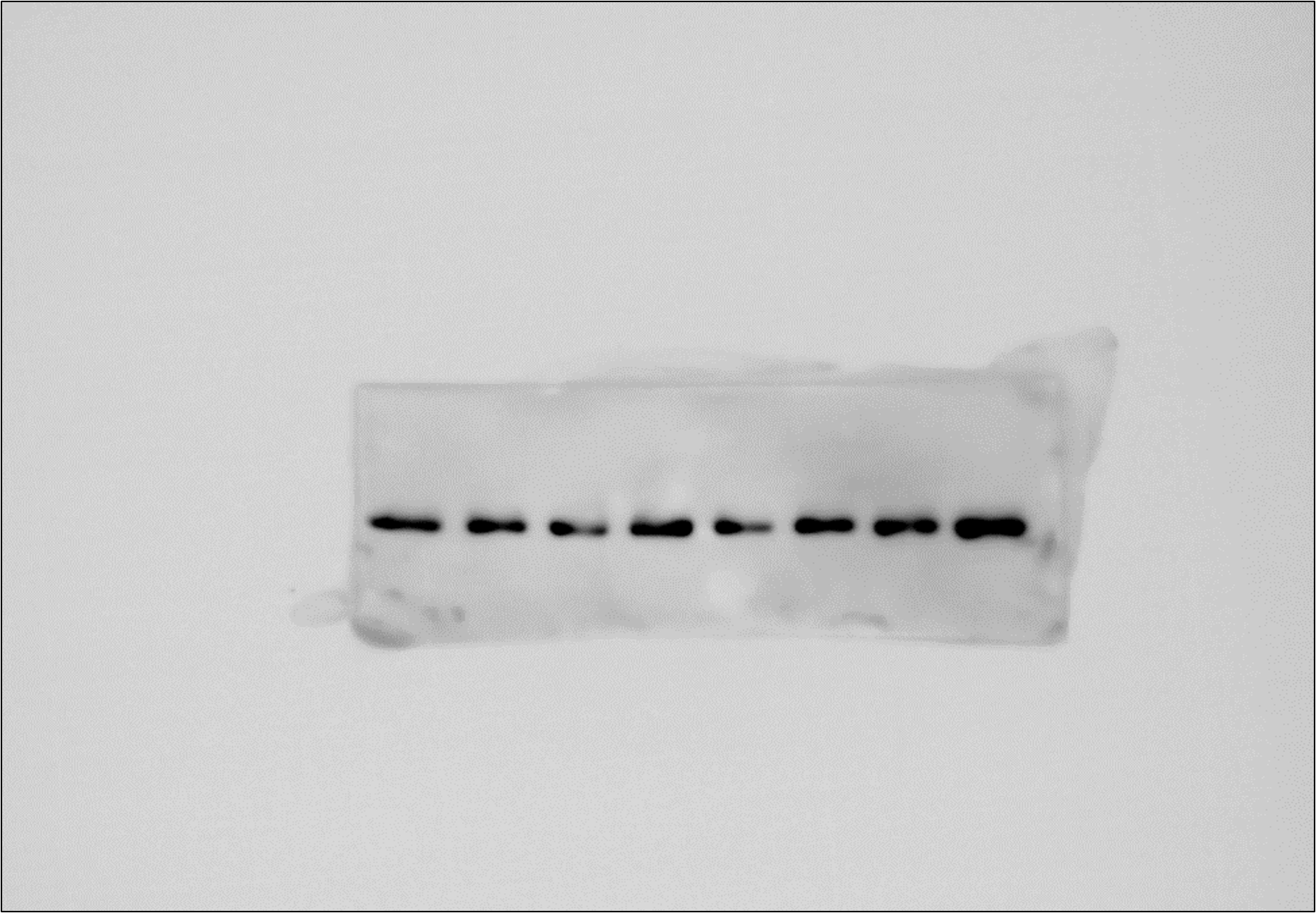

Supplement: Figure 7—source data 2. [file elife-98357-fig7-data2.zip › Figure 7-source data 2/7R-IP-Myc-1.tif]

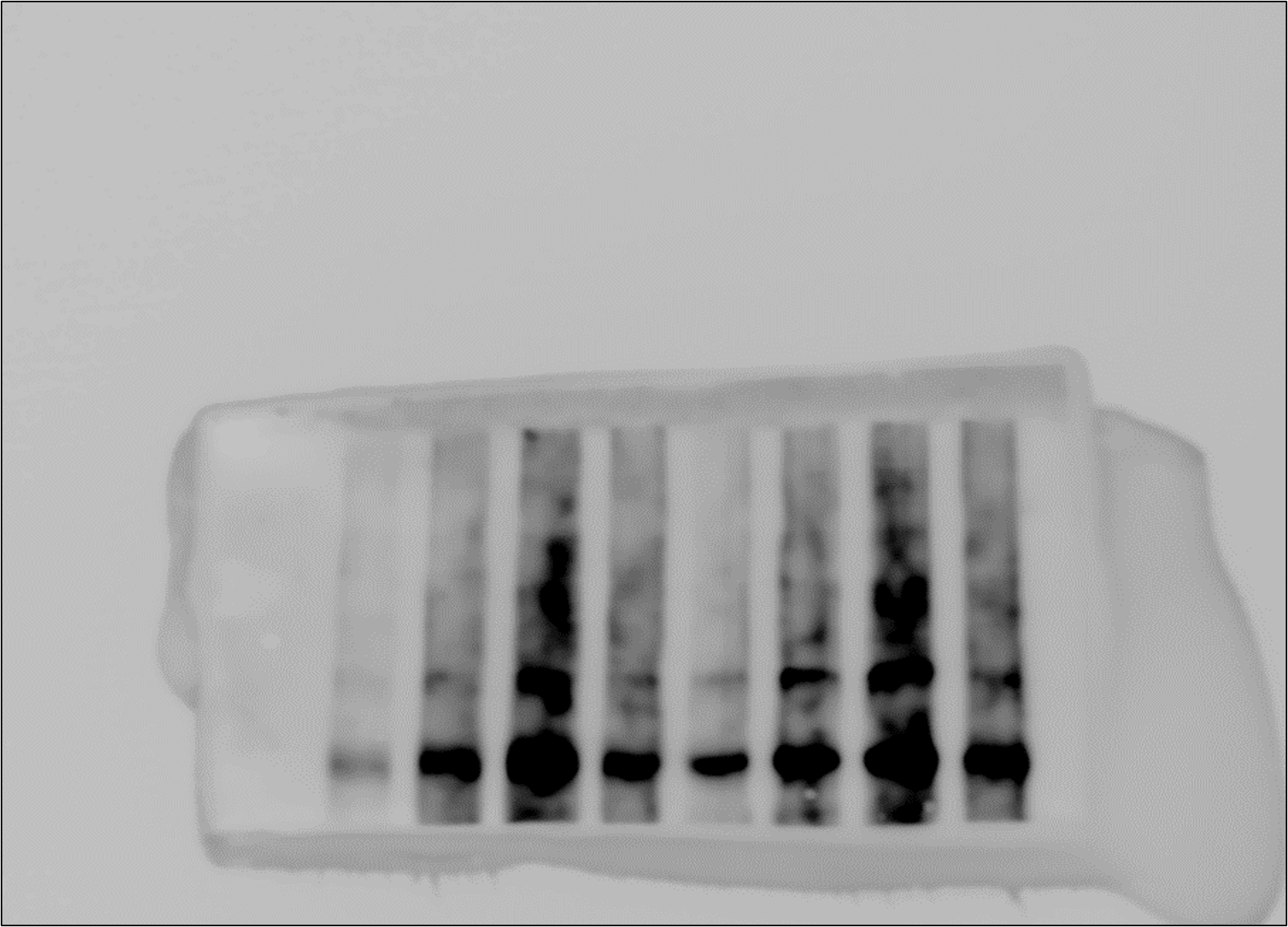

Supplement: Figure 7—source data 2. [file elife-98357-fig7-data2.zip › Figure 7-source data 2/7R-IP-TBK1-HA-Ub-1.tif]

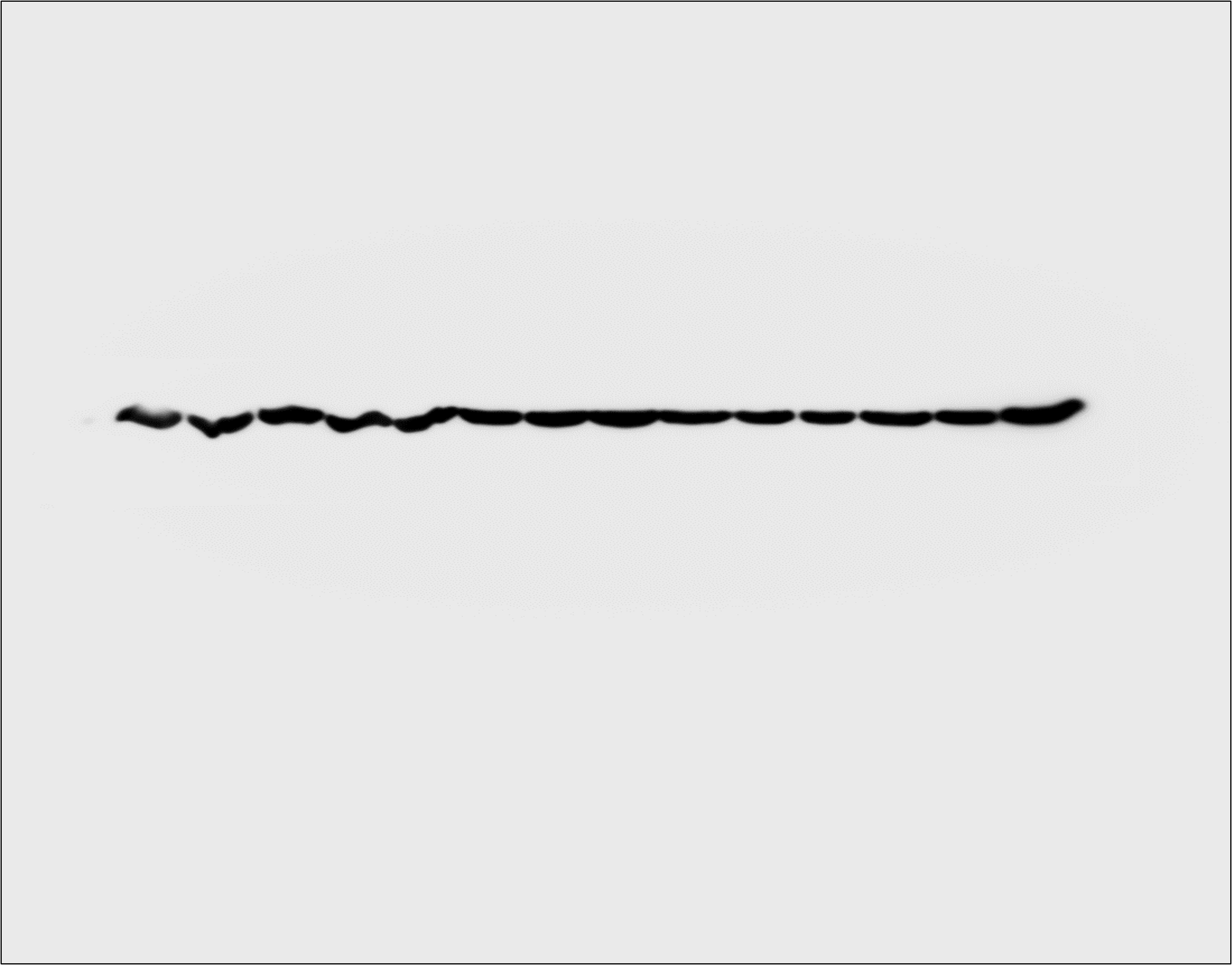

Supplement: Figure 7—source data 2. [file elife-98357-fig7-data2.zip › Figure 7-source data 2/7R-WCL-Actin-1.tif]

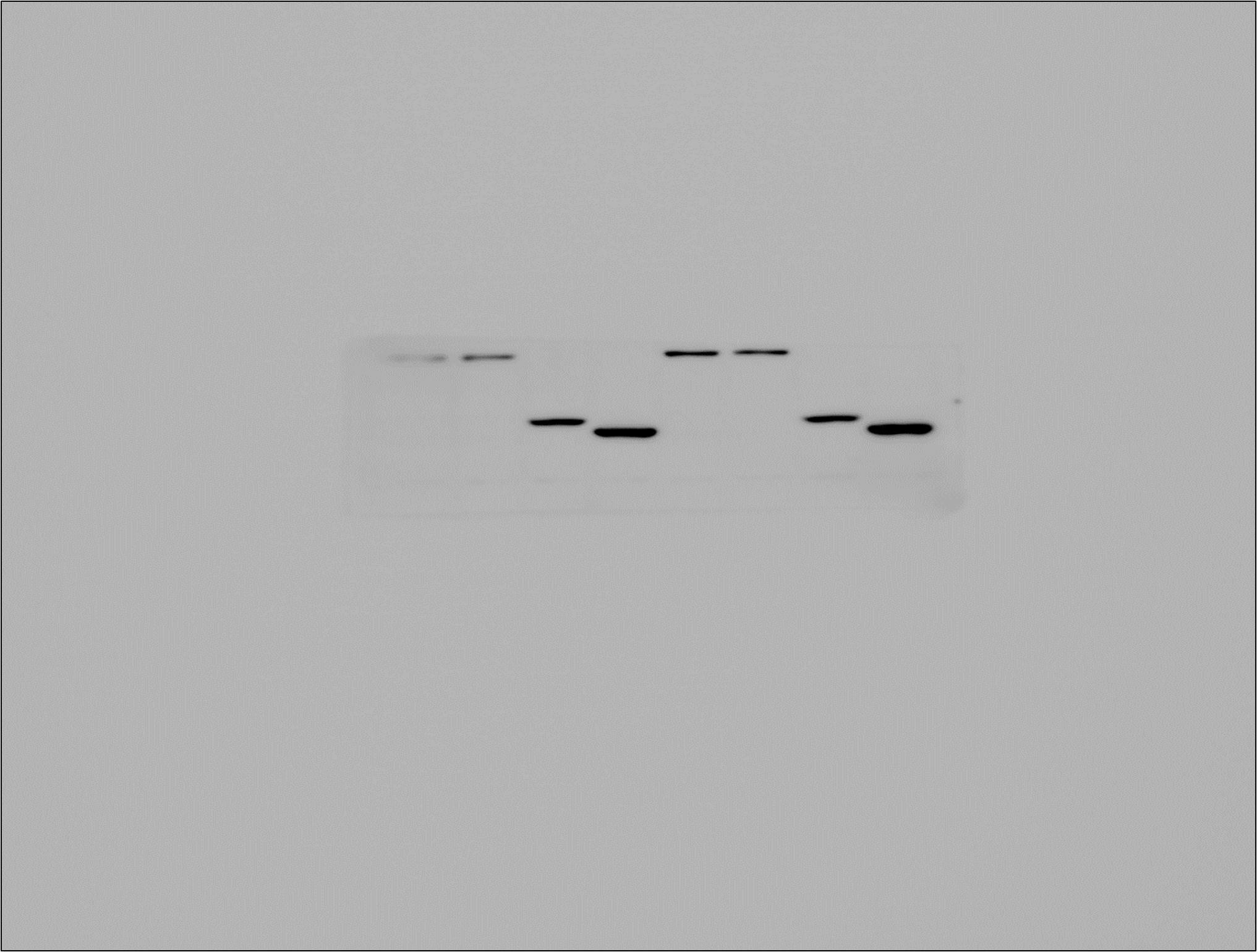

Supplement: Figure 7—source data 2. [file elife-98357-fig7-data2.zip › Figure 7-source data 2/7R-WCL-Flag-1.tif]

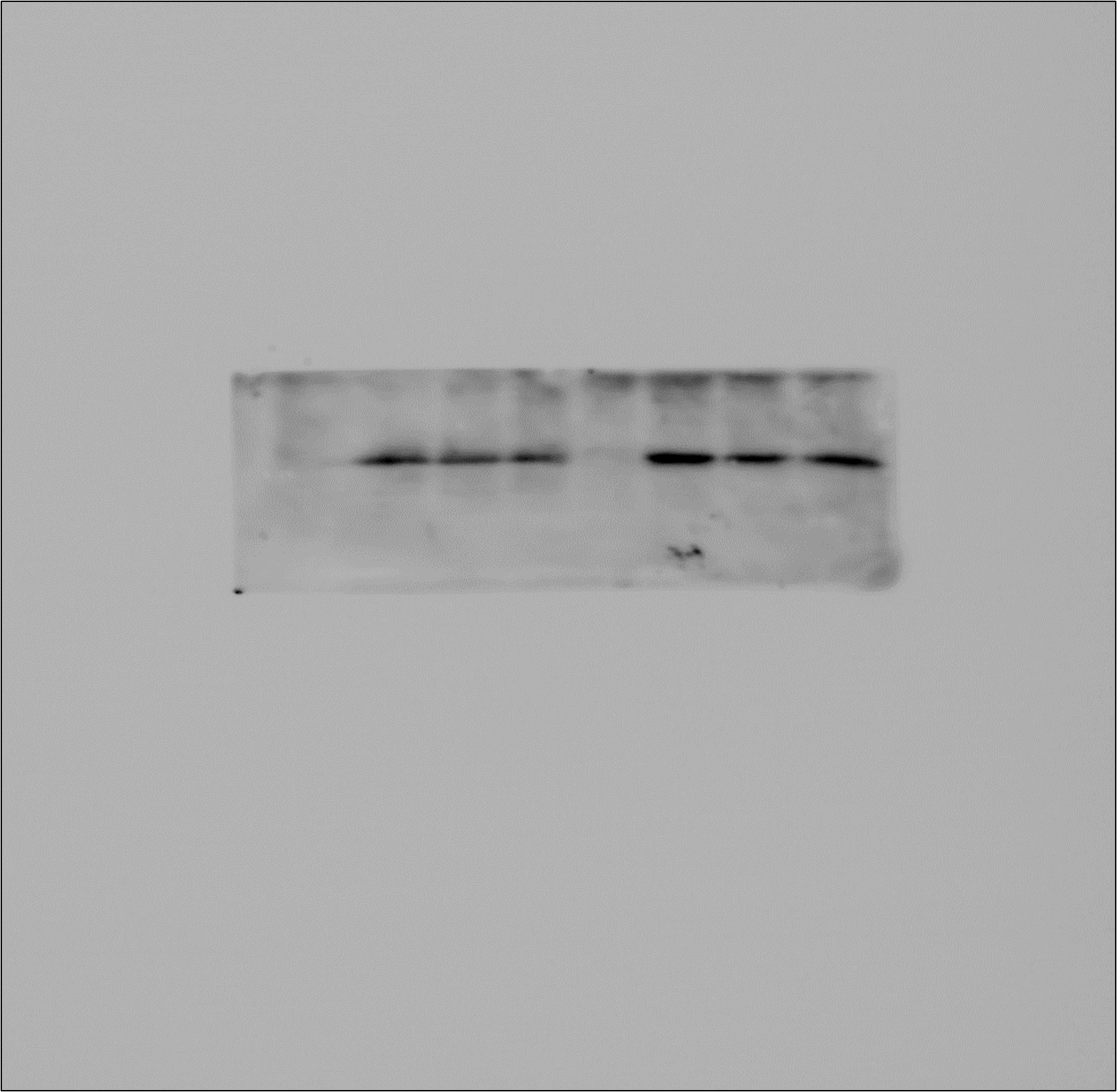

Supplement: Figure 7—source data 2. [file elife-98357-fig7-data2.zip › Figure 7-source data 2/7R-WCL-HA-1.tif]

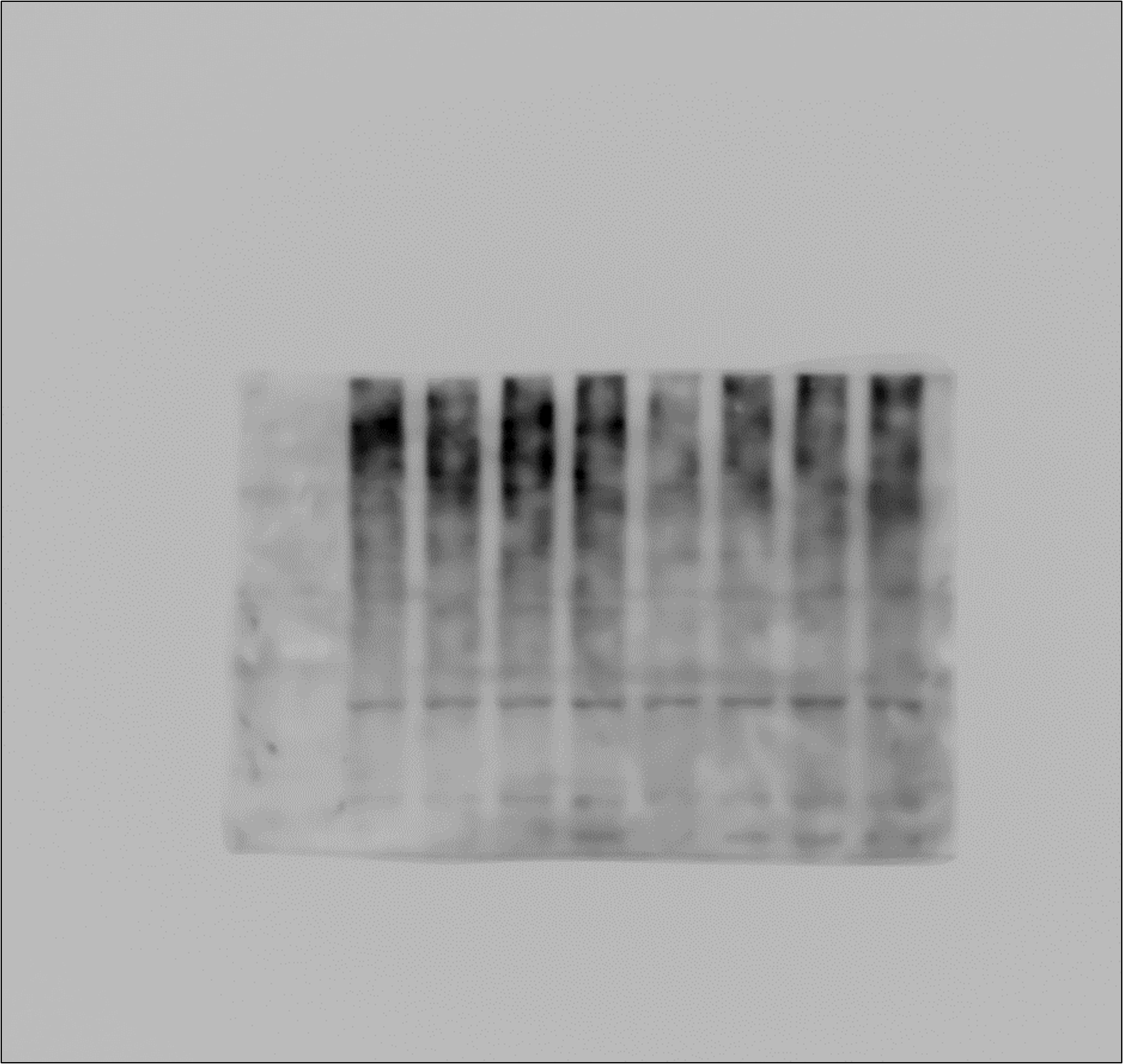

Supplement: Figure 7—source data 2. [file elife-98357-fig7-data2.zip › Figure 7-source data 2/7R-WCL-TBK1-HA-Ub-1.tif]
